# Identification and Expression Analyses of Olfactory Gene Families in the Rice Grasshopper, *Oxya chinensis*, From Antennal Transcriptomes

**DOI:** 10.3389/fphys.2019.01223

**Published:** 2019-09-26

**Authors:** Yang Cui, Cong Kang, Zhongzhen Wu, Jintian Lin

**Affiliations:** Guang Zhou City Key Laboratory of Subtropical Fruit Tree Outbreak Control, Zhongkai University of Agriculture and Engineering, Guangzhou, China

**Keywords:** antennal transcriptome, olfactory gene, identification, expression analysis, *Oxya chinensis*

## Abstract

The rice grasshopper *Oxya chinensis* is an important agricultural pest of rice and other gramineous plants. Chemosensory genes are crucial factors in direct interactions with odorants in the olfactory process. Here we identified genes encoding 18 odorant-binding proteins (OBPs), 13 chemosensory proteins (CSPs), 94 olfactory receptors (ORs), 12 ionotropic receptors (IRs), and two sensory neuron membrane proteins (SNMPs) from *O. chinensis* using an transcriptomic approach. Semi-quantitative RT-PCR assays revealed that six OBP-encoding genes (*OchiOBP4*, *5*, *8*, *9*, *10*, and *14*), one CSP gene (*OchiOBP10*) and two IR genes (*OchiIR28* and *29*) were exclusively expressed in antennae, suggesting their roles in olfaction. Real-time quantitative PCR analyses revealed that genes expressed exclusively or predominantly in antennae also displayed significant differences in expression levels between males and females. Among the differentially expressed genes, 17 OR-encoding genes, one CSP- and one SNMP-gene showed female-biased expression, suggesting that they may be involved in some female-specific behaviors such as seeking oviposition site; whereas the three remaining OR-encoding genes showed male-biased expression, indicating their possible roles in sensing female sex pheromones. Our results laid a solid foundation for future studies to reveal olfactory mechanisms as well as designing strategies for controlling this rice pest.

## Introduction

Olfaction plays an important role in regulating various physiological behaviors, such as foraging, mating, oviposition and avoiding predators (Leal, 2013). The process of olfactory perception is mediated by a series of peripheral olfactory proteins involving odorant-binding proteins (OBPs), chemosensory proteins (CSPs), sensory neuron membrane proteins (SNMPs), olfactory receptors (ORs), gustatory receptors (GRs), and ionotropic receptors (IRs) (Clyne et al., 1999, 2000; Vosshall et al., 1999; Galindo and Smith, 2001; Benton et al., 2007, 2009; Vieira and Rozas, 2011). OBPs and CSPs are suggested to be involve in binding and trafficking of hydrophobic odorant molecules across the sensillum lymph surrounding the olfactory sensory neurons (OSNs) on the sensilla of antennae and other chemosensory organs (Vogt et al., 1985; Sandler et al., 2000; Xu et al., 2005; Gomez-Diaz et al., 2013). ORs and GRs are the principle receptor proteins responsible for the detection of odorants and tastants, whereas IRs are involved in the detection of chemosensory, thermo- and hygro-sensory stimuli (Ai et al., 2010, 2013; Grosjean et al., 2011; Silbering et al., 2011; Kain et al., 2013; Su and Carlson, 2013; Koh et al., 2014; Chen et al., 2015; Stewart et al., 2015; Gorter et al., 2016; Hussain et al., 2016; Knecht et al., 2016, 2017; Ni et al., 2016). SNMPs play important roles in pheromone detection, which are expressed on the dendritic membranes of pheromone sensitive neurons (Rogers et al., 1997, 2001; Li et al., 2014; Jiang et al., 2016).

Gene families involved in olfactory perception have been extensively reported in many species in Lepidoptera, Coleoptera, Diptera, Hemiptera, and Hymenoptera during recent decades (Grosse-Wilde et al., 2011; Andersson et al., 2013, 2014; Brand et al., 2015; Paula et al., 2016). However, in the Orthoptera order, ORs have been identified from only three species: the genome of *Locusta migratoria* (142 ORs) (Wang et al., 2015), the antennal transcriptome of *Schistocerca gregaria* (119 ORs) (Pregitzer et al., 2017), and *Oedaleus asiaticus* (60 ORs) (Zhou et al., 2019). Members of the IR families have been identified in *L. migratoria* (32 IRs) (Wang et al., 2015) and *O. asiaticus* (6 IRs) (Zhou et al., 2019), the OBP families in *S. gregaria* (14 OBPs) (Jiang et al., 2017), *Oedaleus infernalis* (18 OBPs) (Zhang et al., 2018) and *O. asiaticus* (15 OBPs) (Zhou et al., 2019), the CSP families in *L. migratoria* (58 CSPs) and *S. gregaria* (42 CSPs) (Martin-Blazquez et al., 2017), and SNMP families in *S. gregaria* (2 SNMPs) (Jiang et al., 2016) and *O. asiaticus* (3 SNMPs) (Zhou et al., 2019). Consequently, additional Orthoptera species need to be investigated to reach a better understanding on olfactory receptive mechanisms for insect olfactory system.

*Oxya chinensis* is an oligophagous grasshopper pest and primarily feeds on graminaceous grasses. Electroantennogram (EAG) and behavioral bioassay have showed that *O. chinensis* adults have significantly higher olfactory sensitivity to geraniol compared to other host volatiles (Lu et al., 2008). Identification of target genes that are involved in olfactory perception may lead to environmentally-friendly approaches for controlling this pest (Venthur and Zhou, 2018). The objectives of this study were to generate antennal transcriptomes to identify major olfactory-related gene families (OBPs, CSPs, ORs, IRs, and SNMPs) from *O. chinensis*, examine expression profiles of the genes in various tissues to identify the antenna-specific genes, and compare their sex-specific expression patterns to identify female- and male-specific olfactory-related genes.

## Materials and Methods

### Insects, Tissue Collections, and RNA Extraction

The colony of *O. chinensis* used in this study was derived from insects originally collected from rice fields in the environs of Leizhou, Guangdong, China (110°05′E, 20°34′N). The colony was maintained in the laboratory on bristle grass in climate-controlled chamber under 30°C ± 1°C with 60% relative humidity and a photoperiod of 16 h light versus 8 h.

For transcriptomic analyses, 100 pairs of antennae were dissected separately from *O. chinensis* adult females and males. For semi-quantitative RT-PCR analysis, antennae, maxillary palps, foreleg tarsus, wings, and genitals were separately collected from adults (male: female = 1:1, *n* = 25 each). Two replicates were included for each tissue. For RT-qPCR analyses, 50 pairs of antennae were obtained from both females and males, separately along with other body parts including 20 pairs of maxillary palps, 20 pairs of foreleg tarsus, 20 pairs of wings, and 10 genitals from both males and females. Three replicates were included for each tissue sample. Tissues were homogenized to powder immediately in liquid nitrogen and stored at −80°C for further analyses.

Total RNA was extracted using Trizol reagent (Invitrogen, Carlsbad, CA, United States) and treated with RNase-free DNase I (Takara, Dalian, China) to remove potential genomic DNA contamination. RNA concentration, quality and quantity were analyzed on a NanoDrop ND-2000 Spectrophotometer (Nanodrop Technologies, United States) and a Qubit^®^2.0 Fluorometer (Invitrogen, Life Technologies, United States). RNA integrity was assessed with an Agilent 2100 Bioanalyzer (Agilent Technologies, United States).

### cDNA Library Construction, Illumina Sequencing and *de novo* Assembly

Transcriptomes were generated through a commercial contract with Novogene Bioinformatics Technology, Co., Ltd. (Beijing, China). Libraries for sequencing were generated from 1.5 μg purified RNA per sample using a TruSeq RNA Sample Preparation Kit v2 (Illumina, San Diego, CA, United States) according to Illumina instructions and sequenced on an Illumina HiSeq 2500 platform (San Diego, CA, United States). Approximately 150 bp paired-end reads were generated.

Raw reads were firstly processed through in-house perl scripts. High-quality clean reads were obtained by removing reads containing adapter and unknown (poly-N) and low-quality reads. Clean reads were combined from both female and male antennae and were assembled into unigenes using Trinity (Version: r2013-11-10) with min_kmer_cov set to 2 under default settings (Grabherr et al., 2011). The clean reads from the *O. chinensis* female antennae and male antennae were deposited in the NCBI Sequence Read Archive (Female antennae: SAMN11484273; Male antennae: SAMN11484274).

### Functional Annotation

Unigenes were searched against databases including NCBI non-redundant protein (nr), NCBI non-redundant nucleotide (nt), Swiss-Prot^1^, the Kyoto Encyclopedia of Genes and Genomes (KEGG)^2^, using BLASTx with a cut-of E-value of 10^–5^. Gene orthology (GO) and cluster of orthologous groups of proteins (COG)^3^ using Blast2GO program (Conesa et al., 2005; Gotz et al., 2008).

### Gene Identification

To identify genes coding for OBPs, CSPs, ORs, IRs, and SNMPs, known protein sequences (OBPs, CSPs, ORs, IRs, and SNMPs) from other Orthopteran species were selected as queries to search the *O. chinensis* antennal transcriptomes. *L. migratoria*, *S. gregaria*, *O. asiaticus*, *O. infernalis*, and *Ceracris kiangsu* were used for OBPs; *L. migratoria*, *S. gregaria*, and *O. asiaticus* for CSPs; *L. migratoria*, *S. gregaria*, and *O. asiaticus* for ORs; *L. migratoria* and *O. asiaticus* for IRs; *S. gregaria* and *O. asiaticus* for SNMPs (Supplementary Table S1). tBLASTn was used to search and identify candidate chemosensory genes against the *O. chinensis* antennal transcriptomes, with a cut-of E-value of 10^–5^. Putative *O. chinensis* chemosensory genes were in turn used as queries to identify additional genes (tBLASTx and BLASTp). Repetitions were completed until no new candidates were identified. Candidate unigenes from the initial search were further manually examined using BLASTx against Genbank. Open reading frames (ORFs) were predicted with the ORF Finder in NCBI^4^. Amino acid sequences were aligned with MAFFT (version 7.308) (E-INS-I parameter set) (Katoh and Standley, 2013) and visualized with Geneious (version 9.1.3) (Kearse et al., 2012). Transmembrane domains, signal peptides, conserved cysteine locations in candidates were analyzed using the InterProScan tool plug-in in Geneious (Version: 9.1.3.) (Quevillon et al., 2005). Candidate genes coding for OBPs, CSPs, ORs, IRs, and SNMPs were listed in Supplementary Table S2, together with genetic characteristics, best matches in NCBI-nr database, protein domains and estimated expression levels.

### Phylogenetic Analyses

Multiple amino acid sequence alignments were made with MAFFT (E-INS-I parameter) (Katoh and Standley, 2013). Phylogenetic trees were constructed using maximum likelihood analyses as implemented in FastTree2 [Jones-Taylor-Thornton (JTT) amino acid substitution model, 1,000 bootstrap replications] (Price et al., 2009, 2010). Dendrograms were created and colored in FigTree^5^. Sequences used for phylogenetic analysis are listed in Supplementary Table S3.

### Analyses of Expression Levels and Differentially Expressed Genes

Gene expression levels were estimated using RSEM (Version: 1.2.15) (Li and Dewey, 2011) for each sample. The clean reads were mapped back to the assembled transcriptome. The read counts normalized using the Trimmed Mean of *M*-value normalization method (Robinson and Oshlack, 2010) for each mapped gene was used to calculate gene expression levels following the FPKM (fragments per kilobase per million read) method (Cock et al., 2010; Trapnell et al., 2010), and were used to identify differentially expressed genes between females and males using DEGseq edgeR package (Version: 3.4.2) (Robinson et al., 2010). Criteria for estimating significantly differentially expressed genes were set as *q*-value < 0.005 and the absolute value of log2 Fold Change > 1.

### Semi-Quantitative RT-PCR and Real-Time Quantitative PCR

Semi-quantitative RT-PCR was carried out to compare expression levels of genes coding for OBPs, CSPs, and IRs in various tissues including antennae, maxillary palps, foreleg tarsus, wings and genitals. cDNA template was synthesized from total RNA using a PrimeScript RT reagent Kit (Takara, China). Two housekeeping genes, β-actin (*Ochi*β*-actin*) and ribosomal protein 49 (*Ochirp49*) were used as controls. PCR reactions were conducted in a thermal cycler from Bio-Rad (CA, United States). PCR conditions were as follows: 95°C for 2 min, followed by 35 cycles of 95°C for 30 s, 56°C for 30 s, 72°C for 1 min, and a final extension for 10 min at 72°C. PCR products were separated on 1.5% agarose gels. Individual PCR reactions were repeated twice with independently isolated RNA samples.

Expression profiles of the antenna-predominant candidate genes for OBPs, CSPs, and IRs as well as all ORs and SNMPs were analyzed on a LightCycler 480 system (Roche Applied Science). RT-qPCR cycling parameters were set at 95°C for 5 min, followed by 45 cycles of 95°C for 10 s, and 60°C for 20 s. A melting curve analysis was then performed at 95°C for 20 s, 60°C for 30 s, and 95°C for 30 s in order to determine the specificity of primers. Negative controls without cDNA template (nuclease-free water) were included for each reaction. Three independent biological replications were performed with each biological replication measured in three technique replications. The results were analyzed using the LightCycler 480 Gene Scanning Software. Relative quantification was calculated following the 2^–ΔΔ*CT*^ method (Livak and Schmittgen, 2001), and normalized against the two reference genes (*Ochi*β*-actin* and *Ochirp49*). Gene-specific primers were designed using Primer3^6^ and listed in Supplementary Table S4.

### Statistical Analysis

RT-qPCR data were analyzed and plotted using Prism 6.0 (GraphPad Software, CA, United States). The comparative analyses of each target gene among various tissues were determined using a one-way nested analysis of variance (ANOVA) followed by Duncan’s new multiple range test (α = 0.05) using SPSS 22.0 (SPSS Inc., Chicago, IL, United States). Values are presented as mean ± SE.

## Results

### Illumina Sequencing and *de novo* Assembly

A total of 53.4 and 44.8 million raw reads were obtained from male and female antennae cDNA libraries of *O. chinensis*, respectively. After removing adaptor sequences, low quality reads and contaminant sequences, 52.2 and 44.7 million of clean reads were generated. The reads were assembled into 120,803 unigenes with a mean length of 1,213 bp, and an N50 of 2,458 bp. The number of unigenes longer than 500 bp was 33,079, which accounted for 35.21% of all unigenes (Supplementary Table S5).

### Functional Annotation

There were 26,923 (45.5%), 15,092 (25.5%), 20,546 (34.7%), 5,662 (9.57%), 10,911 (18.4%), and 20,542 (34.7%) unigenes that had homologous sequences in NCBI-nr, NCBI-nt, Swiss-Prot, GO, COG, and KEGG databases, respectively. Based on the homologous sequences, a total of 29,067 (49.1%) unigenes were annotated and the remaining unigenes were unmappable at present (Supplementary Figure S1A). GO analyses categorized annotated genes into three functional categories, including “biological process,” “cellular component,” and “molecular function” (Supplementary Figure S1B). In “biological process,” major subcategories included “cellular,” “metabolic” and “single-organism.” In “cellular component,” the subcategories “cell” and “cell part” and “membrane” were major subcategories. In “molecular function,” the subcategories “binding” and “catalytic activity” contained the largest numbers of unigenes.

### Candidate Genes Coding for Odorant Binding Proteins

**FIGURE 1:**
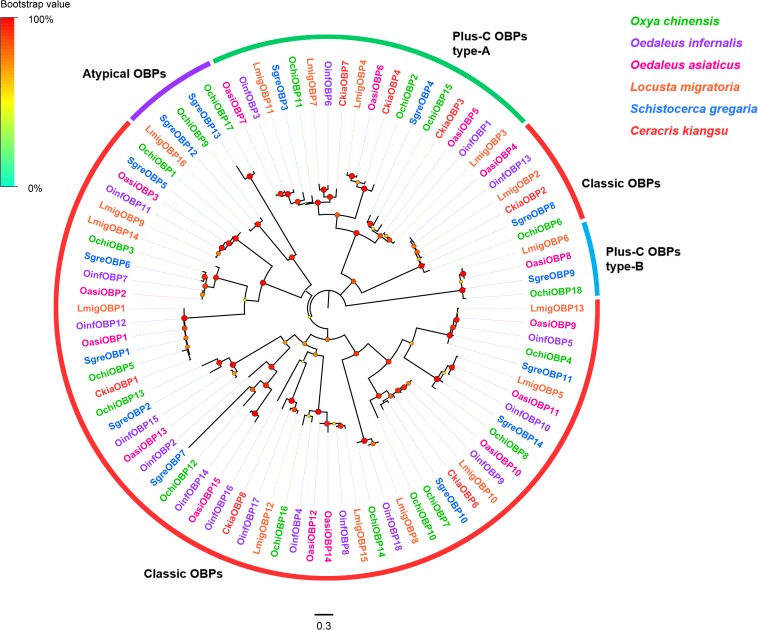
Phylogenetic tree of putative odorant-binding proteins (OBPs). Branch support (circles at the branch nodes) was estimated using an approximate likelihood ratio test based on the scale indicated on the top left. Bars indicate branch lengths in proportion to amino acid substitutions per site. Classic OBPs are in red; plus-C OBPs type-A in green; plus-C OBPs type-B in blue; and atypical OBPs in purple.

Eighteen OBP unigenes were identified from the *O. chinensis* antennal transcriptomes (Supplementary Table S2: Sheet 1). All identified unigenes except *OchiOBP18* had a full-length ORF with sizes ranged from 128 to 266 amino acid residues. Except three OBP genes (*OchiOBP12*, *14*, and *17*), all predicted proteins had a predicted signal peptide. Sequence identities of predicted OBPs with those from other Orthopteran insects in the NCBI-nr database ranged from 45.9 to 88.6%, with an average of 73.8%. Consistent with previous Orthopteran OBP classification by Jiang et al. (2017), twelve OBPs (*OchiOBP1*, *3-8*, *10*, *12-14*, *16*) were classic OBPs, with the typical six conserved C-residues (Supplementary Figure S2). Two (*OchiOBP9* and *17*) were atypical OBPs, with extraordinary long stretches between conserved C1 and C2 (Supplementary Figure S3). Four OBPs were plus-C OBPs, which were further divided into plus-C OBP type-A (*OchiOBP2*, *11*, and *15*) (with the extra C-residue both in C4-C5 and C5-C6) (Supplementary Figure S4) and plus-C OBP type-B (*OchiOBP18*) (with the extra C-residue in front of C1 and behind C6) (Supplementary Figure S5). Phylogenetic analyses of OBPs from *O. chinensis* and other five locust species (*O. infernalis*, *O. asiaticus*, *L. migratoria*, *S. gregaria*, and *C. kiangsu*) showed that all the locust OBPs formed distinct clades based on the number and position of cysteine residues, and segregated into the classic OBP, atypical OBP, and plus-C OBP sub-families as well as other locust OBPs (Figure 1).

### Candidate Genes Coding for Chemosensory Proteins

**FIGURE 2:**
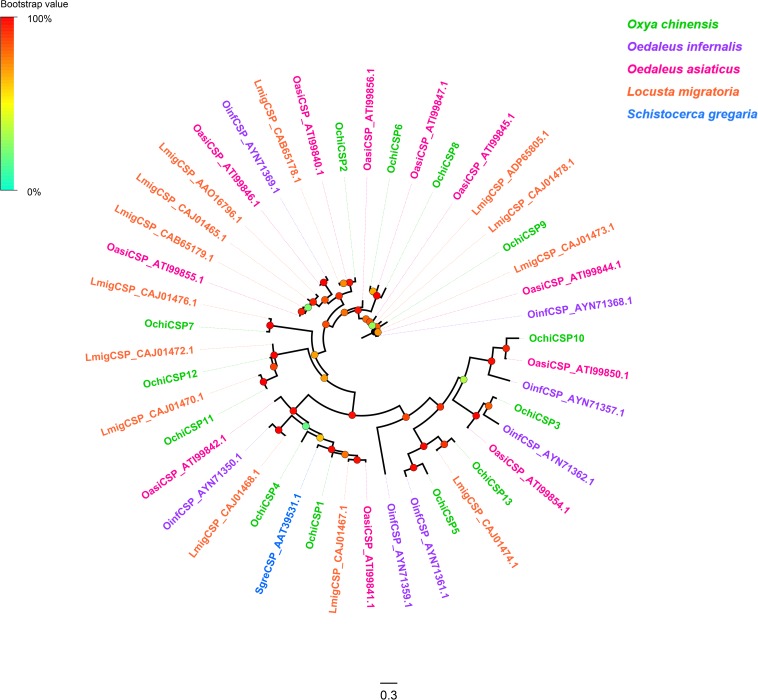
Phylogenetic tree of putative chemosensory proteins (CSPs). Branch support (circles at the branch nodes) was estimated using an approximate likelihood ratio test based on the scale indicated at the top left. Bars indicate branch lengths in proportion to amino acid substitutions per site.

Thirteen unigenes encoding CSPs were identified from the *O. chinensis* antennal transcriptomes (Supplementary Table S2: Sheet 2). All but two (*OchiCSP11* and *13*) had full-length ORFs encoding 120–297 amino acid residues. All predicted OchiCSPs contained four highly conserved four-cysteine profiles (Supplementary Figure S6). Except *OchiCSP1*, all predicted proteins possessed a signal peptide. A phylogenetic tree was built using all predicted OchiCSPs together with those from other locust species. Most OchiCSPs had orthologs with CSPs from other locust species, and no *O. chinensis*-specific CSP lineage was evident (Figure 2).

### Candidate Genes Coding for Odorant Receptors

**FIGURE 3:**
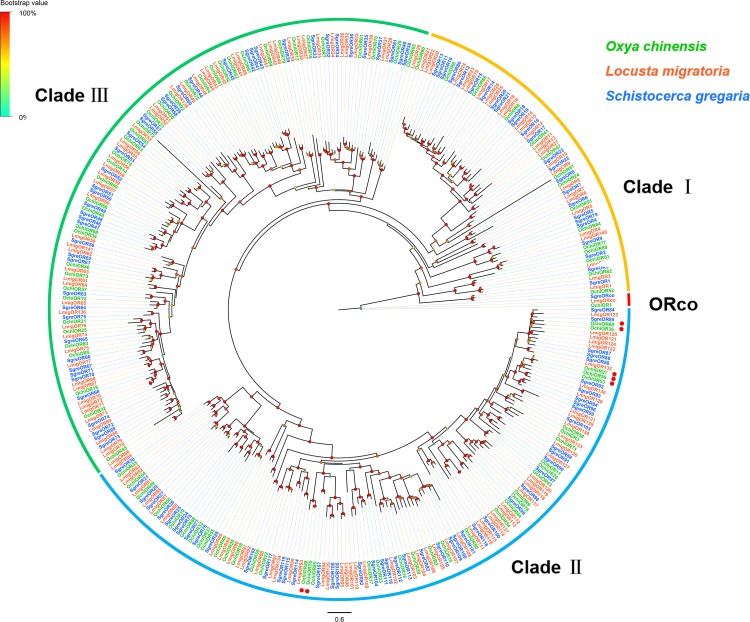
Phylogenetic tree of putative odorant receptors (ORs). The distance tree was rooted by the conservative ORco gene orthologs (red). The species- specific clades and sister pairs are labeled with red dots. Branch support (circles at the branch nodes) was estimated using an approximate likelihood ratio test based on the scale indicated at the top left. Bars indicate branch lengths in proportion to amino acid substitutions per site. The clade I is in orange; clade II in green; and clade III in blue.

Ninety-four OR-encoding unigenes were identified, including one Orco (*OchiOR1*) and 93 conventional OR genes (*OchiOR2-94*), which were classified to the 7-transmembrane receptor superfamily (Supplementary Table S2: Sheet 1). Among these OR unigenes, 54 had full-length ORFs encoding proteins with 367 to 487 amino acid residues. The highly conserved co-receptor *OchiOR1* shared 94.25% identity to a co-receptor from *L. migratoria* (ALD51504.1), while other OchiORs shared 33.56 to 91.13% identity with average 68.7% with the respective *L. migratoria* ORs. A phylogenetic tree was generated using our identified ORs along with a data set containing representative ORs from *L. migratoria* and *S. gregaria* (Figure 3). The locust ORs formed three distinct clades, with the *O. chinensis* OR co-receptor (*OchiOR1*) formed a clade with the protein Orco from two other related species. The vast majority of OchiORs were clustered with orthologs from other locust species. Only a few species-specific clades and sister pairs were observed. Three OchiORs (*OchiOR27*, *53* and *56*) were segregated into unique clades, while *OchiOR36*/*60* and *OchiOR28*/*76* formed the sister pairs.

### Candidate Genes Coding for Ionotropic Receptors (iGluRs/IRs)

**FIGURE 4:**
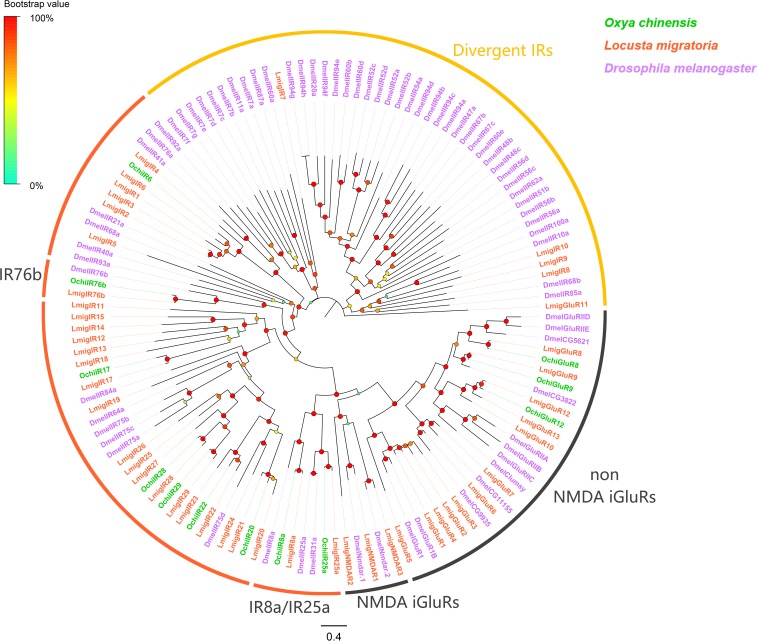
Phylogenetic tree of putative ionotropic receptors (IRs). Branch support (circles at the branch nodes) was estimated using an approximate likelihood ratio test based on the scale indicated at the top left. Bars indicate branch lengths in proportion to amino acid substitutions per site.

Twelve putative iGluR/IR unigenes were identified, which were predicted to encode ligand-binding domain (S1 and S2) with three transmembrane domains (M1, M2, and M3) or portions of domains (Supplementary Table S2: Sheet 2). Of these iGluR/IR unigenes, eight had complete ORFs encoding at least 535 amino acid residues. Distinct clades were observed in a phylogenetic tree generated with our identified sequences and paralogs from other species including *Drosophila melanogaster* and *L. migratoria* (Figure 4). All identified iGluRs/IRs from *O. chinensis* were assigned to two phylogenetic groups, including N-Methyl-D-aspartic acid (NMDA) iGluRs (*OchiGluR8*, *9*, and *12*) and antennal IRs (*OchiIR25a*, *8a*, *76b*, *22*, *6*, *17*, *20*, *28*, and *29*). No candidates were assigned to non-NMDA iGluRs and divergent IRs (Figure 4). A set of “antennal IR” conserved among other species were absent from *O. chinensis* and *L. migratoria*, and only three “IR co-receptor” orthologs (*OchiIR25a*, *8a*, and *76b*) were clustered with *D. melanogaster* orthologs.

### Candidate Genes Coding for Sensory Neuron Membrane Proteins

**FIGURE 5:**
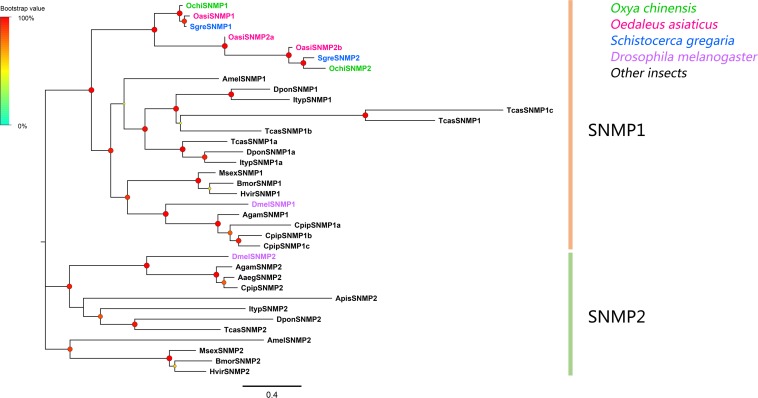
Phylogenetic tree of putative sensory neuron membrane proteins (SNMPs). Branch support (circles at the branch nodes) was estimated using an approximate likelihood ratio test based on the scale indicated at the top left. Bars indicate branch lengths in proportion to amino acid substitutions per site.

Two SNMP unigenes were identified, which matched the CD36 family. The genes contained complete ORFs that encoded proteins with two transmembrane domains (Supplementary Table S2: Sheet 3). Phylogenetic analyses revealed that all SNMPs were classified into two distinct subgroups, SNMP1 and SNMP2 (Figure 5). In the SNMP1 clade, *OchiSNMP1* and *OchiSNMP2* were clustered into two subclades along with those from other locust species.

### Transcript Abundance Based on FPKM

In this study, genes with FPKM values ≥1,000 were defined as highly expressed genes, those with values 300–1,000 were defined as moderately expressed genes, and those with values ≤300 were defined as weakly expressed genes. Based on these criteria, *OchiOBP1-5* and *OchiCSP1-4* were highly expressed in antennae (Supplementary Table S2: Sheets 1,2); *OchiOR1* (Orco) was moderately expressed (Male FPKM: 318.52, Female FPKM: 391.73); other conventional ORs (*OchiOR2-94*), all iGluRs/IRs, and the two SNMPs were weakly expressed (RPKM ≤ 30) (Supplementary Table S2: Sheet 3). DEG analyses showed that *OchiCSP10* and *OchiOR2*, *6*, *8*, *9*, *11*, *15*, *33*, *38*, *39*, *41*, *43*, *44*, *48*, *50*, *53*, *56*, and *62* were female-predominant, whereas *OchiCSP9*, *OchiOR34*, *46* and *86*, and *OchiGluR8* and *12* were male-biased (Supplementary Table S2: Sheets 1–5).

### Tissue- and Sex-Specific Expression

**FIGURE 6:**
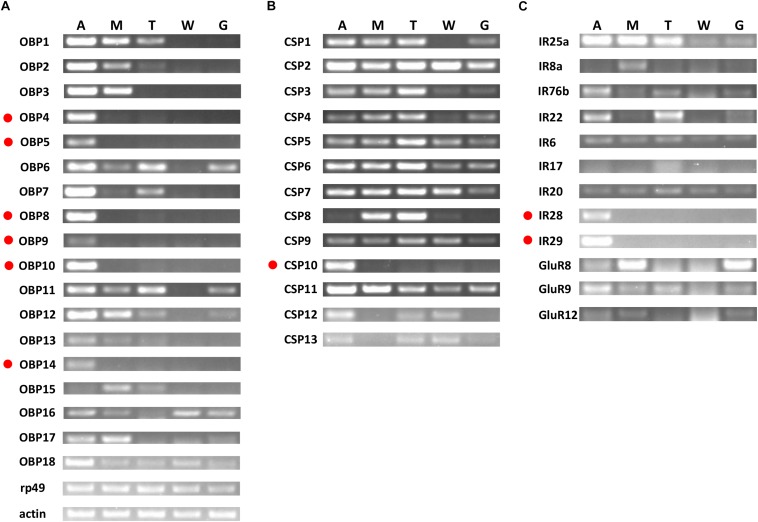
Tissue-specific expression of *OchiOBP*s, *OchiCSP*s, and *OchiIR*s. **(A)** OBPs. **(B)** CSPs. **(C)** IRs. Genes exclusively or predominantly expressed in antennae are labeled in red dots. The β-actin (*Ochi*β*-actin*) and ribosomal protein 49 (*Ochirp49*) were used as internal references to test the integrity of each cDNA template. An, antennae; M, maxillary palps; T, foreleg tarsus; W, wings; G, genitals.

Based on semi-quantitative RT-PCR analyses, *OchiOBP4*, *5*, *8*, *9*, *10*, and *14* were almost exclusively expressed in antennae, while *OchiOBP3* and *17* were abundant in antennae and maxillary palps (Figure 6A). The remaining OBP-encoding genes were abundant in multiple body parts. For CSP-encoding genes, *OchiCSP10* was exclusively expressed in antennae, while other *OchiCSPs* were present multiple body parts (Figure 6B). For iGluR/IR-encoding genes, *OchiIR28* and *29* were expressed predominately in antennae (Figure 6C).

**FIGURE 7:**
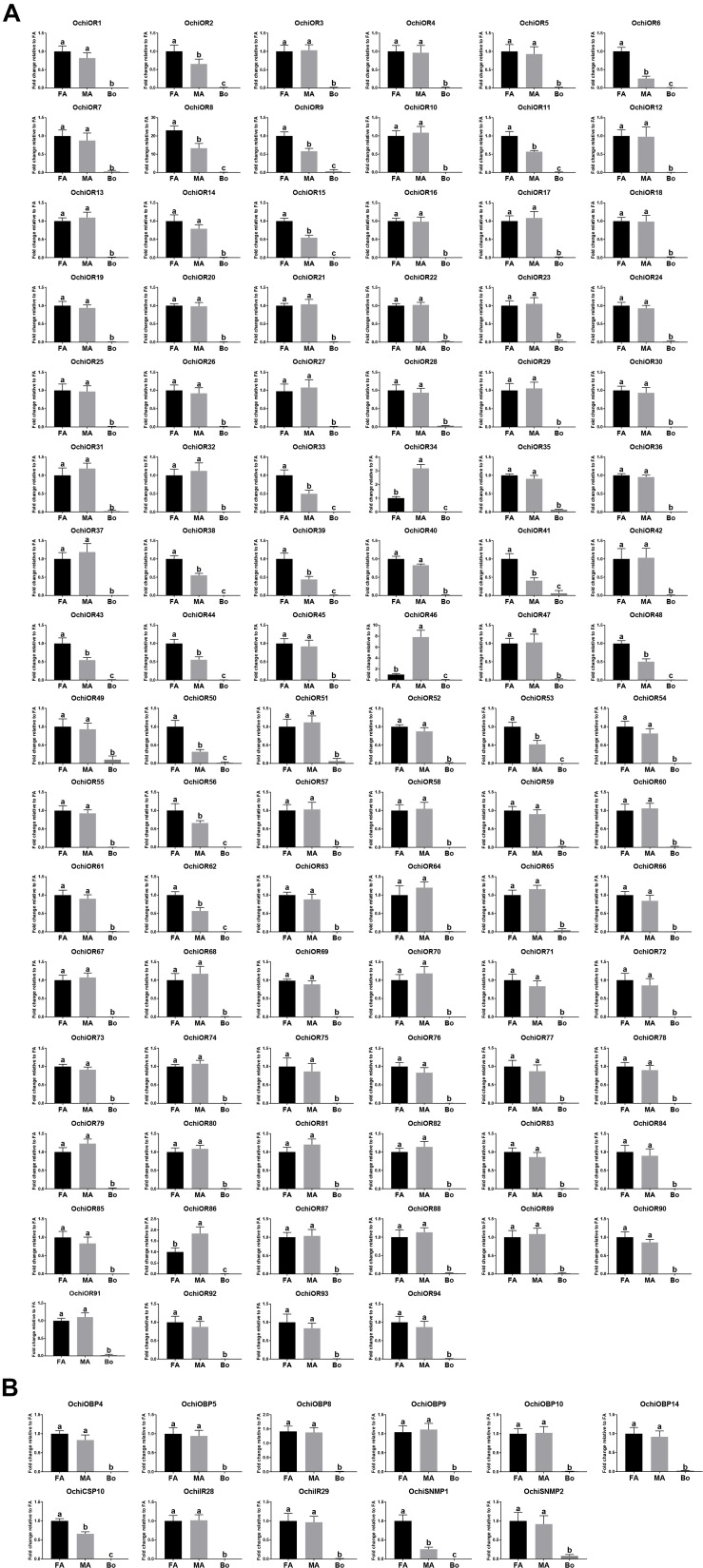
Relative expression levels of the olfactory-related genes in the female antennae, male antennae, and other body parts from *Oxya chinensis*. **(A)** ORs. **(B)** SNMPs and the antenna-predominant candidates (OBPs, CSPs, and IRs). FA, female antennae; MA, male antennae; Bo, other body parts. The expression levels were estimated using the 2^–ΔΔCT^ method. Bars represent standard error of three independent biological replicates with three technical duplicates for each replicate. Different small letters indicate statistically significant difference between tissues (*p* < 0.05, ANOVA, HSD).

All antenna-predominant genes were further analyzed using RT-qPCR (Figure 7). *OchiOR2*, *6*, *8*, *9*, *11*, *15*, *33*, *38*, *39*, *41*, *43*, *44*, *48*, *50*, *53*, *56*, and *62* were significantly expressed at high levels in female antennae and *OchiOR34*, *46* and *86* were significantly expressed at higher levels in male antennae. The remaining OR-encoding unigenes were equally expressed in the antennae of both males and females (Figure 7A). For CSP-encoding genes, *OchiCSP10* was expressed at significantly higher levels in female antennae. Additionally, the SNMP-encoding gene *OchiSNMP1* were significantly upregulated in female antennae (Figure 7B). Other OBP- and IR-encoding genes were expressed largely equally in both males and females (Figure 7B).

## Discussion

Identification and characterization of chemosensory genes are important steps toward understanding the evolution and primary functions of the insect olfactory system. In this study, we identified a set of candidate chemosensory genes from *O. chinensis* via analyzing antennal transcriptomes. Genetic and phylogenetic analyses of candidate chemosensory genes in *O. chinensis* was carried out to examine the similarities and differences of molecular components in chemosensory pathways. We further analyzed the expression profiles of chemosensory genes to identify olfaction-specific genes for future functional studies.

Odorant-binding proteins play vital roles in carrying odorants through the hemolymph to OR neurons, and transducing the resultant signals to downstream effectors in the olfactory system (Hallem et al., 2006). The number of OBPs identified in our antennal transcriptome was comparable to its related Locust species, namely 18 from *O. infernalis*, 16 from *L. migratoria*, 15 from *O. asiaticus*, and 14 from *S. gregaria*) (Ban et al., 2003; Xu et al., 2009; Yu et al., 2009; Zhang et al., 2015, 2018; Jiang et al., 2017). This may reflect physiological and evolutionary consistency between *O. chinensis* and other Locust species. OBPs from *O. chinensis* can be classified into four types: classic, atypical, plus-C (type-A and B), as well as Locust-specific OBPs (Jiang et al., 2017). Expression analysis revealed that five classic OBP (*OchiOBP4*, *5*, *8*, *10*, and *14*) and one atypical OBP (*OchiOBP9*) were expressed exclusively in antennae, suggesting that these genes are likely to have a role in antennal chemical-recognition processes. CSPs and OBPs can act as carriers for odorant molecules (Liu et al., 2012; Yi et al., 2013; Zhang T. et al., 2013; Zhang et al., 2014). However, in our RT-PCR analysis, only *OchiCSP10* showed significantly antennae-biased expression, suggesting that this CSP could be involved in olfactory function.

Olfactory receptors, which connect binding proteins and OSNs to transduce olfactory signals, are the best known group of insect chemoreceptors. Like other locust species, *O. chinensis* possesses an expanded OR family (94) compared with Dipterans, Lepidopterans, and Hemipterans (Vieira and Rozas, 2011). A large number of OR-encoding genes may be associated with its ability for diverse host-odor perception to feed on a wider range of host plants. Great variation in the number of OR genes was found in different Locust species. Specifically, 94 OR genes were identified in *O. chinensis*, less than 120 from *S. gregaria* (Pregitzer et al., 2017), and 141 from *L. migratoria* (Wang et al., 2015), but significantly more than 60 from *O. asiaticus* (Zhou et al., 2019). The variation in gene numbers may reflect that *O. chinensis* inhabits a different ecological niche than other locust species. Generally, sexually dimorphic expression of ORs in antennae indicates possible pheromone receptors contributing sexual behaviors. Typically, Lepidopteran sex pheromones are released via females to attract males. Several moth sex pheromone for ORs have been functionally characterized, and most of them are expressed at higher levels in the male antennae (Krieger et al., 2004; Wanner et al., 2010; Zhang and Löfstedt, 2013). ORs expressed predominantly in female antennae are predicted to function in the detection of egg-laying-related odorant (Pelletier et al., 2010) or male released pheromones (or signals) (Anderson et al., 2009); ORs expressed evenly in the male and female antennae are predicted to function in general odorant perception (Yan et al., 2015). Therefore, we hypothesize that some or all of female-predominant OchiORs, including *OchiOR2*, *6*, *8*, *9*, *11*, *15*, *33*, *38*, *39*, *41*, *43*, *44*, *48*, *50*, *53*, *56*, and *62*), are likely involved in female specific behaviors such as finding plant hosts for oviposition. Male-predominant ORs (*OchiOR34*, *46*, and *86*) may be associated with detecting female sex pheromones. The remaining ORs with roughly equal expression levels in female and male antennae might be dedicated to general odorant detection.

Ionotropic receptors are conserved proteins and play key roles in the synaptic ligand-gated ion channels involved in olfaction, gustation, thermosensation and hygrosensation (Min et al., 2013; Zhang Y.V. et al., 2013; Koh et al., 2014; Chen et al., 2015; Stewart et al., 2015; Croset et al., 2016; Gorter et al., 2016; Hussain et al., 2016; Knecht et al., 2016, 2017; Ni et al., 2016; Prieto-Godino et al., 2016; Ganguly et al., 2017; Tauber et al., 2017). We identified nine antennal IR genes (*OchiIR25a*, *8a*, *76b*, *22*, *6*, *17*, *20*, *28*, and *29*) and three iGluRs (*OchiGluR8*, *9*, and *12*) from *O. chinensis*. In general, most antennal IRs were conserved across orders Diptera, Lepidoptera and Coleoptera, but only three IR co-receptor orthologs (*OchiIR25a*, *8a*, and *76b*) were found in *O. chinensis*, and there were no antennal IRs orthologs with *D. melanogaster* (Benton et al., 2009). In *D. melanogaster*, antennal IRs function as chemoreceptors (Benton et al., 2009) and are expressed in the peripheral olfactory neurons associated with the detection of amines, acids, general odors and sex pheromones (Ai et al., 2010; Silbering et al., 2011; Kain et al., 2013; Koh et al., 2014; Hussain et al., 2016). Notably, we found that two antennal IR genes (*OchiIR28* and *29*) were exclusively expressed in antennae, suggesting they potentially involved in odorant reception.

We also identified two SNMP unigenes. SNMPs are conserved throughout holometabolous insects and the SNMP1 subfamily are usually expressed in pheromone-sensitive OSNs, and mediate responses to lipid pheromones (Jin et al., 2008; Nichols and Vogt, 2008; Vogt et al., 2009; Gomez-Diaz et al., 2016). All SNMPs from locust species were clustered into the SNMP1 subclade. Both SNMPs were predominantly expressed in antennae in *O. chinensis*, supporting that these two SNMPs may perform functions in pheromone perception. Interestingly, *OchiSNMP1* was predominantly expressed in female antennae (Figure 7B), suggesting that *OchiSNMP1* may participate in sex pheromone reception.

## Data Availability Statement

The datasets generated for this study can be found in the clean reads from the *O. chinensis* female antennae and male antennae were deposited in the NCBI Sequence Read Archive (Female antennae: SAMN11484273; Male antennae: SAMN11484274).

## Author Contributions

YC and CK performed the experiments. ZW analyzed the data. ZW and JL wrote and revised the manuscript.

## Conflict of Interest

The authors declare that the research was conducted in the absence of any commercial or financial relationships that could be construed as a potential conflict of interest.

## Footnotes

1 http://www.ebi.ac.uk/uniprot

2 http://www.genome.jp/kegg/

3 http://clovr.org/docs/clusters-of-orthologous-groups-cogs/

4 https://www.ncbi.nlm.nih.gov/orffinder/

5 http://tree.bio.ed.ac.uk/software/figtree

6 http://primer3.ut.ee/

## Supplementary Material

FIGURE S1 Transcriptome overview of *O. chinensis* antennae. **(A)** Unigenes annotated through different databases. **(B)** Gene ontology (GO) classification of the *O. chinensis* unigenes.

FIGURE S2 The amino acid alignment of the predicted classic OBPs.

FIGURE S3 The amino acid alignment of the predicted atypical OBPs.

FIGURE S4 The amino acid alignment of the predicted plus-C OBPs type-A.

FIGURE S5 The amino alignment of the predicted plus-C OBPs type-B.

FIGURE S6 The amino alignment of the predicted CSPs.

TABLE S1 List of OBPs, CSPs, ORs, IRs, and SNMPs in Orthopteran species.

TABLE S2 A list of the identified OBPs, CSPs, ORs, IRs and SNMPs in *O. chinensis*, and detailed genetic characteristics, best matches in NCBI-nr database, protein domains and estimated expression levels found in each petal module.

TABLE S3 The amino acid sequences used for phylogenetic analyses.

TABLE S4 Gene-specific primers used for semi-quantitative RT-PCR and RT-qPCR.

TABLE S5 Overview of the sequencing and assembly process.

## References

[B1] Ai M. Blais S. Park J. Y. Min S. Neubert T. A. Suh G. S. (2013). Ionotropic glutamate receptors IR64a and IR8a form a functional odorant receptor complex in vivo in Drosophila. *J. Neurosci.* 33 10741–10749. 10.1523/JNEUROSCI.5419-12.2013 23804096 PMC3693055

[B2] Ai M. Min S. Grosjean Y. Leblanc C. Bell R. Benton R. (2010). Acid sensing by the Drosophila olfactory system. *Nature* 468 691–695. 10.1038/nature09537 21085119 PMC3105465

[B3] Anderson A. R. Wanner K. W. Trowell S. C. Warr C. G. Jaquin-Joly E. Zagatti P. (2009). Molecular basis of female-specific odorant responses in Bombyx mori. *Insect Biochem. Mol. Biol.* 39 189–197. 10.1016/j.ibmb.2008.11.002 19100833

[B4] Andersson M. N. Grosse-Wilde E. Keeling C. I. Bengtsson J. M. Yuen M. M. Li M. (2013). Antennal transcriptome analysis of the chemosensory gene families in the tree killing bark beetles, *Ips typographus* and *Dendroctonus ponderosae* (Coleoptera: Curculionidae: Scolytinae). *BMC Genomics* 14:198. 10.1186/1471-2164-14-198 23517120 PMC3610139

[B5] Andersson M. N. Videvall E. Walden K. K. Harris M. O. Robertson H. M. Löfstedt C. (2014). Sex- and tissue-specific profiles of chemosensory gene expression in a herbivorous gall-inducing fly (Diptera: Cecidomyiidae). *BMC Genomics* 15:501. 10.1186/1471-2164-15-501 24948464 PMC4230025

[B6] Ban L. Scaloni A. D’Ambrosio C. Zhang L. Yahn Y. Pelosi P. (2003). Biochemical characterization and bacterial expression of an odorant-binding protein from *Locusta migratoria*. *Cell Mol. Life Sci.* 60 390–400. 10.1007/s000180300032 12678502 PMC11138600

[B7] Benton R. Vannice K. S. Gomez-Diaz C. Vosshall L. B. (2009). Variant ionotropic glutamate receptors as chemosensory receptors in Drosophila. *Cell* 136 149–162. 10.1016/j.cell.2008.12.001 19135896 PMC2709536

[B8] Benton R. Vannice K. S. Vosshall L. B. (2007). An essential role for a CD36-related receptor in pheromone detection in Drosophila. *Nature* 450 289–293. 10.1038/nature06328 17943085

[B9] Brand P. Ramírez S. R. Leese F. Quezada-Euan J. J. Tollrian R. Eltz T. (2015). Rapid evolution of chemosensory receptor genes in a pair of sibling species of orchid bees (Apidae: Euglossini). *BMC Evol. Biol.* 15:176. 10.1186/s12862-015-0451-9 26314297 PMC4552289

[B10] Chen C. Buhl E. Xu M. Croset V. Rees J. S. Lilley K. S. (2015). Drosophila ionotropic receptor 25a mediates circadian clock resetting by temperature. *Nature* 527 516–520. 10.1038/nature16148 26580016

[B11] Clyne P. J. Warr C. G. Carlson J. R. (2000). Candidate taste receptors in Drosophila. *Science* 287 1830–1834. 10.1126/science.287.5459.1830 10710312

[B12] Clyne P. J. Warr C. G. Freeman M. R. Lessing D. Kim J. Carlson J. R. (1999). A novel family of divergent seven-transmembrane proteins: candidate odorant receptors in Drosophila. *Neuron* 22 327–338. 10.1016/s0896-6273(00)81093-4 10069338

[B13] Cock P. J. Fields C. J. Goto N. Heuer M. L. Rice P. M. (2010). The sanger FASTQ file format for sequences with quality scores, and the Solexa/Illumina FASTQ variants. *Nucleic Acids Res.* 38 1767–1771. 10.1093/nar/gkp1137 20015970 PMC2847217

[B14] Conesa A. Gotz S. Garcia-Gomez J. M. Terol J. Talon M. Robles M. (2005). Blast2GO: a universal tool for annotation, visualization and analysis in functional genomics research. *Bioinformatics* 21 3674–3676. 10.1093/bioinformatics/bti610 16081474

[B15] Croset V. Schleyer M. Arguello J. R. Gerber B. Benton R. (2016). A molecular and neuronal basis for amino acid sensing in the Drosophila larva. *Sci. Rep.* 6:34871. 10.1038/srep34871 27982028 PMC5159833

[B16] Galindo K. Smith D. P. (2001). A large family of divergent Drosophila odorant-binding proteins expressed in gustatory and olfactory sensilla. *Genetics* 159 1059–1072. 11729153 10.1093/genetics/159.3.1059 PMC1461854

[B17] Ganguly A. Pang L. Duong V. K. Lee A. Schoniger H. Varady E. (2017). A molecular and cellular context-dependent role for Ir76b in detection of amino acid taste. *Cell Rep.* 18 737–750. 10.1016/j.celrep.2016.12.071 28099851 PMC5258133

[B18] Gomez-Diaz C. Bargeton B. Abuin L. Bukar N. Reina J. H. Bartoi T. (2016). A CD36 ectodomain mediates insect pheromone detection via a putative tunnelling mechanism. *Nat. Commun.* 7:11866. 10.1038/ncomms11866 27302750 PMC4912623

[B19] Gomez-Diaz C. Reina J. H. Cambillau C. Benton R. (2013). Ligands for pheromone-sensing neurons are not conformationally activated odorant binding proteins. *PLoS Biol.* 11:e1001546. 10.1371/journal.pbio.1001546 23637570 PMC3640100

[B20] Gorter J. A. Jagadeesh S. Gahr C. Boonekamp J. J. Levine J. D. Billeter J. C. (2016). The nutritional and hedonic value of food modulate sexual receptivity in *Drosophila melanogaster* females. *Sci. Rep.* 6:19441. 10.1038/srep19441 26777264 PMC4726014

[B21] Gotz S. Garcia-Gomez J. M. Terol J. Williams T. D. Nagaraj S. H. Nueda M. J. (2008). High-throughput functional annotation and data mining with the Blast2GO suite. *Nucleic Acids Res.* 36 3420–3435. 10.1093/nar/gkn176 18445632 PMC2425479

[B22] Grabherr M. G. Haas B. J. Yassour M. Levin J. Z. Thompson D. A. Amit I. (2011). Full-length transcriptome assembly from RNA-Seq data without a reference genome. *Nat. Biotechnol.* 29 644–652. 10.1038/nbt.1883 21572440 PMC3571712

[B23] Grosjean Y. Rytz R. Farine J. P. Abuin L. Cortot J. Jefferis G. S. (2011). An olfactory receptor for food-derived odours promotes male courtship in Drosophila. *Nature* 478 236–240. 10.1038/nature10428 21964331

[B24] Grosse-Wilde E. Kuebler L. S. Bucks S. Vogel H. Wicher D. Hansson B. S. (2011). Antennal transcriptome of *Manduca sexta*. *Proc. Natl. Acad. Sci. U.S.A.* 108 7449–7454. 10.1073/pnas.1017963108 21498690 PMC3088587

[B25] Hallem E. A. Dahanukar A. Carlson J. R. (2006). Insect odor and taste receptors. *Annu. Rev. Entomol.* 51 113–135. 10.1146/annurev.ento.51.051705.113646 16332206

[B26] Hussain A. Zhang M. Ucpunar H. K. Svensson T. Quillery E. Gompel N. (2016). Ionotropic chemosensory receptors mediate the taste and smell of polyamines. *PLoS Biol.* 14:e1002454. 10.1371/journal.pbio.1002454 27145030 PMC4856413

[B27] Jiang X. Krieger J. Breer H. Pregitzer P. (2017). Distinct subfamilies of odorant binding proteins in locust (Orthoptera, Acrididae): molecular evolution, structural variation, and sensilla-specific expression. *Front. Physiol.* 8:734. 10.3389/fphys.2017.00734 29018357 PMC5623057

[B28] Jiang X. Pregitzer P. Grosse-Wilde E. Breer H. Krieger J. (2016). Identification and characterization of two “Sensory Neuron Membrane Proteins” (SNMPs) of the desert locust, *Schistocerca gregaria* (Orthoptera: Acrididae). *J. Insect Sci.* 16:33. 10.1093/jisesa/iew015 27012870 PMC4806715

[B29] Jin X. Ha T. S. Smith D. P. (2008). SNMP is a signaling component required for pheromone sensitivity in Drosophila. *Proc. Natl. Acad. Sci. U.S.A.* 105 10996–11001. 10.1073/pnas.0803309105 18653762 PMC2504837

[B30] Kain P. Boyle S. M. Tharadra S. K. Guda T. Christine P. Dahanukar A. (2013). Odour receptors and neurons for DEET and new insect repellents. *Nature* 502 507–512. 10.1038/nature12594 24089210 PMC3927149

[B31] Katoh K. Standley D. M. (2013). MAFFT multiple sequence alignment software version 7: improvements in performance and usability. *Mol. Biol. Evol.* 30 772–780. 10.1093/molbev/mst010 23329690 PMC3603318

[B32] Kearse M. Moir R. Wilson A. Stones-Havas S. Cheung M. Sturrock S. (2012). Geneious basic: an integrated and extendable desktop software platform for the organization and analysis of sequence data. *Bioinformatics* 28 1647–1649. 10.1093/bioinformatics/bts199 22543367 PMC3371832

[B33] Knecht Z. A. Silbering A. F. Cruz J. Yang L. Croset V. Benton R. (2017). Ionotropic receptor-dependent moist and dry cells control hygrosensation in Drosophila. *eLife* 6:e26654. 10.7554/eLife.26654 28621663 PMC5495567

[B34] Knecht Z. A. Silbering A. F. Ni L. Klein M. Budelli G. Bell R. (2016). Distinct combinations of variant ionotropic glutamate receptors mediate thermosensation and hygrosensation in Drosophila. *eLife* 5:e17879. 10.7554/eLife.17879 27656904 PMC5052030

[B35] Koh T. W. He Z. Gorur-Shandilya S. Menuz K. Larter N. K. Stewart S. (2014). The Drosophila IR20a clade of ionotropic receptors are candidate taste and pheromone receptors. *Neuron* 83 850–865. 10.1016/j.neuron.2014.07.012 25123314 PMC4141888

[B36] Krieger J. Grosse-Wilde E. Gohl T. Dewer Y. M. Raming K. Breer H. (2004). Genes encoding candidate pheromone receptors in a moth (*Heliothis virescens*). *Proc. Natl. Acad. Sci. U.S.A.* 101 11845–11850. 10.1073/pnas.0403052101 15289611 PMC511062

[B37] Leal W. S. (2013). Odorant reception in insects: roles of receptors, binding proteins, and degrading enzymes. *Annu. Rev. Entomol.* 58 373–391. 10.1146/annurev-ento-120811-153635 23020622

[B38] Li B. Dewey C. N. (2011). RSEM: accurate transcript quantification from RNA-Seq data with or without a reference genome. *BMC Bioinformatics* 12:323. 10.1186/1471-2105-12-323 21816040 PMC3163565

[B39] Li Z. Ni J. D. Huang J. Montell C. (2014). Requirement for Drosophila SNMP1 for rapid activation and termination of pheromone-induced activity. *PLoS Genet.* 10:e1004600. 10.1371/journal.pgen.1004600 25255106 PMC4177743

[B40] Liu R. He X. Lehane S. Lehane M. Hertz-Fowler C. Berriman M. (2012). Expression of chemosensory proteins in the tsetse fly *Glossina morsitans morsitans* is related to female host-seeking behaviour. *Insect Mol. Biol.* 21 41–48. 10.1111/j.1365-2583.2011.01114.x 22074189 PMC3664020

[B41] Livak K. J. Schmittgen T. D. (2001). Analysis of relative gene expression data using real-time quantitative PCR and the 2-DELTADELTACT method. *Methods* 25 402–408. 10.1006/meth.2001.1262 11846609

[B42] Lu F. P. Zhao D. X. Wang A. P. (2008). Electroantennogram and behavioral responses of *Oxya chinensis* (Orthoptera: Acrididae) to plant volatiles. *Chin. J. Trop. Crops* 29 225–230.

[B43] Martin-Blazquez R. Chen B. Kang L. Bakkali M. (2017). Evolution, expression and association of the chemosensory protein genes with the outbreak phase of the two main pest locusts. *Sci. Rep.* 7:6653. 10.1038/s41598-017-07068-0 28751682 PMC5532218

[B44] Min S. Ai M. Shin S. A. Suh G. S. (2013). Dedicated olfactory neurons mediating attraction behavior to ammonia and amines in Drosophila. *Proc. Natl. Acad. Sci. U.S.A.* 110 E1321–E1329. 10.1073/pnas.1215680110 23509267 PMC3619346

[B45] Ni L. Klein M. Svec K. V. Budelli G. Chang E. C. Ferrer A. J. (2016). The ionotropic receptors IR21a and IR25a mediate cool sensing in Drosophila. *eLife* 5:e13254. 10.7554/eLife.13254 27126188 PMC4851551

[B46] Nichols Z. Vogt R. G. (2008). The SNMP/CD36 gene family in Diptera, Hymenoptera and Coleoptera: *Drosophila melanogaster*, *D-pseudoobscura*, *Anopheles gambiae*, *Aedes aegypti*, *Apis mellifera*, and *Tribolium castaneum*. *Insect Biochem. Mol. Biol.* 38 398–415. 10.1016/j.ibmb.2007.11.003 18342246

[B47] Paula D. P. Togawa R. C. Costa M. M. Grynberg P. Martins N. F. Andow D. A. (2016). Identification and expression profile of odorant-binding proteins in *Halyomorpha halys* (Hemiptera: Pentatomidae). *Insect Mol. Biol.* 25 580–594. 10.1111/imb.12243 27170546

[B48] Pelletier J. Hughes D. T. Luetje C. W. Leal W. S. (2010). An odorant receptor from the southern house mosquito *Culex pipiens quinquefasciatus* sensitive to oviposition attractants. *PLoS One* 5:e10090. 10.1371/journal.pone.0010090 20386699 PMC2851645

[B49] Pregitzer P. Jiang X. Grosse-Wilde E. Breer H. Krieger J. Fleischer J. (2017). In search for pheromone receptors: certain members of the odorant receptor family in the desert locust *Schistocerca gregaria* (Orthoptera: Acrididae) Are Co-expressed with SNMP1. *Int. J. Biol. Sci.* 13 911–922. 10.7150/ijbs.18402 28808423 PMC5555108

[B50] Price M. N. Dehal P. S. Arkin A. P. (2009). FastTree: computing large minimum evolution trees with profiles instead of a distance matrix. *Mol. Biol. Evol.* 26 1641–1650. 10.1093/molbev/msp077 19377059 PMC2693737

[B51] Price M. N. Dehal P. S. Arkin A. P. (2010). FastTree 2–approximately maximum-likelihood trees for large alignments. *PLoS One* 5:e9490. 10.1371/journal.pone.0009490 20224823 PMC2835736

[B52] Prieto-Godino L. L. Rytz R. Bargeton B. Abuin L. Arguello J. R. Peraro M. D. (2016). Olfactory receptor pseudo-pseudogenes. *Nature* 539 93–97. 10.1038/nature19824 27776356 PMC5164928

[B53] Quevillon E. Silventoinen V. Pillai S. Harte N. Mulder N. Apweiler R. (2005). InterProScan: protein domains identifier. *Nucleic Acids Res.* 33 W116–W120. 15980438 10.1093/nar/gki442 PMC1160203

[B54] Robinson M. D. McCarthy D. J. Smyth G. K. (2010). edgeR: a Bioconductor package for differential expression analysis of digital gene expression data. *Bioinformatics* 26 139–140. 10.1093/bioinformatics/btp616 19910308 PMC2796818

[B55] Robinson M. D. Oshlack A. (2010). A scaling normalization method for differential expression analysis of RNA-seq data. *Genome Biol.* 11:R25. 10.1186/gb-2010-11-3-r25 20196867 PMC2864565

[B56] Rogers M. E. Krieger J. Vogt R. G. (2001). Antennal SNMPs (Sensory Neuron Membrane Proteins) of lepidoptera define a unique family of invertebrate CD36-like proteins. *J. Neurobiol.* 49 47–61. 10.1002/neu.1065 11536197

[B57] Rogers M. E. Sun M. Lerner M. R. Vogt R. G. (1997). Snmp-1, a novel membrane protein of olfactory neurons of the silk moth *Antheraea polyphemus* with homology to the CD36 family of membrane proteins. *J. Biol. Chem.* 272 14792–14799. 10.1074/jbc.272.23.14792 9169446

[B58] Sandler B. H. Nikonova L. Leal W. S. Clardy J. (2000). Sexual attraction in the silkworm moth: structure of the pheromone-binding-protein-bombykol complex. *Chem. Biol.* 7 143–151. 10.1016/S1074-5521(00)00078-8 10662696

[B59] Silbering A. F. Rytz R. Grosjean Y. Abuin L. Ramdya P. Jefferis G. S. (2011). Complementary function and integrated wiring of the evolutionarily distinct drosophila olfactory subsystems. *J. Neurosci.* 31 13357–13375. 10.1523/JNEUROSCI.2360-11.2011 21940430 PMC6623294

[B60] Stewart S. Koh T. W. Ghosh A. C. Carlson J. R. (2015). Candidate ionotropic taste receptors in the Drosophila larva. *Proc. Natl. Acad. Sci. U.S.A.* 112 4195–4201. 10.1073/pnas.1503292112 25825777 PMC4394268

[B61] Su C. Carlson J. R. (2013). Neuroscience. Circuit logic of avoidance and attraction. *Science* 340 1295–1297. 10.1126/science.1240139 23766319

[B62] Tauber J. M. Brown E. B. Li Y. Yurgel M. E. Masek P. Keene A. C. (2017). A subset of sweet-sensing neurons identified by IR56d are necessary and sufficient for fatty acid taste. *PLoS Genet.* 13:e1007059. 10.1371/journal.pgen.1007059 29121639 PMC5697886

[B63] Trapnell C. Williams B. A. Pertea G. Mortazavi A. Kwan G. van Baren M. J. (2010). Transcript assembly and quantification by RNA-Seq reveals unannotated transcripts and isoform switching during cell differentiation. *Nat. Biotechnol.* 28 511–515. 10.1038/nbt.1621 20436464 PMC3146043

[B64] Venthur H. Zhou J. J. (2018). Odorant receptors and odorant-binding proteins as insect pest control targets: a comparative analysis. *Front. Physiol.* 9:1163 10.3389/fphys.2018.01163 PMC6117247 30197600

[B65] Vieira F. G. Rozas J. (2011). Comparative genomics of the odorant-binding and chemosensory protein gene families across the arthropoda: origin and evolutionary history of the chemosensory system. *Genome Biol. Evol.* 3 476–490. 10.1093/gbe/evr033 21527792 PMC3134979

[B66] Vogt R. G. Miller N. E. Litvack R. Fandino R. A. Sparks J. Staples J. (2009). The insect SNMP gene family. *Insect Biochem. Mol. Biol.* 39 448–456. 10.1016/j.ibmb.2009.03.007 19364529

[B67] Vogt R. G. Riddiford L. M. Prestwich G. D. (1985). Kinetic properties of a sex pheromone-degrading enzyme: the sensillar esterase of *Antheraea polyphemus*. *Proc. Natl. Acad. Sci. U.S.A.* 82 8827–8831. 10.1073/pnas.82.24.8827 3001718 PMC391531

[B68] Vosshall L. B. Amrein H. Morozov P. S. Rzhetsky A. Axel R. (1999). A spatial map of olfactory receptor expression in the Drosophila antenna. *Cell* 96 725–736. 10.1016/s0092-8674(00)80582-6 10089887

[B69] Wang Z. Yang P. Chen D. Jiang F. Li Y. Wang X. (2015). Identification and functional analysis of olfactory receptor family reveal unusual characteristics of the olfactory system in the migratory locust. *Cell Mol. Life Sci.* 72 4429–4443. 10.1007/s00018-015-2009-9 26265180 PMC4611004

[B70] Wanner K. W. Nichols A. S. Allen J. E. Bunger P. L. Garczynski S. F. Jr. Linn C. E. (2010). Sex pheromone receptor specificity in the european corn borer moth, *Ostrinia nubilalis*. *PLoS One* 5:e8685. 10.1371/journal.pone.0008685 20084285 PMC2801615

[B71] Xu P. Atkinson R. Jones D. N. Smith D. P. (2005). *Drosophila* OBP LUSH is required for activity of pheromone-sensitive neurons. *Neuron* 45 193–200. 10.1016/j.neuron.2004.12.031 15664171

[B72] Xu Y. L. He P. Zhang L. Fang S. Q. Dong S. L. Zhang Y. J. (2009). Large-scale identification of odorant-binding proteins and chemosensory proteins from expressed sequence tags in insects. *BMC Genomics* 10:632. 10.1186/1471-2164-10-632 20034407 PMC2808328

[B73] Yan S. W. Zhang J. Liu Y. Li G. Q. Wang G. R. (2015). An olfactory receptor from *Apolygus lucorum* (Meyer-Dur) mainly tuned to volatiles from flowering host plants. *J. Insect Physiol.* 79 36–41. 10.1016/j.jinsphys.2015.06.002 26050917

[B74] Yi X. Zhao H. Dong X. Wang P. Hu M. Zhong G. (2013). BdorCSP2 is important for antifeed and oviposition-deterring activities induced by Rhodojaponin-III against *Bactrocera dorsalis*. *PLoS One* 8:e77295. 10.1371/journal.pone.0077295 24155937 PMC3796470

[B75] Yu F. Zhang S. Zhang L. Pelosi P. (2009). Intriguing similarities between two novel odorant-binding proteins of locusts. *Biochem. Biophys. Res. Commun.* 385 369–374. 10.1016/j.bbrc.2009.05.074 19464264

[B76] Zhang D. D. Löfstedt C. (2013). Functional evolution of a multigene family: orthologous and paralogous pheromone receptor genes in the turnip moth, *Agrotis segetum*. *PLoS One* 8:e77345. 10.1371/journal.pone.0077345 24130875 PMC3795068

[B77] Zhang S. Pang B. Zhang L. (2015). Novel odorant-binding proteins and their expression patterns in grasshopper, *Oedaleus asiaticus*. *Biochem. Biophys. Res. Commun.* 460 274–280. 10.1016/j.bbrc.2015.03.024 25778868

[B78] Zhang T. Wang W. Zhang Z. Zhang Y. Guo Y. (2013). Functional characteristics of a novel chemosensory protein in the cotton bollworm *Helicoverpa armigera* (Hubner). *J. Integr. Agric.* 12 853–861. 10.1016/s2095-3119(13)60304-4

[B79] Zhang Y. Tan Y. Zhou X. R. Pang B. P. (2018). A whole-body transcriptome analysis and expression profiling of odorant binding protein genes in *Oedaleus infernalis*. *Comp. Biochem. Physiol. Part D Genomics Proteomics* 28 134–141. 10.1016/j.cbd.2018.08.003 30195212

[B80] Zhang Y. N. Ye Z. F. Yang K. Dong S. L. (2014). Antenna-predominant and male-biased CSP19 of *Sesamia inferens* is able to bind the female sex pheromones and host plant volatiles. *Gene* 536 279–286. 10.1016/j.gene.2013.12.011 24361960

[B81] Zhang Y. V. Ni J. Montell C. (2013). The molecular basis for attractive salt-taste coding in Drosophila. *Science* 340 1334–1338. 10.1126/science.1234133 23766326 PMC4091975

[B82] Zhou Y. T. Li L. Zhou X. R. Tan Y. Pang B. P. (2019). Identification and expression profiling of candidate chemosensory membrane proteins in the band-winged grasshopper, *Oedaleus asiaticus*. *Comp. Biochem. Physiol. Part D Genomics Proteomics* 30 33–44. 10.1016/j.cbd.2019.02.002 30771563

