# Supplementary figures and images for: Identification and Expression Analyses of Olfactory Gene Families in the Rice Grasshopper, Oxya chinensis, From Antennal Transcriptomes
Source: Front Physiol. 2019 Sep 26;10:1223. doi: 10.3389/fphys.2019.01223 (PMC6775195; doi:10.3389/fphys.2019.01223)

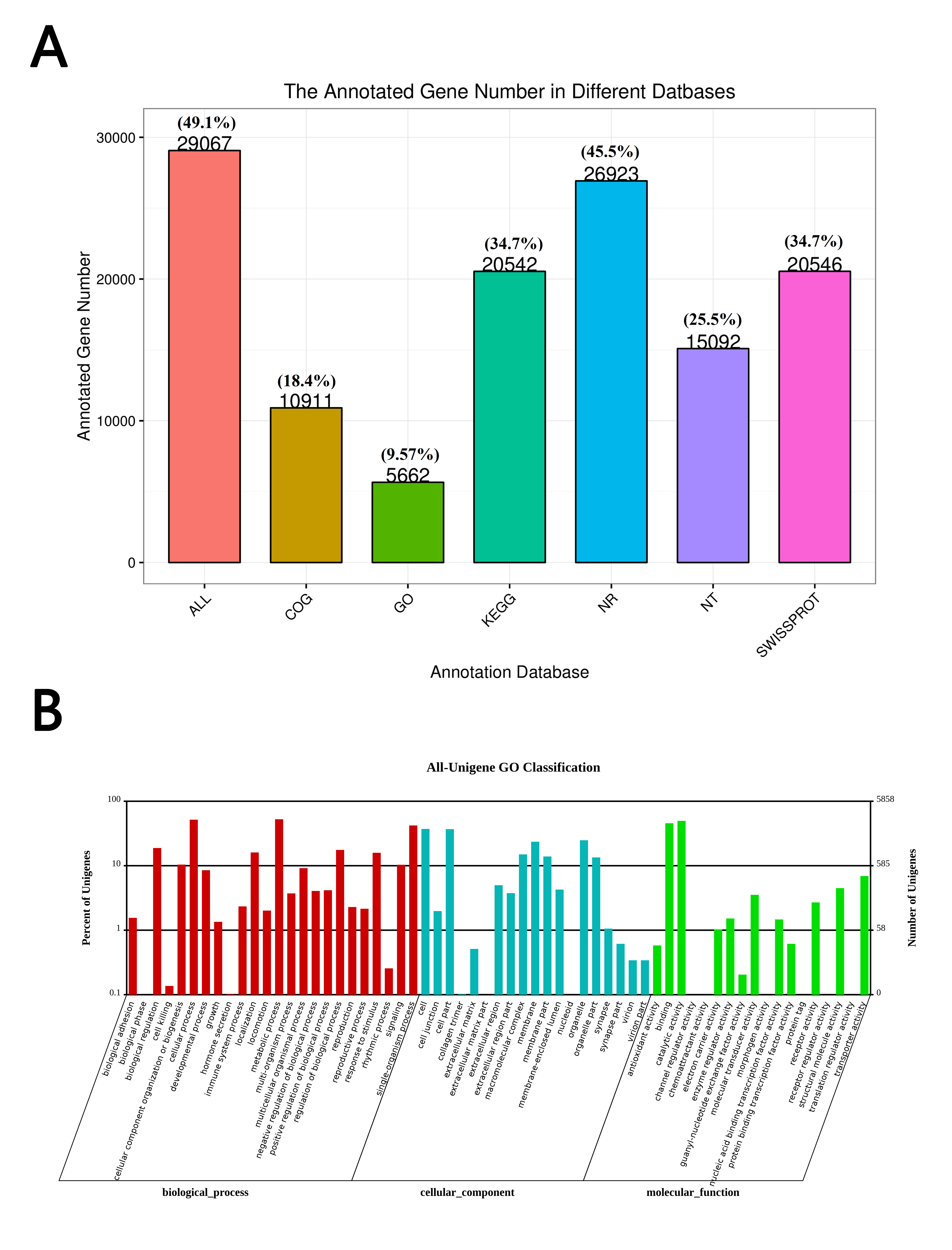

Supplement: FIGURE S1 — Transcriptome overview of O. chinensis antennae. (A) Unigenes annotated through different databases. (B) Gene ontology (GO) classification of the O. chinensis unigenes. [file Image_1.TIF]

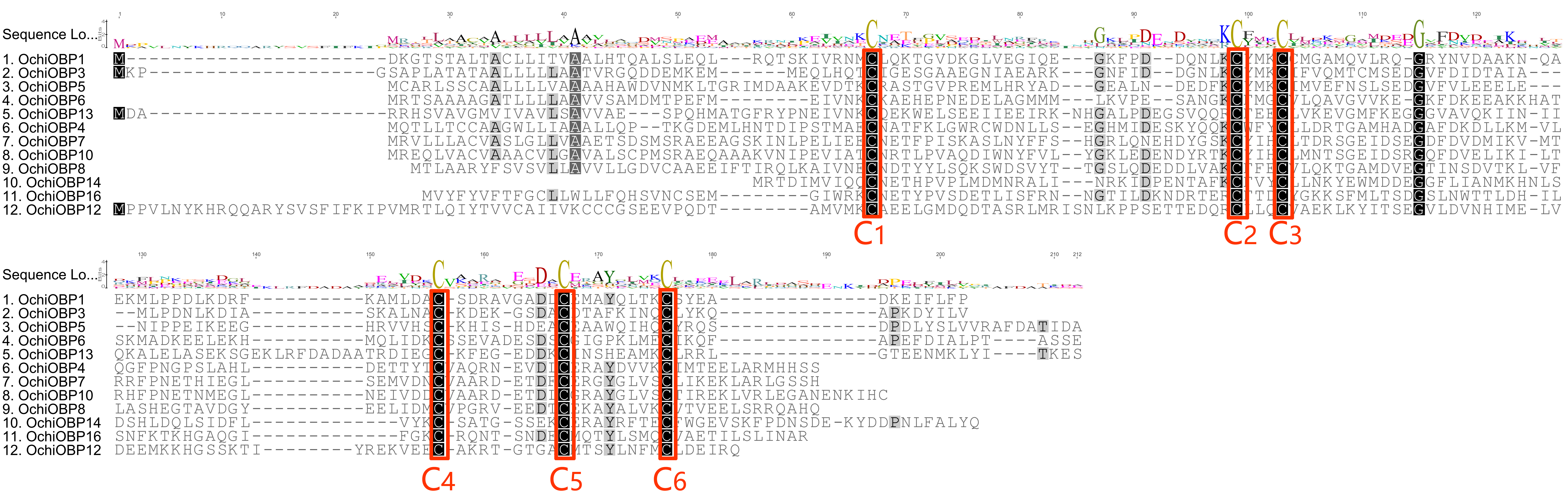

Supplement: FIGURE S2 — The amino acid alignment of the predicted classic OBPs. [file Image_2.TIF]

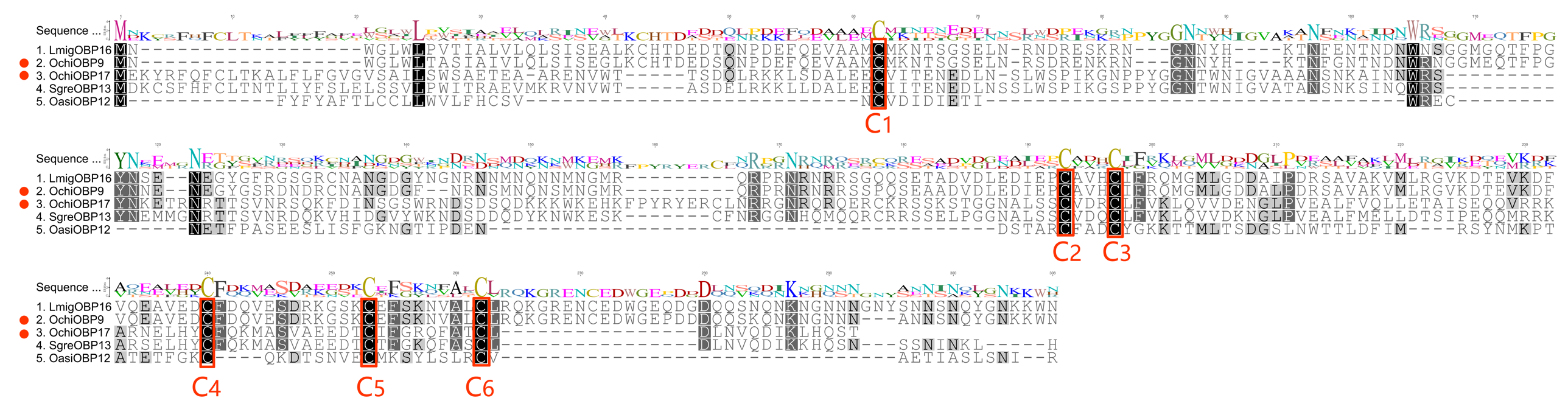

Supplement: FIGURE S3 — The amino acid alignment of the predicted atypical OBPs. [file Image_3.TIF]

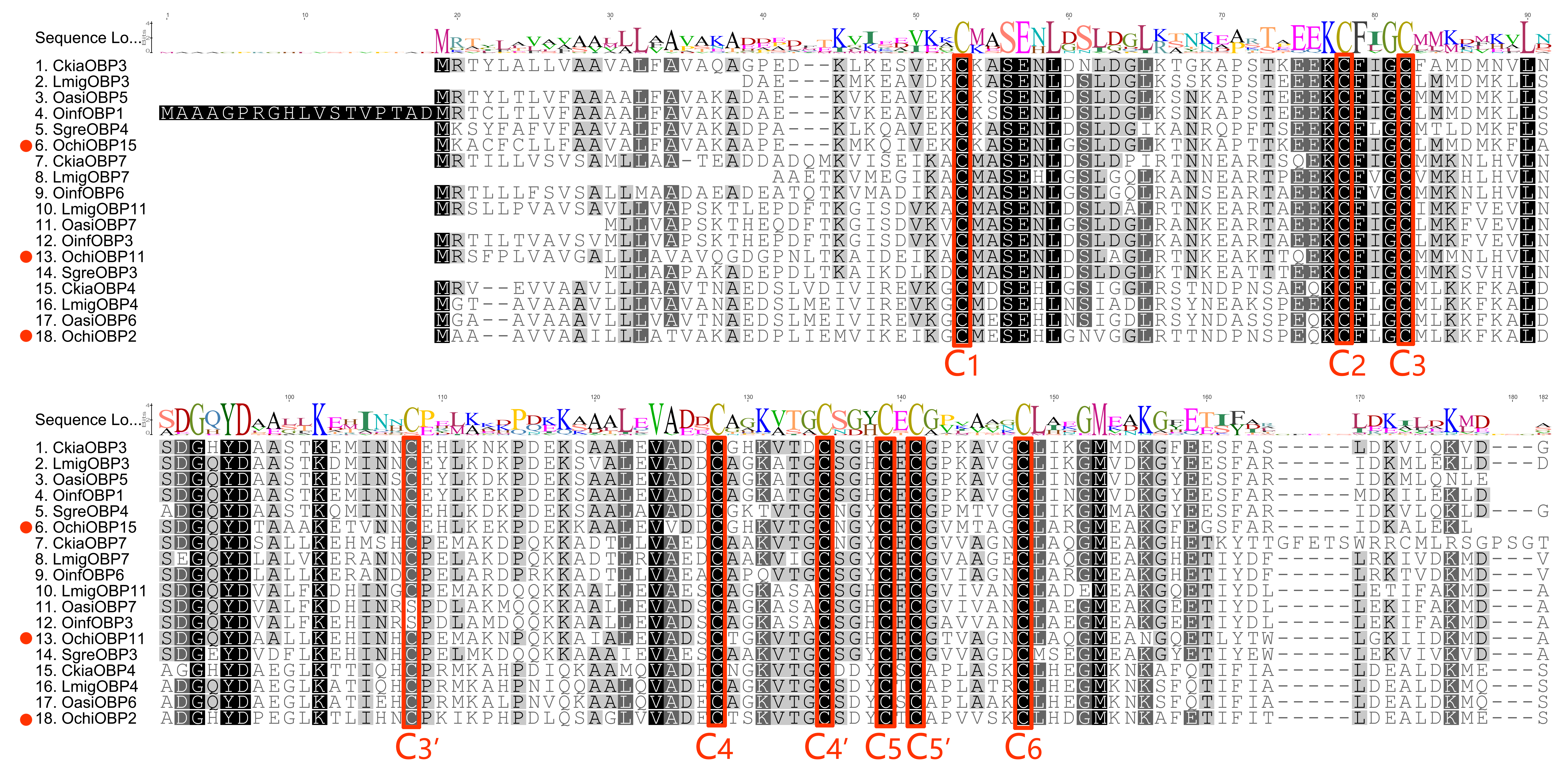

Supplement: FIGURE S4 — The amino acid alignment of the predicted plus-C OBPs type-A. [file Image_4.TIF]

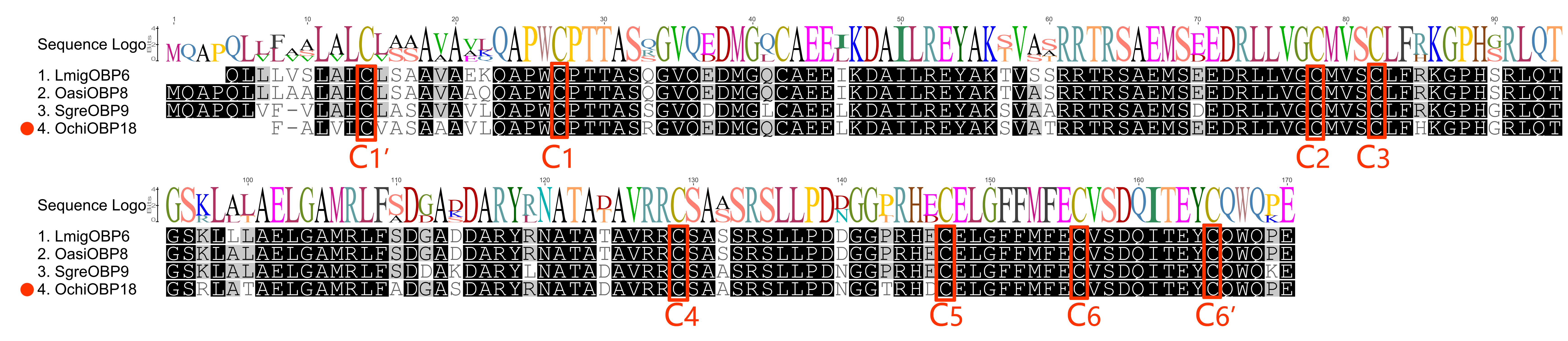

Supplement: FIGURE S5 — The amino alignment of the predicted plus-C OBPs type-B. [file Image_5.TIF]

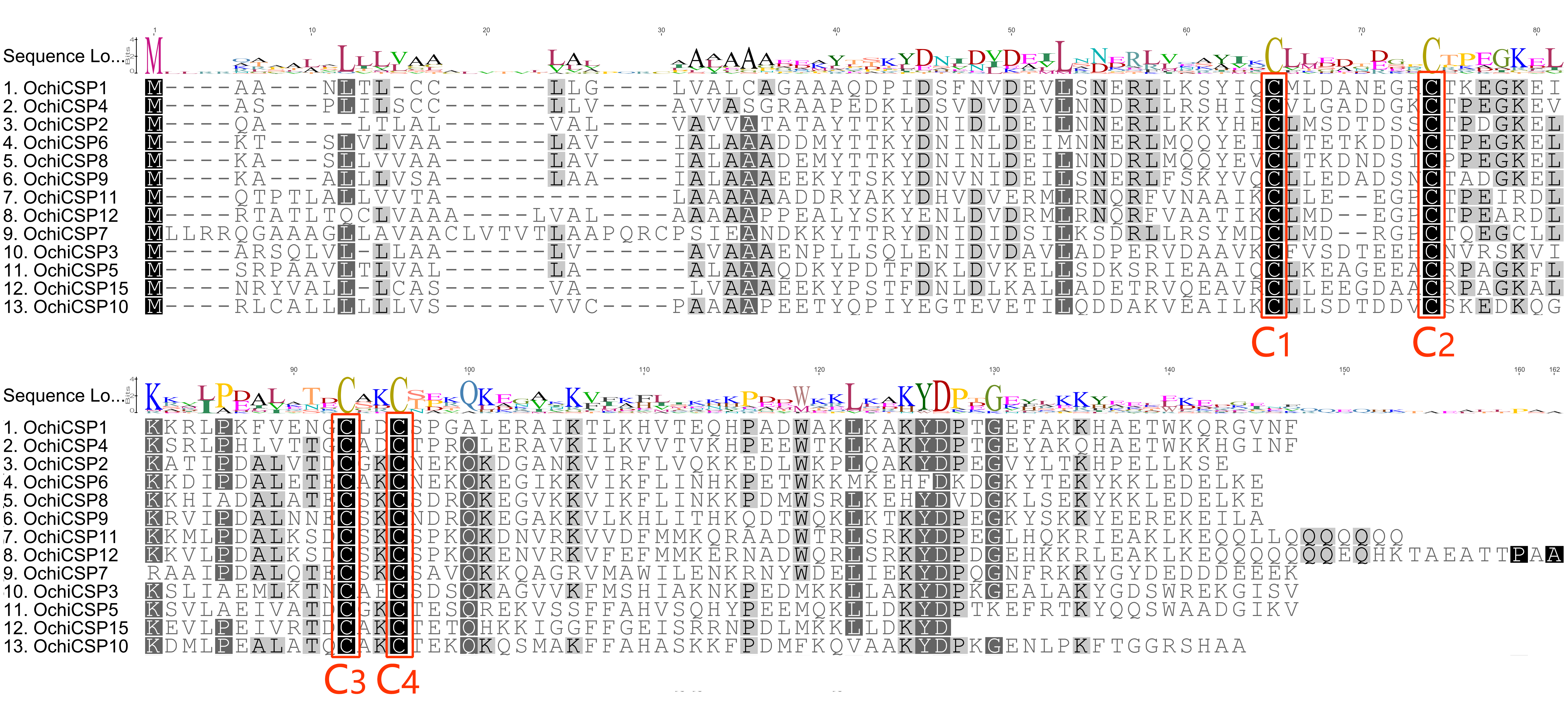

Supplement: FIGURE S6 — The amino alignment of the predicted CSPs. [file Image_6.TIF]
