# Supplementary material for: Identification and Expression Analyses of Olfactory Gene Families in the Rice Grasshopper, Oxya chinensis, From Antennal Transcriptomes
Source: Front Physiol. 2019 Sep 26;10:1223. doi: 10.3389/fphys.2019.01223 (PMC6775195; doi:10.3389/fphys.2019.01223)
Supplement: TABLE S3 — The amino acid sequences used for phylogenetic analyses. [file Table_3.DOC]

**Table S3. Amino acid sequences of *O. chinensis* and other insect used in phylogenetic analyses.**

| **Gene family** | **Species** | **Number** | **References** |
| --- | --- | --- | --- |
| OBP | *Oxya chinensis* | 18 | This study |
|  | *Oedaleus infernalis* | 18 | Zhang et al., 2018 |
|  | *Locusta migratoria* | 16 | Ban et al., 2003; Xu et al., 2009; Yu et al., 2009 |
|  | *Oedaleus asiaticus* | 15 | Zhang et al., 2015 |
|  | *Schistocerca gregaria* | 14 | Jiang et al., 2017 |
|  | *Ceracris kiangsu* | 7 | Jiang et al., 2017 |
| CSP | *Oxya chinensis* | 13 | This study |
|  | Other locust species | 32 | NCBI |
| OR | *Oxya chinensis* | 94 | This study |
|  | *Schistocerca gregaria* | 120 | Yang et al., 2012; Pregitzer et al., 2017 |
|  | *Locusta migratoria* | 141 | Wang et al., 2015 |
| IR | *Oxya chinensis* | 12 | This study |
|  | *Locusta migratoria* | 47 | Wang et al., 2015 |
|  | *Drosophila melanogaster* | 73 | Croset et al., 2010 |
| SNMP | *Oxya chinensis* | 2 | This study |
|  | *Schistocerca gregaria* | 2 | Jiang et al., 2016 |
|  | *Oedaleus asiaticus* | 3 | Zhou et al., 2019 |
|  | Other insect species | 29 | Nichols et al., 2008 |

All amino acid sequences used in phylogenetic analyses are listed below.

**References**

1. Andersson, M.N., Grosse-Wilde, E., Keeling, C.I., Bengtsson, J.M., Yuen, M.M.S., Li, M., Hillbur, Y., Bohlmann, J., Hansson, B.S., Schlyter, F., 2013. Antennal transcriptome analysis of the chemosensory gene families in the tree killing bark beetles, Ips typographus and Dendroctonus ponderosae (Coleoptera: Curculionidae: Scolytinae). BMC Genomics 14, 198.
2. Ban, L., Scaloni, A., D'Ambrosio, C., Zhang, L., Yahn, Y., Pelosi, P., 2003. Biochemical characterization and bacterial expression of an odorant-binding protein from Locusta migratoria. Cell Mol Life Sci 60, 390-400.
3. Croset, V., Rytz, R., Cummins, S.F., Budd, A., Brawand, D., Kaessmann, H., Gibson, T.J., Benton, R., 2010. Ancient protostome origin of chemosensory ionotropic glutamate receptors and the evolution of insect taste and olfaction. PLoS Genet 6, e1001064.
4. Jiang, X., Krieger, J., Breer, H., Pregitzer, P., 2017. Distinct Subfamilies of Odorant Binding Proteins in Locust (Orthoptera, Acrididae): Molecular Evolution, Structural Variation, and Sensilla-Specific Expression. Front Physiol 8, 734.
5. Jiang, X., Pregitzer, P., Grosse-Wilde, E., Breer, H., Krieger, J., 2016. Identification and Characterization of Two "Sensory Neuron Membrane Proteins" (SNMPs) of the Desert Locust, Schistocerca gregaria (Orthoptera: Acrididae). J Insect Sci 16.
6. Nichols, Z., Vogt, R.G., 2008. The SNMP/CD36 gene family in Diptera, Hymenoptera and Coleoptera: Drosophila melanogaster, D-pseudoobscura, Anopheles gambiae, Aedes aegypti, Apis mellifera, and Tribolium castaneum. Insect Biochemistry and Molecular Biology 38, 398-415.
7. Pregitzer, P., Jiang, X., Grosse-Wilde, E., Breer, H., Krieger, J., Fleischer, J., 2017. In Search for Pheromone Receptors: Certain Members of the Odorant Receptor Family in the Desert Locust Schistocerca gregaria (Orthoptera: Acrididae) Are Co-expressed with SNMP1. Int J Biol Sci 13, 911-922.
8. Vogt, R.G., Miller, N.E., Litvack, R., Fandino, R.A., Sparks, J., Staples, J., Friedman, R., Dickens, J.C., 2009. The insect SNMP gene family. Insect Biochemistry and Molecular Biology 39, 448-456.
9. Wang, Z., Yang, P., Chen, D., Jiang, F., Li, Y., Wang, X., Kang, L., 2015. Identification and functional analysis of olfactory receptor family reveal unusual characteristics of the olfactory system in the migratory locust. Cell Mol Life Sci 72, 4429-4443.
10. Xu, Y.L., He, P., Zhang, L., Fang, S.Q., Dong, S.L., Zhang, Y.J., Li, F., 2009. Large-scale identification of odorant-binding proteins and chemosensory proteins from expressed sequence tags in insects. BMC Genomics 10, 632.
11. Yang, Y., Krieger, J., Zhang, L., Breer, H., 2012. The olfactory co-receptor Orco from the migratory locust (Locusta migratoria) and the desert locust (Schistocerca gregaria): identification and expression pattern. Int J Biol Sci 8, 159-170.
12. Yu, F., Zhang, S., Zhang, L., Pelosi, P., 2009. Intriguing similarities between two novel odorant-binding proteins of locusts. Biochem Biophys Res Commun 385, 369-374.
13. Zhang, S., Pang, B., Zhang, L., 2015. Novel odorant-binding proteins and their expression patterns in grasshopper, Oedaleus asiaticus. Biochem Biophys Res Commun 460, 274-280.
14. Zhang, Y., Tan, Y., Zhou, X.R., Pang, B.P., 2018. A whole-body transcriptome analysis and expression profiling of odorant binding protein genes in Oedaleus infernalis. Comp Biochem Physiol Part D Genomics Proteomics 28, 134-141.
15. Zhou, Y.T., Li, L., Zhou, X.R., Tan, Y., Pang, B.P., 2019. Identification and expression profiling of candidate chemosensory membrane proteins in the band-winged grasshopper, Oedaleus asiaticus. Comp Biochem Physiol Part D Genomics Proteomics 30, 33-44.

OBP

>OchiOBP1

MDKGTSTALTACLLITVAALHTQALSLEQLRQTSKIVRNMCLQKTGVDKGLVEGIQEGKFPDDQNLKCYMKCCMGAMQVLRQGRYNVDAAKNQAEKMLPPDLKDRFKAMLDACSDRAVGADDCEMAYQLTKCSYEADKEIFLFP

>OchiOBP2

MAAAVVAAILLLATVAKAEDPLIEMVIKEIKGCMESEHLGNVGGLRTTNDPNSPEQKCFLGCMLKKFKALDADGHYDPEGLKTLIHNCPKIKPHPDLQSAGLVVADECTSKVTGCSDYCTCAPVVSKCLHDGMKNKAFETIFITLDEALDKMES

>OchiOBP3

MKPGSAPLATATAALLLLLAATVRGQDDEMKEMMEQLHQTCIGESGAAEGNIAEARKGNFIDDGNLKCYMKCIFVQMTCMSEDGVFDIDTAIAMLPDNLKDIASKALNACKDEKGSDACDTAFKINQCLYKQAPKDYILV

>OchiOBP4

MQTLLTCCAAGWLLIAAALLQPTKGDEMLHNTDIPSTMAECNATFKLGWRCWDNLLSEGHMIDESKYQQKCWFYCLLDRTGAMHADGAFDKDLLKMVLQGFPNGPSLAHLDETTYTCVAQRNEVDLCERAYDVVKCIMTEELARMHHSS

>OchiOBP5

MCARLSSCAALLLLVAAAAHAWDVNMKLTGRIMDAAKEVDTKCRASTGVPREMLHRYADGEALNDEDFKCYMKCIMVEFNSLSEDGVFVLEEELENIPPEIKEEGHRVVHSCKHISHDEACEAAWQIHQCYRQSDPDLYSLVVRAFDATIDA

>OchiOBP6

MRTSAAAAGATLLLLAAVVSAMDMTPEFMEIVNKCKAEHEPNEDELAGMMMLKVPESANGKCFMGCVLQAVGVVKEGKFDKEEAKKHATSKMADKEELEKHMQLIDKCSSEVADESDSCGIGPKLMECIKQFAPEFDIALPTASSE

>OchiOBP7

MRVLLLACVASLGLLVAAETSDSMSRAEEAGSKINLPELIEECNETFPISKASLNYFFSHGRLQNEHDYGSKCYIHCLTDRSGEIDSEGDFDVDMIKVMTRRFPNETHIEGLSEMVDNCVAARDETDFCERGYGLVSCLIKEKLARLGSSH

>OchiOBP8

MTLAARYFSVSVLLAVVLLGDVCAAEEIFTIRQLKAIVNECNDTYYLSQKSWDSVYTTGSLQDEDDLVAKCFFECVLQKTGAMDVEGTINSDVTKLVFLASHEGTAVDGYEELIDMCVPGRVEEDTCEKAYALVKCVTVEELSRRQAHQ

>OchiOBP9

MNWGLWLTASIAIVLQLSISEGLKCHTDEDSQNPDEFQEVAAMCMKNTSGSELNRSDRENKRNGNNYHKTNFGNTNDNWRNGGMEQTFPGYNNENEGYGSRDNDRCNANGDGFNRNSMNQNSMNGMRQRPRNRNRRSSPQSEAADVDLEDIEPCAVHCIFRQMGMLGDDALPDRSAVAKVMLRGVKDTEVKDFVQEAVEDCFDQVESDRKGSKCEFSKNVALCLRQKGRENCEDWGEPDDDQQSKQNKNGNNNANNSNQYGNKKWN

>OchiOBP10

MREQLVACVAAACVLGAVALSCPMSRAEQAAAKVNIPEVIATCNRTLPVAQDIWNYFVLYGKLEDENDYRTKCYIHCLMNTSGEIDSRGQFDVELIKILTRHFPNETNMEGLNEIVDDCVAARDETDLCGRAYGLVSCTIREKLVRLEGANENKIHC

>OchiOBP11

MRSFPLVAVGALLLAVAVQGDGPNLTKAIDEIKACMASENLDSLAGLRTNKEAKTTQEKCFIGCMMMNLHVLNSDGQYDAALLKEHINHCPEMAKNPQKKAIALEVADSCTGKVTGCSGHCECGTVAGNCLAQGMEANGQETLYTWLGKIIDKMDA

>OchiOBP12

MPPVLNYKHRQQARYSVSFIFKIPVMRTLQIYTVVCAIIVKCCCGSEEVPQDTAMVMKCAEELGMDQDTASRLMRISNLKPPSETTEDQRCLLQCVAEKLKYITSEGVLDVNHIMELVDEEMKKHGSSKTIYREKVEECAKRTGTGACMTSYLNFMCLDEIRQ

>OchiOBP13

MDARRHSVAVGMVIVAVLSAVVAESPQHMATGFRYPNEIVNKCQEKWELSEEIIEEIRKNHGALPDEGSVQQRCFEECLVKEVGMFKEGGGVAVQKIINIIQKALELASEKSGEKLRFDADAATRDIEGCKFEGEDDKCINSHEAMKCLRRLGTEENMKLYITKES

>OchiOBP14

MRTDIMVIQQCNETHPVPLMDMNRALINRKIDPENTAFKCFVYCLLNKYEWMDDEGGFLIANMKHNLSDSHLDQLSIDFLVYKCSATGSSEKCERAYRFTECFWGEVSKFPDNSDEKYDDPNLFALYQ

>OchiOBP15

MKACFCLLFAAVALFAVAKAAPEKMKQIVEKCKASENLGSLDGLKTNKAPTTKEEKCFIGCLMMDMKFLASDGQYDTAAAKETVNNCEHLKEKPDEKKAALEVVDDCGHKVTGCNGYCECGVMTAGCLARGMEAKGFEGSFARIDKALEKL

>OchiOBP16

MVYFYVFTFGCLLWLLFQHSVNCSEMGIWRKCNETYPVSDETLISFRNNGTILDKNDRTERCFTDCYGKKSFMLTSDGSLNWTTLDHILSNFKTKHGAQGIFGKCRQNTSNDECMQTYLSMQCVAETILSLINAR

>OchiOBP17

MEKYRFQFCLTKALFLFGVGVSAILSWSAETEAARENVWTTSDQLRKKLSDALEECVITENEDLNSLWSPIKGNPPYGGNTWNIGVAAANSNKAINNWRSYNKETRNRTTSVNRSQKFDINSGSWRNDSDSQDKKKWKEHKFPYRYERCLNRRGNRQRQERCKRSSKSTGGNALSSCVDRCLFVKLQVVDENGLPVEALFVQLLETAISEQQVRRKARNELHYCFQKMASVAEEDTCIFGRQFATCLDLNVQDIKLHQST

>OchiOBP18

FALVLCVASAAAVLQAPWCPTTASRGVQEDMGQCAEELKDAILREYAKSVATRRTRSAEMSEEDRLLVGCMVSCLFHKGPHGRLQTGSRLATAELGAMRLFADGASDARYRNATADAVRRCSAASRSLLPDNGGTRHDCELGFFMFECVSDQITEYCQWQPE

>LmigOBP1

MWARLNDCAALLLLLAAAARAWDVNMKLTGRIMDAAKEVDHTCRSSTGVPRDMLHRYAEGQTVDDDDFKCYLKCIMVEFNSLSDDGVFVLEEELENVPPEIKEEGHRVVHSCKHINHDEACETAYQIHQCYKQSDPELYSLVVRAFDATIGD

>LmigOBP2

WIPGLRNSNSDMRTSAAAAATGPAFLVLAAVASAMEMTPEFMEIVNKCKTEHEPTEDELKGMMALKVPESANGKCFMGCVLQEIGVVKDGKFDKEEAKKHAASKMTDKDELEKHMQLIEKCSQEVGGETDSRGIGPKLMECIKQFAPEFDIALPQQPSE

>LmigOBP3

DAEKMKEAVDKCKASENLDSLDGLKSSKSPSTEEEKCFIGCLMMDMKLLSSDGQYDAASTKDMINNCEYLKDKPDEKSVALEVADDCAGKATGCSGHCECGPKAVGCLIKGMVDKGYEESFARIDKMLEKLDD

>LmigOBP4

MGTAVAAAVLLLVAVANAEDSLMEIVIREVKGCMDSEHLNSIADLRSYNEAKSPEEKCFLGCMLKKFKALDADGQYDAEGLKATIQHCPRMKAHPNIQQAALQVADECAGKVTGCSDYCTCAPLATRCLHEGMKNKSFQTIFIALDEALDKMQS

>LmigOBP5

MALAARVFSATLMLTAVLFGDISTAGEVFTMSQLKAAVNECNDTYFLSQKNWDTVFTTGSLEDENDLVAKCFFECVLEKTGAMDEKGNINSDITKAVFLASHEGTGTAVQGHDDLIDMCVPGRDETDICERGYALVKCVTVEELSRRQARK

>LmigOBP6

QLLLVSLALCLSAAVAEKQAPWCPTTASQGVQEDMGQCAEEIKDAILREYAKTVSSRRTRSAEMSEEDRLLVGCMVSCLFRKGPHSRLQTGSKLLLAELGAMRLFSDGADDARYRNATATAVRRCSASSRSLLPDDGGPRHECELGFFMFECVSDQITEYCQWQPE

>LmigOBP7

AAETKVMEGIKACMASEHLGSLGQLKANNEARTPEEKCFVGCVMKHLHVLNSEGQYDLALVKERANNCPELAKDPQKKADTLRVAEDCAAKVIGCSGYCECGVAAGECLAQGMEAKGHETIYDFLRKIVDKMDV

>LmigOBP8

AVRLLLLLPPLLLLALSCVTAAPSITSTEMRMDMMVIQHCNETHPVALIDMNKALINKKIEPQNTVFKCFVFCLLNKYEWMDDEGGFLIANMKHNLSDSHLDQLSIDFIVYKCSATGSSDKCERAYRFTECFWGEVTKFPENSDEKYEDPDLFALYQ

>LmigOBP9

MDKASSAAVTACLLIAVAALHTQALSLEQLRQTSKIVRNMCLKKTGVDLALVEGIQEGQFPDNQDLKCYMKCCMGAMQVLRQGRYNVNAAKNQADKMLPPDLKGRFIDMLDACSDRGDGVDDDCEMAYQLTKCSYETDKEIFLFP

>LmigOBP10

AISESMSRAEEAASKIDIPELFEECNETFTIPKVTLNYFFSHGRLQNENDYGSKCFVHCLTDRSGEIDSDGNFDVDLIKVMTRRFPNETNIEGLNEMVETCVADRGETDFCERAYGLVSCLVKEKLARLGNSH

>LmigOBP11

MRSLLPVAVSAVLLVAPSKTLEPDFTKGISDVKACMASENLDSLDALRTNKEARTAEEKCFIGCIMKFVEVLNSDGQYDVALFKDHINGCPEMAKDQQKKAALLEVAESCAGKASACSGHCECGVIVANCLADEMEAKGQETIYDLLETIFAKMDA

>LmigOBP12

AVILTAASTLWFAAAAFAAMVTTEIPTEDILQRVQVCNKTYPVSQEMLRSLASTGGLLSDESDVNTRCYLECYERLGGTVNKDGKFNPEKAVTLLVSYYPKIAELGVDSVTEILKNCNSKSGTGQCMTSYLIRNCFIAGLNAKSPHTSVFDTSSSHI

>LmigOBP13

MSALFTCCVAAWLLLAAALLQPTKGDDVWHNTDIPATMAECNATFRLGWRCWDNLLSDGHVIDESKYQQKCWFYCLLDETGSMHADGAFDKDLLKTVLQGFPNGSSLAHLDETTYTCVAQRNEVDLCERAYAVVKCIMTEELSRMHQSS

>LmigOBP14

MCLVVLLFGFVQSLSLLFVPDSCLLLFVVVMFYFPCKISQANGSFVMTMLCLEQTCKMMDQLHQTCVGESGVSEGNIDAARKGNFIDDGNLKCYMKCIFVQMTCMSDDGVFDADTAIAMLPDNLKDVASKALNACKGEKGSDACDTAFKINQCLFKQAPKDYILV

>LmigOBP15

MFYFYAFTFCCLLWMLFHCSVNCVNVDIETIWRECNETFPASEEALISFGKNGTIPDENDSVARCFTDCYGKKTTLLTSDGSLNWTTLDFLMRSYDMKPTAKETFGKCQKNTSNVECMKSYLSLRCVAETVESLTDIR

>LmigOBP16

MNWGLWLPVTIALVLQLSISEALKCHTDEDTQNPDEFQEVAAMCMKNTSGSELNRNDRESKRNGNNYHKTNFENTNDNWNSGGMGQTFPGYNSENEGYGFRGSGRCNANGDGYNGNRNNMNQNNMNGMRQRPRNRNRRSGQQSETADVDLEDIEPCAVHCIFRQMGMLGDDAIPDRSAVAKVMLRGVKDTEVKDFVQEAVEDCFDQVESDRKGSKCEFSKNVALCLRQKGRENCEDWGEQDGDQQSNQNKNGNNNGNYSNNSNQYGNKKWN

>SgreOBP1

MWARLCNCAALLLLLVAAAQAWDVNMKLTGRIMDAAKEVDHKCRGSTGVPREMLHRYADGETVDDDDFKCYLKCIMIEFNSLSEDGVFVLEEELENIPPEIKEEGHRVVHSCKHINHDEACQTAYQIHQCYKQSDPELYSLVVRAFDATI

>SgreOBP2

MASHCHATVAAVVVAAAFAAVVAESPPHMAAFTFTKEMLSTCQEKSEISQEMVDEMQKNKGLLPDESSVAQRCFQECMAKEMGLLKNGGGVAVDNIVTVLKAALQMASEGSEDTYTIDTDAVTRDLEGCQFEGEDDECNNSHDTMKCLRSLGNPENMKRYITKES

>SgreOBP3

MLLAAPAKADEPDLTKAIKDLKDCMASENLDSLDGLKTNKEATTTEEKCFIGCMMKSVHVLNSDGEYDVDFLKEHINHCPELMKDQQKKAAAIEVAESCAAKVTGCSGYCECGVVAGDCMSEGMEAKGYETIYEWLEKVIVKVDA

>SgreOBP4

MKSYFAFVFAAVALFAVAKADPAKLKQAVEKCKASENLDSLDGIKANRQPFTSEEKCFLGCMTLDMKFLSADGQYDAASTKQMINNCEHLKDKPDEKSAALAVADDCGKTVTGCNGYCECGPMTVGCLIKGMMAKGYEESFARIDKVLQKLDG

>SgreOBP5

MDKGTTAALTASLLIVVAAVHTQALSLEQLRQTSKIVRNMCLKKTSVDLALVEGIQEGKFPDDQNLKCYMKCCMGAMQVLRQGRYNVNAAKNQAEKMLPPDLKDRFIAMLDACSDQAVGEDDCEMAYQLTKCSYEADKEIFLFP

>SgreOBP6

MKAHTASLATATVVILLLVAAVVRGQDDDMKEMMEQLHQSCLGESGASDANIDEARKGNFIEDGNLKCYMKCIFVQMTCMSDDGVFDADTAIAMLPDNLKDVASKALTACKDEKGSDACDTAFKINQCLFKQAPKDYILV

>SgreOBP7

MATTVLSPAALLLALLLAAGSVTARRGPRPFGRCASSVGDIDRDTMMKIVRNFEVPDETSDDQKCMLRCALMTNRLVRDGVLDTRQILMNIRADAHFAGRMASVYGQNITLDMDRLSNDVEACVTSEESPDCDTVYNQFKCVSDLISNGNMASYASFVEVDGSETGDWMRRRQMLASRPHGPPGPMGGHWGPPPPHHRGGPRGGGRPPPPPPESDENDVEELE

>SgreOBP8

MRTSAAATGAALLVFVAVVSAMEMTPEFMEIINKCKAEHEPTEDELKGIMMMKVPESEHGKCFMGCVLQEVGVVKDGKFDKEEAKKHAAAKMSDKDELEKHMQLIDKCSQEVDGETDSCGIGPKLMECIKQFAPEFDIALPHAPSE

>SgreOBP9

MQAPQLVFVLALCLASAVAVLQAPWCPTTASSGVQDDMGLCAEELKDAILREYAKSVAARRTRSAEMSDEDRLLVGCMVSCLFRKGPHGRLQTGSKLALAELGAMRLFSDDAKDARYLNATADAVRRCSAASRSLLPDNGGPRHECELGFFMFECVSDQITEYCQWQKE

>SgreOBP10

MESAMKTLLVVCVAALGFLVAAEISESMSRAEEAAAKINLPELFEECNETFPIPKVTLNYFFSHGRLQNENDYVAKCFIHCLTDRSGEIDSEGDFDVDLIKVMTRRFPNETNIEGLSEMVDKCVAGRGETDFCERAYGLVSCLVKEKLARLGHSH

>SgreOBP11

MNTLFTCCVAVWLLIAAALLQPTKGDEMLPNTDIPATMAECNATFKLGWRCWDNLLSDGHVIDESKYQQKCWFYCLLDRTGAMHADGAFDKDLLKMVLQGFPNGPSLAHLNETTYTCVAQRSEVDLCERAYAIVKCIMTEELSRMHHSS

>SgreOBP12

MKWSLWLTATIALVLQLSISEGLKCHTDEDSQNPDEFQEVAAICMKNTSGSELNRSDRENKRNGNNYHKNNFGNTNDNWSSGGMGQTFPGYNSENEGYGLHGSGRCTANNDGYNNNRNNMNQNNMNGMRQKPRNRNRRSGQQSEAANVDLEDIEPCAVHCIFRQMGMLGDDALPDRSAVAKVMLRGVKDTEVKDFVQEAVEDCFDQVESDRKGSKCDLSKNVALCLRQKGRENCEDWGEQEDDQQSNQNKNGNNNGNNSNNSNNQYGNKKWN

>SgreOBP13

MDKCSFHFCLTNTLIYFSLELSSVLPWITRAEVMKRVNVWTASDELRKKLLDALEECIITENEDLNSSLWSPIKGSPPYGGNTWNIGVATANSNKSINQWRSYNEMMGNRTTSVNRDQKVHIDGVYWKNDSDDQDYKNWKESKCFNRGGNHQMQQRCRRSSELPGGNALSSCVDQCLFVKLQVVDKNGLPVEALFMELLDTSIPEQQMRRKARSELHYCFQKMASVAEEDTCTFGKQFASCLDLNVQDIKKHQSNSSNINKLH

>SgreOBP14

MIFSVRFFTVTLLLGAVLFDGICRAEETFSKNQLKAAVNECNDTYFLSQKSWDSVFTTGSLDDEKDLVAKCFFECVLLQTGAMDDKGTINSDVTKAVFLASHDGTAVDGHGELIDMCVPGRVETDTCEKAYALVKCVTVEELSRRQAR

>OasiOBP1

MWARLSDCAALLLLLASAARAWDVNMKLTGRIMDAAKEVDHTCRTSTGVPREMLHRYADGQTVDDDDFKCYLKCIMIEFNSLSDDGVFVLEEELENVPPEIKEEGHRVVHSCKHINHDEACETAYQIHQCYKQSDPELYSLVVRAFDATIGDD

>OasiOBP2

MLLLLAAAVRGQDDEMREMMDQLHQTCVGESGVSEGNIDAARKGNFIEDANLKCYMKCIFVQMTCMSDDGVFDADTAIAMLPDNLKDVASKALNACKGEKGSDACDTAFKINQCLFKQAPKDYILV

>OasiOBP3

MDKASAAAATAFLLIAVAALHAQALSLEQLRQTSKIVRNMCLKKTGVDLALVEGIQEGQFPDNQDLKCYMKCCMGAMQVLRQGRYNVNAAKNQAEKMLPPDLKDRFLSMLDACSDRGDGADDDCEMAYQLTKCSYETDKEIFLFP

>OasiOBP4

MRTSAAAAAAATGAALLVLAAVASAMEMTPEFMEIVNKCKTEHEPTEDELKGMMALKVPESSNGKCFMGCVLQEIGVVKDGKFDKEEAKKHAAAKMTDKDELEKHMQLIEKCSQEVGGETDSCGIGPKLMECIKQFAPEFDIALPKPSE

>OasiOBP5

MRTYLTLVFAAAALFAVAKADAEKVKEAVEKCKSSENLDSLDGLKSNKAPSTEEEKCFIGCMMMDMKLLSSDGQYDAASTKEMINSCEYLKDKPDEKSAALEVADDCAGKATGCSGHCECGPKAVGCLINGMVDKGYEESFARIDKMLQNLE

>OasiOBP6

MGAAVAAAVLLLVAVTNAEDSLMEIVIREVKGCMESEHLNSIGDLRSYNDASSPEQKCFLGCMLKKFKALDADGQYDAEGLKATIEHCPRMKALPNVQKAALQVADECAGKVTGCSDYCSCAPLAAKCLHEGMKNKSFQTIFIALDEALDKMQS

>OasiOBP7

MLLVAPSKTHEQDFTKGISDVKVCMASENLGSLDGLRANKEARTAEEKCFIGCLMKFVEVLNSDGQYDVALFKDHINRSPDLAKMQQKKAALLEVADSCAGKASACSGHCECGVIVANCLAEGMEAKGEETIYDLLEKIFAKMDA

>OasiOBP8

MQAPQLLLAALALCLSAAVAAQQAPWCPTTASQGVQEDMGQCAEEIKDAILREYAKTVASRRTRSAEMSEEDRLLVGCMVSCLFRKGPHSRLQTGSKLALAELGAMRLFSDGADDARYRNATATAVRRCSASSRSLLPDDGGPRHECELGFFMFECVSDQITEYCQWQPE

>OasiOBP9

MAAALLQPTKGDEVWHNTDIPATMAECNATFRLGWRCWDNLLSDGHVIDESKYQQKCWFYCLLDKTGAMHADGAFDKDLLKTVLQGFPNGSSLAHLDETTYTCVAQRNEVDLCERAYAVVKCIMTEELSRMHHSS

>OasiOBP10

MKALLVACVAALGCLAVAVAAISESMSRAEEAAAKIDLPELFEECNETFTTPKATLNYFFSHGRLQNENDYGSKCFIHCLTDRSGEIDSDGNFDVDLIKVMTRRFPNETNIEGLNEMVETCVADRGETDFCERAYGLVSCLIKEKLTRLGHSH

>OasiOBP11

MSLAARLFSVTLLLAPVLFSDISTAGEVFTMSQIKAAVNECNDTYFLSQKNWDSVFTTGSLEDEKDLVAKCFFECVLEKTGAMDEKGTINSDITKAVFLASHEGTGTPVQGHDELIDMCVPGRDETDICEKGYALVKCVTLEELSRRHARK

>OasiOBP12

MFYFYAFTLCCLLWVLFHCSVNCVDIDIETIWRECNETFPASEESLISFGKNGTIPDENDSTARCFADCYGKKTTMLTSDGSLNWTTLDFIMRSYNMKPTATETFGKCQKDTSNVECMKSYLSLRCVAETIASLSNIR

>OasiOBP13

MVNHHQGVVAIAAALTAMAAAAPSSIAEATRFSKETVSKCQEKWQVSEEIIEEMQRNKGALPNEDSVEQRCFAECVAKEMGMINNGGGVAADKIVKMLEAVFQMASKETGEKLKLDSRALKRDLEACQFKGEDDECTNSYDTLKCLRTLGTSDNMRRYVTKES

>OasiOBP14

MFYFYAFTLCCLLWVLFHCSVNCVDIDIETIWRECNETFPASEESLISFGKNGTIPDENDSTARCFADCYGKKTTMLTSDGSLNWTTLDFIMRSYNMKPTATETFGKCQKDIPTSHQLKTLCTSLSLKHVNPSHQFHRGRGHGWLLKHVNRGPCTQRCKYT

>OasiOBP15

MRTSHVYTIFCAIIVTCYCDSVEVSDGPEEATMMKCAVELGFGHDEIQRIKSSPIPDETNENERCLMKCIGRKMKYLTSEDIVDVHHLLELSGEMIEKEGYTKSEMRQMLVECTKKTGTEKCMTAFKNLRCLMNAFK

>CkiaOBP1

MWARLYNCAALLLLLVAATHGWDVNMKLTGRIMDAAKEVDTKCRSSTGVPREMLHRYADGQTVDDADFKCYLKCIMIEFNSLSDEGVFVLEEELENVPPEIKEEGHRIVHSCKHINHDEACETAYQIHQCYKQSDPDLYSLVVRAFDATIDA

>CkiaOBP2

MRTSAAAAGAALLVLAAVASAMEMTPEFMEIVNKCKTEHEPTDDELKGMMMLKVPESEKGKCFMGCVLQEIGVVKEGKFDKEEAKKHAESKMTDKDELEKHMQLIEKCSQEVGGETDSCGIGPKLMECIKQFAPEFDIALPQPPSE

>CkiaOBP3

MRTYLALLVAAVALFAVAQAGPEDKLKESVEKCKASENLDNLDGLKTGKAPSTKEEKCFIGCFAMDMNVLNSDGHYDAASTKEMINNCEHLKNKPDEKSAALEVADDCGHKVTDCSGHCECGPKAVGCLIKGMMDKGFEESFASLDKVLQKVDG

>CkiaOBP4

MRVEVVAAVLLLAAVTNAEDSLVDIVIREVKGCMDSEHLGSIGGLRSTNDPNSAEQKCFLGCMLKKFKALDAGGHYDAEGLKTTIQHCPRMKAHPDIQKAAMQVADECNGKVTGCDDYCSCAPLASKCLHEGMKNKAFQTIFIALDEALDKMES

>CkiaOBP6

MKALLVAFVAALGCLALAVAAISESMARAEEAAAKIELSELFEECNETFPIPKATINYFFSHGRLQNENDYGSKCYIHCLTDRSGEIDSDGNFDADMIKVMTRRFPNETHIEGLNEMVDGCVAARGESDFCERAYGLVSCLIKEKLARLGHSH

>CkiaOBP7

MRTILLVSVSAMLLAATEADDADQMKVISEIKACMASENLDSLDPIRTNNEARTSQEKCFIGCMMKNLHVLNSDGQYDSALLKEHMSHCPEMAKDPQKKADTLLVAEDCAAKVTGCNGYCECGVVAGNCLAQGMEAKGHETKYTTGFETSWRRCMLRSGPSGT

>CkiaOBP8

MAVSVIAGALGLLAAALATTVTTDIPTEEILRWVETCNKSHPISQELLRSLATSGGLLADESDTNARCYLECYDRLVGVANSDGMLNVENVVAILIHYYPKIAEIGAESVAEIVRNCSSKSGTGQCMTSYLIRKCYTEGLGVKSPNVSIFDSSFS

>OinfOBP1

MAAAGPRGHLVSTVPTADMRTCLTLVFAAAALFAVAKADAEKVKEAVEKCKSSENLDSLDGLKSNKAPSTEEEKCFIGCLMMDMKLLSSDGQYDAASTKEMINNCEYLKEKPDEKSAALEVADDCAGKATGCSGHCECGPKAVGCLINGMVDKGYEESFARMDKILEKLD

>OinfOBP2

MKGFQICTLICAVVAHCMCDKEEAIKILRASVDKCSTGYGLSRETTQYIVKHNFIIQDENDENQRCFVQCVGQELGDFNSEGIFDVDHATETAEKWLQWNGRNKSNLREAMEECAKITGTGTCMTTYLITKCAMKAGE

>OinfOBP3

MRTILTVAVSVMLLVAPSKTHEPDFTKGISDVKVCMASENLDSLDGLKANKEARTAEEKCFIGCLMKFVEVLNSDGQYDVALFKEHINRSPDLAMDQQKKAALLEVADSCAGKASACSGHCECGAVVANCLAEGMEAKGEETIYDLLEKIFAKMDA

>OinfOBP4

MFYFYALTFCCLLWLLFHCSVNCSDINIWRECNETYPVPDATLISFRNNGSIPDENNITARCFTDCYGKKTNMLTSDGSFNWTTLEHILSNFKMKHTAMDVLGKCRKDPSDDECMQSYLSLKCVAETILSLINAR

>OinfOBP5

MSALYTCCVAAWLLMAAALLQPAKGDEMWHNTDIPATMAECNATFRLGWRCWDNLLSDGHVIDESKYQQKCWFYCLLDKTGSMHADGAFDKDLLKTVLQGFPNGSSLAHLDETTYTCVAQRNEVDLCERAYAVVKCIMTEELSRMHHSS

>OinfOBP6

MRTLLLFSVSALLMAADAEADEATQTKVMADIKACMASENLGSLGQLRANSEARTAEEKCFVGCMMKNLHVLNSDGQYDLALLKERANDCPELARDPRKKADTLLVAEACAPQVTGCSGYCECGVIAGNCLARGMEAKGHETIYDFLRKTVDKMDV

>OinfOBP7

MKAAMAPLATATAAILLLLAAAVRGQDDEMREMMDQLHQTCVGESGVSEGNIDAARKGNFIEDANLKCYMKCIFVQMTCMSDDGVFDADTAIAMLPDNLKDVASKALNACKGEKGSDACDTAFKINQCLFKQAPKDYILV

>OinfOBP8

MFYFYAFTFCYLLWMLFHCSVDCVDIDIGTIWRECNETFPASEEALISFGKNGTIPDEKDSTARCFADCYGKKTTMLTSDGSLNWTTLDFLMRSYDMKPTATETFGKCQKDTSNVECMKSYLSLRCVAETVESLTDIR

>OinfOBP9

MKALLAGCVAALGCLAVAVAAISESMSRAEEAAAKIDLPELFEECNETFTTPKATLNYFFSHGRLQNENDYGSKCFIHCLADRSGEIDSDGNFDVDLIKVMTRRFPNETNIEGLNEMVESCVADRGETDFCERAYGLVSCLIKEKLARLGHSD

>OinfOBP10

MALAVRLFSATLLLASVLFSDISTAGEVFTMSQLKAAVNECNDTYFLSQKNWDSVFTTGSLEDENDLVAKCFFECVLEKTGAMDEKGTINSDITKAVFLASHESTGTAVQGHDELIDMCVPGRDETEICEKGYALVKCVTVEELLRRQARK

>OinfOBP11

MDKASSAAATACLLIAVAALHAQALSLEQLRQTSKIVRNMCLKKTGVDLALVEGIQEGQFPDNQDLKCYMKCCMGAMQVLRQGRYNVDAAKNQAEKMLPPDLKGRFISMLDACSDRGDGADDDCEMAYQLTKCSYETDKEIFLFP

>OinfOBP12

MKLTGRIMDAAKEVDHTCRSSTGVPREMLHRYADGQTVDDDDFKCYLKCIMVEFNSLSDDGVFVLEEELENVPPEIKEEGHRVVHSCKHINHDEACETAYQIHQCYKQSDPELYSLVVRAFDATIGD

>OinfOBP13

MRTSAAATGAALLALAAVASAMEMTPEFMEIVNKCKTEHEPTEDELKGMMALKVPESSNGKCFMGCVLQEIGVVKDGKFDKEEAKKHAAAKMTDKDELEKHMQLIEKCSQEVGGETDSCGIGPKLMECIKQFAPEFDIALPKPSE

>OinfOBP14

MRTLLIYAIVCAIIVKCYCDSAEVSDGSEEDAMMKCAEELGFGHDEIQRIKNSTVPDERNENERCLMKCIGQKMKYLTSEGIVDVDHLLELSGEMIEKEGYSKSEMRQMLEKCAKKTGTETCMTAFKNMRCLMNRSK

>OinfOBP15

MVNHHQRVVAIAAALAAMVAATPSPIAEAIRFSKETVSKCQEKWQVSEEIIEEMQRNKGALPNEDSVEQRCFAECVAKEMGVINNGGGVAVDKIVKILEAVFEMASKETGEKLKLDSRALKRDLEACEFKGEDDECTNSYDTLKCLRTLGTSENMRLYVTKES

>OinfOBP16

MASALCLMAAAHRRHSHRYGIGIKNLNETMDFCNKTYPVSVDTLLERALNNGTLPDETNENARCFIECVERAKGTVNTDGTWNTTRAKQIEIDMNLALGRNISQDISNIVDECSTNSGSGSCMTVYLIKKCIDSRMSAFREPSQHNTHE

>OinfOBP17

MAVTLTAASALWFVAAAFAAVVTTEIPTEDILRWVQTCNKTYPVSQEMLRSLASTGGLLSDESDVNSRCYLECYERLGGTVNSDGKVNVEKAVTVMVSYYPKVAELGVDTVTEILKNCNSKSGTGQCMTSYLIRNCFIEGLNAKSPHTSVFDTSSYYI

>OinfOBP18

MAAAAAHLWCLLPPLLLAVCCALAAPSITSAEMRMDMMVIQHCNETHPVALIDMNKALINKKIEPQNTVFKCFVFCLLNKYEWMDDEGGFLIANMKHNLSDSHLDQLSIDFIVYKCSATGSSDKCERAYRFTECFWGEVTKFPENSDEKYDDPNLFALYQ

**CSP**

>OchiCSP1

MAANLTLCCLLGLVALCAGAAAQDPIDSFNVDEVLSNERLLKSYIQCMLDANEGRCTKEGKEIKKRLPKFVENGCLDCSPGALERAIKTLKHVTEQHPADWAKLKAKYDPTGEFAKKHAETWKQRGVNF

>OchiCSP2

MQALTLALVALVAVVATATAYTTKYDNIDLDEILNNERLLKKYHECLMSDTDSSCTPDGKELKATIPDALVTDCGKCNEKQKDGANKVIRFLVQKKEDLWKPLQAKYDPEGVYLTKHPELLKSE

>OchiCSP3

MARSQLVLLLAALVAVAAAENPLLSQLENIDVDAVLADPERVDAAVKCFVSDTEEHCNVRSKVIKSLIAEMLKTNCAECSDSQKAGVVKFMSHIAKNKPEDMKKLLAKYDPKGEALAKYGDSWREKGISV

>OchiCSP4

MASPLILSCCLLVAVVASGRAAPEDKLDSVDVDAVLNNDRLLRSHISCVLGADDGKCTPEGKEVKSRLPHLVTTGCADCTPRQLERAVKILKVVTVKHPEEWTKLKAKYDPTGEYAKQHAETWKKHGINF

>OchiCSP5

MSRPAAVLTLVALLAALAAAQDKYPDTFDKLDVKELLSDKSRIEAAIQCLKEAGEEACRPAGKFLKSVLAEIVATDCGKCTESQREKVSSFFAHVSQHYPEEMQKLLDKYDPTKEFRTKYQQSWAADGIKV

>OchiCSP6

MKTSLVLVAALAVIALAAADDMYTTKYDNINLDEIMNNERLMQQYEICLTETKDDNCTPEGKELKKDIPDALETECAKCNEKQKEGIKKVIKFLINHKPETWKKMKEHFDKDGKYTEKYKKLEDELKE

>OchiCSP7

MLLRRQGAAAGLLAVAACLVTVTLAAPQRCPSIEANDKKYTTRYDNIDIDSILKSDRLLRSYMDCLMDRGPCTQEGCLLRAAIPDALQTECSKCSAVQKKQAGRVMAWILENKRNYWDELIEKYDPQGNFRKKYGYDEDDDEEEK

>OchiCSP8

MKASLLVVAALAVIALAAADEMYTTKYDNINLDEILNNDRLMQQYEVCLTKDNDSICPPEGKELKKHIADALATECSKCSDRQKEGVKKVIKFLINKKPDMWSRLKEHYDVDGKLSEKYKKLEDELKE

>OchiCSP9

MKAALLLVSALAAIALAAAEEKYTSKYDNVNIDEILSNERLFSKYVQCLLEDADSNCTADGKELKRVIPDALNNECSKCNDRQKEGAKKVLKHLITHKQDTWQKLKTKYDPEGKYSKKYEEREKEILA

>OchiCSP10

MRLCALLLLLLVSVVCPAAAAPEETYQPIYEGTEVETILQDDAKVEAILKCLLSDTDDVCSKEDKQGKDMLPEALATQCAKCTEKQKQSMAKFFAHASKKFPDMFKQVAAKYDPKGENLPKFTGGRSHAA

>OchiCSP11

MQTPTLALLVVTALAAAAAADDRYAKYDHVDVERMLRNQRFVNAAIKCLLEEGPCTPEIRDLKKMLPDALKSDCSKCSPKQKDNVRKVVDFMMKQRAADWTRLSRKYDPEGLHQKRIEAKLKEQQLLQQQQQQQ

>OchiCSP12

MRTATLTQCLVAAALVALAAAAAPPEALYSKYENLDVDRMLRNQRFVAATIKCLMDEGPCTPEARDLKKVLPDALKSDCSKCSPKQKENVRKVFEFMMKERNADWQRLSRKYDPDGEHKKRLEAKLKEQQQQQQQEQHKTAEATTPAA

>OchiCSP13

MNRYVALLLCASVALVAAAEEKYPSTFDNLDLKALLADETRVQEAVRCLLEEGDAACRPAGKALKEVLPEIVRTDCAKCTETQHKKIGGFFGEISRRNPDLMKKLLDKYD

>OinfCSP_AYN71368.1

MKAALVLIAALAVVVVAAAEEKYTTKYDNVNLDEILNNDRLLNKYVQCLMEDGESNCTADGKELKKAVPDALSNECAKCNDKQKEGTKKVLKHLVNHKPDVWQQLKAKYDPDGTYTKKYEDREKELHQ

>LmigCSP_ADP65805.1

MKAALVLLVALAVIAAAAAEEKFTTKYDNVNLNEILANDRLFDKYAQCLLEDGESNCTADGKELKKAVPDALSNECAKCNEKQKEGTKKVLKHLINHKPDVWQKLKAKYDPDGTYSKKYEDREKELHE

>LmigCSP_CAJ01473.1

MKAALVLLSALAVVVIAAAEEKYTTKYDNVNLDEILANDRLLDKYVQCLLEEEDNNCTADGKELKRVIPDALSNECAKCNDKQKEGTKKVLRHLINNKPDVWQQLKAKYDPDGTYTKKYEDREKELHQ

>OasiCSP_ATI99844.1

MKASLVLLSALAAIALAAADDKYTTKYDNVNLDEILANDRLFDKYAQCLLEEGDSNCTADGKELKRVIPDALSNECAKCNDKQKEGTKKVLKHLVNNKPDVWQQLKAKYDPDGTYTKKYEDREKELHQ

>LmigCSP_CAJ01478.1

MKAALILVSALAVVALAAAEEKYTTKFDNVNLDEILRNDRLLNKYVQCLLEDSDSNCTADGKELKRNLPDALSNECAKCNDKQKEGTKKVLRHLINNKPDAWQKLKAKYDPDGKYSKKYEDQEKQLRG

>OasiCSP_ATI99845.1

MKAALLLVSALAAIAMAAAEEKYTTKYDNVNLDDILANDRLFNSYAQCLLDDGEDRCTADGKELKKIIPDALSTECSKCNEKQKEGAKKVLKHLINHKQDVWQQLKAKYDPDGTYSKKYEARERELHE

>OasiCSP_ATI99847.1

MKAILVLTAVLAVVASVAADDKYTTKFDDIDIDKILANERLLHQYELCLTEDETKCTPEGKELRKDIPDALETECAKCNEKQKEGIKKVIKFLINHKPETWQKLKEHYDKDGKYSEKYKKLEDELKE

>OasiCSP_ATI99855.1

MSEGAEGTAACVGSCRDVTAVYIAAAGGCGRRLSRHSLPTPHRTLANMKSCAFALLLVGLVAAAAAYTTKYDNIDLDEILHNDRLLNKYHECLLADTDTSCTADGKELKAAIPDALTNECAKCNEKQKAGAEKVIRFLIKEKPDLWTPLEKKYDPNGTYRQKYGEELKKVSS

>LmigCSP_CAB65179.1

MKSCALALLLVGLVAAAAAYTTKYDNIDLDEILHNDRLLNKYHECLLSDTDTPCTADGKELKAAIPDALTNECAKCNEKQKNGAEKVIRFLIKEKPDLWTPLEKKYDPNGTYRQKYGEELKKVSS

>LmigCSP_AAO16796.1

AAAYTTKYDNIDLDEILHNDRLLKKYHECLLADDDASCTPDGKELKAAIPDALTNECAQCNEKQKNGAEKVIRFLIKEKPDLWTPLENKYDPSGSYRQKYDQELKRVSA

>LmigCSP_CAJ01465.1

MNKTCSSALLLACLVAAAAAAAYTTKYDNIDLDDILHNDRLLKKYHECLVSSSDASCTPDGKELKAVIPDALTNECAKCNEKQKAGAEKVIKFLIKEKPDLWEPLEKKYDPSGSFRQKYGPELKKVSA

>LmigCSP_CAB65178.1

MQALTLVLFALVASAAAYTTKYDNIDLDEILNNERLLKKYHECLMSDSDASCTPDGKELKVSIPDALVTDCSKCNEKQKEGSNKVIRFLIQKKEDLWKPLQAKYDPEGTYLKKHPELLSA

>OasiCSP_ATI99840.1

MAIALSARALSYTRRSDMQALTLALFALVASAAAYTTKYDNIDLDEILNNERLLKKYHECLVSDSDSSCTPDGKELKATIPDALVTDCSKCSEKQKEGANKVIKFLIQKKEDLWKPLQAKYDPEGTYLKKHPELLSA

>OasiCSP_ATI99856.1

MSEGAEGTAACVGSCRDVTAVYIAAAGGCGRRLSRHSLPTPHRTLANMKSCAFALLLVGLVAAAAAYTTKYDNIDLDEILHNDRLLQNYYECLMKDDETGCTPDGVELKRTIPDALKTECSKCNEKQKEGTKKVLKFLINHKPDMWGNLKAKYDPDGTYAKKWEDKEKELHE

>OasiCSP_ATI99846.1

MRNSCLAVALLTTVAVVCGGYTTKYDNFDVDQVLHNDRLLKRYHECLVSDSDAACTVEGKELKSVIPDALQTDCSQCNEKQKAQAEKVISFLIHNKPDLWQSLQNKYDPDGSYRKRHDAELKKLSS

>OinfCSP_AYN71369.1

MRNSCLAVALLATVAVVCGGYTTRYDDFDVDQVLNNDRLLKRYHECLVSDSDAACTVEGKELKSVIPDALQTDCSQCNEKQKSQTEKVLSFLIHNKPDLWRSLQDKYDPDGSYRKRHDAELKKLSS

>LmigCSP_CAJ01476.1

MVTVSSAAPAKCASIAANDKKYTTRYDNIDIESILKSERLLRNYFDCLMDRGTCTQEGCLLRAAIPDALQNDCSKCSDVQKKQAGRVMAWILENKRNYWDELIAKYDPEGNFRKKYGYDEDDDEEEK

>OasiCSP_ATI99841.1

MAGKLTVCCLLGLLALCVEAAPQDPLDSFNVDEVLSNERLLKSYIQCMLDDGEGRCTKEGKEIKKRLPQFVATGCLECTPSQLDRAIKTLKHVTEEHAEDWARLKAKYDPTGEYSRKHADTWKQRGINF

>LmigCSP_CAJ01467.1

MAGKLTAVCCLLGLLAVLVQAATQDPLDSFNVDEVLSNERLLKSYIQCMLDDGEGRCTKEGKELKKRLPQFVASGCLECTPSQLDRAIKALKHVTEEHADDWVKLKAKYDPTGEYAKKHAETWKQRGINF

>SgreCSP_AAT39531.1

MAGKLVLCCLLGLFVLCIEAAPQDKLDSFNVDEVLNNERLLKSYIQCMLDADEGRCTNEGKEIKKRLPKFVANGCLDCTPSQLERAIKTLRHVTEKYPEEWTKLKAKFDPTGEYAKKHAETWKQRGITF

>OasiCSP_ATI99842.1

MASNLLVYCCLFAVVAVWVTAEEDKLDAINVDEVLGNDRLVHSYLECVMDDNDSKCTKEGREVKSRLPGLVKTGCNDCTPKQLERAIKTLKHITEKHPEEWKKLKAKYDPTGEYTQKYAETWKQRGVDF

>LmigCSP_CAJ01468.1

MARTLSLCCLLALFAVAADGAPQDRLDNINVDEVLGNRRLLKTFVQCILDEGEGRCTKEGKDLKQELPRLVETGCSDCSPRQLENGVKVLKHITENYPQEWAQMKAKYDPTGEYAKKYADAWKQRGVTF

>OinfCSP_AYN71350.1

MARALALCCLLALFALAADGAPQDRLDNVNVDEVLSNRRLLKTFVQCILDEGDGRCTKEGKDLKQSIPRLVETGCSDCSARQLENGVKVLKHLTENYPQEWAQMKAKYDPT

>OasiCSP_ATI99854.1

MARSSLLLLLALVALAAAENPLISQLENIDVDAVLADPQRVDAAVKCFLSDADDDCNVRSKVIKSLISEMLKTNCAECSEKQKAGVAKFMAHIAKNKPEEMKQLLAKYDPSGEARAKYGDSWRQKGIIP

>OinfCSP_AYN71362.1

MFRCVFAVLAALVALAATQSQIENIDIDAVLADPAKVDAVVSCFLNDDYQGCNERSKFIKGLIAETVKTNCGSCSDGQKAGVVKFLVHISRNKPESMKQLLAKYDPNGEALVKYGDIWRQNGISV

>OinfCSP_AYN71361.1

MVQMEPHFRDATMGRLCFALSLLSLLAALAAAQDKYPDTFDKLDLQELLGDKERVQAAIKCLKEEADTECRPAGKLLKSVLAEIVKTDCAKCTETQKQKVAGFFSFVSQNYPEQMQQLLDKYDPSKEYRTKYAQSWAADGIKV

>LmigCSP_CAJ01474.1

LSSSGASVALVVAAEEKYPATFDSLDLQALLADEARVQAAVRCLLEDGDGACRPAGKALKEVLPEIVRTDCAKCTETQHKKIGGFFGEISQRHPDLMKKLLDKYDPTGEFRKKYAKSWAEHKIQH

>OasiCSP_ATI99850.1

MKASALLLLLLLTAACVVAAAPEEKYQSPYEGTDVDAILQDDAKVQAILKCLLSDADDVCSKEDKQSKDMLPEALATQCAKCTEKQKHGMARFFAHVSQKFPDLFKQLAAKYDPTGENLAKFSAARRLSA

>OinfCSP_AYN71357.1

MSRVARVVICLGVAAVLATASAQEGYQTAYDSVDVDVIIQDDTLVQSIMKCLVSATDDLCGPENKHVKGLLPEMLATGCAKCTEKQKHSMTKFFGHVARKHTDLYKQFLEKYDPTGELLSKIKTTA

>OinfCSP_AYN71359.1

MAGLRLRLALVLVGCVLLGGAQAQRRGRGRAGGAAAASHDLDVDAVLADERTLAALLRCVVSRGDGPCSEQGKAVKAFVVSAVETNCASCTEQQQAAAEKVVRHLTTRRKSDWNKLVARYDPTGELRSRYRKHWEQRGVRI

>LmigCSP_CAJ01470.1

RHEADADARPADRDGGAGRGGGADDRYAKYDHVDVERMLRNQRFVNAAIKCLLEEGPCTPEIRDLKKMLPDALKSDCSKCSAKQKENVRKVVDFMMKQRAADWARLSRKYDPEGLHQKRIEAKLREQQLLQQKQQQQQ

>LmigCSP_CAJ01472.1

MRTCQPLCAALLLRVVGVAVAAPQDALYAKYENLDVDRMLRNQRLVAATIKCLMDEGPCTQEARDLKKALPDAMKTDCSKCSAKQKENVRKVVEFMMKERSADWQRLSRKYDPDGEHKKRL

**OR**

>OchiOR1

MQKPHGLVADLWPLIRMVQYSGHWMLEYSGGGVTALRAIYSSVVSVLVVTQFALMAVNLIQRSGDVNELAANTITVLFFLHPITKFAYFAVRSKAFYRTLATWNQSNNHPLFSESQARFHQLSVVRMRRLVMYVVAVTALSVVAWTSITFMGDSTREVPDPDNANETITEEIPRLMINTWYPFDAVSGMGYMLGFVYQLYWLTATLMHSNLMDVMFCCWLIYACEQLVHLKEIMKPLMELSATLDTVVPHTSELFRAASTLPTNEPLYGMGPDMSNGVTDGMTIRGIYSSQRDFSGFNRRNSALSTVREADAGGAVTSAGGIGPNGLSKRQEMLVRSAIKYWVERHKHVVRFVGNIGDAYGAALLLHMLTTTVTLTLLAYQATKIDSVDVYAASVLGYLFYTLGQVFLFCVFGNRLIEESSSVMEAAYSCHWYDGSEEAKTFVQIVCQQCQKSLMISGAKFFTVSLDLFASVLGAVVTYFMVLVQLK

>OchiOR2

MAAPVHSGGKVWDTTKEADAILGPTAPIMRAMGLWQPPGGRTAGAKLLVAVGIVLVFTISVACGIAKIVIDPPSEQDTFIEAIFTYACTITWEVRIVVILVRQRQLQQLVLDVLNMRKRFTENGTVLRESYRRRSTIVCVVWVAFPLVGIPMWFVEPALSKTVVTTAENVTTVIRKTPVIMWMPMDTQRHPNYEITYVIQIAIICSIVQTNVVVDLFFACLMINITADLEILNNNIANMRLNIEDAFLKKSAEGVKISAGWETTYKSKKSVAEEGEYYGNITAHASTTTESRVKMYRTLVKNIQHHQCIMSVINDLESIMSETSVLLLAVNSVNICMQALGFVDGFRPGAKRSTALKKVLTFPAYINQTAHYCWYGQEIIDQSERLLEAAFSCGWADGDPRFCSSLRIFMLQTSRPLKLQIGKIFPLSRNLFLQILNTSYTIFNMMINF

>OchiOR3

MGRPRKSLECPGAAFMRLTGLWLPQDPASRLLPAIRSCLSVGFLAFLAVSAALKLCMDTPQELEELATCSFALIICSVLSIRVIAFIWYGDTFRQLLQLLDDTKTEHNEGKNNDSTRRSYQMLVERVYLVFMVAVVCSLIGWVSSPLLFRAVLGNDDRSRQLPLPIWIPTDVYASPTYEIVYAAQSIGLVVIAVCTTCINTFFIHLMQGIGAEFEVLSDNLSSMQKNHEQTGKSRNEGGIWECQSIMEKQALSCADINAECITDEQIHRQLTKNFQHHQTLLRSISLLQTTMNVPIFILLTINMLNLCANLFIAGVLLQRDGGVSKALNSLTSIPPLLYETGLYCVFGHVLTDQSEKLLHSAFSCGWIERDIRFRRDLFMFMTATLQPVELTVGRMTKLSKQTLLQVLNGTYGLLNMLYHFHTNL

>OchiOR4

MARLKSGLAAATAQRVLRLNVRLLTLAGAWPPRARGACGRLFPLYTASIYFCQSATIAMGVWLTYDLWGDVDAIMLTYVNTFTLIGGLIKLAYFSSSVRDYRQLVEVLRDVTREQWPHCERDAELMAAFTEAYRKGLWMTLGPLAYLNILGPTWFFMPLIVRALGSEERLLPFVNMRESVTEIFPLYVTIYLVQVYSIFYWNFISVGLDMFFVTCMIHVAAQLKILNGRLSKLGKDQADDAYRAVLDPKDIRGFGGSQKRPFTRDGRSRDMYEELRNCIKTHQHILSLLKALQHVMSPVAMTQFMCSASGACITLFQATFNPEGNSTLKCLMFLPMPTFQMFIYCWGGHEIVVQEELLSLSGYRSAWVGTGRRISKVLHILMCNAQKPLQLTAGKFYPVNRDTFVTLINASYTFYTLMRQTRDQGSTVQT

>OchiOR5

MKGGAPEDLQRPLTWAASGQSILKLNIRHLWLCGMWPLPGSWIFKVYSYLCLAVGVWNAIECSLAVYFTWGDMEETTLVLITLFTLGCCTVKMAYFMRHQGEFNALARRVEMLLSLQSEFCSADPTMADIQRSAQRRGFRLTLTMLLVVFSQCFVWFPMPLVAGGDQRRLPFGQHAWDNNTNLYELSYVLQCAAGNWTTQLGFGFDLLFVSVMLILAAQLQILTLRISSLKPENSKIAIELGSKSEKALVSNFGDYMYENLCLTIKSHQKILRFVNHLEDTMSSVVLIQFFFSVLVSCVGLFQATYSTDYTGVFKSVSFLPVLGSQVFMYCWAAHSVTEQAEAVALAAYSCSWVEASGRFKHALRILVSRAQKPVVLTVGRLYPINREVFISLVNASYSYYALLGQMNKR

>OchiOR6

MAEKTASPSLAEAAEAASDLNYLLRALHWTAVLRHPRGAAGSPLLFRLCTIAMATFSVTFFFAEVIVLFRVGTADLDVFTMTLGVVDTDSTWLFRLVHTALFERAFHKLALQVANDFAEFLTWDDIPVVRAQCRTVRRFTLTYIWFGIGACAYYLVSPVSEEGLPFIMALPFDARPPLVFAATWTYCTITTLHVVVMTMVLDSFYVSLIAQLRIQLSLLSTKIVSLAKEVSAKPEQSSEKPQPSELHDRLLKCIRHHQTIIKNADLLERSLGAMLLTQSVSIGASACFQMYQVVTSSNGLQQAGKFGSHLFAMLAELFVYCYFGDDLITESENLALAAYDAVTSLQECPLSIKRSLLLLMTRAQRPLRITAGGFFPLSRESYVSVVNVSYSFFAILRNFKNEE

>OchiOR7

MPEAIPWSDTALWVNARFLALAGVWRPPWLQPPWFLLYRAWVLFTLLSFLVAQFQGLWYFWGNMDKITHDICLQIAVTQSLIKFFSFVLRQDDFFRMVHKIDNIRAEQSQTGDQEILSILEASYKSAKAITTYMTIMGSLITTFWGIIPSIMRKAGVFPPERELPATARYTSMDTVTPVYEILCTLQYFSMQYGFFVAVGSNLLYISIIIHASGQLEVLNARLARVGHTTVNSSANTSKKQKATEKILDLLSSEELAWNDLCYCIKQHQVVIELIKEIERLLSNIVLSQFMGATIIICVTLYQSSKNTENFAALFMLEAYLGVVTFEIFMYCWYAEDTLHQSSRLADSAYSCAWPDAPPKLKLALVLIICRTQRPLGITAGKFYFVSRETFVRLMSASYSYYALLSQVNDK

>OchiOR8

MPSVPVEQSLAAATQQACDLDYLLSFLHWTGTMRHPRAGPMSSRAFYVANSAVTLAFVYFVCSQVVILFRNGTSDLDSFTLTLSLIDTQGTWLLRIRHIAAFESHFHRLAYQVGRDFGQFASAEDVGCLRAGSRRMRAVMLLYLAFGLAECCVWLTAPASETGLPFVLALPYDVTRPGAYVATAVYCCFITLHTIMANFAADAFNVSLIVQLRMQLTLLNRNISNCNKAIEEPHPVAYKAESADATRPYLPFSASDVSYRLQKNILHHQAIIRNVELLQSCLGSILLGQSLSIGISVCFQLYQVAKSAESLQDAGKYSSYLFTMFAELFVYCWFADDLISESEKVAQAAYDAVPSLLECPVSLKRSLLILMHRAQRPLSITAAGLFPLSRESFVSIVNVSYSFFAILRNFKED

>OchiOR9

MAGRARPRESVLGSSATPLRLLGLWSAGGEDAGRAAGLLTGSVALAVICGATIISLLKLLMDRPRELEELVACIFVVTMHSEIFIKTVCFVMEGSTLRQLIQLLSEMRGENSVHERQEEIRRRYQRLGDRVFVAIMGMTMVTASLWMAAPLAQQVVSGPRRAPRLLPLPAWLPLDIYASPTFQLLYAAQVLFIPLVATITICCDCVFIDLMLRVAAELHILNDSVSGLRKIDLDATAATRNECKSIDVVSDDEINLRLVKSVKHHQAIMKSVALLEKAMSTPIFVLFVSMMIAICADIFAAAAILRTDEGTTKAAKLILALPPLLYDVGVYCTFGQIITDQSEKLMHSAYSCGWVDCDVRFRRSLLTFSMGARRPLEITVGSMYKLSKETFLQVLHGSYAMFNMLYGCQSNK

>OchiOR10

MATIPVTEGVTSPLRVQIPLHLAVLRAVSLWAPGRRSAQRHVYHVYHVYTAGVLALLAALVAGQVLAARYFWGDLLSVAMNTCVVFTYVSAALKAITFVTLRQFADTLIEDINIGLETFGESCRQEKEAVYAACARQSRLLMCVFVGKVGVAHAYWLALPATKMAACQTTDCRIKEGLPTVVWFPFPFTESPFYEVTYVFVSLALFYGYIVTTMVDSFFHALIIYNTGQLRVLNLLAARVCDPEPGAKACSEQQMLQRMAELVQGHTHVDRCVQRLSALLGPILLGQFLSNIASISITAFVATVMKADSQWLVKFGSYLYVVAEETLIYCWLGDNVLTESARLQTSAYSSDWTGAPRSLRRQLPIVLSRAHRPLLLTASKFYTISRETFLLMMNASFSYYAVLRQLNGD

>OchiOR11

MDGKKGAPIQLLGPEIPVLQLLGLWRAEQSAGNWVVALRAALTLVSIAVIPAGAVLRLCGEFPKELEELALCCFILFITIGSLIKGAFFIGEGEMLRELVHLLETTREMYGISGSSDCIRNHYKRMVDRLYIYFQVMSITAVIWWSGYPLLSNGTVEEDQENLRQLPIPLWLPLDVHSSPMYEMLYFLQTFCLTVTSEAAICLDVFFVRLMMLVAAEIEVLNQSVSTMHNINLKSTVSDDHMYVVRDGDQPWLVPGSGQPSQAANKSSEKVSDEEMFSTIVKNVLHHQTILRSISLLQSAMNVSTFIILFINMANLCFNTFVMAGLLQRDGNLTNAMKALFTVPSILFQTALYCIFGQITTDQSEKLSHSAFDCGWINCSSNFKRSLLIFMTMAGRPVEITVGKTCQLSKEMLLQVLNGTYVLLNMFLQMRGID

>OchiOR12

MKEKDVQQWGTSSEAEADETASLTWRETATSVLKVNVRCLFLNGSWPLPDCWIYHVFFSIVFASKLCNMVEAFVSIYLSEGGLQEITLVLPNTLLIASGVFKMAFFYSERGRYYGLVRRTDRLTVSQLAASGEVGAAIVSAAKRHCLKLTSAVFAFISLQIVVWFPMPLIAYPGQHKLPFIQIPYNNETEIPIYELSYALQCFSSFFNIFITLGMDCLFAVIMIHVAAQFEILISRIRSLGVATTVMQSKPSLNQHIRNPQTSINAQMQPKEMLEYQDRVTEAHDKMYSDLCGCIETHQQIISFARHMEAVMSPIALTQFAFSVLGACVTLYQATYSEDFNAVFRCAVFLPIPGSQVYLYCWAAHHVMEQSEAVLPAAYACFWVDASDRFKRTLRILMCRTQRPLVLTAGRLYQINRATFLSLVNASYSYYALLGQISKR

>OchiOR13

MSAEADRCLTSLPGPNSVLDVNIGVLKVAGLWPTRPFSLFAIYTAWIYLTQWAVFALDSMSLYYYWGNLNMITAVFCNLTSITAGIIKMTHFFVYKQSYYSLVNKLDALVASQQQITDPNVRSKSILNQTSKLNKYSTYIIVTYGNLVGVPWIILPFVMDSGETERTLPVVAWYGIRQDRSPVFEIGYVLQCLTIMYWFFASWGLDLFFGALMIHLAGQFKILNNRIENLRPKVENVSSGISSTTENEQKILNIKRDALQRRISFQHQNDELLLDLRRCIKDHQHMINFMNDLEQTVNFVVLVQFMAGTLVICVNLFQAALNVQDFSSMLKVCMYMFELILQLFIYCWCAHDVMVESERLSTSAYFSEWTGAPRRFTTALHILMARAQKPLTVSAGRIYTINRSTFVSLINASYSYYAILRQMSDR

>OchiOR14

MEDGPLTWRGTASSIVKVDVRILYATGVWALPVTRTYRLYTGVILVLVLGYSVEAVIHIWLVRNNMDAVTLAMSCYAVVFTSACKLLSFLQHEPNYWRLVRWLDALVADQKRYVQEKPPLQEIFDEAQKRARRIPKGLLIYNTSLILIWVFLPLLAPPGQRPLPFQQLPLSEKDDFTLFLASYLLQSIGMLYMNLVSSSLDGFFTAVMIQTAAQLKILALRLTDLRQNNEGQKQRIISDVNEKPPKNAMTDAVYEELCLCIRTHQEITSFVAHLESVMKSIAALQLATGVINGCLMIFPTAASSESGALLKCVACVPTISAQVLIYCLGAHSVQEQGEAVSLAAYSCGWPDMSHSSRRALLVLLTRAMKRLTLTAGGIYTIERATFLSLLNAGYSYYALLKNFNNR

>OchiOR15

MEVEAQARSLMGPSGSVLRLLGMWRPPGSASAGPHLLAALFVVATDALVSASSVVQLIVDTPTDPETLRDVFFQSTCSCAWAIRVVLFMQERRRLGRLVITLLDARKKYPEHVPGTRSSYDRHATMVFFVWQLLPLITMLMWAVEPMVGGPELVWVGNSSVEMRREPLVLWLPIDTQQSPFYEIVFAVQVVGIVTVAEVSILQDIFFVCLMIYVTAEVAVLDKNVSTLRLFRVDGNQSQMHGAGDVAAGGRGRYKDTESDESGEWSAAPPLPLPRNSLLYSQDMDGEKRRLYGSLKTNIQHHQTIILCVNELEQVMSNSTYIILLVNALTICLHAFGFVELFQGGGKGTAVVKRLLAFPIYLGQTALFCLIGQSLIDHSERLQDAAFSCGWPGTDGRFCSALLIFMQEASQPLKIRVGKIVTLSRSSFLQIMNVSYTIFNMLLNTQ

>OchiOR16

MADTARQCDALLGPSAVALSWIGIWSSPGRSPGFRQLGAIFVCTVDIIVPLLTCVQMLYYTPEDPAVFREVFFICTCGSSWAVKVVAFLLYQKRIQEMMLKLLDARRRFPDNGAGIREKYARIAYTLFKMWMGVTVTVVMWMGDPLMQSLTTSRPENSTRPLIFWLPIDVPHSPAYEITYAVQAFCIGFVAEISILLDIIVIVLFIHAASEIAVLNENIAGMGISKLRDRPNVSKRDMSLQRLENSTNDVSRSQASYVFSENLPASEVSTDNTGDLLVLDRTVMHWKLYSTLVSNIRHHQLIIEYIEDLEVVTSASLYLLLLANALNICLHSFGIVALFQEGATSSTAIKEVISFPSFLVQTALFCFFGQLIIDEAERLQFSAFSCDWPQADEPFNRALRVFMLQAARPLCVRVGKLVTLSRNSFLQVLNASYTIFNMLFNIEARD

>OchiOR17

MVRPSEDERQVLGLVGGLLGLAGGDRSSVSARLAEWARVAGAVINIVANISLVTTTGVELFTDSSEETERASITAFLFSVSTINFVKAVSLVRQHWRLRRLVASLVSIRSSFRDSEGARRRYARRGLVLGFIWVMMAEMHVSFWFLDPLISQALVGVKPDRQLPVPLWLPFNQTDPSNYASLFTLEATMLLSGVQLSLYIDALFASLIINVTAELHVLNSNIQSMKRAADHIGGQQSITAASSLAGTLQNAGTSDEAILVTNDTQSEIVSITSSISYFPSVQSKEEKDADNKMYRLLVNNIKHHQLIIKCVSELEKAISMGTFVVLSINILNLCSHIFSLVVMLERDSSTAARIRMFLAVPVFMCQSGLYCLTGQSIVDQSDRLINSAFSCGWADGDERFKRSLRVLMARSAQPLCIRVGKLIELSRATFQELLKGSYQLFNLVYQVHTS

>OchiOR18

MDAKNDMESTLSSTSVVRWNVIHLQMAGLWPVENSRLFGLYKAWLFFTQCHIVVLELIDLCSYTGDMSRAILNICAMATFFANVCKIFHFQLLKDDYFRLVTELDTFVSEQRSKYPNDETVQDLLNASTKRTASITKVVIVYLICWTFMVVPALFLIEAPYGVFPLMAWYPFTANIWPRYEIIITVHFLTIGHCFFTSWGMDLFFGSLMYHLSLQLRLLNYHLSNIRYRHKENGAIRDSIFETMSVSRSHVAGEIIKSETQKQIQEENDGAAENEMYENLLQCIKHHQRIIRYAEGVEKVANPVILVQFLVGVLVLCVVLFQTSSELETLTAFVRFLVYLLELLLQIFIYCWVAHEIFVESERVGEAAYSSDWPDASARYKSALRLVMRRSQRPITISAGKIYAV

>OchiOR19

MQKYRREGMEKPLSWNESGQSVLKLNIRHLWLLGVWPLSRSSVFKLYTGFTFTMGIWGLVECLSYVYFSWGHLEETTLVLIFTSTCSCGIVKLIFFVRDPQCFSSLVRELDALMDAQSEVTSKDPALAAILQDARRRAFRLTLGMLLFMMSQGIIWFPVPLIAYAGERRLPFSQHGWDNNSNYYELSYIVQCVSASYMSETSFGLDCLFASIMILVAAQLQILKGRILKLNKELIPVIGNESMPAKKSYDKMYDRLCVCIDSHQKILRFSTHLQDVMSPIAMTQFASSVIIACMALFQTTYGEDISAAFKCVSYLPIPGGQVFLYCWAAHNVTENADSVSTAAYSSPWVDCSAKFKRALRILIIRAQKPLVFTAGHLYPIDRGAFLSLVNASYSYYALLGQMNKR

>OchiOR20

MQKSSAQYSQTGAEVRRGLDLTTSKRVLHWFSCWGNGSVWYDAYVVLTRLSVAGVVATQVGLLPHVWGDLYSASLCVYLLLAVLSAWFKMVQFNLARPKVDELLREVTQELLLHADDVTDSTEDVFRLFQKRAGMFRRAFLVLGHLTILSWAIKPLLDRLLGSGGTLSLPQDAWYPFDTTHSPYYEIAYLHQVLSLYVAAMCVVGVDVFFTTLVMYLTAQLQVLNLHLRAMHCRPRLPPDSHKEPTSDIPGAASKAVVAQKPKEEEGLPPADPDDPHVQLVRNVQHHEVIIRSVTTLEQAMGPCVFVQFLFNVIVICIYAFVLAGVHDMNIGSVVICLTNLISTCVENFGYCWFGNEIMAQSEQLAFSAYSSRWVGGDSHFQKSLMVLTMRAQRPLCLTVGSLYTLSRETFLTLLNGSYSLLAVLLQMKNSN

>OchiOR21

MGKQKGRSMRLEALDSIGTGDLTWDEVNRSVLGHNIRLLHYMGLWPLESSRAYHSYTVFNLALSAVAIVMKVVGACYDLSNIDQVTGALSTVLPMTGGLINGLFLLKNRPAICRMVRAVDRLVAHQLEYSEGDGQLAVVVDGARRKTLLVTVGVSGYLFVIASYWVVIAFFQPPSSRILPFVQLPWMAGSGLAHFWCTFALQLYTAPFCSYTTLFVEFFFLAVMLHLSAQFKILGSRFANVGRSQQSGAKAVAEWHLSMLDSDAVYEELRPCVETHQQLLRFVRFLDDVMSPFAMMQFVAGTLAVCVVLFQAANNQDLNTNLKCAGWLPGPSLELFIYCGGAHEVVHEGESLEQAVYASPWYAAQPRVGRAIRLVMTRAQVAPVLTAGHLYPITRPTFVTLVNAAYSYYALLSQMQNK

>OchiOR22

MVLGSAVVLSGAKLCADPPAVYEELIAVIFILVASVSWTFKAAAFVGQRERLQELAACLVASGRRYGGGGGGIRGQYRALARRVFIYTQQVITAVPVAMWALEPLLSGGQNTPLPAWLPLDLHAAPTYQLLCTYQAVAVTLSVEASVCLDMFFIVLMIAVAAELHILNDNLEAIRLQPLDKLPLKSADDSATPWRRNVSSLADKQLPCSQQDEYHRENRKSANVLYGTADAQEVMYRSLVKNIQHHQLILKCVKELETAMNYSIFVLLFLNMVTICTLIISTTVLLQNDSDPTSLYKMVSSLPIVMFQTGLFCIFGQMIIDQSEQLPGAAFSSGWLEGDVRLRRALLLMMRRAANPLRITVGRMYPLSRHTYLQLLNGSYTIFNMMYQVRGRSD

>OchiOR23

MRQWERESIRLMGGSAAALRLLGLWPDGGRRDGGRRVGDAPVGALLLVANDVSLGLMGLSYVLMVQARDLKSLSEAFFDVATSISLTNRILILMRQKVLVEELVTLLMEARKRFPESVPGIRARYQALATTMFWIWQILPLFPLMLWVLEPVLVARFATHEANATADSLRTTPLPIWLPGDVRRSPTYEITYVLQAVGLVLAIGLNGFFDIIYVVFMVHIAAELDVLNQNMSTMKTEGNNNAGDRGRYDTRLKLETDYESAVVIAKSQARSLPFPSDLDVEDRMYLILVENIKHHQLIMAYIRKLEEFMRTSIFFILLTNALSICLQVLGLAVMLQEGVDLPSVLKMMLTFPAYSCETGLFCMFGQTIIDQSDRLVNSAFSCDWMNMGSRFRRSLTIFMLQASQPLQVKVGKQVVLSRHTFLRLLNGSYSLFNMLHGVQK

>OchiOR24

MALDQKRSLAGFGLNRWLLRCVGLWPDERGSPWRPTRRPVALLLQGCLLAVVSGELAAMRRYWGGELSRTTINACIILLVAVAFCKASSMLLLRRPILQLMHTLQDNSVPQNEGEARIYSAAARHAHLLTMVLCVDYAIVAFIWDSLPLLNFLRQTPEGRYNNSEAIFSQYPIIALHPWEVQSGAAYAFTFSLQVVGGAIFTMTHLSCDTFLLSLLIHICSQIDVLCASLRQLGRRAEGAAAEAEAGAEHAGGGRKEAGGQAADQRLYGELVACVRHHQSIIGYVEVLQQVLSPVALAQFMCSMVIICLSGFGIAISNDFGALFRYSVYFSGAAIQLLIFCWYGEVLITKSERVSEAAMGCGWTEARGRRFKSSTLILMIRAQRPLALKGSKFYVVSLKTFVQLLNASYSFFAVLRQLNESGHREEEGVMTSL

>OchiOR25

MKSRDEDKTEMPESWSASGHSVLKLNIRHLCLFGVWPLSRWDIFYVYSVLSFVLEIWYVCDAATSFYFLRGDMEEATLVLISTFLNSSIIVKMALFVWHSRQYVALVRRLDALMQVQSGPCSRDSVLGELLWRSKRTAARFTKGMLLFMVSQYATWFTMPLITNGTERRLPLAQHPWDNNSHNYALFYALQCVSGVWMSQISFGIDCLFVSIMILVAAQMKILATRVVSLKIGGQEYSPKMGESISPGDKVYRDLCLCIDTHQQLLRFVKQLDVAMGPIALTQFACSVLVTCVTLFPTTYSTDISAVFLCAGFLPVPLGPVYLYCWAAHNVSEQVEEVSKAAYRCAWVEASERFKRALRILIVRAQKPLVLTAGHLYPINRAAFVSLVNASYTYYALLCQINKRSVQEAL

>OchiOR26

MGRLAVDADCLLGPGCTLRLLLGLWWPEGRWLCLRGLLLATLSQGGTLWLTAGAVMKLYMDRPEDPEELALCCMVGMLCIGYLIKNVTFIIEGSTLRRLVKRLSDIRKEYGQEEDSKKIRQRYLGITERMYFHFQVMSVSAVMCWVCAPLVSSLFKNSDRISTQQRQYPLPIWGPQSINASPTYEILYCLQSISTLCTCQCMICTDILFFHLMLIITAELEILNNNIAEMHEGHVETAGGKEDILYVGCESNDDKTISFKNGNSTIYEKTLSTHVSFNESHHQLSKIVGHHRSVARCVSLLQSAMSVSIYILLAINMANLCFVMFLTVELFQRENSFGKVLKTVLVMPALLYQTWMYCIFGQIITDQSERLENSLFGCGWPDRDSRFKRSLRFLMLATSRPLRIRVGKTCDLSRELLLQVMNGTYGLLNLLLHVIKAK

>OchiOR27

MAQSEVEYKLLLGSGATLRRLMGLWWPQGRQGRVQSAVCASVTLICLASLTTFSTLKLIMDTPPELEEITQCSFVITICFSFFIKAAFFIYKGDTLKDLLKHLSDTRNIYRSHTGSERIRHSNLKLSGRVYNYMLAAVLPAVAVWVSTPLLVRVFLSTEDYSAASNRLFPAPLWFPGNMYLTPTYEILYGIQSFSLLVAALSIICIDPFFVHLMMMVATELQVLNENISLLHKLNVETLRHEDEVWTLGVKSNNHELAYSISADASCSTHPWLEDVSDEKMYQQLVKNIQHHQEILRSVPLLQSVMNVSIFILLFVNMADLCTCMFVTSVVLQRDGSVTKALKPLLTIPPLLYVTGMYCIFGQILTDQSEKLVNSIISSGWVDCNARFKYDLLFFLMASRKPLEITVGKMSKLSKEMLVQVMNGSYALLNLLYHFHNAE

>OchiOR28

MEDKALAVGAGLCPCFRGLRVLGLWTSRPAPSAGCSRQCLAVASQLRTALLAVAHLVLPVTLAANFLNCRFDSLELLATNIFLFSGMTGVSSKALLFVLDKKRFEQLLLQMQQTRIRFADNSEDQVGARERCRKFATRLYYTQHSGVRFTVLLWVTLPATASILSSDPPSTRELPLPLCLPGVDLTLSPYFELGYVIQALCLFIAVEGMSLLDSSFLALMLNIAAELEVLNNNLVSLGSKKAPKNEKCSDLARRYPYTSSGRLDSNPDSDDDSYNQLLENIKHHQHIIRRARELEALMSRPTF

>OchiOR29

MALHTSLSLCVRLLHVAGLSPPEAARRRLSPAGLLYGAYSLLVVSFACHIVLPQAAGLVHFRGDMKVATENMCLMSAFVTTSYKLLSLLLRRPRVMRFVAALDAHVTAMAAESAEARAVKSRRDLWSRRLALFMVIQSTSAAAIWSVNSLRISLTKGFCLRTLPIIAWYPYDVSVTSNYEVTFFMQFMTLITGAFSNRSSDILIITLMTQVSSLLEMLNLKFCDISKRHESPNDKHRKEDGLNNMWKSAASVFPTAFGVNRVLEPVRATASENSSEDEMYNSLSRCIEVHQEIIRYARELEFLVNDILLVDFVCCMVVTCSTLYVSTALNENFGDMLSHFGYLIAMTYPLLLYCLSAHDIIEQSERVSMSAYFTPWSQAGKKYRRAVCTVMCRAQRPLTLTAGKFSVVSRATFLTIMNASYSYYNILREMNDSKRSD

>OchiOR30

MKKQEAGELKTWTEPQHLSWRESGQSPLMLTIRVLNFFGQWPLSGSRVYRAYTTFTLVSTAVTVTLTIVTTFLLEGLEEVTSTLTTLLPVATGFAKSVLLTWYQPDLTRLMRHLDTLILEQGGGSWKRARSAKAFREAHRKTLVVTLLVNLYFGVVSVYWPIVPLLEGSETKRLPFAQFPWMKTPGALQYWLTFFLQTYTTDTAGLITINVELFFLGVMNHISAQFKILGERLTDLHRSSQCKGTSFDEREPVGKRSGDIHEELSACIKTHQDFLRFAETLDDVMSPFAMAQFAAGTLSVCVVLFQTMHSQLISSKLKCAGWLPGLGLELFIYCWGGHQVAEEGEAMVLAAYSSPWYEASLRSQRDVLLMMCRAQKPPELTAGHLYKVNRAAFVSLVNMAYSYYALLSQINNR

>OchiOR31

MMEKEREVEMEEPLSWSASGRSVLRLNVRQLCLAGGWPLCGSPLFNAYTLFAFALGAWHTVESALCCYCSWGNMEEATMVLMTTFNIGGSTTKLAVLALQRRKYSALARHADLLVRHGAETRALPEPVLSARKRAARLTAAMMSLIVTQYVTWFPMPLVVNRSERRLPLAQHPWDDNRHLYALSYTVQCMAAAWMTQVSVGLDCLFVTTMMLVTAQLQALASRVEGIRVELRHCSAHNTEKVSDHKYMELCLCIEIHQKILSFVKLLETTMSPIVITQFAISVLVVCVALFPATYGTNITAVLLCAGFLPVPLAQLYLYCWAAHDVTGQAETVSSAAYNCSWVDASEHFKRTLKILISYAQRPVVLTAGRFYPINRAAFVSLVNASYSYYALLGQINSRSTDASH

>OchiOR32

MRSSKSKEVYAPLSWADSGRSILSLNIRHLWLFGLWPLHDFWPFYLYTACGLLLGFWNVVEGGLSAYYSWGDMDNMTLALLTGMTQGCGALKMVFFTCTKDHYSALVWRLDALLAMQSEFCSGDPALAAIQRASQRRAYRVTLGTLLFMLSQCFVWYPIPLVAHAEQRRLPFAQHPWDDNRHLYALSYLAQCAAGLLMTQISYGVDCLFASAMVLVAAQLEILAGRIKGMAIQVDFSKTEKTECSGKDVARRNTDQMYRLLRICIKDHLRILRFVSHLQDTMSPIAMMQFGVSVLVICFALFQATYSNDFSSALKCTSFLPVPCTQLYLYCWAANEVTVQAEAVAVAAYSCSWVETSPRFKLALRILISRAQKPLILTAGHLYRIDREAFLALVNASYSYYALLSQITKR

>OchiOR33

MSKLQEAAGIRTDLEVQQRFLRAAGAWAPSGGRLAYSCYCAFVTASLVAFVATQVSAMLHFWGDIISITTSSCVTFIYMLAIFKQLVLLKMRRSADKLLQELDACLKNYGRAFGQEKATIFGECARKGRLISRMQVYLGVSVCIAWLVLPILRVRKCSTVECRSQNGFPAIVWYPFSFTEAPVYQVTFVVVSTGLCYGSFVSTTVHGVFSALMLYVAAHLRFLNLMASHMCTTGQAGNAAASHKHRDTAEGAEESMRSCLRECVRYHIDIERLVQRLSELVGPILLGQFLSDMITISASAFVTKALRADLGLLLQYGSYVSGVIEQMLLICWCGNEILEESERLQVSVYSCDWTGGSPRFRKELRIFLCRSGRPLLVTASKFYTISRETFLLMMNASFSYYAVLRQLNGD

>OchiOR34

MDMKNRDTNADEEETEVRAFTWKDTADSVFKYNIRKLYLWGVWPLPGSHLFHIYTAFVLAVGFGGLVQDLVCVYANWGDLQEVSMALHHNLSVGSGFVKLVFFVTRRRKLYALVRRTDQLVSAQKELCASDEGLTSLLKTSHRRAVRMSLFVFSYITAQACLWLITPLVAYPGQRKLPFVQLPHPESISIYLHILMYFTQSFASFLVTFLGVSIDCFFAVVMIHAAVQFRILNTRIKALRPDSTAPSETSIRKPEFHDDMYNQLCHYIQTHQRLLRYVRFLDSIMNPMAMTQFTFGVMVAGVTLFQASYSPSASTIFKCATWLPTPGSQVYLYCWGAHNIMEQGAEVSMSLYSSRWVEAPSHVKRAMRVAMCCSQRPLCLTAGRVYAINKATFISLMNAAYSYYALLTQVNGRD

>OchiOR35

MDAAELPDPVVDLRRPCLFLRAIGLWAPARLRWLYSLYTLWSLLQLVVSVLGQLAGLQGHWDNLPTVSTSICLIVTSICTMFKASSFALRRGRVDALVRHVGSNLSRFCAHRPRARAGIVRGARARALLMFNTFLGMGGVALVFFYLGPAIQNVKDSRVKVQAAANETLDPLLGRNLAMLLWWPRGQPVETPAYQLTYVGVCYWLMLLYLSTSTLDTFYVTLIIYLSSQLKLLNVDFLSIAGDADEDDPVDAVKSLTKPEVVHKLSGKHQTEDHNTKVQRTQERLLECIIFHQEVIKTVDEMESILSASILVQFLASTLVICFTAFVITTAKNKQDLPTYITYLATMFYELFLYCWYGNELLAESERLQTSAYACGWPGAAPGFQRSLRVVMARLQRPVCLTAGKFYKISRETFLLLLNGSYSYFALLHQMNDH

>OchiOR36

MKHPATDIASLLGPGASLRRLLGLWSPTDGGVRFLHSLLAAMTLSCTAFMALCVCLKLYADSPGELELVTLCCLVTSVCVGIFCKAVLFVAQEGTLRQIVRLLADMREQFCTGDENQQTVRRYETLSKSIYFQCQTVSVLAVIGWALCPLLTHSVTHSDQEQWDAGRELPLPTWLPADVHASPTYEVLYAAHTFCVLVSVESCLIIDMFFIHMMLMVAAELEILNSNVSAMEHINVESMGKEGEGFIYGSKSNDKRLSLFNSGKAYGEPMLTENAVHKRLHQQLVKNVLHHQGILRSVSLLQSVMTVSISIQLFVNMANLCSSLFVSTVLLEKEGNVGKALNALVTIPPLVYETVNYCYYGHNVTEQSERLYYSAFKSGWVNSDARFKRSMLIFMKATMCPTVITVGKTCKLSKETLIQILNGTYGLLNMLYHMHKQ

>OchiOR37

MMTERETGMKAVAGWFPRTDLRLELWLLRAVGAWPPGRGQSTSASWLLYGGYLAFLLLTLVVFVASQVLTMMHYWGDILSVTTSICVFFMYAMAIYKLAAFLAMRRAAEQLLEELDGCLRMYGGSYMSKKAIIFSGCARKGRKMALMQTAMGLLVCVWWLVMPAIRVQSCESTECFMQDGLPVLVWYPFPFTESPIYEVVYVSVSLGLVYGCCIFTSLDGFFWALIIYVAGHLQFLNFMAQNMCSRESEGGPVTDSKRVSSTEPAELEEMRRRLKECVRYHNHIDRCVQQLSSLVGPVLLGQLLTDMVTISASAFIATTLTADSGWVFKYGSYLSAVIEKMFLYCWFGNDIMAESERLQLSVFSSDWTAASPRFRKELRIILCRTHRPLVLTASNIYTISRETFLQLMNASYSYFALLNHLNSE

>OchiOR38

MNDVEEQPGDELLRLVVAALRYGGLWRPVGAGALYRLYQGAVLSVGPLLVAGVLLSAGHVARDGGAALQVALHAYYCTVLLSNSAKTAVFATCRTRLGQALTVLARCRRSERSEGTEAASRRKARLMFALPQAMVVLAVATHSVVPLLGSGATICSSPEAADDGGCFSGRFPLELWYPRATHAAPLYHTVYILQLVAIYYTCHTAINVDLFFFAVTNHASYQLQQLNNALCRMGSRRTVLHSPSLQHLRRGSAESDHSPDDKRGSGPLSDMNRETVALVDQHRAMYQSLILLIRQHQTITRAIKELEPVISYALFGPILTNILNICLHMLVLTTERENMATNSKAFVGIVFNLIQNGLYCSFGETLTQQSDRLFTSIYSSGWENSCRKYKKTATIFMFQTRKPLQIKVAKLYTLSRRTFLQLLNNSYGLFNLLYQVKNPE

>OchiOR39

MDEKATSTAQEVRLLLGPSARWMEALGMLHLPGSPPLGLKVLKGVVVVAVDLLLCAPSGLLLLVDPPADAKIYREVTYCFFAILTWMIRTVFFMLERTRLEKLVTILLDLRKRFPDDGSGIRKKHQKKASMICFAWLFVPVLVLPSWLLQPLLERHYVTHGNITELQLRTPMFMLLPCDDQKSPNYEIVYALQVVAVVFAAQTSILMDTYFVNVMIFLTAELDVLNANISRMHLNVAAQRQTLTVEKSVAPEYSDADRRLFKATYFYKKSKTKAEVELPVSEPVYTNGASGEYTSLVKNIRHHQRIMICINELEAAMNKSFTIVLVLSTVIICLHAFGLVELLEGDSTRASVIKRMQTFIAYLIHNGFFCFLGQSITNQSERLAHSAFRCGWADADPKFKRSLVIVMRQASRPLVVNVGKFFPLSRSTYLQIINTSYTIFNMMLSLHYKDT

>OchiOR40

VQFVFNMLLICVYAFVLTTRKSDFGTLAKFGMTLESYLFENLLYCWFGNNLIEQSERLPFSAYSSDWPDGGPRFQQALRVLSLRASQPLQVTVGSLYTLSRQTFLHLLNGSYSLFAVLHHLNSK

>OchiOR41

MATPPTARLSVASAAAEASDLGYLLRMLHWTGTMRQPRATTWASCAFHLVHAVSVAVVVLFLSSLAAAMWRVGRTDLEHTTLLLSHCNTVIMWLLRLRIIAAHERDFHYLARQVERDFVEFLTPQDVPLLRDRGRLMRRVVQGYVWSGLVACVWWLVFPVIRNGFTVEGVPYIMVLPYDVTRPLVFVGNFLFCTAATLHVGVMTMASDSFCISLMVQLRLQLVLLSRNLISVAKEDEEIKQEKCVKDGLSTKTLDQTTHLQNRIRQNIRHHQTIIRNTELLEKCLGTTLLAQSVSISVAVCFQLYQVALSAERLVDAGKFGCYLFMMLAQLFIFCWFGDDLITESQRVSTAAYQSVTSLDGYPCSTKKSLLLVMLRAQRPLRITARGFFPFSRESFVMVVNMSYSFFAILRNFKEE

>OchiOR42

MSPVAMAQFLCSVGAVCVTLYQATFNPEGNSSLKCLMYLPIPALQIFVYCWGGHELMENGLSVSLSAYSSQWTRAGRHVTRALRMLMCRAQQPLLLHAGKLYPISRDTFLSLLNASFSFYTVLRHMKNR

>OchiOR43

MGAPAETPVNLLGRNARMLQLLGLYQQKDDHSKVKPVILSAFSLVFLLYHPVFAAMKLYMDPPEDLVEFALCSFSFIISDGVFIKTAIFIADRGMLHQMLEVLSDSRRLYGGEETSKKIRSRYENLAERVLLYMQVSTMLASVGWLGAPLVFRALLTASGESGEVPRKLPLPVWLPVDVQASPTYEVLYVIEAYCTTLTGLVTLCIDVLFIRLMLMVTAELEVLNYNVANMAKRREKVSDEGYNHGHQKMDTQDSYRADRAFPMEEISYEDGSDDQLYQQLVTNVRHHLIVLRTVDLLEAAMSKSIIILLFINMGALCSNLLVVGVLLQAGGGVTRPLTLTAMIPFLLYQTGMFCVFGQLVTDQSEKLMVSAFSCGWNESDARFRRSLLIFMAMVNRPLEITVGKTCKLSREMFLQVLNGSYTLFNMFYQVHST

>OchiOR44

VKACCLLLEQDSLHKLVTLLAEAKIKSIIDVQHNEEIRSRYGRTSLKIYQYLKVMIAMSTITWLFVAVPFRVITAGSTKIDWPTPIPIWLPVDMQLSPTYELIYLAQILCTLPAAAAMLGTDTLFFHLTLMVVAELQVLNDSVSALARVAPPHEEEAVFRIKDAGEQRPNYSGGINNKHNEGADSLTNLQETVTEKKNYFQLIEAIQHHEIIIKLVSSLQRLMDYSVSVVLLTNVLNVCFLIFTMSELLHHEKSSNAVLQTILSLPANLCESGAFCTFGQMIIDQSENLVHAAFSREWLEADVRLKKSLYTFMLMASRPLQIKLGGTVTLSKSTFLQVLNGSYSLINLLYHSPRSHG

>OchiOR45

MTSGRQNDVESPISWTESGSSVLKLNIRHLWLCGVWPLPGSWLFYSYAASVLGLGVWNAVEGPLAVYFCWGNLEQTTMVFMNTFTNGSGLVKMAFFLRNKRQFSSLARHVDALMSEQTEASSADPALGDILLTARRRAFALTLGMVLFMASQGLVWFPMPLIAHPGERILPFVQHFWDNNTNYYELSYLVQCVAGLWMAEISFGMDCLFASIMIIAAAQLEILSGRVLKLGQHSYVSDKTNQDTDKIYKELCRCVESHQKVLRFVSRLQDTMSPIAMTQFVCSVLVLCVTLFQATYNKDIITSLSSMTFLSNPCGQVYLYCWAAHNVAEKANAVSTAAYSCSWVEGNGRFKRAIRILISRAQKPLVLTAGSLYPIDR

>OchiOR46

MKDNGGRDYANDASPLTWGYTKKSLLWLNIRIMWAFGVWPLSESCFRYVTKCILFFLAVGNAVENALGAKYNLGDMEVVTYSLMNIFTTTAGMAKTCHFSFLQDRYCLLVRRVDGLVLSQRDYWESDPEMLAVTRRCRKMALQVTVVAFTYLTALCLVWSMSPLVVHPEERWLPFNPLPLEPAPMPLYYEFSYAVQSASTLIYIQISFAVDFFFAVVMIIITEQLMILNVRLSQLHLYSDGGKSKITATTVLENAAVEIRDEMYDELCRCIETHKDIMRLIAFLDNVMSPIVLTQFLLSVLAGCLTLYQQTYSPDGNAVLKCSSYLPTPAILVFVYCWGAHSILEQGEAVSLAAYSCSWFDGSRRFKRALRMIMCRAQKPLIVTAGKLYPISRATFVSLVNASYSYFALLSRVHNR

>OchiOR47

MAAPDVGSGSSGDLEYLLSLLHWTGTLRHPLADRGSSRAFYARNAAVSACVLLFVCSQAAVILLEGAADLEQFTRAICFFNTAAAWLLRLAHVAVRERHFHALALQVGSDFDEFLTQRDAALLRKRGRVLRRFVLVYLWFGLSAGVGWDVVPLVRHGVCGDGLPYHMPLPYRVDRPLPFVATWLYCVSMTMHVVVATMIFDSYNVSLMAQLRQQLSVLSEHIRALADEGRRVSLSVVYSNQTEDSPTHVDSNRVRFRLRGIIRHHQAIIRNVESLEKCLGGMLLGQCLSIGASICIQLFQVAMSAESLAEVGKFGCYLSVMLAQLFVYCWFGDELITESEKVSTEVYSTVTSLQGCPASLKKSLLLMMTRAQRPLHVTAGGFFPLSRESFVNVLNVSYSFFAILRNFKED

>OchiOR48

MDWESQAVEPLTWQYAAGSVVQYNLRWLHACGVWSLSGSRLYRIFTATITAMSMAHIAEGIVHLCTMHGDLEDYTVALSCLSIMVVGTTKAAFFFFNEAGYCRLVRWLDALVESQREHVRSRPIHEKTFNEVQKRVLRISKTFFVYSTSLLSVWFVAPLMLPEEKRLPFQQLPTINATALPMYTYEVSYVLQVISMFFVATAHVLVDCFFIVVMFHTSAQLKILTARIADLQLPKGEMNGLCESNKDKRLQSVADNETYKQLCLCIQTHQDITRFIGYLETVMNPIAMMQLAIGVFNGCMLIFPAAYSNEMGAVLKGVACAPLVSMQLLLYCVGAHSVREQGELVSLAAYNCDWPDASVSFQRTLLLVMIRGQKPLTLTAGGIYPIQRGTFLTLLNAGYSYYAVLKNCAGR

>OchiOR49

LVTFMTGPLVQNAKDQQLLATGLGNVTLNRQLGHNYPMLMWWFGGLPVDSPGYEVAYLVMCYWLVLMYICTNVPDAFYVGLINYISAQLRMLHLALPKMATLDQDDLLPEKLGHTSGLAIKGGVPVKVGAQDRAYARLVDCIRFHQEIIKCVEEMESMLSLTVLIQFFTSTLVICLTAITVITTELAYLPTYAAYLATMFYELYIYCWYGGEVYLESESLQFSAYSCNWPDMDARFKKTFRICLVRMQKPIYLTACRFYKLSRETFLLLLNGSYSYFTLLLQMNQKNG

>OchiOR50

RGEHQEQRMRWRLAQCVAYHCRIDRAVQRLSTLFGPILLGQFLMDIVAISATAFVAIAKNADSTWLVKYTSYLSAVIQQLLFYCWFGTDVLTESERLQTSAYSSDWVDASPRFRMELRIFLCRTHRPMRLTASKFYTISKETFLMLMNASVSYFAVLREINAK

>OchiOR51

MLRQVYNALHNADEPSRYLEFNVLMVRFMGVLVGRDLPGTLFTWSLLALLLSQGLAGVLDLVDNHGNIADITANLPVTSIVFSSAYRLFFFTLHRDRYRAIVDTVGARFVSSSSGVDMAVWLRRSRIISILYFTYGSFVAGTWQLHPLISAQLTSAAMAKESNGTFEGLPRELWEFPTRAQYPFDARQPYVYTVVFILQGAAIFVSGCMILVLDMMFITITLLICGQLEILKDKLRNMRKIAMGSSQNEDIGLVGKAMQRQALERRKKILNSNIETNKKNEIFHEDLVTKKINLLLGECVEHHNMLLSLISEIEFMHWSAFMVNFCVLLIIFSFAAFEVTSGTPTSPAKVVNLAEYLLISILQMFLLCDCGDKLVDQEISVSQAAYESEWYHCSESVKRTLQIIVLRARQPEHITVGKIAGLNLDTFSDMLSRSFSYFTVLRQIRDG

>OchiOR52

MSDRNEGVYFGAIINLMHFFKIWSPDGSKRTISFSVYILVPAYLFFFSLSCVEIYHNWGDMLSTTDAVNTFVIYLATSHKYFRLIYHENDLKKLMKTVENNFSVPIRLNDALQNSIIKSYVQEVKKLTILWTTLCFTTLCGFMILPLADGLFHYYTANATTEIEWKLPYRTWTPFNDYSTIVTVPLYIYHMFMGFVLIAEIPAFDTIYFSLINHACAQLKILQNSLRNILMISAQNTHSNERNSVTFDTMFDDDYVVEENTDLSSLPQAHSMNGALNPPSRNYNSLNHIRASELENKIQHNVGELVNHHERILEFVDGVEAIVNAVFLTQFLCSATLFCLTGFQLTLILKEQQLARFLNMMELLGAAIFEMGMFCYYANRVMDEGINVGKAAYDSQWYYISKDYGKSVSIIMARCTRPPKITFGKFADLTMENFASILQIS

>OchiOR53

MAQSDGEYKLLLGSGATLRRLMGLWWPQGRQGRVQSAVCASVTLICLASLTTFSTLKLIMDTPPELEEITQCSFVITICFSFFIKAAFFIYKGDTLKDLLKHLSDTRKIYRSHTGSERIRHSNLKLSGRVYNYMLAAVLPAVAVWVSTPLLVRVFLSTEDYSAASNRLFPAPLWFPGNMYLTPTYEILYGIQSFSLLVAALSIICIDPFFVHLMMMVATELQVLNENISLLHKLNVETLRHEDEVWTLGVKSNNHELAYSISADASCSTHPWLEDVSDEKMYQLLVKNIQHHQEILRSVPLLQSVMNVSIFILLFVNMADLCTCMFVTSVVLQRDGNILKAMKPLLTIPPLTIETAMYCIFGQMLSDQSERLVNSAASCGWADCDTRFKRGLLVLMTVAQRPVEITVGKMSKLSKQMLVQVINGTYCLLNFLYHYNRS

>OchiOR54

MTTPKRQETSHPRKMTDEELDSVAPCAARIGLLGYWRRSASREGAHRYLRGWLSCALVSCISLSAAEKLILDTPSDLAELTMTAFELTVPLTVVSKGVCFILQRDTIHSLIELLVDMRRRYAERDGIAGRRSACLQYVLTVQRVLLIMAVFIMGAWAMAPVLPHVLPFASSNATSVTWVTPLPLWLPLDTQRSPLYECLYLLQTVFLVTSLSSASALDAFFCNITLMIAAELQVLNDNISSLMGKQPVLEKAGQKLPAIEVFADDEATVQRFPVGDTEHPGLSTTGRRTPRNQTYQRLVENIRHHQDILRCASLLETSMKYSVPFQLLNSITSMCFVLFVCSASLQQGGGLSSALKTILCIPYLFCETGIYCIYGQEILDQSEELVHSAVNYEYIEAGADCRKTLLIFMLMSSSPLEIKVSKTVPLSKPTFLQILNGSYTLINMLYHFSVPVE

>OchiOR55

MPQDSPRTDLGQQTRLLRAAGLWTPQPRAGVRAPLYAAHAACVVGSLLFLVGSQLSAMLHFWGDILSATNNACVISSYSMAIFKFFAFRMMRGSIEQLIRDLDRCLQVYGEEYAAQKAAIFDACARQGRLVSRLQVSLGVGVYLSWALLPAIRARACDTTDCRVHSGFPALVWYSFSFTEPPLYEVIYAIISIGLFYGCIIFTSQDGFFWSLIIYVGGHLRFLNLMVSNMSSGERDAKSARVSPELEEKMCRRLKECVDYHNDIDRCVQRLSSLLGPVMLGQFLTDIVTISASAFVATMLKADSGWLLKYGSYLSGTIEHMFLFCWFGDDILRESERLQ

>OchiOR56

IYRSHTGSERIRHSNLKLSGRVYNYMLAAVLPAVAVWVSTPLLVRVFLSTEDYSAASNRLFPAPLWFPGNMYLTPTYEILYGIQSFSLLVAALSIICIDPFFVHLMMMVATELQVLNENISLLHKLNVETLRHEDEVWTLGVKSNNHELAYSISADASCSTHPWLEDVSDEKMYQQLVKNIQHHQVIIRSVHLLQSVMNVSIFILLFFNMAALCCYMYVAIALFQRNGIVPNTLKLMMTCASFMYQTAIYCIFGQILTDQSESLRYSAFNCGWVEGNARFKQDLQIFLMGAERPLEVTVGKMNKLSKDMLVQVLKGSYGLLNVLYNFHTVN

>OchiOR57

MLTKERRDQQRREGLLIDEAATFTWSETSRSLLKVNIRVLFFGGAWPLSETLLSYAYIAAVAMASLANIAEAAVGLALGPASLDEVTLVLPNTLTTACGVVKLAFFLRDRRRYFALVRRADRLVAPQEQLSGEAEAFLRGVRRRVRAFAVFVVAITCVQAVVWFPMPLIVHPGERRLPFAQIPWSNDTQVPVYELSYAVQCLSSFTIAIITLGFDSLFMGIMEFVGAELHLLTLRMSRVRLDEGDHNREKTGGSGFAAQTAKGGDRDYRELCLCVERHQEIIRFTRFLEEMMSPIAMTQFIFSVLSVCLLMFQATFSDDFSAVIRCVAFLPFPGGQMYIYCWAAHRITEKAEALSMAAYSCNWVEASQQFKRALRIVIIRAQQPVVITAGRLYPVNRTTFLSLVNASYSYYALLGHLNKR

>OchiOR58

MGLPSLDRELLACPPGSAFLRFVGIWQPQEAAFRYLPGARGCLSLVSIAFVAVGAALKLFLDPPQNLEELALFFFVAVICSELWIRAVLFIVHGSTLWQLVQLLVDIRAEHNQGEGNECTRRRYQRTVKRVSWFLQVGMLGSLVYWLSSPLVSRVLLGFDHSSRQLPLPVWIPADVHASPTYETLYTAQCIGLTIVTASTACIDIFFIHLMQLIGAEFEVLNENFSAMQKDYEQYAETKNHGAIYECISDEQIHRQLTKNIQHHQMLLRSISLLQTAMSAPIFILLFINMANLCSNLFIAAVLLQRDRNFSKALKALMSIPPLLYETGLYCTFGHVLTDQSENLVQSAFSCGWADCDVRFRRSLLLFMTATVQPLELTMGGMTKLSKQTLLQVLNGTYGLLNMLYHFHKDI

>OchiOR59

MTEGQSLSEVDPKYHGAVTHFERSLLQQQMYCDLANNIRHHQLIIKCVRELEAIMNLPIFVLLFLHMVNICAQIFVTSLLLQKENDSTTMFKVLFTLPIYLYETGLYCVFGQIIIDQSDRLPSSAYSGAWLRGDARFRRALLLLTSHAARPLSLTVGKTYTLSRHTFLQILNGSYSLFNMLYQVQ

>OchiOR60

MEGEDALLGPGATALKLLGLWPLRGRGGRLLSALLAAGTLVSLALLVVCVILKLYADPPVELEEITLCGLVISLCVGFILKAGLFLAQAGTLRQMLRLLADTMEQFQTGDSNKLTRHRYQKLSKNIYFYFQIMAVPAVIGWVLSPVVSNSIASSDHEQMVAHRTFPVPVWLPEDFYMSPTYEILYAVNAFWALLTTECGLVVDIFFVHVMLMIAAEFEVLNDNLSAMGHLQMMRTDTETFISRYEMNDSRLAVLNTGQSLGEQRIAENAAYEWIHRQLVKNVLHHQAILRSVSLLQTAMDVSIFIFLFIDMANLCCSIFVAVVLFQREGNLGKSLNALLTIPALLYETVVYCIYAHIMTDQSERLVYSAYKCAWVNSDARFKRSLIIFMTATMHPTVITLGKTCTLSKQMLIQVLNGTYGLLNMLY

>OchiOR61

MQGLYYFWGDASLIIQDVCLLITTISGIFKFFVFVIRQEDVFAIVRTIDDRRREQAKLGNPRITAVLDASYRSGRTITIWMAGIGGTGPVVWAVSPAILRVLGVGPPERELPAMARYTSRDTESPIFELLNILQYFSMQYSYYAAMCLDLFFSCLIIHVAAQLEVLNVRLSQIREDLYHSGQSRHPRDGTSNEDTEEHSAFIQLCECVEHHKDAIKLVDDLESLVNPIILSQFMGATIIICVTLFLITTNKQHIAALVRLQAYLAVVTYEIFMYCWFGDDVMYQNSRLVNSVYTCGWPGAPQKLQKALIIILLRAQRPLGLTAGKFYHVSRETFVSLMKASYSYYALLNQMNN

>OchiOR62

DDLLGYSIAYFIQCHAIFYWNFISVGLDVFFATAMLHAAAQLKILSHRLSRLGKGAGYHHEQRSHWDDLQGDAAPVRLIVPHQETNDMYSQLRSCIINHQEILRLVLFLETVMGPVAFIQFLCSVVAACVALFQATFNTEGNGVLKCTMYLPTPAFQIFIYCWCGHEIMEEGLSVSLAAYSSGWVGAAQRVSRLLRVVMCRAHRPLQLTAGKLYPVNRLTFVSLINASYTFYALLRQMRDR

>OchiOR63

MKEPPAYWFLYVHQCMATFFFCSIDMHTDCFFATIMAHISMQFRILASRLAGLQLRENKQKSKLYLELDALSTVHDDMYNELCLCIDTHKELIRLVGLLESLMSPVAMLQFLVGAVSSCVVLFSATYSPDSSSAMKCWGSLPLLLTQLFLYCSGAQHILDQSEQVSLAAYDCAWCESSSEVRRALQLVMSRAQRPLALTAGSLYPVKRATFLSLVQATYSYYTLLQNLDN

>OchiOR64

MLMWWYNGQPEQTPSYQLTYMLMCYWLMLIYLACDVQDAFYVTLIIYLSAQLTMLNAALANSLNLGNGEDGRTSTGRRSLPTRGWRRDVERVLPDKHGDRDRLVECIKFHQEIIKSASEMESILSVSVLMQFMTSTLVICFTAFTVVTAETAYLHTYITYLATMFYQLFLYCWYGGEMVLESEKLQLSAYSCAWPDADPVLKRCLRIMMSRLQSPVKLTACKLYGLTRETFLLLMNGSYSYFALLHRMNQAPS

>OchiOR65

MPLLLERDRSLPTIAWYPYDETVTPFFELTYLLQGISTFYCCITNVGLNVFLVSLIIYVSDELQNLNCRISSIDYSTGYNCSCSEDKQHRSSKTALDDDGDRNVDTYKKRSNRPRQRAQFCCVVKAQDYLCRCLQYHQELIRTVKKLET

>OchiOR66

MGGDTQETEPLTWQYTAHSVLKYDLRILHFLCLWPLPGSLMFRTLTGVVIALCVGNFVEAVVSLCTLSGDLEAYTLALSNVSVVIIGVVKVAFFLRHERKYCHLVRWLDALVAAESESSRGRPLLGTIFAATRKRAARIAWGLLLYNCFLLVIWLTMPLTTPPEARILPMQQLPFTDANAHPLYEISYAMQGLSIVFIGLINVHIDCFFTVAMIHTAALLGSLASRLGDLQVHNTSSSSKGYEQGKRPLSAHDMFSELCLCIRTHQEIIRFVQHLESVMNPIAMMQLALGVFDGCMLIFPAAYSPETGAWLKCVITGPTVAVQLLLYCLGAHSVREQGERVSVAAYSCGWPDASARFRKAVQLVITRAQQPLSLTAGGVYPIQRATFLSLLNAGYSYYAVLQNFNSH

>OchiOR67

MVAQETLGREALGETREPRDNVLGRNVKILRFAGVWRPASRSGWRPCLFPLYFASVCGSLLNIITLDMARSWLLWGDMTEVTFALVSAMTNLNGVVKMLHYIRHSEAYSRLVSDLSGLVALQRPHCESNAARMEAFDQACRTAARLTVGCLAYMGVLGQMWCVVPLASQDPPDSRESPLPLVSLPGLRRENRGWYTFAFLVEGHAVLYWNFASLGMDMFFSSAMIHVTGQLNILNIRLAQLRQEGSTEDSYNGFNRSRNSPQQGSANGSARMYIELCECVKHHQAILRYLEFLESVMSPVALTQFLCSVVAVCVTLYQITFNPEGSGVIKCAMFLPIPALQIFVYCWSAHDIMEAGLSVSLAAYSCAWVGVDRRVARALRIVMCRAQRPLLLTAGKVYPVNRDTFLSLINASYTFYTLLRQMRNREIDNES

>OchiOR68

MDWDSQESQSLTWQYTADSVLRYNVRFLHVCGLWPLYGSRLFRVFIVATLILCFAHIAEAGINLCTLRGDLEDYTLALSNVTAVIVGVLKAAIFLRKEDGLCLLVRWLDALVERQRDDLGDQPSLQATFTEARKRAGRISKGLDTYNLSLLVLWILVPVMAPSEDRRLPFQQVPMANTTTFPLYELSYAVQSISVILICLISVHLDCFFIAVMILTGAQLQIVATRIDDLHLRNVAEKKDLTIGDTYKELRLCIQTHQNITRFIEHLERVMNPIAMLQLAFGVFTGCMLIFPAAYSSERGSLVKVVVTMPAVSVQLLLYCLEAHRVREQGELVSLAAYSCGWPDCDVSLQRTLLLVMTRAQKPLRLTAGGVY

>OchiOR69

MGWDSHEGQPLTWQYTRDSVLKYDVRILHIIGVWPLTGSRWYRCLVTVIITLCLGHFVEAVINLCTLRGDLEDFTLALSNVSVVIVGGLKVSFFLRHENGYCRLVRWLDALISSQREYVKGRPQLEGVSVGAQSLAVRITRGFCMYNASVVLAWVLLPLTAPPEAKRLPFQQLPFTEQSPFALYALSYAMQGVSMLLIALISVQMDCFFTAAMIHAASQLRILSSRIADLQLRNPDLKQQEKASFDVMYKELCLCIQTHQEITRFVKHLENVMNPIAMMQLAVGVFNGCMLIFPATYSSENDALLKCLAAAPTISAQLLLYCLGAHSVREQGEAVPLAAYSCGWADASTSFRRALLVVMTRAQKPLSLTAGRIYPIQRATFLSLLNAGYSYYAVLRNFNSR

>OchiOR70

AKFKPQVTDEISSHNELGLWVEQHKNVIKLVQNLEQLLNMIILFQFIGGTIIICVTLYQSSAKTGDVTTMFKLQLYLGTMLSEIFMYCWYADGIVQQSARLATSAYSCGWPDASQSLRRSVLVVMSRSQRPLTLTAGKFYTVSRATFVRLVNASYSYYALLRQMNDH

>OchiOR71

MDADSLVGSGLPLMRLIGLWRLEDDTRSVPQVLRLTPVLLAIALVVAGSALKLTVDTPEQFEEMTLCGFTTNTVGVIVVKGVMFVVHGATLREMVQLLYRAREQFTVADSNARIRSHYDKLADRLRMIVLVSMLPAMTCWMSAPLVAALGVGAAAGPAQRQLPVPAWFPVDLHSSPTYELLYALQVLGCTLACAVITSTDSFYIRLILLIAAELDILNENISNIRKRNTGPGDEGTTCRCQENVIISFSCKDCLNTFTSTAENTSDEIHKLLVNSICHHQDIIRMVSLLQQAMDTSVFILLFANMANLCSSFFTTAILLQEGGSTAKILKGLFVVPLVVFQTSYFCIFGHIITDKSEKLLNAAMNCEWIACDTRFKRNLLIFMIAAMKPLEITVGKICKLSRQMLLQALNGSYALMNMLYHYQHTTV

>OchiOR72

MKDKKQLESLDDTRPLTWDDTQRSLLKVNILILWALGVWPLPGSRMYSLFKIWLATLAVGNYIELGLGLWRNWGDMTEAALVPGQRDEPHSLDPVHVQRRGCLRHTLRGIFHPRRQLCYDQHLLPADTCHPGLLVLLECTQRPRRGSRGVGGGVRLPVVHGQRPLQEYPPHRHVPRPEGAGGHRGEALPHQ

>OchiOR73

MHRKDPGSKSGAVSASLRTAGGGTKNIDTHATLPIELTWKGTSHSVLKYNVRYLCLLGAWSLTHSRLYHLFSVTVFLLGTMHIGVAIFGAYLSRDNIEEMTLMLANLFTVCSGIAKLLYFVKDRDMYRQLLTLTHELTARQRSYCEGDPALKSIVVDSERLAARLTLSVPAYIASMSIVWVPMPFIAYANERRLPFVQLPLAKDVSSSTYALMYVMQSVPSLLTFNISFGGDVFFASVMINAAAQLRLLFHRIKDLRIGGRNGGPLSTGGFRSEYTMHNKHDRMYGELCTCIQIHQQIVSYLKLVGKLMGPIAMSQIVFSVLIACISMFQGTYSEDSNAAFKCLAFLPTPGAQVFLYCWGAHNLMEQSEAVSEAAYSCSWVEASGRFKRAISLLMCRAQKPLVLSAGGLLQINRPTFISLLKASYSYYTLLGRINNR

>OchiOR74

MAHTSVQFRILASRFAKLNAGEASLADAKPNTVRELSSQVRLGHDAIHQELRACIATHQKLLRLVSFLNDVMSPMAMMQLAVGAINSCMVLFPATYSEDDAAVMKCWGALPLLATQVFLYCSGAQNLMDQAEAVSLAAYSCGWVEASCR

>OchiOR75

MSSQRQYLTANKKENLLLVLESAHKKANFLTIVLTGYIYGLVFVWLPFPLILSPSERLMPLVQLPGNYFKDHLLAYITAYAIQSFVPLALIMVVDGLDCFFVSSVVHAEALLKVLSERIASLGKTHNYDFTVRRLPGPGQESYKTDVGNNSSITAELRSCIVFHQEIIEFLQKLEKAMSIMVLIQLSFSMLNLCMAMYQQTKIPDFTSALKYVMYVPFPTMKIFFYCWAAHNVKEQGEEVSWAAYSCAWP

>OchiOR76

ELEALMSRPTFVHLFYSLICISLSIVAFTVVLQTEGYSPKILKVIVVITAFVFQLGFFCLLGNSIIEQSERLMLSAFSCHWPDAHPRFKKALLVFMLRALHPLNIRVAKLYPLSRETFLQILNASYTLFNLVYQTNRRNGLDEPSS

>OchiOR77

MALAGLWPAARGGSEPAWRRVARWLLLANSLLTSAALVREAARARQSTVTDFACRCAFAVSLGCNNAIMLLMARRRRRLQRLMALATAIRPRASCRWVRRYAHTAAAFMAVQIMLWIAGCVCIGPHALLLDISYPVDGLVSGRARQLLLLLDQAAVAFNHMCSVSAFNSMFVHFVVIACQHLQRSIDDLTADNCDIVAVVRHHQQILRYIKELEDVYHIVMLWVFLPMMVVMCIIMFALLTITSVDIAFLEMLAIFLIYFITCGVISICGSMLTSKAERVVLAAYSSGWPERGRRFSGLVRVVMMRFQQPATLTIAKFTPLSINSFSTLVQESFSYLMVMLSLINEKEGEAQPGTVLEVASNYSSHY

>OchiOR78

MAAPPAAPEPWLSAAVLRLLGLWQPRTGGRSVGSLLAAGVSLGFPCWALSLSGLRLLMDRPQEVEELATCIFIPTMHGAMFIKSLYLVLRGPTLRELALLLSNMAAVNAPGGRGEQTRQRYRRLGDRVFLAMMATTAVTVVGWVCGPLVQQALSADSPGAPRSLPLPAWLPLDVFASPTYEALYLSQALFIALCSVATTSTDCFYIDLMLRIAAELEILNHSISDLGKKRASTAGSTQYKSADHFSGVEVHLQLSKNVKYHQDILRSIVLLEEAMSTSIFILFLVMMSGICASVLAAAAFLQNVGDLTRALKSILTLPSQLYDIAIYCIFGQIVTDQSETLVLSAYSSGWLEWDPRSRRALLTFSTMAARLLEVRVGKMYTLNRETLLQVLQGSYAMFNMFYGFKSNK

>OchiOR79

MHGLNFTSIPLYELAYTTQCTATFFWHLVSVGLDMFYASVMVHVTAQLAILNARFTNLRLQTGDVSGRSRASLDYMVVTDAHSKMYEELCDLVRSHQKIIEYTNYLEDVMNSTVLVQFLSSVLVVCVTLFQATINPEGNTVIKCCLYLPMPAFQIFVYCWCAHDLMDQGLEVCT

>OchiOR80

MIIACVEELQVAMNYSIFALLFFNMTSICLNIFVIASLLQSDADLITALKAVLTTPVFLYESAMYCIFGQMIIDQSEQLPQSAFSCGWTDADANSQRALLIFMLRSSQPLKIKVGKTYELSKVTFLRVLNGSYALFNMLYTFHGKK

>OchiOR81

ILSARLSNVGGSHAADGSERADFPRMRKKIRSCQQHSSNSEVLEELGSCINNHQHILSFLQVLQRVMSPVAMTQFVCSSVAVCITLFQATCNPEGNSSVKCFMYLPMPAFQIFIYCYGGHELIDQGLEVSLSAYSCEWFGATRRVTSSLLILMCRAQKPLLLTAGKLYPINRITFVSLLNASYSFYALLRQTRDR

>OchiOR82

MHRVVAHCVQYHDMLLRIVSEIEDLHWTSMLVNFALQLIILSFSAFEATASADMASPLKGTNLVTYLVLAIFQLFLLCRCGDKLMEAEEAVSRAVYESQWFDAPQGAKRSLSIIVMRSRLMQRVTVGKVVALNLVTFSETLSRAFSYFTVLRQIRTSN

>OchiOR83

WFPMPIVANREEWRLPFAQHAWDNNTNFYVLSYAEQCVIAAWTSQLSFGVDLLFVAVMILVAAQLQILALRIASMKTDIYQGKLDDEARCVNGIPATLSDKMYENFCLCVESHQHILRFLKDLETTMSPVVMTQFCFSVLVACVALFQATYSTDFTAVLKCASFLPVPGGQVYLYCWAAQAVTQQAEMVSAAAYGCSWVEASERFKHAMRILINRAKKPLVLTAGHLYPIDKPAFLSLVNASYSYYALLGQMNKR

>OchiOR84

MYETLERTRYRSWLVTVVWTVMCVLGALHWYFVPLLKGDSNLRFDAWYPFDSQQPTVKQVVYWLQFLCSFYSLLMLCFFDCLLVWLQQLLCVQLRYLAANIRDLSRNDAALHVDRRRMLAICVSHHDHILRAMRELNACVAPLLFLQCFKNMMVLCVVAFLASVAGVNDLLELSSLLLYFMAACQQLFFYCWCCEELTHLGLELGDAAYDSGWENWDVSSQKSIVIIMWRAQKPFIFKGAWFYTLNVATFIDLIKLSFSYYTVLQSMREG

>OchiOR85

VFLDTSFQILMLQMAAELEVLNDNIAAIHNRDVSPSEVKGAEDGKDDASVQNKKAEAFGRLRHGELLTQTAEDEMYSHLVSSIEHHQTVIGCLNQLETVMSHSTLILLSSNMVCICLHVFVTAVLLQDEIQFDKTFKMMCAFAIYTYQTGLFCLIGQTIIDQSERLVNSAFSCAWPDADARFKRSLSIFMLRSARLLEIRVGKMYTLSRETFLQILNGSYRLFNLLYQTNLEN

>OchiOR86

MASVSSVAAAAEAASNLNHLLRPLHWTAVLRHPSDATRPPLLFRLCSVVIALVVMCFICSSVVVLFREGTADMDVFILVLSTVDTAAIWIFRMVHTAVLERDFHKLALQVGHDFAEFLSWDDIPELRTQSRVVRRFTLAFSWFGIAACSYCLVSPVGSQGLPFILALPFDATQPLAYAASWLFCTVVTLHAVVMTIVLDCFNVSLISQLRMQLALLSNKIVGLAKDMSDGPIQSAETSTYKELHYRLHRCILHHQAIIKNADLLERSLGAMLLAQSISIGAAACFQMFQVATSADGLQQMGKFGCYMFTMLSELFVYCWFGDDLITESENLVLAAYDAVTSLQGCPVSISRSLLLLMHRAQRPLCITAGGFFPLSRESFVAVLNVSYSFFAILRNFKEEEP

>OchiOR87

MQSRKVLTSSCSVSAGAATSSTLCTNLQTCRWCLAHSLSMAVATASDAARDVITRRSSMGSSSLRAPIWSSVQLRTRSCPAAYTSSTSRKASRAPTQSATSSSVSTW

>OchiOR88

ALPAVVWGPFPFTESPAYEAVYAAVSVAMFYGYILTTTLDAFFFTLIVYTAGQLRVLNLMAAGLCAPGSPGQMRARLADVVCYHVHIERCVQRLCALVGPVVLGQFLADIVTISAATFVATVTEADSVWLLKFSSYLLAIAEQLLLYCWFGSDVTTESERLQLSVYSSDWPAAPARFQRDLCMVLRRGQRPLRLSAYKFYSISRETFLMLMNTALSYYAVLRQLHGN

>OchiOR89

MRQLPPLPARRRLQRQVVRATLATTAMLSTHIALQMLYCLLCEPDQLLTDIRYPPLDLGRPRVRQLLLVVLQGSVVFYNIYTFTAFVGLFMYLSVQVRAHLSAISDALTHQAADIDLRAIVHHHQEVLSFLSEMEAVYHPLLLLDFLPLVVVICLMAFVLISMEGVDLMFVQTLTIFFLYFLNVGTFCFFGTQITSQAEAVSLSAYSSGWEERGRAFSSAVLLVLQRAQRPFTLTAGKFAPVSITTFSQMMQDAFSYLMVMRTLIGSKDHASPAAA

>OchiOR90

MALFLKFIMYLLYMTVGMFIYCWYGQDLIQKSGDVRWAAYSCCWQGAPRAFTALLRVAMLWAQRPLTLSAGKYYRVSLKTFVTLLNASYSYYAVLRQMNDSQK

>OchiOR91

MLRFCRELSDALSPVLLSQFTFTIVIICQTALQTTMEPELNPRTMKFMGLLWLGISELFLYSYMGHRIMSQASDVQLSVYSCGWPDLDDRFKKSVSFIIQRAQKPLHFTIGKMYVLSLRTFVEMMRISYAFFTLLQQVNGD

>OchiOR92

QIYFYCWTADNITEQGEAISGAAYRSQWVGAGTQFQRALSTVMARAHQPLLVTAGRLYPVNRAAFVALMKATYSYYTLLRQLDT

>OchiOR93

MSPIAMTQFVVGAVNACMVLFPATYSTDVGAVLKCWAALPMVGIQIFLYCSGAHDIMEQAEAVSNAAYGCSWLGADRRRWRALLLVMCRAQRPLQLTAGKLYPINRPTFL

>OchiOR94

MVAIGSRAKLIFVQQRKNSSKGLDGGDVEGAPAQENVLWRNIMVLRLAGVWRPAGGWRARVYPLYFSVVCASMLHIGVLGALRTYSIWGNMTEVTFALVSAFTCFNGAVKMIHHYTHSETYYRLVSELNVLIDRQRPYCDQDDELMAAYQATYRRARRLTVGVLAYMFVTGAMWATVPLFMEFPPGDPSSPLPLVTITGVHRVYNNTLYSLGYLSECHTVLYWNFSSLGMDVFFGSVMIHVTGQLNLLHIRLARLGVDRAGEDLIGSSAFGKDSQQQKRDIHDPPSMYAQLCDCVKDHQEILRYLDFLEELMNPVPLAQFLLCVGGICLTLYQITFNPDGGGVIENILFLPIPALQIFIYCWAGHGIMEESESLSFAAYSCDWSGVGREVSNALRIVMSRSQKASLLTAGKVQPINRDTFLSLLNASYSFYTLLRQMKNLEEEN

>SgreORco

MQKPHGLVADLWPLIRMVQYSGHWMLEYSGGLTALRAIYSSVVSVLVVTQFALMAVNPIQRSGDVNELAANTITVLFFLHPITKFAYFAVRSKAFYRTLATWNQSNNHPLFAESQARFHQLSVVRMRRLVMYVVSVTALSVVSWTSITFMGDSTREVADPDNANETITEEVPRLMISTWYPFDASSGMGYMLAFVYQLYWLTATLMHSNLMDVMFCCWLIYACEQLVYLKEIMKPLMELSATLDTVVPHTSELFRAASTLPTNEPLYGMGPDMSNGVTDGMTIRGIYSSQRDFSGFNRRSAALSTVREADSGGAVTSAGGIGPNGLSKRQEMLVRSAIKYWVERHKHVVRFVGNIGDAYGAALLLHMLTTTVTLTLLAYQATKIDSVDVYAASVLGYLFYTLGQVFLFCVFGNRLIEESSSVMEAAYSCHWYDGSEEAKTFVQIVCQQCQ

>SgreOR1

ASTDVSNHIQKPYEINMSNRNEGEYFGAIINLMHFFKIWIPDSSKRNISFSVFILVPAYLFFFALSCIEIYHNWGDMLSTTDAVNTFVIYLATSHKYFRLIYHEKDLKKLMNMVENNFSVPIRQNDAMRNSIIKSYVQEVKKLTILWTTLCFTTLCGFMILPLADGLFHYYRTNSTKEIEWKLPYRTWTPFNDYGTVVTVPLYIYHMFMGFVLIAEIPAFDTIYFSLVNHSCAQLKILQNSLRNIVTISAQNVHSNEKNDVTFDTMLEERYIIEENIGHSSLSPAHKMNSVLNSFTPSGNCDRPLNHIHASELDNKIRQNVGELVNHHEKILEYIDGVEAIVNAVFLTQFLCSATLFCLTGFQLTVILKEQQLARFLNMMELLGAAIFEMGMFCYFANRVMDEGINVGKAAYESQWYYVSKDYGISVSIIMARCTRPPKITFGKFADLTMENFASVLQISYSYFTLLTRLNE

>SgreOR2

NADEPSRYMEFNILMVRSMGVLVNRGMLGVLLPWSLLALLLSQGLAGVLDLYENHSDIADITANLPVTTIVFSSAYRLFFFTLHRDRYQAIVDTVAERFVASSGSINMAPWLRRSRIISILYFTYGSFVASTWQLHPLISAQLASAEMRKESNATFEGLPRELWEFPFRAQYPFDARQPYVYTVVFLLQGACIFVCGCIIVVQDMMFITLTLLICGQLEILKDKLRNMRSIATDQRQNEDIGLVGKALEKQVQERRKMILNSSTDITKKDENFNEDLVTKKINLLLGECVEHHNMLLSMISEIEFMHWSAFMINFCVLLIILSFAAFEVTSGIPTSPAKVVNLAEYLLVSILQMFLLCDCGDKLVDQELSVSQAAYESEWYHCSESVKRTLQIIVLRTRQPEQLTVGKIAGLNLETFSDMLSRSFSYFTVLRQIRDG

>SgreOR3

PGQPLWATIQPLVWSCLLMHLLCELADIGLNITDVQQLGKNLPVSSLVGGSWYRLSYFTLRRDAYCRLVSKVGESFRRGAPGRMRRWLRRSRAYTLVYFVYGTIVCLFWLGHPLLIQQTTHTMFSTNSTNHSRMSETAEFPSGAWYPFDVRERRVYGAVYAFQCLAIYFAGMLIMVTDIMFITLMLLTCGQFEELGDKLRNCWEIAAARTLRRAGTTQERELHKVVAHCVQYHDMLLRIVGDIEDLHWTSMLVNFVLQLIILSFSAFEATASADLASPLKGTNLVTYLVLAIFQLFLLCRCGDKLMEAEEAVSSAVYESQWFEAPQSAKRSLGVIVMRSRLPQRVTVGKLVGLNLVTFSETLSRAFSYFTVLRQIRTSN

>SgreOR4

ACNMAVSGKDLQTNVLASGIPCLDLIVYLEPILRPLATTGMWSSSVFSNNVLKVVAMTAGLSLFGMFLVQLLAEVVGTPLLLAANGDIDRLIHDMSVLGIHLDTFWKWLFMLTQRRRFIQLLRQMESCMCLGVLACPTDRLALQDGYLQGQGIQRWAVPTKLYEVLERTRFRYWMVTVVWSVLCVLGASHWYFVPLLKGDDSLRYDAWYPFDSQQPTIKKMVYWMQYACSMYSVMILCFFDCLLVWLQQLLCVQLRYLAGNLRELSRSGGAFSVDRRQTLAVCISHHEHILRVMRELNAFVAPLLFLQCFKNMMILCLVAFLASM

>SgreOR5

TDEHKKLLVACVAHHSDLLSTIQELNSCAGPSFFLQCFENTIRMCMIAFMATTTVADQMQVWSSAQFFLAAVGQLFLYCWCGQQISHLTESISDAAYDSGWEEHDVSTQKNVAFIMQRAQKTIVFKGGWFYTLTTETFVELLRLSFSYYTVLRNINDR

>SgreOR6

LTLGCVACCSGVGCVWAAGPLLADGDALPYRLWSPLDVTAAPGYHITYLLSVTILAPLVEAFVNLNALYLGLMVLTSARFSSLNSRFAAISKEKDGSGRLATAELARCVALHVDLLRFCNELSDVLSPVLLSQFGFTIVIICQTALQTTMELELSPRTMKFMGLLWLGISELFLYSYMGHKIMSQASDVQLSAYSCGWPDLDSSFKKSVSFIIQRAQKPLHFSIGKMYVLSLRTFVEMMRVSYAFFTLLQQVNGD

>SgreOR7

QRQVKDVRTEAEATAMLRECARHHLQVCRFVQSVEEVAAPALTLQLLVSTFLLCMSAFTATQIPVGSPLLARVVIYMLTGASELLIYCKYCDDVISESGRVQQALYGSGWAARGGAFSSGVLIMMARAQRPLCLRAAHVLPVSLQTFTKVLNASYTVFTLMRQIKD

>SgreOR8

MLDKRDTMALQEKRSLVQFGLNRRLLRCVGLWPEERGSPWRPSRWPNALLQQGSLAAMVTSELAALRQYWGGELSHTTINACIILLVAVALCKASSLVSLRRPIIQLMHTLQDTSVPQNEWEERIYSGAARHARLLTLVLCVDYAIVAFIWDSLPLLNFMQQSPESRYNNSDAIFSQYPIIALYPWEVQTGPAYAFTYTLQVMCGAIFTMTHLACDTFLMSLVIYICSQIDVLRASLQQLGRRADRVGAAAAVAEAQADAELSVGRLGGGSDPVKALCGQGSEQELYQELVACIKHHKNIIGYVGVLQQVLSPVALAQFMCSMVIICLSGFGIAISNDFGALCRYCVYFTGAAIQLLIFCWYGEVLITKSERVSEAAMGCGWTEVRGRHFKSSALILMIRAQRPLALTGSKFYVISLKTFVQLLNASYSFFAVLRQLNESGHREEEGALASL

>SgreOR9

YIKELEDVYYIIMLWVFLPMMAVMCLIMFALLTITSVDIEFLEMLAFFLIYFITNGVISICGSMLTSKAESVVLAAYSSAWPERGRRFSGAVRVVMVRFLQPAELTVAKFVPLSINTFSKLLQESFSYLMVMLSLVNEKDNETQPAAILEPASNHSAYH

>SgreOR10

YLLRPLHWMAVLRHPSGAGRSPLCFRLYTFAMASFTLSFFCSEVIVLYREGTEDLDVFTLTLSVADTDIIWLFRMAHAIVCERAFHKLALQVGHDFAEFLTWDDIPVLRVQCRKVRRFTLTYISFGIGACSCYLVSPVSAEGLPFILALPFDATQPLGFGVTWVYCMITTLHVVVMTMILDSFNVSLIAQLRMQITLLSSKIVSLAKEVSERPENCSETYAQYELRYRLLKCIRHHQTIIKNADLLERSLGAMLLAQSLSIGASACFQMFQIATSANGLQQTGKFICYLFAMLAELFVYCWFGDDLITESENLALAAYDAVTSLQECPLSLKRSLLLLMHRAQRPLRVTAGGFFPLSRE

>SgreOR11

MPLTRGSATSLLCYRLYTVGVIVLNLSFFASTVIVLYSEGTADLDVFTLTLSVADTNGTWLFRMAHTFVCERAFHKLSHQVGDDFAEFLTWDDIPMLRAQCRVMRRFTLTYIWFGIIACAYYLVSPVSAEGLPFILALPIDAMQPVGFAVTWLYCTVVTLHAVVMTMVLDSFNVSLISQLRVQLTLLNKKIVSLAREMSERTDNSLETTDHAELHYRLQKCILHHQAIIKNADLLESSLAGMLLAQSMSIGASTCFQMFQLATRTNGMQQTGKSGSYLIAMLAELFVYCYFGDDLINESENVALAAYDAVTSLQECPLSIKRSLLLLMHRAQRPLRITAGGFFPLSRES

>SgreOR12

NHLLQPLHWTAVLRHPRGATRSPLCFRLCTIVMTALILSFFISEVTVLYREGTDDLTAFTMTLGVADTNIIWLFRMVHITLCERAFHKLALQVGNDFAEFLTLDDIPMLRSQSRAVRRFTLIYIWFGIGAVVYYLFSPVSDEGLPVILAVPFDAKQPLGFAVTWIYVSIVTMHVVVMTMVFDSFNISIIAQLRIQLTLLNSKLVNLAKEMSERPVHSSKTPAYRELHYRLEKCVRHHQAIIKNADLLEKSLGAMLLAQSLAIGACACFQMFQVATNKNGLQETGKFGCHLTVMLAELFEYCWFGDDLITESENVALAAYDAVTSLLECPVSIKRSLLLVMQRAQRPLCITAGGFFPLSRE

>SgreOR13

LTLLSSKIVNLAKLLSERPERSSETTACREVHNRLEKCVRHHQAIIKNADLLEKTLGAMLLGQSISIGASTCFQMFQLATTANGLRQVGKSGCYLYAMLTELFVYCWFGDDLITESENVALAAYDAATLLQECPLSIKRSLL

>SgreOR14

AVAAPATSSALTARSREIDPCIPDTMAAAGPAKASVAAVEAASDLGYLTRPLHWAAIMRHPRSVAGSVLLFRLRTLFFATVCFTFLCSQLTVMCRQGTTDLDVFTLTLSVADTTAIWLVRLLHIAMCERDFHRLALQVGQDFAEFLSPEDVPVLLSQGRSVRRFTLTYISLGVLVTTYWLFTPVSPEGLPFFLALPYDASTPLGFAVSWVYCTVGTVHVVVMTMVLDSFNVSLINQLRMQLALLHNKIVSLPRENSSTPLDSLQTTGYWDLRYRLEKCVLHHQAIIRNSDLLEKSLKGMLLAQSLSIGAAACFQMFEVAMSADGLQQIGKFGLYLTGMLAELFIYCWFGDDLITESENLAVAAYDAVTSLQGCPLSIKKSLLLLMHRAQRPLRITAGGFFPFSRESFVAVVNVSYSFFAILRNF

>SgreOR15

VFSELLVLYHQGTEDLDLFILILGIADTIAMWVFRMAHITLHESDFHKLALQVSQDFADFVTWEDVPVMRRRCRTVRRISLTYMCFGLSNCAYFLSLPSSPDGLPYVLALPFDATKPASHTVAWLYCMIVTVHTVIMTIAINSFNISVITQLCIQLTQLNKKIVSLNKDMPEEPGQSGKKPARRDLYCRLKQCILHHQAIIKNVDLLQSCLGVLLLGQSLSVGTAACFEMFQIATSADGLQQIGKFGTHLFTILAELFIYCWFGDDLITESENL

>SgreOR16

HWDGRNTELLENCTGGILLAQCLSIGAAVSFQLFQVAVSTQSLVQAGKFSCYLTVVLVELFMYCWFGDDLITESENVTLAAYSAVTSLQGFPASVRKSLLIVMTRAQLPLRITAGGLFPFCRESFVSIVNMSYSYFAILRNFKND

>SgreOR17

MEPAADLAEELQLLQWLMHWAGTMRHPRAGPWAGRAYYLLNAIALAAIVLFLCGQGTAILREGTRDLDRFMLMVSTFNSVTIWFLRMCHIAIHEHQFHFLALQMDRDFREFLNLRDIPPLRKRCRGHRRFLLFYLTLGVIVSVVWSVLPMATFGVGPEAIPNAMALPYDVSHLHTFIPTWIFSAFIVVHVTIMTITTDTFNVSLIAQLRFQLFVLNRNLITLNADAETIKSPLTKGNYITNKSSNESLEHRNIHHRLKQNVLHHQLIIRNTELMEKCIGGILLAQCLSIGAAVSLQLFQVAVNSRSLTQAGKCSWYLTLMLAEVFVYCWFGDELITESENVTMSAYTAATSLQGFPADVRKSLLLVMTRAQRPLRITAGGLFPFCRESFVSIVNMSYSYFAILRNFKDD

>SgreOR18

DFGEFMAPRDVPLLRRHSRNQRRFVLAYLSLGMCDVSTWLTSPMRDTGLPLDVILPFDTAHPIGWAAGWLYCAFITVHAVVMNMMADAFNVSLMAQLRMQLIILRKNINDLASNGESAVSSKSRTTFKGAERPWKLYTRMYKPYPLRKFNNHNQKMNSIMLVAEAKKHQQSSEFSNSNDIHYRLKRIIIQHQTIIRNTEFLQKCLGGMLLGQSLALGVAICILLFQVGLSAQSARETGKFGAYLCAMFTELSVYCWFGDKLMSESEKVAFAAYDVVTSMQECPVSIKRTLLLIMLRAQRPLCITAAGFFPFSRESFVSIINVSYSFFAILRNFKEE

>SgreOR19

MEPLASAASAAEDAANLDYLLWFLHWSATLRHPRTRAWSARIFYLRYIVCVMILVYSCSVHVVLMWQMGFDDFDYLTLALCLLDTYITWIARLGHIAKHERDFHRLARQVRDDFGEFMAPRDVPVLLSRSRTLRRFVLTYLSLGVCDVTMWLTFPMRGAGLPLDVVLPFDTAHPVGWAAGWLYCAFITVHAIVMNMMVDAFNVSLMAQLSMQLVILGRKINDLANDAESMASSPPQTAFEGDEQPRTFCSRMYNRYSSKKINDHNQRMNSLALDVETKEIRQSEELSSGSDVHCRLKKIILHHQTIIRNVEFLQQCLGDMLLGQSLGLGIAICILLLQVGLSAQNVRETGKFGAYLCAMFTELSVYCWFGDQLMSESENVAFCAYDAVTSMQELPTSIKRSLLLVMLRAQRPLCITAAGLFPFSRESFVSILNVSYSFFAILRNF

>SgreOR20

SCSWMIRLRHIASWEVEFQRLATEVRFDFDEFLSADDLWLLRLRSRSQRRFVSAYLCSGVFTSAVFAAIPVSAEGFPFIMALPYDANQPLAFATTWLYTAYIVFFVVLGNRAADSFNISLIVQLRNQLDLLSRKLRNLNGSISHTKSSIKKTKRSEDVSEDVYYQLRKSILHHQSIIRNVQLLEQCLGRMLLGQCLSNGTSFCLLLFQAAKRAKGVQELGKTCSYLLNTLFDLFVYCWFGDDLIFESEKVASSAYDAVPSLQECPTGIKRSLLLLMLRAQRPLRISAGGFFALCRESFVSVLNVSYSFFTILRNFKED

>SgreOR21

VALLLFICSQAVLLWRDGAADLERFTLSLSVLDTTSVFMIRLRHIAALEADFHRLAMQERRDFGEFLSADDLRLVHLRSRSIRRVVSAYLLSGVFTSIVYAATPISAEGLPFILALPYDATRPLAFAATWLYSAYIVFSVDIGTMAADSFNITLMVQLRNQLDLLSRNLRSLNDIVSPTKPTSFQTHSIKNIRSSEDLSHDIHYRLRKSVLHHQAIIRNVELLEQCLGRMLLGQSLSIGVCFCFQLFQTAKRSNGVQEVGKTSSYLVIVLSLLFVYCWYGDDLISESEKVALSAYDAVTSLQECPTSTKRSLLLLMLRAQRPLRITAGGFFSLSRESFVSVLNVSYSFFTILRNFNDE

>SgreOR22

MKSEQEDNRNVAMDFLVTLMHAWALVSRSTSGPWLLVHHFYKAVVFLCMGSLLVSELIAIGIGGDDMLTLSLNTCVMLSNAVAIYKLVLSTLWPGRYRSLVEHVVRDLTDHLSHDPSGRRRLGADSRTAYTFTMGFVVCGHVTVASWSLLPLLLKPVEKLRLPLVAWTPFDSSCGIGFLVTYVYQFTCTLFMAWTSGATDLICVNVVMQLCSHLEILCCHLQRVGGQCCSDVGSSCDHGYLGKSAEKPAPDKDDLRDQLRRCIRYHQEIISVAREMDSLLWSIVLSQCLCGMTVLCLLLFQMALYTLTIETIAKYLSYMASILLQIFCYCWFGDNLSSKSTDVARVAYSCAWTRGSAGFGRSLCILMARAQKPLIVSGGSFYVLSREAFIRILNASYSYFAVLY

>SgreOR23

QLVVVGVVAVVVPCSDLFTVACVQRLGTLLDILERRVERLGVAGSPQSPGGSRHSPADPLHRALRDCVSLHQRIISQAECLNQAVGGALLPQVVASAAGICFLLFQVAKKTSFHVVETGKLLGYLTFMLSQLLFYCWFGDDMLSKSERVALAAYRCAWPGAPTHFQRSLLLVSMRARKPLTLRAGKFFVFSRQAFVQVMNVSYSYFTVLRSLSEA

>SgreOR24

HFADTLVHDINRGLQEFGEAYRQEKEAVYAACARQSVLLTCVHVGMGAVVNACWLTMPATKVAACQTIECRIKEGLPAPVWFPFPFTEPPVYEAVYVGVSLALFYGYILTTMLDGFFFTLIIYTAGQLRVLNLMATRLCDPEPGSKVCLDEQLQWRIAEVVQCHTHIDRKTDSEWLFKYGSYLSAISEQLLLYCWLGHDILTESERLQMSAYSSDWTAAPRRLRAQLRVFLCRAHRPLRLTASKFYTISRDTFLLLMNASFSYYAVLRQLNGD

>SgreOR25

AESMCSGQPAHSRRDVAAPADTSEECVRRRLAQCVRYHTDVDRCVQQLSTLLGPILLGQVLADVVTISATAFVTTTGKTDSGWVFKYGSYLAAIAEQLLLYCWFGNDVLTESERLQLSAYSSQWVAASPRFRKELLLFLCRAHRPLRLTASKFYTISRETFLMLMNASFSYYAVLRQLNSD

>SgreOR26

RRRASSQLYGTYSGCVVVILLLFVASLLFAMVHYWGHMLGVTMNACLMFTYVMNSIKIVAFLRMRPAIDQFIDELDSCMQEYGQELRSQKAAVFRWTALKSRIVSVARLSVTAMGCVYWAVMPAVRASACGHTVECRASVGLPAHVWYPFSYTVSPVYEVIYAAVAAGLMYGALLSSIMDGFLVSLFIYMAGHLQLLNLMLRSISGDHEEDDEDGDHHGKQRRDVAAAGPFLQEQLMRWRLAQCVTYHCRIDMGVQRLSTLFGPILLGQFMMDIIAISATAFVAIAKNADSTWLVKYTSYLSAVIQQLLFYCWFGTDVLTESERLQMSAYSCNWMDASPRFRMELRIFLCRTHRPMRLTASKFYTISKETFLMLMNASVSYFAVLREINAK

>SgreOR27

TLVAGGLLCGALISCIVDAFFVSLIIYQAAHLQLLNLMLVNVCGAQGGAEPGLADSRGQKRREAAQSADVEGQMRRRLAECVCYHCDIDSCVQHLSSLLGPIILGQFLMDMVTISATAFVAIANNADSNWLVKYMSYLSSVIQQLLLYCWFGTDIITQSERLQMSAYSSDWVGATPRFGRELRIFLCRAHRPLRLTASKFYTISRDTFLLLMNASLSYFAVLREISNK

>SgreOR28

MGLFYGCIIFTSQDGFFWSLIIYVGAHLRFLNFMVTNMSSGESDTKSPGGSPQLEEKMRRRLKECICYHNDIDRCVQRLSSLLGPVMLGQFLTDIVTISASAFVATMLKADSGWLLKYGSYLSGTIEHMFLFCWFGNDILTESERLQLSAYSSAWADASPRFRKELRIFLCRSQRPLILTASKFCAISRQTFLRLMNASYSYFALLNQLSSE

>SgreOR29

AERMAKAKDLPDPVVDLRWMSRALAPVGMWGPSGGSRLYDAYSLWTIVQLVLTAAGQVAGLQGHWDDLQTVFTTLCFAFTVTCTIIKGTIFVLQRESMDALKRHIERNAVEFCSHLPEERRSLLVRARNLSRLIVCSFQSVGGVTLVSFITGPIVQNGRDRELLASGLNNATLDRRLGHNYPMLMWWFGGLPVTSPGYEAAYIIMCYWLVLMYICTNVPDAYYVGLINYITAQLRLLHIALPKMATLEPDAVLQEKLAHSYGLTIKADVPADAGAQDVIQDRVYARLVECIRFHQEIMKCVDEMESVLSLTVLIQFFTSTLVICLTAITVISTETAYLPTYAAYLATMFYQLFIYCWYGGEVYLESESLQFSAYSCNWPETDARFRKTLRICLARMQRPISLTACKFYKLSRETFLLLLNGSYSYFTLLLQMNQKND

>SgreOR30

AFSPLRAAGRAAILREARRRALLMFCVFFGIGAVALSLFYAGPALQNLQDAEVLASAAGNGTLERHLGRNLPMLIGWYGGQPVRTPYYQLSYALICYWFMLIYLSTSTLDAFYVTLIIYLSSQLKMLNAALADTVQLPGLSEAATVGDQRPAASAARQGDYRRLVQSVLFHQEIIKSVEEMESLLSPSVLAQFATSTLVICFTAFAVMTSTKRQEMPAYATYLATMFYELFMYCWYGNELLEQSDALQLSAYSCAWPGAGGRFQRSLCILMARVQRPLCLTAAKLYKISRETFLVLLKGSYTYFALLHQMNDPQHEA

>SgreOR31

NSGTALRSGEMADYVHRKPVVEIGLSKAALVAIGLWAPSRPSRLYWVYTRWSLLQLVVNIVGQVAGLQGHWDNLPVVSTSLCFVLTITCTFIKAVIFVIQRPRVDALMAHLERNLAMFCSFMPRVKDAILRSCRLRVLFMFTSFMGIGGITLLSFYTGPIVQNFKDAKLLADEASNVTMRPHMGRNLPMLMWWYNGQPEQTPYYQLTYVLMCYWLMLIYLACDVQDAFYVTLIIYLSAQLTMLNAALTNALHPGDGEDAHTSSERRQLPSRGWRGDAESVVPTKRSGRDRLVECIRFHQEIIKSAAEMESILSASVLMQFMTSTLVICFTAFTVITAERAYLHTYITYLATMFYQLFLYCWYGGELLLESEKLQLSAYSCAWPDADPSLKRSLRIMMARLQIPVKLTACKLYGLTRETFLLLMNGSYSYFTLLHRMNQAPA

>SgreOR32

LSKIVLLQFTGATVIICVTLFQSSKNTENIAALLLLQAYLGVVIYEIFMYCWYADDILYQNCRLAKSAYYCGWPGAPPDLQRALVFIICRTHRPLGLTAGKFYYVSRETFVRLMSASYSYYALLNQVNGN

>SgreOR33

GMWRPPWIQPKWYLLYRAWVLFTLFSFLVAQIQALWHFWGNIDKITHDTCLMISIILSLIKFFSFVLRQEEFFRMVRRIDGISAEQKKTGDSETISILEASYRSARAVTLYMTFLGGSMPGVWATIPSIMRKLGVFPPERELPATAWYSSRDTQTPYYEILSTLQYFSMQYSFFTAVGPDLLFVSMIIHAAGQLEVLNARLRRVGKMNGKRCTSSSKQQKTEEVLSLEVSSGEVMWKDLCSCIEHHRDVIELIKGVERLVSKTVLLQFMGATVIICVTLYQSSTNTENIAALLMLQGYLGLIMYEVFMYCWYAEDILYQNSRLADSAYSCGWPGAPLKLQRALVIVICRTQKPLGLTAGKFYYVSRETFVRLMSASYSYYALLRQVNDK

>SgreOR34

GCVLSTCWSLYACWALCTQLSFLVAQARALFHFWGDVGKVTHDVCLMVTVVLGLIKSYVFCRRKGDFFRIVNKIDSARSEQSKSADPEVTSILSASYKSARNVTLYMTLLGGSSPAVWAVTPTVMRRLHVGPPERELPATAWYSSRDTESPLYELLCVLQLFSMQYSFFAAVCLDLFFVSIIIHIAAQLQVLGVRLRRIGELYDKRSNSSRNSPTIRRHAEVGSSEKESQWMDLCTCVRDHHAIIELVKELEGLLNIIILFQFLGATVVICVTLFQSSTNTGNVMTLLKLQAYLMVIIYEIFIYCWYADDILYQSSQLAVGAYSCCWLDSTPRLRRALVLVLCRAQRPLGLTAGKFYHISRATFVRLISASYSYYALLNQMNDK

>SgreOR35

GGGGRQRVVHSATTAASSFTLQKFEITKMKDGHIPWSETALWMNARVLALGGSWRPPEVRGFTLYRIWVLFTQFSFLIGQLQGLYYFWGDANRIIQDVCLLVTTILGLFKFFVFVIKQEDVFKIVQTIDDRRREQGKLENPRIAAILDASYSSAKTITVCMAGVGGTAPAVWAIMPLVMRSLGVGPPDRELPAMAWYTSRDTVSPVYELLYILQYFSMQYSYFAAMCLDLFFACLIIHVAAQLEVLNVRLSQIREDLYRKDRTSEPLGDTKDEDVEEDSAWRELSECVEHHKDAIKLVDDLETLVNPIILSQFMGATIIICVTLFLITTNKQHFVALVRLQAYLAVVVYEIFMYCWFG

>SgreOR36

LNVRITQIREDIYRNGHVSESPAGTRDEGTEEDSAWRELCECVEHHKDVIKLVGDLEGLANIVILSQFMGATIIICVTLFVITTSKQHFAALVKLDGYLVVVVYEIFMYCWFGDDIMYQNSRLVNSVYTCGWPGAPQKLQKALIIILLRAQRPLGVTAGKFYHVSRRTFVSLLKASYSYYALLNQMNK

>SgreOR37

PVVCWYGSWDTVTPYYEVLYVVQFVTIQGGYLVVMGSDLFFISLMIHAAAQLRILNMKLEKIVKNEDEWNGKGIEALTFISGYRISGLKGIEKCEGESKLTHYVTDQTSSYSELRVWVEQHKDVIKLVQRLEQLLNMIILFQFLGGTIIICVTLYQSSAKTGEVTTMCKLQLYLSTMLSEIFMYCWYADGIVQQSARLARSAYSCGWPGAPQSLRRAVLIVMCRTQRPLSLTAGKFYTISRTTFVRLVNASYSYYALLRQMNDH

>SgreOR38

RSLPTIAWYPYDETETPFFELTYVLQGISTFYCCITNVGLNVFLVSLIIYVSDELKNLNDSISSINYSTSHNCNCSYGKQHGLLYRSSKIVADDGERDIDTYKSHSKNLRQRTQFCCVVKAQQYLWMCLQYHQELIKTVKKLETTMTGVVFIEFVAGIIVTCLTLFHAAVNAGNMALFLKFVMYLLYMTVGMFIYCWYGQDLMEKSEDLKWAAYSCNWQGAPRSFTDLLKIVMLWAQQPLILSAGKYYRISLKTFVTLLNASYSYYAVLRQINDTEK

>SgreOR39

MSVEPDQRLTSLPGPHSVLDVNIGVLKVAGLWPTRPYGLFAIYTAWIYLTQWAVFALDSMSLYYYWGNLNMITAVFCNLTSITAGIIKMTHFFVYKPKYYMLVNKLDALVASQQRITDPNVNSKSILVQTSKLNKYSTYIIVTYGNLVGVPWIVLPFVMDTGETERTLPVVEWYGIRQEKSPLFEIGYVLQCLTIMYWFFASWGLDLFFGALMIHLAGQLKILNNRVANVGRKENAALNGSSAEESERKIMDFNRDVVSRKTPVQQQEVTLYSELRNCIIDHQEMISFMNDLEQTVNFVVLVQFSAGTLVICVNLFQAALNVQDFSSVLKVCMYMFELILQLFIYCWCAHDVMVESERLSTSAYFSEWTGASRRFNTALHILMARAQMPLTVSAGRIYTINRSTFVSLINASYSYYAILRQMSDR

>SgreOR40

KGDRNYTRNSTASVLSSSFGRNGVLEPASTMNSKIHSEAEMYNSFVRCIQQHQEIISFAEELEKLMNDVLLVDFLCCMVVTCSTLYVSTSANENFGDLLSHFGYLVAMTYPLLLYCLSAHEIKDQSQQVSMSAYCSPWHQATKGYRRALCVVMCRAQRPLTLTAGKFYVVSRATFLTLMNASYSYYNILREINDSKRSD

>SgreOR41

DICIQSPGGESAGMITIGSSAKPIVEQPSKSWDDVLGRNVKILIFGGVWRPVSRSGWRPYVFPLYFASVCGSLLTIIGLDIVRIWLLWGDMTEITYAVVTAVCCFNGVYKMVHCFKHGGTYSRLVRGLNDLVALQRPYCERDDTLMEVFQEVCRKTKRLTIGCLTYMSVLGQMWCVLPLVSPVPPDSREDPMPLVSLPGLHKGNTGWYSFIYLLECHTVFYWNFSSFGMDMFFASIMSHVTGQLNILNMRLTQLRQEESSEDQVRPSTCNGFNLNTSNQDQEQNVNDSASMYIELCECVKHHQAIIRYLEFLDSVMSPVSLTQFLCSVVAVCVTLYQITFNPQDSAVVKCALYLPIPALQIFIYCWCGHDTMEALINASYTYYTLLRQMRNL

>SgreOR42

MAVLETFARQRLGATREPWDNVLRRNVKVLRFGGVWRPASKSGWRHCAFPLYFASVCGSLLNIITLDIVRSWLLWGDMTEVTFALVSAMTNVNGVFKMVHCFRHCETYSRLVSELNGLVELQRPYCEGNGDLMAAFRKACRRAARLTIGCLTYMNVLGQMWCVVPLVSHVPPGSRESPLPLVSLPGLHRENRGWYLFAYLVECHAVFYWNFASLGMDMFFASIMIQVTGQLNILNIRLTQLRQEGSTEDQAQLPRSTGFNLSRNDQHQQRSANDFTRMYSELCECVKHHQAILKYLEFLERVMSPVVLTQFLCSVVAVCVTLYQITFNPEGSGVIKCAMFLPIPALQIFVYCWCGHDIMEAGLSVSLAAYSCAWVGVGQRVTSALRIVMCRAQRPLQLTAGKVYPVNRDTFLSLINASYTFYTLLRQMRNR

>SgreOR43

MVAIGSRTKPILVQGSKYSNKGQEDGASPSENVLWRNIRVLRLAGVWRPEGRCLARLYPLYFGTVCTSMLHIGALAILRSYTIWGNMTEVTFALVSGLTCFNGAVKMIHHYSHSESYYRLVDELNILIDRQRPLCEGDAELAAALHAAYEKAKRLTFGVLGYMFVLGQMWATVPLFMQFPPDDPSSPLPLVTITRVHKVHNHTLYSLAYLSECHTVLYWNFSSLGMDVFFGSVMIHVTGQLNILNIRLSRLSDGGAAEDLAHSSNFAKGSQQQKWNTHDSVSMYDEVRNCVKDHQEILRYLDFLENLMNPVPLAQFLLCVGGICLTLYQITFNPDDGGVIECILFLPIPALQIFIYCWAGHGIMEESEYVSFAVYSCRWPGADRRVTNLLRIVMSRAQKASFLTAGKVHPINRDTFLSLLNASYSFYTLLRQMKNLEEGNEASP

>SgreOR44

HLACLSCCSHISQEQSTPMAELKSGRGPTTGVCADDVLRHNVKLLTLGGAWPPSERSGLRRLFPLYTATIYFCQSATIAMGIWLTYDLWGDVDEIMLTYVNTFTLLGGYIKLIYFSRDVRGYRELVSILRDVAREQWPYCENDAKLMAIFTGAYRKGLWLTFGPLVYLNILGPTWFFMPLILRAFGSEERLLPFVNLRESVTNIFPLYVAIYVVQVYCMFFWNIISVGLDMFFVTSMVHVAAQLKILNERLSNLGEDPPHDDYGAVLDRQIMRGFVNPQKWPFSRDGRRRDMYEELRNCIKTHQHILSSLKTLQRVMSPVAMTQFLCSASGACITLFQATFNPEGNSTLKCLIFLPLPAFQIFIYCWAGHEIVYQEELLSVSGYGSAWVGASRRISVVLHILMCNAQKPLRLTAGKFYPVNRDTFVTLINASYTFYTLMRQTRDQGSTVQT

>SgreOR45

MAQTNRKMPPANVLATNISILRWSGLWPPERQTGWARLFTAYTAVAFLSQVVAIDMTLYHIYNSEGDIYEITLTMMVTMTLVGGVLKMLHFFANADTYSRLVRDLQEVIDLQRGNCERDDAVSAIFCSYHRQAILFTCGSLGYLNVLAPTWFLMPVITGVATDPNDRKLPFSQLKGLRSDDLIGYSAAYFVQCHAIFYWNFISVGLDVFFATAMLHAAAQLKILSHRLSRLGKQTTRYEEQPPYWNALQGDAAPVRQIITYEEENDLYLQLRNCIKNHQEILRLVLFLETVMGPVAFIQFLCSVVAACVALFQATFNAEGNGVLKCTMYLPTPAFQIFIYCWCGHEIMEEGLYVSRAAYSSGWVGASQRFTRALRIVMCRAQRPLLLTAGKLYPVNRLTFVSLINASYTFYALLRQMRDR

>SgreOR46

RKCRPIHISGARTGTVNGGGKEGAQKCNSLRFWPFFVMDSDTQGSKSLTWQYTADSILKYDVRFLSVCGLWPLQGSRLFRIFTVTIITLCLGHICEASINLCTLRGELEDYTLALSNVSVIIVGVLKVAIFLRDEGSLCRLVRWLDALVKRQTEYVRGQPCREAIFREARQRASRISKGLDAYNVSLLFVWILAPLLASPGDKRLPLQQLPMANTTTFPLYELSYALQGISLTFIALINVHMDCFFTVVMIHIAAQLKIMASRIADLHLTRYGGENSLTESKLGKNSLVMEDLYKKLCLCIHTHQDITRFIVHLEKVMNPIAMMQLALGVFNGCMLIFPAAYSAESESLLKILVSAPVVSTQLLLYCLGAHSVREQGELVSLAAYSCGWPD

>SgreOR47

MDWDPQRSQPLTWQYTASSVLKYNVRFLNVSGLWPLQHLQLFRIFIATTVTLCLGHIAEAGINLCTLRGELEDYTLALSNVSVIIVGMLKVAIFLRNEGSFCFLVRWLDALVERQTEYVRDQPSREAIFRGARQRASRISKGLDAYNLSLLTLWTVAPLIASTGDKRLPFQQLPMANTTTFPLYELSYALQGTSLIIICLINVHLDCFFTAVMILIGAQLKLVGSRIADLHPRNVAVKNDSMIDDTYKDLCLCIQTHQDITRFIKHLERVMNPIAMLQLALGVFNGCMLIFPAAYNAESGSLIKVLVTAPAVSMQLLVYCLGAHSVREQGKLVSVAAYSCGWPDTDIKFQRALLLVMARAQKPLSLTAGGVYPIQRATFLSLLNAGYSYYAVLQNFTGR

>SgreOR48

VLSRPHHLSCRWPYGTVELKPSRVPVFGQSLPMERDSQAVEPLTGQYAAGSVLRYNLRWLHAFGVWPLSGSPLFRIFTATISALSLAHIAEGVVHLCTLRGDLEDYTLALSCLSIMVVGSTKATFFLCNEAGYCRLVRWLDALVNSQREYVRSRSTREAIFEEVQRRGIRISKAFNAYNISLLSLWFVAPLMSPEKRLPFQQLPVTNTTILPTYAYELSYALQVISMFFVGLIHVQMDSFFIVVMFHISAQLKILSERIADLQLRRGDKNELFESNENKRLHKSMSAIETHKQLCLCIRTHQDITRFIGYLESVMNPIAMMQ

>SgreOR49

MGCDLEETEPLTWQYTAHSVLKYDLRILHLLCLWPLPGSLLFRTLTAFFTALCLGHIAEAGVNLCTLSGDMEAYTLALSAVSVVIVGVLKVTFFLRHERKYCRLVRWLDALVAAEREAVHGRPLLEAIFPAVQKRAVRIARGLLLYNCFLLAIWLTVPLAAPPEARRLPLQQLPFTDENAYPLYELSYALQALSIIFIGLINVHMDSFFTVAMIYTAALLRSLALRLADLQAHDTLSRAKGNGRGQKTATADEMYGELCFCIRTHQEITRFVQHLESVMNPIAMMQLALGVFDACMLIFPAAYSPERGALLKCLVSGPTVAMQILLYCLGAHSVREQGESVSLAAYNCGWPDASARFTRAVQLIINRAQQPLALTAGGVYPIQRATFLSLLNAGYSYYAVLQNFNGR

>SgreOR50

MGWDSEGSQPLTWQYTGTSVLKCDLRMLHLIGVWPLSGSRLYRCLVTVVIALCFGHFVEAVIHLCTLDGYLEDFTLALSNISVVIVGVLKVTFFLRHERAYCRLVRWLDDLVTGQREYTRGRPQLEEVSAGAQRLARRITKAFTVYDAIVVLAWTLVPLTAPPEAKRLPFEQLPLTEESPHSLYALSYAIQGASTFWIAVISVQMDCFFTAVMIHAASQLRILSLRISDLQLGNGHLQLEDNGSLDGMYEELRFCIHTHQDIARFVKHLESVMNPIAMMQLAVGVFNGCMLIFPATYSSENEALLKCVAAAPTISAQLLLYCLGAHSV

>SgreOR51

RFSPEAKRLPFQQLPLTEDSPFSVYALSYALQGVSMLWIALISVQMDCFFTAAMIHATSQLRILSSIIADLQLGNDDQKLQEKISLDSMYKELRLCIQAHQEITRFVDHLESVMNPIAMMQLAVGVFNGCMLIFPATYSSENDALLKCVIAAPTISAQLLLYCL

>SgreOR52

RGVRFARNFLTDCWNNGCNCAFPAILHFLTPMMSWHSQQDEPLTWQYTAASILKFDVRILHAIGVWALPVTRVYRAYTGAILVLVVAYSVEAVIHIRLARFNLDDVTLAVSTYAVIVSSACKLLSFLHHEPGYWRLVRWLDALVAEQKQFCEERPRLQAIFDRAQKRATRFTHALRIYNTSLILAWVFIPLMAPPGQRPLPFQQLPLSETDDFPLYIASYLLQSVCMLYMSLVSGCLDGFFTAVMIHTAAQFRILAWRIAELRQNNEERKQQARSDVYEKSRKAAATDNVYEELRLCIRTHQEITSFAAHLESVMKSIAALQLVTGVTNGCLMIFPTAASSESGALLKCVACVPTISAQVLIYCLGAHSVQEQLLNAGYSYYALLKNFNSR

>SgreOR53

VAHIAVAAVGIWYRPGDLAEVTIGLANVFVIFTALSKSLLFVSRRPLFYALARRVDLMTVEQQAFCTSDPTLQHAVSIARRRAGRLSVFFHWYVLVADILWSLIPLVQASKEKRWPFQQMPLGTWERSPFYELSYLLQCASTVFFSLISVDVDCFFVAVMIHITVQLRILTSRFATVGTGMNVTVNELHCQASRLKDAIHEQLRACVETHQNLLRLVSFLNVVMSPVAMLQLAVGAVSSCMVLFPATYSTDNAAAMKCWAALPVLGIQLYLYCSTAHHLKDQGEAVSSAVYCCAWPESGCRLQRSLLLVMCRAQRPLVLTAGLMFPINRATFLSLVNATYSYYTVLKHVNNR

>SgreOR54

MPAIKRQRRLPYQQLRWLDTSSAAVYGASYALQCTATFFCSFINTHLDVFFMAVMIHVADQFRILAARFAALRLDADCKPESRVLQDGSSRLGEELYRELSLCIQNHQDLVRLVHLLDDVMSPIAMTQFVVGAVNACMVLFPATYAETVSSAAYECCWLGADRKRRRALLLVMCRAQRPLQLTAGKLYPINRPTFLSLLQVTYSYYTLLQNLNSR

>SgreOR55

ERRRTATWRRHHQLVTEDTDSNSIWPQAMETSRNDHREMESGRCGEERVVTWRGAAGSVLKHNVRFLCLLGVWPACRGSAYHAFSALVVALCGTHMAFVVLGVGKGSEDLQETTLALTNAFAVSGGATKVALLLRGRRRFYALVRRLDRLVAAQAEHRRADARRQAAFDAFHRKAVRLTFLLHSYLFLLSFVWFPMPLIAQRGERRLPFVQLSWLDTSQLAVYAATYTLQCAGTFLLAFATVSVDCFFVAIMVHVSVQLRILSSRINALGLRTNLAGSARTFESDVSGEASDIQRDMYRELRLCIQYHQDILRFVRYLDSVMSPLAMTQFISGVLLACVALFQATL

>SgreOR56

NMYNQLCQCIQTHQSLLRFVSFLDSVMNPIVMTQFTFGVMVAGVTLFQATFSPSNSTIFKCVTWLPMPSTQIFLYCWGAHDIMDQGEAVSEALYSCGWMEAPARFRRAMRIVMCCAQRPLSLTAGRVYAINRA

>SgreOR57

AETASGRSLVSAMEKQKGRSRQLKDTGSAVTEDLTWDEVNCSVLGTNIRLLYYMGLWPLGSSRAYHAYTAFSLSFSAAAILMKVVGACYNLSDIDQVTGALSTILPMSGGLANGLFLMRRRPAICRMVRSIDRLVDQQR

>SgreOR58

LLGQRISGLRLSGDGAVDLAGDRGHSTRQMMYVELCSCIRSHQKITKFLRDLEAAMSTMVLVQLSTTMVGLCMTLYQQIQIEDALTALQYALILPFYSAEIYFYCWTADVITQQGEALSLAAYSSHWVGAGTSLQRALSIVMARAQKPLLITAGHLYPVNTAAFVALMKASYSYYTLLRQLDGD

>SgreOR59

RREATAAMAGALDKTKPWSEVDGQHGNRCTLAGHILYLRVAGLWPHAQGARYLLHTVMVQLCAVAYIAVGVASIYTARGDVDGISHTLMHLLEVVSGMVKAGLFFSKRQSFYRLVQDLDLMVSEDWDRPELVSARRWARRMTVSLTAYIYTLILLWLPAPLLAGGDQKLLPVVQIEGVDWSLWPGAYAALYALQCSVLLTQVPVVIGLDCFFVAAMLHVAALLQLLGQRISGLQVISGSVADLAGDVSLKRRQVLYAELCTCIVNHQKITKYLRNLEAAMSTMVLVQLSTNMICLCMGLYQQIQQGEALSEAAYSSHWVGAGAGFQRALCIVMARAHKPLLITAGHLYPVNTAAFVALMKASYSYYTL

>SgreOR60

VGALLQLLGQRVSSLRVSSDGDSEDCASDEGRHILYAELCSCIQSHQKITKFLQDLEEAMSGMVLVQLSSTMVNLCTALYQQTKIKDVSSALQYAMLLPFHTAQSTLLLAAHTVTEQAQMISAAAYGCSWVEESERFKHAMMILINRAQKPLVLTAGHLYPIDKPAFLSLVNASYSYYALLGQMNKR

>SgreOR61

LNARLAQLHLYSGGGKLRITATRVLRKVTTEDRDDMYDELCLCIDTHQEIMRLISFLDSVMNPIVLTQFMLSVMAACLTLYQQTYSPDGNSVMKSASYLPTPGIEVFVYCWGAHSIREQGEAVSEAAYSCSWFDGSPRFKRALRMVMCRAHKPLVVTAGRLYAINRATFLSLVNASYSYFALLSRIHNR

>SgreOR62

LAVGNAIENVLGVWKNWGDLTEVTYSLLNAFTIGAGVAKACHLMLHEERYCLLVRRVDRLARSQGVYCDADAGMLAVAVGCRGTARRVTRAAFAYLTVLCLIWMFMPLVAHPGERQLPFNHIPWEPQSMPLFYELSYAVQSASSVIYVFISFAFDCFFAVVMILVTEQLMVLNLRIKQLYAPTAGDGPIKKEKQFPDKTVTDRHEVMYKELCLCIDTHEDIIRLVSFLDSVMHPIALTQFMLSVMAACVTLFLESYSPDSSSVLNSVSYLPTPGIQVYLYCWSAHNVLEEGAAVSEAAYGCSWYDGSPRFKRALRIVMCRAQKPLVVTAGKLYPISRATFVSLVNASYTYYALLSRVHNRG

>SgreOR63

LACAYIGAVLLASLANMAEAALGIAFGEASMDEITLVLPNTLTTACGVVKLGFFLRDHRRYYALVRRTERLVMSHEELAGGAAEACARDVSRRVRAFALFVVALICVQGAVWCPMPLIAYPGERKLPFGQLPSTNYTQVPVYELSYVLQCMSSFTIANVTLGFDCLFMGIMEFLGAQLDLLTLRLRSLRLDAALCTTGQKDGRGCAVQATKGHDSAHRELCLCIERHQEIIRFTRYLEETMSPVAMTQFMFSVLSVCVLLFQATYSDDFSAVFRCVAFLPLPGGQMYIYCWAAHRITEKAEALSMAAYSCQWVESSQCFKRSLAIVILRAQQQLVITAGHLYPVNRTTFVSLVNASYSYYALLGQINKR

>SgreOR64

SGGGIFFAVVFVSNLGNMTEAALGLYLGHGGLQEITLVLPNTLTVASGVFKMVFFYRDRGRYYGLVRRTDRLTMLQLAAPGEDAATIVRDAGRHSLKLSSSVYAFVSLQIIVWFPMPLIAYPGQRKLPFVQLPWNNNTEIPVYELSYALQCFSSFTIIFITLGMDCLFAVIMIHVAAQFEILITRIRNLRLDLSTSVMQQKPILSQHSRNSRTSMNTPTESKEILEFQHQITEAHDKMYSDLCNCVEVHQEIIRFVRHLETMMSPIAMTQFVFSVLVACVALYQATYSEDFSAVFRCAGFLPVPGGQVYLYCWAAHLIMEQSEAVSAAAYACSWIEASPRFKRTLRILMCRAQKPLVLTAGRLYQVHRSTFLSLVNASYSYYALLGQISKR

>SgreOR65

MKSREKGDTELPLSWSESERSVLKLNMRHLCLFGAWPLSRYRIFHVYSVINLALGIWYVNEASTYVSYIWGDMEEATLVLISTFNIGSIVVKTTLFLWYRGQYFALVRRVDDLMLVQSEACSQDPVLGHLLWRSKRTAARLTTAMLLLMVSQYVTWYPMPLIMNWGERRLPLAQHPWDNNSNYYVLSYAAQCISVSWMTQISFGIDCLFVSIMILVAAQMKILASRVASLKMQDDELSRKKHAIKCPRYKLYRDLCLCIETHQQLLRFIRELEDAMSPIVLTQFACSVLVTCVTLFPAVFSTDFTAVIRSAGFLPVPLGQPYLYCWSAHTVTEQAEAVSAAAYSCAWMEASESFKRALRILISRAQKPLVITAGHLYPINRPAFVSLVNASYTYYALLCQFNKRAKERAL

>SgreOR66

MARGVPEDIELPLSWAASGQSTLKLNIRHLWLFGAWPLSGSWFYDVYNAFCLAMGVWNAVESLLSLYFCWGDMDERTMVFISTFTNACGTAKMAFFMRNRRQYYALARSVEVLMSLQSEFCSADPALADIRRGSQKRGYRLTLGMLLLMFSQYPVWFPMPIIAQPEDRRLPFAQHGWDNNTSYYELSYVVQCVLSTFATQLSNSVDLLFVTVMILTAAEMRILTLRIVSLKSENNEVGLVKRDNVVIGQTENTCDDEMYEKLCQCIESHQKVIRFVKQLENSMSSIVMLQFYFSVLIICVTLFQATYSTDYSSVLKCACYLPVPTGQIFLYCWGAHNITEQAEAVTLAAYSCSWLGASPRFKRALRILMCRAQKPLVITAAHLYPMSKDTFVSLVNASYSFYALLGQMNRR

>SgreOR67

MAKGAEEEIDRPLTWAESGQSILKLNIRHLWLCGVWPLPGCWLYDAYAIMGLIVGAVNAVESAVSLYFYWGDMEETTLLLTSSVSNGCGTVKMAVFMRNQRQYFAMARRVEMMMSMQDEYTSEDPALDEIQQVAHKRGYRLTLFWTLLMFSQYFVWYPMPFYAHPGERRLPFAQHAWDNNTNQYALSYFLQCAASARGTQLSWSLDLLFVSVMLLVAAQLEILTRRITSLKEESHEGKAAAVGEKSVKGMAANIYDNMYDNLRLCIETHQKLLGFIRYLDDTMSSVVMIQFCVSVLLVCVALFQATYSTDSTAVLRCALYLPMPGSQVFLYCWAAHSVTEQAEAVSLAAYSCSWVGTARRIKQSLRIIISRAQKPLVLTAGHIYPIDREAFVSLVNASYSYYALLGQMSKR

>SgreOR68

HLEETTLVFIFTSTCSCGIIKMVFFVRNRQSYSLMAREVAGLIALQNGASSKDPALAAILHDSRKHAFRITMGMLLFMFSQCFIWYPIPIVAHAGERRLPFSQHGWDNNSNFYELSYTLQCVSGLYMSQISFGLDCLFASIMILVTAQLKILRRRILKLHKEVIPADGNDSFRRWDQMPADKCYDNMYERLCLCIDSHQHILRFVKHLQVTMSPIAMTQFASSVVIACMALFQSTYGEDISAALKCASYLPIPGGQVYLYCWAANSVTENGESVSTAAYSCSWVECSARFKHALRILIVRAQKPLVLTAGHLYPINRGAFLSLLNASYSYYALLGQMNKR

>SgreOR69

WLSQISYGIDCLFASVMLLVAAQLEILAGRIVALGKVANAAGCRPDESLAANTRNQTYRDLCVCVEAHQKVLRFVTHLETTMSPIAMTQFFFSVVVACMALFQATYSKDFTAVFRCVAFMPIPCGQVLLYCWAAHNVTEQLVNASYSYYAMLGQMNRRSAQA

>SgreOR70

MKDNLSWAASGNSVLKLNIRHLSLLGVWELGESRLLKFQSTFAFGLGLWGTVECVLAVYCIWGDLEQTTLVLLITFTCGSGVAKMAVFLYDRRQYNSLAHQLDKLLSLQSDSCSEDPNLAAISEWSRRKAARLTLGLLLFMLSQSFLWNFVPLLVYPEERRLPYVQHQWNNNSLYELSYAVQGLSAIWVSQISFGVDCLFAVVMILVAAQLEILGQRLVNLKNDVGIVGEDKSVSEKKERDSKTGETMYDDLCLCIETHQEILRFVAYLQDIMSPIAMTQFALSVVIACMALFQATFSEEFSAVLKCASFLPIPGGQVYLYCWAANNVTAQAEAVSVAAYNSSWVDASERFKRALRIIVSRSQKPLVLTAGHLYPINREAFLTLVNASYSYYALLSQMNSR

>SgreOR71

MNDNLSWTASGKSVLKLNIRHLWVLGIWELGETSLFTLQSIIAFGMGAFIIVDRAVAVYFIWGNFELTTLVLLITFTGGSGVLKMAVFVYNRRQYHTLANQLDELLSLQRGSCSEDPDLAAILVSSHRKAARLTKGLLLLMLSQTLLWISVPLVAYPEESRLPFVQHQWDNNNHFYGISYIVQCLSAVWVSQISLGVDCLFAAVMILVAAQLEILVNRLLNVRNEVDLMDKETAVFEKKELDSKSDQNMYDDLCICIKTHQNILRFVACLQDTMSPIAMTQFVMSVVIACMALFQATYSEEFSAVLKCASFLTIPVGQLYLYCWAATNVTAQAEAVSAAIYSCSWVDASERFKRALRIIISRSQKPLVLTAGHLYPIDKEAFLTLVNASYSYYALLSQMYNR

>SgreOR72

IAMSEFFFSVLVICLGLFQATFNEDFGAVFECASFLPIPCAHVFLYCWAANSVTVQAEALSVAAYSCSWMEASERFKRAMRILISRAQKPLILTAGHLYPIDKKAFVSLVNASYSYYALLSQTNKR

>SgreOR73

IWGDLDEMTLVFANTFTIASGLVKLAFCTRDRGLYSALARRLDALLSVQRAVCSEDPELASILRGSRKQAASLTVGMLLMMFSQGFVWFPIPVVAHPGERRLPFAQHNWDNNTNYYGLSYAVQCVAGVYLSQISFGMDCLFAAIMIIVAAELKILSCRIVKLEADDISKQVGESDDRLGDTTLRARQKPYSNLCQCIETHQNILRFVVRLQNTMSPIAMT

>SgreOR74

MKQDMQQIRPENMERPVAWTESGESVLRFNIRHLWFFGLWPLYESRMFLIYTAYGFSLGVWNVLEGALAAYFTWGDMEQTTLVLMATSTNCNGLVKMGFFLWDRRLYYSLVRRVDALMSLQSEFCCRESVLWNILRGSERHAFRLTLGMLLFMFSQCFIWFPMPLVSHSEEQRLPFAQHVWDNNTGNYELSYIAQCAAGLWMTQISFGMDCLFASVMILLAAQLEIVAERIMGIRVEASTPMKEKAEGYKKSSTAKHADTMYKDIRLCIESHQKILSFVAHLQDTMSPIAMTQFTSAVLVICFGLFQATYSEDFSAVLKCASFLPIPCGQVYLYCWAANNVTMQAEAVMMAAYSCSWVEASGRFKRVLRILISRAQKPLVLTAGHLYPIDREAFLSLVNASYSYYALLSQMNNR

>SgreOR75

VRYLNGVMSPIAMTQFVFSVLVGCLVLFQATYSTDITTVIKCISFLPIPGGQVYLYCWAAHLIIDQADAVSTAAYCCSWVDADPRFKRAMRILISRGQKPLLLTAGYIYPINRGAFLSLVNASYSYYALLGQMNKRSAS

>SgreOR76

QRAANFVMSKVSEAARLRTEFGVQLRLLRAAGAWTPGCGGVVQAVYGCYCAFIVVSLVVFVVTQVSAILHFWGDIISITNSVCVTFVYILATFKLVVLLKMRGLTDELLDNLDRCMERYGRTFGREKAFVFGECARRGRWISLMQAWMGLCLYVAWVMQPMLRVQSCSTAECRSQSGFPTVVWYPFSFTEPPAYQVVFIVVSTGLFYGNIISITVHSTFCTLMIYVAGHLRVLNHMARNLCSTQQYDEATGDHKQRNRCDAGEERVREHLRECVRYHIDIERMVQQLSKLVGPILLGELLADMITVSASAFITTALKSNSGLLLKYGWYVFVVAEQMFLVCWCGDEVLAESERLQVSVYSCDWTGGSPRLLRELRIVLSRTGRPLLLTASKFY

>SgreOR77

VNFVMSKLPGAGRPRTELRLQQKALRVAGVWAPTGGKASLAYGAYSGFVVLSVVVFLASQLSAMLHFWGDILSVTTNACVTFTYAMSTFKFIVLLKMRPLTNKLIEELDRCMECYGRTYAHQKASIFDTCARRSRRVSQMQLFVGVSIYVVWEVVPILRVQSCSSTECRIQSGFPALVWYPFSFTEAPLYQVVFAVVSTGLFYGCFISVSLDGAFCSLMIYVAGHLRFLNFMARNMCRAEKNGSRNRTPHGRHMTSATDPDEEMRGRLRECVRYHNDIERLIQRLSALVGPIMLGQFLADMITVSVSAFVTTTLSERLQVSV

>SgreOR78

TLLAFVASQVSAARHFWGDLLNVTMSTCLMFTYSMAIVKVIVFLRMRSSAEELIRELNSSMERYGRQVGGAAGVYAWCTRRSRLVSLSHVGMGAVAYCLWVAVPAAMGRRCRSADCRATTALPAVVWCPFPFTESPAYEVVYAAVSVAMFYGYILTTTLDAFFFTLIIYTAGQLRVLNLMARDLCARGSLRQMNARLADCVSYHIHIERCVQRLGALVGPIVLGQFLADIVTISAATFVATVS

>SgreOR79

LQGLGKWAFMVARRRRYTRLVDRLQHCFRLSGLADTNDSASVLATSQTAQKVAVTRLQQQLSECRRLSMWATAFWLWVCVYGVTHWCLVPLIIGDNSLPFDALYLFDASQPPLRQVVHCLQYVAGLQNILLSVFFDLFVLWLHLLLCAQLRYLADNLR

>SgreOR80

DHLLRPLHWAAVLRHPRRAAGSPLCFRLCSIAMASFAFSSTCSEVTVLFRDGTADLDVFTLTLSVADTNTIWLFRMAHIVMCERAYHRLTGQVGHDFSEFMTWDDIPVLRSQCRLVRRFTLTYIWFGIGACACYLVSPASAEGLPFIMVLPYDVTQPLSFAATWLFCTITCLHVVVMTMVLDSLNVSLISQLRVQLILLNKKIVSLAKEMSAKSVNSSETSVSPDLHYRLDKCVRHHQAIIKNADLLERSLGAMLLAQSLAIGAAICFQMFQIATSANGLQQTGKFGCYLFAMLAELFVYCYFGDDLITESENVALAAYDAVTSLQECPLSIKRSLLLLMHRAQRPLRITAGGFFPLSRE

>SgreOR81

MALHTSLSLCVRLLNMAGLWPPASARHWLTPAGLLYRLFTAVMVAPSWHILLPEAAGLVHFAGDMKAATEDMCLLCAFVTTSYKLLVILLRRTQIVRFVEALDARVTAMAAESAEARAVVRRRDLWSRRLALFMVIQSTSAAVIWSVNGLRIGLTMGSSLRTLPIISWYPYNMTVWSNYEVTFFTQMLALVTAAFCNRACDILIITLMTQISSLLEMLNLKFRDVSKIDQSP

>SgreOR82

CATTAILLSTAAANFLITVCSALKFYLDPPEETEKASQVSFLLTVSIPNTVKGISLTRQRTRLQQVVVWLLVMRRAVQDGAGRRRLYARSVNLVGKMWMVMALMGGMIWAVDPLLSQPLPLNGTSPDPALPLPVWLPFDGSAPDTYSVTFALEAIVFGWVAFVAICVDVLYITLIINFAAELHVLNDNVEITGNGADVAGHSNRKIGEVRHVGASRAGATHDGPAVIPNFPSAQSAGAKPLIPAYIPGHCDVERSDGNDTYRLLVKNIQHHQLIIKCIDEFQKATGTPVLFIVAINVLNLCSSIISLAALLEKDPRSSTVVKSLIGCLGFVIETAIYCLPGQ

>SgreOR83

WVKAVFFMLDRTRLEELMSILVDSTKRFPEDGSGIRSKYHRRAAMLSLAWQAAPVLSLPTWMMIPMLERHYVTHGNITEVYRRPMFYMLTPGNGLASPNYEIIYVVQCVAVLVAVEGSVLMDIYFVNIMIKVTSELELLNVNLVRMRLCTPAQRQIQSVGNIAVPEHSHTRKGHSKATYFLNKTNTQAGEELHDSAPIYESTNGASEEFSKLYKSLVKNVRHHQQIIVCVDELEAAMTKSITTVLAVSVFSICLQAFGFVEMFQGVVSQATVVKRMMSFLAYMTHNALFCFLGQSI

>SgreOR84

LMGLWQPRGRVARVLNRLLAGMTLSCITFLVLCASLKLYMDPPEDLEQIALCTLVATVGAGFIIRAVLFKAQGGTLRQTLRLLEDTRLQFCNGDDNELMRRRYQTLSNNIYYYCQMVAVPAVTAWAVCPLLSRIVTKTDQEQMEAQCQLPVPVWLPADIYSSPTFEFLYVVQSFSVLVATESCLSIDIFFVHMMLMVAAELEVLNDNLSAMEHINLEMLTTEGEGFISGYKRKDRRLSVITSGQSLGEQTLTEKAAHEWLHQQLVKNVLHHQAILRSVSLLQSAMNVSIFSLLFVNMANLCSSLFVASILLQKEGNVGKALNALFTIPAQVYETTIYCIYGHIMTDQSERLMYSAFSCGWVNSDARFKRSLIIFMKVTARPIVITVGKTCTLSKQMLLQVLNGTYGLLNMLYSMH

>SgreOR85

MTARPPAGTSMEHPPGDSDWLVRPGATLRRLMGLWRPRGRAASLLNRLLAGMTLSCIAFLVLCVALKLIADPPQELEQIALCSLVATIGVGFFVKAALFMAQGGTLRHTVGLLADARLHFSRGDNELTRRRYLKLSNNVHFYGQMVAVPAMIGWVVCPLLSRSVAKADQGLQEAQRQLPAPVWLPVDAYASPTYEILYVAQSFCVLVTAESCVSINLFFVHMMLMVAAELEVLNDNLSALENINLKTRRTEREGLISRYKINGRGLALLNGGQCLDEHALTENAAHEWLHRQLVKNVIHHQAIIRSVALLQSAMNVSIFILLFINMANLCSSLFVAAVLLQRDGNIGKAINALFSIPGLLYETTIYCIFAHIMSDQSERLMY

>SgreOR86

MEQPPADSDSMVVPGSTLRMVLGLWQPRGAAPSSLNRLLACVTLASITFLALCVALKLCADPPEELEQITLCGLVGSACVGYFFKAGLFVAQGGTLRHTVRLLADTRLRFCSEDSSETIRRRYQNHSNIIYYLCQMVAVPAAIGWVLCPVLSRAVTKTDKEHQEAHRQLPLPVWLPVDVYASPTYELLYVVQSFCSLVTSQSCLSVDSFFVHMMLMVAAELEVLNCNLSSMEHTKSTRTEGERFFSGHERNGKRLAVLDSGQVVDQQTLPKNAAHGWLHRQLLKNVLHHQAILRSVSLLQSAMSVSIFSLLFINMATLCSSLFVAAVLLQRDGNIGKAMNALFSIPGLLYETTIFCIYAHIMTDQSEKLMYSAFSCGWVNSDARFK

>SgreOR87

QELEQLTLCCLVVSICVGFLCKVALFVAQGSTMRESVRLLEDARTQFCNGDNNQLTRRRYQKLSDNIYYYYQVVAVPAVIGWVLCPLLSSSVTMTDQDQQEARRQLPMPVWLPFDINKSPTYEFLYVAQSICSLVTSQSCLSVDIFFVYMMLMAAAEFEVLNDNVSAMEYTNLKTARTEPEEFISKYKRSGGNLALPQNGQDLGEETYIENAKHTWLHQQLLKNVLHHQKILRSVSLLQSGMNVSIFCLLFINMANLCSSLFVAAVLLQRDGSVGKALNALSTIPVQLYETTIYCIYAHILTDQSDRLMYSAF

>SgreOR88

MSRRINRCCTLPAITQFEVSASEEGLRLSDSAVSLLGPGATLRRLMGLWQPEDGAVLSVSSVLASITLGSIAFPVVCSILKLCVDPPQPLEEVTLCGFVACMDFGFLIKAALFIKQGATMHQLVRLLWETRGRYSNGLNNEKSRARYEKLVGRLYVYMQVVVVPALLCWVSSPLLSQVFLTSREDAPDGSRQLPVHVWLPADVNRSPTYEILYVAQSFSLMVLSQATVCMDVFFVHLMLMVAAELEVLNDNISAMEQGQLQRGRSRYDGTTITDDRNIDEHEFIMSDHLQDVALTSSLGAEDKHMYAELVKNVHHHQQVLRSVSLLQKAMDASIFILLFINMANLCGTVFVAAVLLQRDGNITKALKELMLIPCVLYETGMYCLCGHMIINQSEKLVSSAFSCGWPDCDRRFKHSLLIFMVAAMRPLEITV

>SgreOR89

QDYSGRLRAALTLTAVALMPAFVALKLTMDPPDELEELAMCGFVALVAGGMLVKAALFVHGGRALRQLVQLLYWTRGRYGSGGSGDATRRRYRKLSDRVYFYLQAASVPAISGWVCSPLLSRIVLQGAPESGDARRQLPTPAWLPLDIQASPTYELLYAVLAFCLLIASETSVGIDSFFIHLMLMISAEIEVLSQNVSAMQRTLTETTQHKDEDWCSNVEINNETQKFSRSQHSLNTTLITEDVSDEEIYSRLVNNVRHHQTIFRLVALLQSSMTVSIFVLLLVNMANLCSSVFVTAVLLQRDKSVTKALKSLVAIPPVLYQTGLYCIFGNIITDQSEK

>SgreOR90

VLLASVTLASIAVVPAGAALRLCADFPEELEELVLCSYMFIICSGSVIKVALLIGEGGTVRELVRLLSDTRRQYDSGGNSDCIRNRYTRIVDRLHRYFQVMTVPALMCWVASPLLSNGTLTADQQDQRQLPLPLWLPADTHTSPTYELLFIIQATCLTITAEATLCLDVFFVRLMMFVAAEIEVLDQSISTMHNFYLKDTGPRDYVYGVGSERSVMAPGVGHSTPPVDESSDTASDEMFSKLVNNVLHHQAILRSASLLQTAMNVSTFILLFFNMANLCSIMFVTSGLLQRDGNLTKAMKALLTIPPVLFQTGMYCIFGQITTDQSEKLPDSAFGCGWINCDAHFKRSLIILMMMVRRPVEITVGKTCQLSKQMLLQVLNGTYVLLNMLFQVRSTE

>SgreOR91

MGKPSNDPARLLGSEASVLRLLGLWCPQDHGGHIVPTMLAAVTLASVCFLPAGVVLKLCGDFPEEIEEMAHCCYIFIVCFGSIVKAVLFIVEGGTLREMVQLMYSMRTQYGADEGSENIRSCYQDNVDRMYRYFQVMALLPTLYWICSPLLFGAVTSAALDARDNQRQLPLPFWLPSGVDSSPTYQLLYVIQAFSLTVTVESAICLDVFFIRLMMMVAAELQVLNENISAIECDQANRREDQECDSLIPRGDCAPQFTEKYPENFSDDEIFSRLLKNILHHQTILRCIWLLQTAMNVSIFILLFVNMANLCFNMFVTAGLLQDGRNVTKAVTALSTVPGLLLQTAMYCLFGQITTDQ

>SgreOR92

FSALKLIMDTPSELEEITLCCFVMSIAPGFLTKAVLFIYQGDTLKHLLKLMSGIRRLYRNNKRSDDIRHSYQILSGRVYLYMQVAVVPAYAGWVSIPLLARFFFNSDDDSIESNHQFPVPLWFPGNIYVAPTYEILYGIQSFCILVTAQSAICIDVFFFHVMLMVAAELQVLNENISVMQKLNVKIQGYEDYRFKFYGNNYELTFPRNALPISPIYSRSAGASDNKMCHHLVKNIRHHQEIIRCFHVLQSVMNVSIFTLLFVNMADLCSCMFVTAVLLQRDGNVTKALKPMLTIPPLLYETGIYCIFGQIITDQSEKLMDSAISSGWVDCDPRFKHDLLFFLMAAKKPLEITV

>SgreOR93

MELVKREEMQTLLGPGATVRRLMGLWWPRRGRGRARSAVCAAVSLVSLAWLPTFSGLKLIIDPPPELEEIAMCYLLIFACTGFLTKAAFFIYKGNTMKELLQLLSDTRRFYCDGEISEVIRSSYLQQSRRVYRYMQGAICLAMVFWVSTPILVRAFLSSDEDSPESYRLFPVPLWFPGNIYRSPTYEILYGVQSFSVLVAAQSTKTNAKPKTYGDQESRIIIDGKTKELAFRHNGHFTRKALSEEDSDEKMRLQLVKNIHHHQVILRSVLLLQNIMTVSIFILLFVNMVDLCTCIFVGAVLLQRDGNVTKALKPLSTVPPLLYETGLFCIFGQILTDQSEKLADSAFSCGWVDCDDRFKRDLLFLLMSARKPLEITVGKMSKLSKQMFVQVLNGSYGLLNLLYHFQSIQ

>SgreOR94

TSAAAAMALALSDLRTLQGSGASILRLLGVWWSQGRLGRLRSAASAIVTLSAYVWLTVFAALKLIIDPPEQLEDISLCCFVIVICFGYFIKVAFFIYKRDALRELLQILSDVRGSYGNGKSSHRIRSRNQKLSRRLYIFMQVALIQGLVAWLSTPLLARVFLASDQESPEPARQFPVPLWFPEYMYQSPSYELLYVVQAFCLLVGTQSATCVDAFFIHIMLVVAAELEVLNGNITAMQKDRLTVQGTEYQQRSHRTVPKRDGWTVESNDYLQSTEHVSFEGIANDMYVQLVKNIHHHHEILRSVSFLQRVMNVSIFILLFVNMADLCSCIFVSAVVLQRDGHITKALKPLMTIPPILYETGLYCIAGQMLTDQSEKLVNSAASCDWADCDARFKRSLLVFITVAARPLEITVGKTCKLS

>SgreOR95

MERADSDLQALLGPGASIRRLMGVWWSQGRYGRICSAASASVTVSSFIWLSTFTGLKLVLDPPQQLEDIALCCFVIVLCSGFLAKVAFFIYKSDALRELLQLLSDARRNYGDGKSSRSIRRRYQKLSRRLYLCMQVAVLQGLAAWVSTPVLVRVFLTSEQNSLESARQFPVPLWFPGNMYASPTYEILYILQAFSQLAATQSSLCVDAFFIHMILMVAAELEVLNENISTMQKIHLKFQGTEHQHHRTITKYEEPTIENGYLHTTTYVSSEDFSGEMYLQLVKNVQHHHVILRSVSLLQRVMTISIFILLFVNMADLCSCIFVTAVVLQRDGNITKALKPLLTIPPLLYETGMYCIFGQILTDQSEKLAYSAVNCGWADCDARFKRSLLVFMTVAGRPLEITVG

>SgreOR96

GVWWCQGRRGRFCSTAAASVTLSTFAWLSIFAALKLVKETQQQLEDISLCCFVIVICFGYFSKVAFFIYKGGALRELLQLLSDARGNYGNGKTSQSIRLRNQKLAHRLYIFMQVALLQGLAAWFSTPLLARAFLTSDEDSLESARQLPVPFWFPGNMYLSPTYEILYISQAFCQLVVTQSATCVDAFFIHIMLVVAAELEVLNENITAMQTVRIKLQGTEYQEHNLRIVAKHGEPTAYVASQCTPDEMYLQMVKNIQHHYVILRSVSLLQRIMNVSIFILLFVNMADLCSCIFVSAVVLQRDGNITKALKPLMTIPPILYETFLYCIVGQILTDQSEKLAYSAVNCGWADSDARFKRSLLVFMTVAGRPLEITVG

>SgreOR97

QVSTMVASVGWLGAPLVSRALLAAAGESGEVPRKLPLPVWLPVDVQASPTYEILYALEAYTTTLTGLVTLCIDVLFIRLMLMVTAELEVLNYNVSTMANTHEKISDRLHNESHKQNTETQDFYKSDRALQKTETVYKHGSDFQLYQELVTNVRHHMIILRTIDLLEAAMSKSIIILLFINMGALCSNLLVVGVLLQAGGGVTRPLTLTAMIPFLLYQTGMFCVFGQLVTDQSEKLMISAFSCGWNESDARFRRSLLIFMAMVNRPLEITVGKTCKLSREMF

>SgreOR98

WAAFPLVQQLFSSNGEVARLLPLPFWLPLDIYTSPTYEVIYVVQVLLIPLSTTSLFSDFVFVDLMMRIAAELEILNYSISGLQNIKNSVSTKSKNEYKSVDRGSCDSIQLQLAKNVKHHQQILRCVVLLEEAMNTGVFILFLATTIALSSNIFAATA

>SgreOR99

ATRGQPKKPSADESKKQPGMEDAGGLIGPSLAVLRLMGLWRPAAASGGACAGAAASLPGALSCVAIGLLSLSCASRLFIDTPTELTELTVCAYLFVIITANFVKACCLLLQRNTLHELVTLLAEAKKKNVIDVQHNEEIRSSYGRTSVRLYRYLQVMIVVSSIAWLFVTVPFRVFTAGSTNIDWPTPIPIWLPIDMQLSPSYELIYLGQVLCAVATAGAMLGVDTLFFHLTLMIVAELQVLNDNISVVGRVASPYEKEVFFRVKDAGEHQQNFSDVINNGQNEGGASLSNPQATITEKNYSDLVEIIQHHQIIIKMVGLLQTIMDYSVSVLLLTNVLDVCFLIFTMSE

>SgreOR100

RQQMLGGRRQTAATLEEVQDLVSPNAAMLRLLGLWRSPRSDGEGTGGALAVVRGWLCCAMVGFASVTTGAKLCVDTPEDLTALTDCGYSLLRLSAIAVKVACFILQRTTLQELVKQLADIRKIHGRRQANGRVRSFYQRRATIVYRSLQVLVMTVICGWVTAPLLQHKKADEIPESQKVERQTPLPIWLPLDLQRSPTYEIIYVVQALCGTAAVQLSMLLDTSFYKLTLLVTAELQVLNDNLAVLGRADVSAERRGSFPTAVPGQERDGVDVPRIKQTAAPVTENNDRSRKLLYFQLVENVRHHQAIIKCFQLLQSALNYSISILLLTNILTVCFAIFVASVMLQSDGGLRKAMKTITSIPTLLCETGMFCIFGQMVVDQSERLARTAYSCNWVDADARFKRALLLLVTRASQPLEFSVGKLIKLSRETFLKILNSSYTLISLLYQFQVPKD

>SgreOR101

WGLAPLLSCAFYSANSSVPCGKTPFPVWLPPALQVSPGYEVMYITQVSSHLVTTSTTTGVNLFFINLMLMIAAELDVLNENISDVRKATGKPAGGAVEEGAVASYVFTADRRTSTQNGFGRRDISDGVQSSGSVQRHTSGDIIIDPDDEMYQQLVVNIRHHQYMIESVTLLQTAMDYPVFILLFMNMSTICANILIIFVNGMNPASALKSVVASLFFISETAIYCCFGHIIVQQSERLTNSAFSCNWPDGSARFKRSVLIFMLRASQPMEITVGKTYSLSRQTLLQVLNGAYTLFNMLYRLNSTK

>SgreOR102

PAPLLGLQLGMVGVLASTAAHIHQGGGGDEDEEARQTSVAIFIVGTVIGMLVKIWSFMGQRGRMQLLLGLLLEMRRRYQRDSRGARPRAQANGVTLFYILQVNAVAAALAWSVQPLISGADRRLPLPAWLPFDATASPYYEAVYVGQALSLLLVPQISLCLNICYFALMLHLAAELAILRDNVAVVGWRRPSKEAAFQAERQAVDREFAAAPENRLLEDNVRHHQLIIRAVSELEQIMSTSVYIHLFVNMINVCSHIFVISVVLLETDEMAVVVSTASSLAVFLSGIALYCIIGHTIIDQSEQLPEAVYSSGWTGADASFRSTVSILMVRASQPLSITVGKMRVLSKPTFVQVLNGSYTLFNFLYRTQSDKERKS

>SgreOR103

YEILYAAQCVALTIASVCSFCTDIFFIHLMQVLGTEFEVLSDNFSAVQKNYEQPARASDRGELSEYQTTKETLVSSCAKIYVDCVSGEQMHRQLGKNIQHHQALLRSLSLLQTTMSVPIFFVLAINIVNLCGNLFISIVLLQRDG

>SgreOR104

CVSELEKAVSMGIFAVLSINILNLCSHVFSLVVMLETDTSMSTKIKMFLAAPVFMCQSGLYCLTGQAIIDQSDRLADSAYSCGWPDADQRFKRSLRVLMTRAEQPLCIKVGKLIELSRATFQELLKGTYQLFNLVYQVHTN

>SgreOR105

FSFSLDGHRLTELLRLLAASRQRFPDTQGRRSRHHVTATRLHRFLQVTYRMNSAYWSVAPIIRIVAAGSASRASRDLVIPLWLPLDTRASPAYETLYCLQLAFGWTVSETTVLVDGSLIALMLQVAAELAVLNDRLAATAADIGEGVPGTTRVQVSPASPSSLVVGERCGSSITTMSRSRYPPDEMYQQLVDNIKHHQMIIRCVSLLQKLLSRATSVLLFCNTVSICFQIMATAVLLQEDVEVIQTVKMLMSSTLYAYQVALFCLLGQKIINESDRLPRSAFSCGWPEADTHCWRLLVALCRRSSRALSLKVCGLYTLSRETLLQILNVSYSLFNFMYQTDKSAASRH

>SgreOR106

DTQSSPTYEILLGVQVPCCWICSETSVLLDCAMLALMLQAAAELAVLNDRLCGVGADRAPSAAPTACHKDTPPAKASYHSEGKSAGAAEAEVRSPLYSHDEMFRSLVENINHHQIIINYMHLLQMLLSRGISVLLICNTISICFHIVATVALLQEDIEPVGMTKMVLGSTLYAYQTAILCLLGQRITTQSERLPASAFSCDWPAADGRFRRLLIVFCVRSSQALVIRVCGLYSLSRETLLQVLKAAYTLFNFVYQTVGEEEPLN

>SgreOR107

AAMEESSLLGPSAVGVRHLGLWAAPGDTGPPGGSLRLALVVAAHLGLFSTSTANLVMDTPDDLEVLTINAFACMTTLGMIVKVVSYVVDRGSFNALLVRLAAARRAHADPGGRNAATRARRLAFGTALYRFTQVSTVFCTSLWSVAPLLGGSSGSRDFPVPIWLPLDMRATPTYQVVYLLQVFCIWAVVQGTVFLDTTFQILMLQLAAELEVLNDNVAAIYNREVGPSAVKRAEDIKIDASPQREKDEAIDRHGYIELHTDTAEDEMYSHLVSNIKHHQTVIGCLSQLETVMSHSTLILLSSNMVCICLHIFVTAVLLQDEIQFDKTFKMMCAFAIYTYQTGLFCLIGQTIIDQSERLVNSAFSCAWPDADARFKLSLSVFMVRSARLLEIRVGKMYTLSRETFLQILNGSYRLFNLLYQTNLEN

>SgreOR108

FQCTNCSSWTIRIVLFMQQRVRLGRLVMTLLDTRKRYAEQVPGIRSSYDQGAVIIFFAWQVLPFTSISLWALGPVTDTPKLVTAGNYSVVERREPMVLWLPLDTQQSPTYEIVFVLQVVGVVAMSEVSILLDIFFVCLMIHVTAEIAVLNKNLSSIHLLSLNGDRSQMLELVDVTALGRQRYNGGEQHTDSEETIEWSVTGSGPLRSSTFLSSQDMNDAKRRLYATLKTNIQHHQTIILCVSELEQGMSESTYVVLLVNALTICLQAFGFVELFQGGWKGPAVMKRLLACPIYMAQTALFCLFGQSLTDHSERLLDSGFSCGWPTADSRFCSALLIFMQQASQPLSIRVGKIFTLSRNSFLQIMNASYTIFNMLLNTQ

>SgreOR109

DDAKRRLYAILKMNIQHHQAIMLCVNELEQVMSMSTYIILLVNALTICLHAFGFVELFQGGWKGPAVVKRLLACPIYMAQTALFCLFGQSLTDHSERLLDSAFSCGWPGADGRFCSALLIFMQQASQPLKIRVGKIFTLSRNSFLQIMNVSYTIFNMLLNTQ

>SgreOR110

HLAILVGGLVEIVIDPPPLDVILEGIFTIACSFTWGLRMVSVLVRQSRVQQLVVDVLNMRKQFTENSAVLRKSYHRRALIVCVAWVVFPLLAVPMWFVEPALTKTLVTTSENTTIVIRKTPFVMWMPLDTQTHPNYEITYVIQIVLLVSVVQATVLVDLFFASLMINITADIAILNNNIANMRLHKENGVTKESAEGVKVSGAWEVTYKRKKSLAEKGEQHDENIATTTPYTSDPSAQLYRTLARNIQHHQVIMSIINDLESLMSESSVLMLGVNSVNICLQGLGFVDAFRPGAKRSTVLKKVLTFPAYINQTAHFCWYGQEIIDQSERLLESAFSCGWAEADQRFCSSLRIFMLQASRPLKLQIGKIFTLSRNLFLQILNTSYTIFNMMINF

>SgreOR111

ILVENIKHHQLILVYIKKLEELMRTSIFIILLSNALSICLHVLGLAVMLQEGVDLPSVLKMLITFPAYSCETGLYCMFGQAIIDQSERLVDSAFNCDWINMGPRFRRSLTIFMLQASQPLNVKVGKRVILSRHTFLRLLNGSYSLFNMLHGVQK

>SgreOR112

VMLWMGDPLMQTLMSPPAENGTRPLIFWIPVEVKNSPAYEITYAIEAFFIGSATETSILIDIILIILLVYAAGEIAVLNENIAGMRLSNTRQREKVEPNKGLKALERSIDEVSISQTAYVFSEDSSDSKRNLFVLDRNDTYWKLHSSLVRNIRHHEVILTYINDLEIVLSTSIYVLLLANALNICLHSFGIVALFQEGATSSTVFKEVISFASFLAQTGLFCFIGQLIIDQADRLQFSAFSCDWPQADETFCRSLRIFMVQAARPLSVRVGKLVELSRNSFLQAMNASYTIFNMLFNLQTAD

>SgreOR113

YVFSGYLVAPEVDADSQRDLFFLNRDHKGWKLYTSLVNNIRHHQLIIEYVKDLEVVTSTSLYLLLLANALNVCLHSFGFVALFQEGATGSTVIKEVLSFPSFLGQTALYCFFGQLVIDQADRLQFSGFSCDWPQADETFRRSLRIFMLQAARPLSVRVGKLVTLSRNSFLQALNA

>SgreOR114

MRDEVDELLAPCAAALRLLGMWGVATRRGVFVLLLNLAGTACAAVTLAFQPPRGLERLAMNAYVCAQGTLLSVKVAAFLWHRKRLRQLALQLVSCWRQFEDVGGGVRAIYRSQAARVVRYMQVMTGIPTMMWMLEPLFSGGEDSPQGRSLPLPTWLPDTLQQSPTYELLYVLQVFTIVVAIAVSVYLNIFFAVLMLSTAAELHVLNNNMMAMGKFGDNETAESYRGDEGVQRDGVTSSLLSSHRRPRGKLVSAGHSLPVAVYTHNGSHDQMYHQLIKNIRHHQVILRSVAELQKAMTHSIFVLLFLNMLNICVVIFAGTTLLQKQADQVAMYKMLFSVPIYMYETGFFCVVGQTIIDQGERLSMSAFASAWLDSPRKLHRLLLVFMLRCTRPPTITVGKTYTLSKRTFLRTQNSTKSQFK

>SgreOR115

LCWLGKGMKQPEERLLQPCAGPLRFLGLWGPPVRPLRACAVLLLNAAGTLCAALALWAAPGRSLERLAADAYVGVTGIAMTVKVSAFLWHRSRLQRLARILGAAGERSGGARGRYRRNAARVVRYMQMMVSVPAVMWTLDPVIVDDGGASAGRSLPLPIWLPGLDTSASPLYELVYALQVCTLVVAVEASLYLDVFFVVLMLAVAGELHALNDAVAAIRSPGGRPRPAPTARAATGTVDAAPGAAPSAYQAMVGVVGRHQLILASIRELELVMNHSIFLLLFLNMLNICVHTFVTAVLLQKEVQPTTMYKMVSTIPIYMYETGLFCIFGQSIIDQGER

>SgreOR116

CVGDLERVMGQSIFVILLTNVINICSQAFASAVLFRSGIDWGLVTKMLFTFVAYMFETGMFCMFGQAITDQSERLVESVGGCGWLEAEAGVRRALLVMLTQASRPLTIHVGKVAALSRSSFLQLLNMSYTVFNLLFELQSAE

>SgreOR117

MEEEGDLLGATALALQLMGLWRGGGGGGSAAARLAVAAPTLFVMGSAVVLSGAKLCADTPAVYEELIAVIFILVASVSWTFKVITAVPIAMWALEPLLSGGQNTPLPAWLPLDIHATPAYELLCTFQAVAVTLSVEASVCLDMFFIVLMVAVAGELHILNDNLEAMRLHPVDTFPRKAAENSATRWNGKASPLMEKGIPNTQQYGYHPENHKSTSALYATADAHELMYRSLVKNIQHHQLILKCVKELETAMSYSIFVLLFLNMVTICTLIISTTVLLQSDSDPTSLYKMVSSLPIVMFQTGLFCIFGQMIIDQSERLPATAFGSGWLEGNVRLRRALLLLMRRAATPLRITVGRMYPLSRHTYLQLLNGSYTIFNMMYQVRGRSD

>SgreOR118

VSDVPVDSTTQRISDDMYDQLVGNIRHHQLIIECVQVLQMAMSYSIFALLFFNMTSICLNIFVTASLLQSDADLVTALKAVFTTPVFLYESAMYCIFGQMIIDQSEHLPLSAFSCGWTDADTRLQKALLVFMLRSSQPLRIQVGKTYELSKDTFVRVLNGSYALFNMLYTFQGKK

>SgreOR119

GGATSRGRRTVVQSGLLSTRSQSSLGCVSFTRIVLISLLVKALWGPGEMSDDLGELLGPVAAALRLLGLQAPSTSWSLTWRALLLLVVNASVPAMAASKLWVAPAAKLEELAVEVFVCVTGVAMTVKVAAFLWQQEQTQRLAVLLADCRRRFVCSGGGPGDTRARYRRHIRRIVVYMQVMVVVPTTLWLLDPLVGAGSSAGRLPMPMWVPPGLRSSPGHQILYAFQALAMITVVEASLYLDVCFVVLMLSVSAELHVLNDAVASIKAPAVLRPALYSASYRVTNVINGSWLRCGLSSPRGAIAYNGHQSTEQRTESSAIITDDANGIGTEMYRVLVKSIKHHQAIIRCVEELESVMSQSVFVLLFLNMMNICVHIFVTSVLLQKDVERTTMSKMLCTLPIYMYETGLYCIFGQTIIDQGEQLTASAFSGDWPEGDARMRKALLVLMLRSSKPLKLTVGKLYVLSRHTFLQILNGSYTLFNMLYQVQTKK

>LmigOR125

WRPRGGRAARLLNGLLTAFILASHAFLPVCVALKLYVDPPEELEQITLCSLVTSICMGFLFKAALFVAQGETLRQTVRLLADIRAQFGDRQQNHSTRRRYRRLSDSVYRRYQMVAVPAVIGWVLCPMLSRSVRGSDQAPQVAQRQLPVPVWFPVDVYASPTYEFLYVAHSFCALVAAESSVCVDIFFIHMMLMVAAELDVLNDNLTVMEDVNLYATPNERRGLTSVNGSTGRQSAIHDSSQSTLGENAAREGIHEQLSKNVQHHQAILRSVSLLQSAMNVSIFSLLFFNMANLCSSLFVAAVLVQRDGNVGKALNALTSIPALLYETRMYCIYGHIMTEQRARC

>LmigOR40

MEGRLSARHRESVLKWNVWVLSSGGLWPAGPPRLFAAFTSFVFIVKWTHVLMAVRTLYLSWGDLNEITLTLLSMITMLGGSVKMTLFLKNKSAYYQLVQRLDEVVRYQEQYYLGNETMVSTFQKARKKALRLTFITLGYLNVLGPLWFVMPLLENSSEKHLPFIPMHGLNVTSLPLYELAYVTQCTATFFWHLVSVGLDMFYASVMIYVTAQLTILNLRFMNLGLETKDFIGRPSLGSITASFDNRMFADAHDKMYKELCDLVRSHQKIIEFTNYLEQVMNSTVLVQFLSSVLVACVTLFQATINSQGNTVVKCWLYLPMPAFQIFVYCWCAHDLMDQGLEVSTSAFLSAWVEGSRGLRRGLLLVMVRSGRPLEH

>LmigOR74

MGDAEQPLSWSETEHSALRLNLRHLCLFGAWPLSRCRAYHAYGAFNLALGGWYVGEATASLCFLWGDMEEATLVLISAFNIASIVLKLALFAARRPRYAXXXXXXXXXXXXXXXXXXXXXXXXXXXXXAARLTAAMLLLMVSQYATWFPMPLLASAGARRLPLAQHPWDNNSHLYALSYAVQCAAGAWMTQISFGIDCLFVSVMILVAAQMKVLASRVASLRTADVGFGRKQHPLSSPGEQVYRELCVCVETHQQLLGFVKELENAMSPIVMTQFACSVLVTCVTLFPATYSTDFSAVIRCAGFLPVPLGQLYLYCWAAHTVSEQAEAVSAAAYTCTWVEASERFKRALRIIVSRAQKPLVLTAGRIYPINRAAFVSLVNASYTYYALLCQINKRSTQRVP

>LmigOR47

MVAIGSRAKPILVQQSKTSSKGQDGGEESVVSPSENVLRRNIRILRLAAVWRPPGRWRQRLYPLYFGTVCTSMLHIGALAILRSYTIWGNMTEVTFALVSGLTCFNGAVKMIHHYTHSETYYRLVDELNLLIDRQRPYCEGDAELTEALQTAYKKAKRLTWGVLLYMFVLGQMWCIVPLFMKFPPDDPSSPLPLVTITRVHKVHNHTLYSMAYLSECHTVLYWNWSSLGMDVFFGSIMIHVTGQLNILNIRLSRLSHEGVGDGLAQYSSFVKGSELHKGGIHDSASMYDELCKCVKDHQEILRYLDFLESLMNPVPLAQFLLCVGGICLTLYQITFNPDDGGVIECILFLPIPALQIFIYCWAGHGIMEESEYVSFAAYSCRWGGAERKVTNVLRIIMSRAQRASLLTAGKVHPINRDTFLSLLNASYSFYTLLRQMKNLEEENEASS

>LmigOR138

GEAEAGAEAEAGAEAEAAGHCHKSRRALYAELCDCIRTHQRITKLVRDLQEAMSAMVVAQFSSNVVALCMALYQQAQIKDALTALQYALVLPFLSGQLYFYCWAADAITEQGEAVSLAAYSSGWVGAGGRFQRALSVVMARARQPLLITVGRLYAINTATFVTLVKASYSYYTLLRQLGADSP

>LmigOR52

MAKQKEKSSRLQDLDSGDGEDLSWDEVSHSVLKNSVRVLYCMGLWPLRSSRAYHCFTAFSLASSAVVIAMDIVAACYSLGDIDQMTGALSTILPMSGGLANGLLMILHRPDLCRVVRAVDRLVVHQQRYLRQDSHLAAVVARVRRQTLLVTIGVSCYLITIASYWIVIAFGKPTGLRVLPFVQLPWVQSSGLAHYWSTFAVQFYTAPFCSYATLSVEFFFLAVMLQLSAQFEILGSRFASLGRNPPPKAVVTTKSDMGTADSDAVYEELCLCVKTHQELLRFVRFLDDVMSPFAMLQFVAGTLAVCVVLFQAANNQDLNTNLKAAGWLPAPSLELYIYCGGAHEVVYEAEALVQAAYDCLWYNTAPRVSRAIRLVITRAQVPPVLTAGHLYPITRPTFVSLVNAAYSYYALLCQMQNK

>LmigOR16

MAASAAAAAAPEESVGSAAQSACDLGYLLTFLHWTGTMRHPRAGRRASRAYAAANAAVTLAFVYFVCSQVVVLFRAGTADLDNFTLTLSLIDTQGTWLLRIRHIAAMEPHFHRLAYQVGRDFGQFASAEDVRVLREGSRRMRTVMLLYLAFGLAECCVWLTAPASETGLPFVLALPYDVTRPAAYVATAVYCCFITLHTIMANFAADAFNASLIVQLRMQLALLNRNIVNVNRIVEQERSSPYKSESADALRPYKSYSTSDVNERLRKNILHHQAIIRNVQLLQSCLGSVLLGQSLSIGISVCFQLYQVAKSAESLQDAGKYSSYLFTMFAELFVYCWFADDLISESENVAQAAYEAVPSLLECPTPVKRSLLILMQRAQRPLTITAAGLFPLSRESFVSIVNVSYSFFAILRNFKED

>LmigOR96

PGRRLRHRLVLAFVVASHLGCFFGALASITMDTPSDLPQLSFIAYNCLTDAGLTCKMLSFSLDGRRLTELLRLLSESRRRFPDRAGHRARQHATAVRMHRFLQVTYRINTAYWLLAPVVRNIVAVISNKPYSRDIPIPIWLPFDIRSSPAFEILYSLELAFGWAISETTVLVDGSLIAMILQLAAELAVLNDRLAAGTTPAVKQTPSDAATTEEGTLKDSRVRMPTKSSSAFVETTCTDFLQSRSSIGSEDEMYQQMVGYIQHHQTIISCVRLLQKILSRATSVLLFCNTISICFQVIATAVLLQEDGEIVQTLKMLMGSTLYAYQVALFCLLGQRIINQSERLIRSAFCGDWPDGDVRSWRLVYMLCMSTKKSLSLRICGIYTLSRMTLLQILNVSYSLLNFIYQTKTEQSSGKQEL

>LmigOR120

MLRLLGLYQQKGGGGRVKPKLLSALSLIFLLYHPVFAAMKLYMEPPEDLVEFALCSFSFIISDGVFVKTAIFIADRGMLHQMLQVLSDSRRLYGGEETSKKIRNRYENLAERVLLYMQVSTMLASVGWLAAPLVFRALAMASGDGGEVPRKLPLPVWLPVDVQETPTYEILYVIEAYCVTLTGLVTLCIDVLFIRLMLMVTAELEVLNYNVATMAKRREKISDERRSLEYQQGTESQDFYRGDKALPMASESYEDAMDNELYQQLVTNVRHHLIILRTVDLLEAAMSKSIIILLFINMGALCSNLLVVGVLLQAGEGVTRPLTLTAMIPFLLYQTGMFCVFGQMVTDQSEMLTTSAFSCGWNESDARFRRSLLIFMAMVNRPLEITVGKTCKLSREMFLQVLNGSYTLFNMFYQVHSTS

>LmigOR39

MSKELFSLPGPNSVINVNIGVLKVAGLWPTRPYGLFTIYTVWIYLTQWAVFALDFMSLFYYWGNLNMITAVFCNLTSITAGIIKMTHFFVYKPKYYMLVNKLDALVDSQQKITHPNVDSKSILLQTSKLNKYSTYIIVTYGNLVGVPWIVLPFVVDSGDTERTLPVVEWYGITQDKSPVFQIGYVLQCLTIMYWFFASWGLDLFFGALMIHLAGQFRILNNRIANVGREVDPRLDVSSTLKIEMKEINIDAATRTTLVQRKDAELYSELRKCIMDHQEMISFMNDLEETVNFVVLVQFMAGTLVICVNLFQAALNVQDFSSVLKVCMYMFELILQLFIYCWCAHDVMVESERLSTSAYFSEWAGAPRRFTTALHILMARAQKPLTVSAGRIYTINRSTFVSLINASYSYYAILRQMSDR

>LmigOR45

MAATERRQGEVGEDEVLRSNVRLLQLAGAWPPTAPRGLGRLFPLHLASVYLSQLANIAMALRLMQTARGDMHEITQALMNAMTLVGGILKLLHLSTHVPAYRRLVLALRDVIRIQRHQCERDPHVASLLARAHRKALRLTFWPIAYLNMLALGWYSIPVVFWALGWEKRLLPFFTLHGVDSYDFVLYAAIYFVQCHAIFYWCFISIGQDMFFVTCMVHVAAQLQILNARLSNLGGGQGASNELGRLYSCRAEEDGPCERKPISDTMYTELRNCIKTHQEILKFVQLLQQVMSPVAMAQFLCSVGAACVTLYQATFNPEGNSSLKCLMYLPIPAFQIFVYCWGGHELMENEENIGSVRACRSSASQWLFAGRRVTSSLRTLMCRAQRPLLLHAGKLYPVSRDTFLSLINASFSFYTVLRHMNNR

>LmigOR103

MEALEKGEPTNGELHLINGTFTGPQMAGRSCSSPASYSKYFHDAPVSGHLGFVKTLDWKGFYRSVNHYEASAANGAESAGRQEAVPRQRSRHPGKIRQDCLHRVETVDGESPRTGVTVTVVLWMGDPLLQMLTSPAGNSSRPLIFWVPLEVRHSPAYEITYAVQALGIAAIGQTSILMDIFFVVLLLQAASEIAVLNENIAGMGFKKLRDRENESEEHTSIQRVESSTYVVRTSYVLSECTPHPEISSDNNGNPFAFDRGHKYCKMYSTLVRNIRHHQHIIAYVKDLEVVMSTSLYLLLLANALNVCLHSFGFVALFQEGATRSTVIKEVLSFPSFLGQTALYCFFGQVVIDQADRLHYSAFSCDWPHADEPFRRSLRIFMMQAARPLNVKVGKLVTLSRKTFLQALNTSYTIFNMLFNVERRS

>LmigOR87

RRESLRSHVTATCLMNCGGGRRLRTDLRLQQWLLFFVGAWAPHRGAPAVCSLLYGVYSACVVLVLLLFVASLLFAMVHYWGHMLGVTMNACLMFTYVMNSIKIVAFLKMRPAIDQFIDELDNCMQEYGGEQQSERAALFGWTALKSRIVSVARLSVTAMGCVYWSVMPAVRARACGDTVRCRARVGLPAHVWYPFSYTQSPVYEVIYAGVAAGLMYGALLSSIMDGFLVSLFIYMAAHLQMLNLMLQNLCVDQPQDGSKGLPPGHHQHLCRWRLAQCVNYHCRIDRSVQRLSMLFGPILLGQFMMDIIAISATAFVAIAKNADSTWLVKYTSYLSAVIQQLLFYCWFGTDVLTESERLQTSAYSSQWVDASPLFRLELRVFLCLAHRPMRLTASKFYTISRETFLMLMNASLSYFAVLREINAK

>LmigOR133

MDAVVGPLLPLMRLLGLWPCSGGSGRLPAAARCALTQLPVALMVAGSALKLCVDTPDQFEDVALCAFITNVVAAILVKAVMLVARGQRLRRLARLLADARARFPAHRSCTRGRYQALADRMERLFQVGGLVPLACWLSAPLVPQLTAAPGQGRGRPRQLPVPTWLPADLAASPTYQLVYTLQVLGCIGACASTVCADSLFVRLMLLIAAELQVLKENISSLRKTDSVRGGGYACRCRETVSFLASACKDCHDIVTPLSEKTTDEMHQLLVKIIRHHHMIMRMVSLLQEVMDVSIFILLFANMVNLCSSLFTAAILLQGGGSVVKVLKGLSPLPVVLYQTSLFCVFGHIITDKSGELTDAAVSCQWVDCDTRFKRSLLILMTVALKPLKITVGRVCTLSREMLLQVFHGSYALMNMFYYYHHKTK

>LmigOR3

MEEAVEAARGRAPRLLQTLRGDVAAPAHYLAFNVWLCRLVGLLPRPGQPLWATVQPLVWSCLLLHLMCELVDIALNIADVQQLGKNLPISSLVGGSWYRLSYFTMRRDAYWRLVSKVGESFHRGAPGRMRRWLRRSRGFTLAYFVYGTIVCLFWLGHPLLLQQTTHTMFTSSNSTNRSRMSETAEFPSGAWYPFDVRERRVYGAVYGFQCLALYFAAMLIMVTDIMFITLMLLACGQFEELGDKLRHCWEIATTRALSRAGTTPERELQKVLAHCVRYHDMLLGIVGDIEDLHWTSMLVNFVLQLIILSFLAFEATASADLTNPLKGTNLLMYLVMAIFQLFLLCSCGDRLMEAEEMVARAAYESQWFDAPQGAKRSLSIIVMRARLPQRVTVGKVVGLNLVTFSETLSRAFSYFTVLRQIRTSN

>LmigOR63

LTALCLIWALSPVVVHPGQRWLPFNHFPLEPAPLPLYYELSYAVQSASSLLYIQVSFGVDFFFTVVMILITEQLMILNARLAQLHLYAGGGKSRAAATRVMTTAATEDRDEMYEELCHCIDTHKDIMRLISFLDSVMNPIVLTQFTLSVMAACLTLYQQTYSPDGNSVMKSASYLPTPGIQVFVYCWGAHSIMEQGEAVSAAA

>LmigOR84

MAAGELPEPLVDLRLPCGVLRAVGLWRTGEGGLFYSAYTVWCLLMLVCSVLGQLAGLQGHWANLPTVSTSVCLTLTTSCTIFKALVFLRQRRRVDALVSRVERSVSAFWPLPPARRAAMLLEARRSALLMFSIFFGIGAVALSLFYAGPALQNIKDRELIDSAPDNATLDRHLGRNLPMLIWWYGGQPVEAPYYQLAYALICYWFMLIYLSTSTLDAFYVTFIIFLSSQLKMLNAALADAVQSAGLSDELGNRSGGQPTGAAAGRRADYRRLVHCILFHQQIIQSVHEMESLLSPSVLTQFATSTLVICFTAFAVTTSTKKQEMPAYATYLATMFYELFMYCWYGNELLEQSDRLRLSAYSSAWPDAGGHFQHSLCIVLSRAQRPVCLTAAKLYKLSRETFLVLLKGSYTYFALLHQMNDRQYNA

>LmigOR48

MASPDALLRHNVRLLRLGGAWPPEHGRGLRRLFPLYTASVYFCQSATIAMGALLTYELWGDVDAIMLTYVNTFTLLGGFVKLVCFSGDVRGYRALVAQLRAVARHQWPHCQADAHLMAIFGAAQRAALWLTFGPLAYLNVLGPTWFFMPLIVRATTGSHQRLLPFVNMRDSVTEIFPLYVAIYVVQVYCMFYWNIISVGLDMFFVSCMIHVAAQLRILNERLSNLGRARADDDDDSCRAEANHKNIPGFGSSQKRSFSREGRVGNMYEELRNCIKTHQHILSLLKTLQRMMSPVAMTQFMCSASGACITLFQATFNPEGNSTLKCLMFLPMPAFQIFIYCWAGHEIVYQEELLSLSGYRSGWVGCGRRASALLHILMCNAQKPLQLTAGKFYPVNRDTFVTLINASYTFYTLMRQTRDQGSIVQT

>LmigOR90

MKKASAQYSQESLSARRGLDVSTCERILHWFSCWRGGSAWYVVYVVVARASVAGVVVSQLGLIPHVWGDLYSTSLCVYLTLAVLSAWFKMVQFDLARPKVDALLQELRGELLLHAEDLSEGTEDVFRLFQKRSGAFRRVFLVLDHLTILSWAAKPFLDRLLGSDGPLSLPQDAWYPFDTTRSPYYEIAYLHQVVSLYVAATCVVTVDVFFTTLFMYVTAQLRVLNLHLRAMDCRPQLTQSEKITRCDKGVVAEKSGDANRLPSADPPDAYAQLVHNVQHHQVIIRTVKILEDAMGPCVFVQFLFNVIVICIYAFVLAGVHSMNIGSLILCLTNLISTCVENFGYCWFGNEIMSQSEQLAFSAYSSGWVGGGGRFQKALVVLTMRAQRPLCLTVGSLYTLSRETFLALLNGSYSFLAVLLQMNNSN

>LmigOR31

MCVRVPWRDSALWANARLLSVAGVWPPPSSGGWYLLYTCWLFGSQLFMVAGQLAGLWRFRGDLDKLTLDVCLTVTVVMGVIKAGAIVARRRRFFGIVRRLDAATAAQLLAGDPEEAAVVASAASLARTITVWAPVLGSLSPVVWGLAPLLLRLLGNAPRPRELPVVCWYGGWDAASPYYELLYLVQFVTIQGGYLVVMGSDLFFVSLMIHAAAQLRILNMRLVKIARNEVENDKTFGGMKCVPGYRNSNLKIIDKEWTSSATKTQDLTDETSSYDELRSWVEQHKDVIRLVQQLEQLLNVIILFQFLGGTIIICVTLYQSSAKTGEVTTLFKLQLYLGTMLSEIFMYCWYADGIVQQSARLATSAYSCGWPDAPQPFRRSLLIIMRRCQRPLSLTAGKFYTISRATFVRLVNASYSYYALLRQMNDH

>LmigOR61

MQSKDPVSKSVSFRPPDGGSQNKDGDSAQPTESQDSFLTWKGSSESVLKYNVRCLCILGVWSLTRSRLYYLLSGTAFLLGVMHIVVAIFGSYLYRDNMEEMTLIVANMFVVCAGVTKLVIFVVYRNNYRQLVTVTDGLTDRQRSYCQGDPALKSILEDSERLAVRLTLFVPAYIATLSVVWVPMPLIAYNERRLPFVQLPFVNEVSASTYALLYVMQTVPSLLFFNVGFAVDAFFASVMIHAATQLRILFHRIKDLRLDGRGTLKLLSGDRHDIMYGELCTCIQLHQQLVRYLSFIGKVMDPIAMTQVVFSVLIACTTLFQANYSADTNTAFRCLAFLPTPGTQVFLYCWGAHNLMEQSAAVSEAAYSCSWVEASRRFKRALCLLMCRAQRPLVLTAGGLLQINRPTFISLLKASYSYYTLLGRVNNR

>LmigOR46

MAVLEASGRQTLGEATELWGDVLRRNVKVLRFGGAWRPAALVGWRPRLFPLYFGSVCGSLLNIITLDMVRSWLLWGDMTAVTFALVSAMTNLNGVVKMVHCFRHHGTYGRLVSELNGLVALQRPYCEADGALLAAFRRASRRAARLTVGCLAYMNVLGQMWCVVPLLTPEAPDSRESALPLVSLPGLRSRNRLWYSFAYLVECHAVFYWNFASLGMDMFFASAMIHVTGQLDILNIRLAQLRREGSTEDQFRSFASDAGRSDRQRERGDGDSSKMYSELCECVKHHQAILKYLEFLESVMSPVALTQFLCSVVAVCVTLYQITFNPEGSGVIKCAMFLPIPALQMFVYCWCGHDIMEAGLSVSLAAYSGAWVGVGRRVTGALRVLMCRAQRPLQLTAGKVYPVNRDTFLSLINASYTFYTLLRQMRNR

>LmigOR126

MEFESMMGPGLPLMRLTGLWQMGRQGGGVSRGLRLATIVLSVLLVVAGSTLHLVFDTPDQFEDITLCGFNIDIVSLDLLKGVLFVVQGAPLRELVQLLCDARAGFTFADINHAIRGRYEAVADRMRILLQATVVLPLVGWLSAPLMSRLAAGAGGSRAPRQLPVPAWLPVDIHATPTYELLYALQAFGCTAAGAFSICVDAFFIRLMLLISAEIEVLCENISAIGVPHPAQGSGGCICRCQPNAADLACTCKGCVKAFTSSPEEASDEMYQLLVKAVRHHQTIIRMVALLQQTMDALVFIVLFANMANLCCSLFATAILLQRGGSLTKTLKGLSAVPVVLYQTSLYCLFGHIVTDQSEKLYNAAISCGWVNCDARFKRSLLIFMVEAMKPLEITVGKFCKLSRQMLLQVFHSSYALMNLLYYYHYNTE

>LmigOR49

MESSARHDDSVGAKRGAVRLTLWKGPEQTTSILRLNVRCLLLGGIWPKSRGALYLAYSAFIQLCSISYIVMCMLSIFSPDGDMNDITLTLLHTFEVVCGVVKAAIFYLKRHQYYQIVRDLDQLVSSQRQYLTASKDDHLLAMLDAAHKKANFLTLVLTGYIYGLVFVWLPFPLILSPSDRLLPFVPLPGRYYKDSLLRYITVYAIQSFVPLALIMVVDGLDCLFVASVVHAEALLKVLSERLASLGHSHYTDFRLQRSSGREQEPRKGNVGDNSNITAQLRSCIIHHQQIIEFLQNLEKAMNIMVLIQLSFAMFNLCMALYQQTKIPDFTSALKYVMYLPFPTMKIFFYCWAAHNVKEQGEEVSWAAYSCAWPDADQEFQKSLAIIMCRAQRPLLMTAGRIYPINKDAFVSLLKGSYSYYTLLRQFESK

>LmigOR85

VCCRQLSLHASRPRAGAGMEAHLSPAVRALGAVCLWKPSRAGCWYHAATAAGSLAALSLLASVAAGLPAQWARGDITSFSMNAYVCFAIFAAQIKVSTFGYWGGPQRLVAQLGAERRAAGAAEAALPPGRAERLLGESGTLLRRSAAAFYACGHLMMVAWYASPLIANARLAPDPDTNATLPRHLLFDAWFPFDPVPSPNYEAALLYQSVTLYIAFITTAVIDVFYVSVMVYLGVELEILNEAVARSCRPFEEQENKDKDKEGEKKEEGRGQGDDCSLLVACVRHHQHLNRCVGTLQEVMGISIFVQFVFNMLLICVYAFVITTTKSDFGTLAKFAMTLESYLFENLLYCWFGNNLIEQSERLPFSAYSSAWPDAGRRFQRSLRILALRASRPLQVTVGSLYTLSRQTFLHLLNGSYSLFAVLHHLNSK

>LmigOR4

CEMAVLGADWETRMLTHGAPCLDLTVYLQPILKPLAATGMWSSSIYSNSVHKRAARAAGLSLLALFLAELCAELVGTPLVVARGGGVDRLIHHLSVLGIHLDSFWKWLFMLTQRRRLTCLLRLLQRCFQLGVLASPTHHLPLRNTYIQKPQQSLDPQPQAMPAQLSEVLGKTRMRSWLVTVVWTVSCVLGASHWFFVPLLKGDTTLRFDALYPFDSQQPTTKQTVYWLQYVCSIYSLLLLCFFDCLLVWLQQLLCVQLRCLATNLRELSPNDEHRRKLAICVSHHEHILRAMRELNAFVAPLLFLQCFKNMIVLCVVAFLASVAGVNDLLELSSLVLYFMAACQQLFFYCWCCEELTHLGMEVCDAAYDSGWENWDVSSQKSIIIIMWRAQKPFFFRGGWFYTLNVATFVDLIRLSFSYYTVLRSMREG

>LmigOR141

RQSVTVAERLPLAVYAYIALLCLIWFPMPLLVEPHNPKLPFIQLHYWIDHTRFPVYEASYLMQVVSSFFFIFISTGMDCFFAVVMIHVTVQLKLLIYRITEIRLRDAPAVAAESEARWNRDVGDAHAEMYKELCLCIESHQKILGFVKYLESVMNPIALTQFIFSVLAACVTLFQETYNPDISAVFKCASYLPTPGAQVFLYCWGAHS

>LmigOR36

MPAAAPWSESALWLNARVLALGAMWRPPGCRGPALSACWALYTGWMLFTQLSFLVAQARALWHFWGDVGKVTHDVCLMVTVVLGLIKFGVFSLRKDDFFRIVRQIDSARSEQSRSDDAEIASILRASYRSARNVTLYMTLLGGSSPAVWAVTPALMRKLRVGPPERELPATAWYSGRDTDSPRYELLCVLQLFSMQYSFFAAIGLDLFFVSIIIHIAAQLQVLGVRLRGIGKLGHKQSSSLSNSPILLRDVEDGFYNEKSLWMEFCSCIQQHHSIIELVKEVESLLNIIILLQFLGATVVICVTLFQSSTNTSNFMTLLKLQAYLMVIIYEIFIYCWYADDILYQVNSFTSGIYFTVRLQDLDASQVLQGIREWDAVVEDKRGGGEPDTSSRLSAAAAGKFYYISRATFVRLISASYSYYALLNQMNDK

>LmigOR97

MEEEEASELLGATAVALQLMGLWRGGGGGVAAQAAVAAPTLLVMGSAVLLSGAKLCAEPPAVYEELIAVIFILVASVSWTFKATAFVGQRRRLQALAALLVAGSQNYGDGSGTRAHYRALARRVFIYTQAITAVPIAMWALEPLLSGGQNTPLPAWLPLDLHATPAYELLCTFQAVAVTLSVEASVCLDMFFIVLMIAVAAELHILNDNLESIRLQPVHSLPLKPVDDSAMQSYRNASSLTEKDIPQQYEFYRDSHKSMNAIHGTADAHEVMYCLLVKNIQHHQLILKCIKELETAMTYSIFVLLFLNMVTICTLIISTTVLLQSDSDPTSLYKMVSSLPIVMFQTGLFCIFGQMIIDQSERLPAAAFSSGWLDGDIRLRRALLLLMRRAASPLCIIVGRMYPLSRHTYLQLLNGSYTIFNMMYQVRGRSD

>LmigOR83

MDDGALPQPVVDLWRPCLFLRAIGLWAPARLRWLYSAYTVWSLLQLVVSVAGQLAGLQGHWDHLPTVATSVCLIVTSICTLFKASSFALRRGRVDSLVSRISHNLSTFCAHRPRTRAAVVWAARRRATRMFDTFLGIGGVALVFFYLGPIIQNAKDARVAASAAGNETLPPLLGRNLAMLLWWPSGQPVETPAYQLTYVGVCYWLMLLYLSTSTLDAFYVTVIIYLSSQLKVLNVDFLSITEGDDDSPAEETDPLSKRGGKHELEAKLPKGYEEATDQRTQERLLECIIFHQEIIKTVDEMESILSASILVQFLASTLVICFTAFVITTAENKQDLPTYITYLATMFYELFLYCWYGNELLAESERLQTSAYSCGWVGRSAGLQRSLRVVMVRLQRPVCLTAGKFYQISRETFLLLLNGSYSYFALLHQMNDH

>LmigOR95

MQQADPLWPLCSVVGELGLLGLWLPPPGRRLRHRLVLAFVVASHLGCFFGALASITMDTPSDLPQLSFIAYNCLTDAGLTCKMLSFSLDGCRLTELLRLLSESRRRFPDRAGHRASQHATAVRIHRFLQMMYRINTAYWLLGPVVRNIVAAVSRQPSISVRDIPVPLWLPFDARRSPVYEALYGLVLAFGWAISETSVLVDSSLIALLLQVVAELAVLNDNLASGTPPAGKLTTRVTADAAGSRVQIPLTTAPSVLEGAVCDLVHRGEVYQHILDNIQHHQTIISCVRLLQKVLSRATCVLLFCNTISICFQVIATAVLLQEDGETMQTLKILMGSTLYGYQVALFCLLGQRVINQSERLIRSAFSGDWPEGDVRSWRLVHMLCMSTRQSLSLRICGIYTLSRMTLLQILNVSYSLLNFIYQTKTEQSSGKQEL

>LmigOR130

MKPVGGGCGGLLGPGLTIRRLMGLWWPQGGRGRIAAAAAATLTVASLTMLVAFPALKLIMDTPSELEEITLCCFVIFLCSGFIIKSALFIYQGDILKELLQLFSDNRRIYSNDRNSEGIRQSYIKLSERVYVYMQVSVLPAVAGWVSAPMLARFFLTAESTQQFPVPLWFPGDIYQTPTYEILYAVQSFCVLVTGQCTVVIDVFFIHLMLMVAAELHVLNENISLMQKLNVKTRVSEAEEWQFRIRRNDEELTFPIHDHRARVGYFCSEDVSDENMCLKLVKNIQHHQQILRSVLLLKSVMNVSIFILLLLNMADLCSCMFITAVLLQRGGDVTKALKPLLTIPPLLYETGMYCFFGQILTDQSENLIDSAFSSGWVDCDSRFKRDLLIFLMAANRPLEVTVGKISKLSKQMLVQVLNGTYGLLNLLYHFHGSQ

>LmigOR123

MGHSSEERESLEGPGVVLRRLLGLWRPRGRLARGLDMLLAGVTLVAISFLVVCVALKLYADPPEELEQIALCGLVASLCIGFFFKALLFMVLGGTLRQTVRLLEDTRLEFFSGDKNETTRRRYQQLSRNIYNYGQMVAVPAAIGWITCPLLSRLLTHTGDDQHEVKRQFPVPVWFPVDVYTSPTFELLYAVQSFCVLVVAECCIATDIFFVHTMLMVAAELEVLNSNLCAMGDAKLQMKRVKEEEAVSRYKTKGRRWAFLNSDQPVGEHGLPENAVHEWLHEQLVKNVRHHQAILRSVSLLQSTMDVSIFILLFVNMANLCASLFVAGVLVHKEGNVGRALNALVSIPALLYETTMYCIFGHVMTDHSERLMYSAFSSGWINSDARFKRSMLIFMMVTMQPMNITVGKTYTLSKQMLLQVLNGTYGLLNMLYHMHGSE

>LmigOR92

VQRPAAGMRVTAVHKQTALASEAGRDLMGPGEAVLRLLRVWPPPGPEARGLLHRLPYPLLALQAGMAGVLASTAAHLHQGGAGGDEESEAQQTTVALFIVGTIIGMLVKIWSFMGQRGRMQVLLSLLLEMRRRYLRDHTGARPRAQDHGVTLFYILQGNAVLAALAWSVQPLLSGSARRLPLPAWLPFDATVSPYYEAVYAGQALSLLLVPQISLCINICYFALMLHLAAELAVLCDNVAAVGWRRTSKEPAPQSQQQVLDREFAASSENRLLEDNVRHHQLIIRAVSELQQIMSTSVYIHLFVNMINVCSHIFVISVVLLETGEMAVVVSQACSLAVFLSGIALYCVIGHTIIDQSERLPEAVYSSGWTGADLSFRRSVSILLVRASHPLSITVGKMRVLSKPTFVQVLNGSYTLFNFLYRTQSDKEQRERLGLQDS

>LmigOR129

MSMPDSELRTLLGSGASIRQLMGLWWPRGRRGRGRACSAAAAAVSLASLAWLPTFSGLKLLIDPPPEIEEIAMCYLLIFACTGFFSKAAFLIYKGETVWKLLDLLSETRRLHRNGESNDNIRLSYQQQSRRVYLYMQGAICVAFVFWVSTPLLVRAFLASDEDSPESYRLFPVPLWFPGNMYLSPTYEILYSVQSFSVLVAAQSTVCVDIFFFHLMLMISAEVQVLNENIALMEKVNLKSEKQEDHELRLNIKEKAEDLSFRSVGYSTGKALTEEVSDENMCVQLVKNIQHHQLILRSVVLLQDIMNLSVFILLFVNMVDLCSCIFVGAVLLQRDGNVTKALKPLSTVPPLLYETGMYCIFGQILSDQSEKLTDSAISCGWVDCNDRFKRDFMFLLISAKKPLEITVGKTSKLSKQMLVQVLNGSYGLLNLLYHFQSIQ

>LmigOR8

MDYQLGDHGCGYPLRCITDRSACDGVRSDEPGCTNGKTSFFSDESRFCLRHHDGGIRVWRHSGKRTLQACIRHRHTCVSPGVMVWVAIGYTSRSPLIRIDGTKRQEELCLATDSSYIVDNGIETLAVLWGFEKFRYFLYGCKTREYTAHSLDLSPTENVLSMVVEQFARHNPPVTTLDELWYRVEAAWASVPVHAIKVLFDSMPRRIKAVITARGAVLTRLCPVVPQYPIIALYPWPVQSGPAYALTFSLQVLCGGLFTMTHLACDTFLLSLLIYICSQIDVLCASLRQLGRRLLRIVSLVSYRYVGVLQQVLSPVALAQFMCSMVIICLSGFGIAISNDFGSLCRYSVYFTGAAIQLLLFCWYGEVLITKSEHVSEAAMACGWPAVRRGRFQSSALLLMVRAQRPLALTGSKFYVVSLKTFVQVTNAIITYFKIAPTLGK

>LmigOR112

MGDGCEALGASVSVLRLLGLWVPTESSGAGGKAAYVPGALSCVAIGLLSLSCASKLFIDTPTELTELTVCAYLFVIITANFVKAFCLLLQQGTLHELVTLLVEAKKKNVIDVQHNEDIRSLYGVMSARLYRYLQVMIVVSSVAWLFVTVVFRVITAGSTNIEWPTPLPIWLPLDTQRSPAYELVYVAQVSCAVVTAATMLGADTLFFHLTLMIVAELQVLNDNVSVLGRPAPPSADTRQVVCTMNGAGERQTHPPGGVAERQSDSDGAVSLSDHQGTAAAESKYLVLIEIIQHHQIIIKMVSLLQTIMDYSVSVQLLTNVLDMCFLIFTMSELLHHEKSLHAVLQTILSLPCLLCESGAFCMFGQMIIDQSENLVHAAFSCEWLEADGRFRKPLYTFMLMATHPLQIKLGGTAKLSRSTFLQALNGSYSLINLLYHSRRPVG

>LmigOR66

SYAVQCVAGLWMAEISFGMDCLFASVMILAAAQLEILSGRILKLGQSPYVAKKGSPTPDEIYKELCRCVETHQKILRFVSRLQETMSPIAMTQFVCSVLVLCVTLFNATYNKDIITSLSSMTFLSNPCGQVYLYCWAAHNVAEKANAVSTAAYSCSWVEGSERFKRAVRILMSRAQKPLVLTAGSLYPIDRAAFLSLVNTSYSYYALLGQINNR

>LmigOR70

MEMYQGKGMLKTLSWTESGRSVLKLNIRHLWLLGVWPLGHSQVFKVYTSFTFAMGVWSVVECLLAVYFTWGHLEETTLVLIFTSTCSCAIIKLVFFLRDERSYSLMVREVASVMAAQSEACRDPALAAILRDSRSRTFRLSLGMLLFMFSQCFIWFPIPIVANAGERRLPFSQHGWDNNCHFYELSYTLQCLSGLYMSQISFGLDCLFASIMILVAAQLKILSGRVLKLNQEVIPPERNDSVLLRNQLAGDKYCDKFYEGLCFCIDSHQRILSGGVQTEECLRVAILDVWRVALFCWNWFVTLLQDTMSPVAMAQFASSVVIACMALFQATYTLHSALYHIHEQLLSVDSKNVLSAIFLWSPSPWHSPPDVQLGTIPRSFANGEGESVSVAAYSCSWVEGSAAGRLYPINRAAFLSLLNASYSYYALLGQMNKRSMKDLASHN

>LmigOR107

MESEAKSLVGPSGWALRQLGLWRPPGAAPAGPRLLAAFFVVATDALVSTSSAVQLAVDTPTNPETLRDVFFQTTCSGAWAIRTVLFMQQRDRLQRLVMTLLDTRKRYAENVPGTRSSYDRGAAIVFFAWQMLPLTAISLWALEPATVPAEPVLVGNTTVVLRREPLVLWLPIDTQRSPTYEVVFVMQVIGIATVSEVSVLLDIFFVSLMIHVTAEVEVLSGNVLNIHLSTLDGQLTQSREYGDGRLSYKGGGQLAAIDEPTDLSAAGSGYLPNKTLFYSQDMDDAQRRLYACLKTNIQHHQTIIHCVNELEEAMSNSTYLILLVNALTICLHAFGFVELFQGGGKGPAVVKRLLACPIYMGQTALFCVIGQSLIDHSERLLDSAFSCGWPSADRRFCSALLIFMRQASQPLKIRVGKIVTLSRNSFLQIMNVSYTIFNMLLNTQ

>LmigOR108

MACTFSLPTGCTTRGLTAAPCGADGVGGAFAGGPQRVGAAAAGAVAALVSAASVVQLAVDTPTDPETLRDVLFTSTCGLAWASRMVLFMKQGVRLQRLVTTLLVTRKRYAEEFPGIRNSYDRAAAVIFYAWQVLPLTAVSLWALGPVTGAPLAVASGNYTVLERREPLVMWLPVDTQRSPTYELVFAMEMVGVYAVAEISILLDIFLVCLMILVTAEVAVLNRNVSSTRLSRLDQDQPGGVTAIGREGYGGLSADGEWSAAANQTLPNSTLLPYQDTDAAKRRLYDCLKRNIQHHQTIMTRREQRTAFYYLGSLPASHKSVNLFHNLVSSLAGIRFLKYAAIKLVLRPELQMLVFGVLVTIIKTALFCLIGQSLTDNSERLLDSAFSCGWPTADRRFCSALLIFMQQASQPLSIRVGKIVTLSRNSFLQVMNVSYTIFNMLLNTQ

>LmigOR2

MLRQVYEALRDADEPSRYLEFNVLMVRFMGVLVSRSLTGALLTWSLFVLLATHCLAGVFDLVNNSGDIADITANLPVTTIIFSSTYRLFFFTLHRDRYQAIVDTVGARFVASSDSIDMAPWLRRSRIISILYFTYGFFVASTWQLHPLISAQLTAAQMAKESNGTFEGLPRELWEFPTRAEYPFDARQPYVYTVVFILQGAAIFVSGCMILVLDMMFITLTSLICGQFEILKDKLRNMRKIATNQRQNEDIGLVGKALQKQALERRKRILDSNIEITKQDENFNEDLITRKINLLLGECVEHHNMLLSLISEIEFMHWSAYLVNFCVLLIIFSFSAFEVTSGTPTSPAKVVNLAEYLLVSVLQMFLLCDCGDKLVDQELSVSQAAYESEWYHCSESVKRTLQIIVLRSRQPEQITVGKIAGLNLDTFSDMLSRSFSYFTVLRQIRDDS

>LmigOR105

MKNQMHSDEKVWDTTKDVEAMLGPSAVVMSAIGLWQPPGGRPAGAKVLVTSAVLLMMLTIFLGGLVQLVLEPLPLEEILDAVFTCACSFTWGIRVMVIRLRQRRVQQLVVDVLNMRKRFTENAAALRKKYHRRGLMVCLAWVVFPALAVPMWFVEPAMTKKLVTTSENTTMVIRKTPFIMWMPMETQTHPNYEITYVAHLSLLVCSVSPTLIIDLFFACLMTAITADIDILNNNIANMRLYKEDGFTENSEKGVEVTATWETTYQSKKSLAEKSERVDDNTQVLATYSTDPYEQLHRTLAKNIQHHQTLMSIVGDLESIMSESSVLMLIVNSINICLQALGFVDAFRPGAKRSTVLKKVLTFPAYINQTAHFCWLGQAIIDQSERLQDSAFSCGWADADQRFCSSLRIFMLQASRPLKLQIGKIFTLSRNLFLQILNTSYTIFNMMINF

>LmigOR102

MEDAAGQSATLLSPCSVALAWLGIWRPPGGRRSGLGLPGAVFIAALDITISSLALVQMLIDRPEDPADFREVFFICSCGVSWSVKVVAFLLQGDRLERMVLSLLDAKTRFPDNGSRVREKYTAMAYTVWRMWQAMPAVTVLLWMADPLLQTIISPPADNATRPLIFWLPVQVHDSPAYEVTYAVEAFFIGTVSETSILMDIFLIILLVYAAGEIAVLNENVARMGLTMQRETAKAQPVESSSEKASMSKTRYVPSGDSPAADVGAGGKGQLPLLDGDDALWDMYSALVTNIRHHQAIIAYINDLEVVLSTSIYILLLTNALNVCLHSFGLVAFVIIVFLQLLAEGATSSTVFKEVISFASFLAQTALFCFFGQLIIDQADRLQFSAFCCDWPDADESFRRSLRIFMARATCPIKVTVGKLVELSRNTFLQALNASYTIFNMLFNLQTSDE

>LmigOR41

MAHAQRPRGLPPANVLSANIAILRWSGLWPPERRGGWARLFAAYSAVAFLSQAVAAGMTLHLIYHSWGDIYEITLTMMVTMTLVGGVLKMLHFFRHAGAYHLLVRELPAVAAGMTLHLIYHSWGDIYEITLTMMVTMTLVGGVLKMLHFFRHAGAYHLLVRELRRAALFTRGALGYLNILAPTWFLMPVISGAADDPAGRKLPFTQLKGLRADDLVGYSVAYFVQCHAIFYWNFISVGLDVFFATAMLHAAGQLKILSHRLSRLGKGPAARQHWNPQDDTTQIRQIMPQEGTNDLYTELRSCIKNHQEILRLVLLLESVMGPVAFIQFLCSVVAACVALFQATFNAEGNGVLKCTMYLPTPAFQIFIYCWCGHEIMEEFSRYSLATRRACXYSSGWVGAGRRVSRGLRVLMCRAQRPLLLTAGKLYPVNRLTFVSLINASYTFYALLRQMRDR

>LmigOR1

MSNGNEGIYFGTIINLMHCFKIWSPDDSKRTISFSVYILVPAYVFFFALSCIEIYHNWGDMLSTTDAVNTFVIYLATSHKYFRLIYHEKDLKKLMKMVENNFSVPVWQNDALRNSIVKSYVQEVKKLTILWTTLCFTTLCGFMILPLVDGLFHYYTTNVTTEIEWKLPYRTWTPFNDYGAIVTVPLYVYHMFMGFVLIAEIPAFDTIYFSLINHSCAQLKIIQNSLINIVSISAQNVLSNEKNGVTFDTMLDEHYTAEENKDQSSLENKMNNGLNSYTPSGSYDSMLNLLHDSELDKKIRKNVGELVNHHEKILEFIDGVEAIVNAVFLTQFLCSATLFCLTGFQLTVILKEQQLARFLNMMELLGAAIFEMGMFCYYANRVMDEGINVGKAAYDSQWYYASKDYGNSVSIIMARCTRPPKITFGKFADLTMENFASVLQISYSYFTLLTRINE

>LmigOR106

AGLGSQLACLTAAKAMKDHVQSDGQVRDTTQEVEELLGFSAVLMRWMGLWQPPGGAPAGPKLLIALSFDAMFFVIVAGGFASVVLDPPPMDSVLEVVLTLASSVTWGVRNVAILVRQNRLQNLILDVLDMKKRFAENGTEFRKAFQRRAKIRTALLVGIPMLGIPMWLVEPAFSKTIVATSENTTVVVRKTPLVMWMPMDTQTHPNYEITYAFQMLLISIVVDANVIIDIFFSCLLITVTADIAVLNNNVANMRLCKDDGFTKESGKEVNLSATWETTYKSKKSLEEKDQLYDGNTDVYASHTTDPSTQLYRTLVKNIQHHQVLMSIVNDLESIMSESSVLMLVVNSINICTQGIGFVDGFRPGSNKTTLMKRFLTFPAYVNQTAHFCWYGQDIIDQSERLLESAFNCDWANADSRFCSTLRIFMLQTSRPLKMQIGKIFTLSRNMFLQILNTSYTIFNMFINF

>LmigOR44

MSRAYIAHVGNSLTESFSPKLLRKRTVLFFLSLRSRGRALEMPEQKVQLQRGARICDVLRHNVLLLVATGAWPPTTRRWWRPLYPLYTASIYFSMLATIAMGFQFAYQSWGDWDSIMLTFVNTFTLIGGAVKLAHFSSHVDAYRRLVTALRDVIGTQWAHCERDAALMAAFAGSHRKALWLTWAPVVYLNILGPVWFMMPVVAWASGAPGRQFPFANVRGVLKTNFPLYVAVYFVQCHSVFYWNFLSFGLDIFFVTCMIYVSAQLHILGKRLSNVGRGPNVDQNGIVDEKQTKLQQFGQKPYKSLEVDRESNEMYAELVDCVKAHQHILSFVAVLQGVMSPVAMAQFVCSATAACITPFQATFNPEGNSIFKCLMYLPMPAFQIYIYCWGGHEIIDEGAALSASAYSCAWMGAPRRVTSAMHVLMCRAQKPLTLTAGKLYPVNRDTFVSLINGSYSFYALLRQMRGH

>LmigOR139

AEFEVLSDNFAALHKVHEQSEKGKIRGTVYERQIKEESPPASCAHIYIECVSDEQMYRQLTQNVRHHQALLRSISLLQTAMSAPIYLVLFVNMVNLCTNLFIATLLLQRDGSFSKALAVLLTIPTLLSQTAIYCLFGHALTEKSEKLTQSAFSSGWPECDVRFRRGLLMVMTLAEQPAEVTVGKMTKLSKQTLLQVLNGTYGLLNMFYQLHSQM

>LmigOR104

MLHVYLGTCFTASPLRQWVSAPACRQVATAAQEVRRLVGPAAPALEWLGLWHPPGSPPTGFKALRGATVLLIDLLLFVFCSLLLLLDPPADAEGQRETFFYIMASFTWTVRGVFFMLERTRHEKLVSILLSLRQRFPDDGCDIRGTHLKNAALLSLAWQVAPVLVLPAWIIEPMLETHYVTYGNITEVYRRTMLYMWTPGDMQQSPNYEISYVSQVVVSLIAVEASVLQDIFFVNIMVQVTSELDVLNANISSMRLPAAAKSQSVANGPEYSDTKLEQYEATYFSHKSKTSAEDELQRSVTIYKNTNDENEELYKKLVKNVRHHQMIMVCIDELEAAMSKSIAVVLVISTLNICVHAFGFVGMFQEDAPRVTVFKRTVAFVIYMTHNALFCFLGQSITDQSERLLHSTFSCGWADADRPFKRSLMIVMRQTSRPLVINVGKFFTLSRNTYMQIVNTSYTIFNMLLSVQ

>LmigOR33

MQVTRHERRFCPQPGTRTVSFLIRYRESIRTIGAIFQKHPFPELFNVVMQVTSHERRFCPQPETASMREEDIPWSDTVLWLNARVLALGGSWRPPGARGFALYRLWVLFTQFSFLIGQLQGLYYFWGDTNRILQDVCLLITTILGLFKFFTFVARQEQVFRIVRSVDDRRREQSKLGDARVTSVLEASRRAARTITVWMAGVGGLAPAVWASMPLVMRGLGLAPPERELPARARYTDRDTATPVYELLYLLQFFSMQYSYFAAMCIDLFFACLILHVAAQLEVLNVRFGQIREDYYRNGQTREARDDAKVEDVEEDAAWKELCECVEHHKGAIKLVDDLETLVNPIILSQFMGSTIIICVTLFLITTNKQHFAALVRLKAYLAVVVYEIFMYCWFGDDVMYQNSRLVESVYACGWPGAPPRLQKALVVVLQRAHRPLGVTAGKFYRVSRETFVSLMKASYSFYALLNQMND

>LmigOR131

MGRASAEVSNSRPLLGPGAAQLRAMGLWRPGRSLLHSLAAALMLACLAWVSATAALRLLIHPPAELEEVALCSFIATICSGFTIKHSERIIVAKVDRIPTPTTVVQVYMSTSTADDEDIEEMYEEIKEIQKVKGDENLIVMGDWNSVVGKGCVVPALIGWTFFPLVSRALNDSGEESPGAVADWQFPVPHWVPVDMQRSPTYHLLYVLQSFCLLVASQSTIAVDLFFIHMMLMVAAEIEVLSENVSAMGKIDSGLVALDDEDCGPSTKYLLSYRDGNGRISEISRKDDISEDQVRALLVKNVQHHQTILQIGEKIQFCRISSKRRHRLVGHVLRLEGIVNLFLKGSGVGRRYFPCLIDLTYQVEHFCSRLALLMISVLLQFYSRGFVSTQILQCSVKFLSHANSKERNVDHGDEGFVSHVVFHVRRALLLFLTEAVRPVEITVGKTFKLSKQMLLQVLNGSYALLNLLYSIR

>LmigOR54

PRLAAAFRESCRWSAKLTFLFFSWVLLALVAWSLMPLTLYPRVRLFPFQQLPWPVLTQSPTYWFLYLHQILATFFFCSIDMNTDCFFATVMTHMSTQFKILASRIADLRLRENTQKSKLCAEVDTSTPHDEMYKELCLCIETHKELIRLVGLLESLMNPVAMLQFLVGAVSSCVVLFSATYSPDSSSAMKCWGSLPLLLTQLFLYCSGAQHILDESE

>LmigOR137

SKAAALLDGAEELVCPNATFLRLMGLWRARGWTGAARGWFCWTLVLFVTATSAGKLCLDTPQELAAVADYGYAVFHLSAVTVKVACFILQRSTIEELVNLLDETRKTYGKTEANYQVRQIYQRRATNIYRVLQALAVAVICMWISSLVIQRQGLKEGERPPPNPIWMPDNSPGYEIVYSVQSLCGSAAVQASMLIDTSFYKLTLMVTAELQILNDNLATLGRAAEA

>LmigOR79

ELSYAVQCVAGLYLSQISFSMDCLFAAVMILVAAQLKIVSCRILKLKAEESGEQGGGSGDVGGGVALENEQKPYENLCQCIESHQQILRFIIRLQNAMSPIAMTQFFFSVLVACMALFQATYSKDFTAVFRCVAFMPIPCGQVLLYCLAAHDVTEQAEAVSQAAYSCSWVEADRRFKRALRLVMCRAQRPLVLTAGRLYPIDRGAFLTLVNASYSYYALLGHINRRSMQDQL

>LmigOR136

RLPFAQHLWDDNGHWYGLSYAVQCVTGLWMAEVSFGVDCLFATVLMLAAAQLRVLALRLVRLKVDAGGAPGDQPGATDGAYRELCLCVESHQEILRFITHLNGTMSPVAMTQFVFSVLVACVALFQATYSTDITAVIKCVSFLPIPGGQVYLYCWAAHHVTEQAEAVSTAAYCSPWVDAGPRFKRALRILISRAQKPLVLTAGRLYPVNRETFLSLVNASYTYYALLGQMNKRSAN

>LmigOR78

HDWSGDGGSFYWPSYAQQCCSAFWLGKISVVLDCQFAAIMVLVTAQLEILSARLANLRPDGRALRESPNCKVKLSYVDHDSEMYDELRRAIQSHQEILSFVSCLQQVMSPLAMTQFVCSVIVICVVLFQATYSQDFATVLKCVAFLPVPCGQVFIYCWAADNMTEQAKEVSSAAYRCCWVDAGPRFKRCLLLVIRRAQRRLVLNAGHLNIDRAAFLSLVNASYSFYTLLAQMNRS

>LmigOR127

MGERGEAPAGLLGPEAAVLRLLGLWRPRERQGQGLTPPAVVAAATIAAVAFIPAGVVLRLCGDFPEEIEETAHCSYICIVCFGCIVKAVLFVMEGDTVRELVHLLQATRAEYGSDEGSDRIRSGYQGTVDRMYRYFQVMALLPTLYWICWPLVAAAVSPGEASGVSGARQLPLPFWLPSGASGTPTYHLLYAVQALSLSLTVASAVCLDVFFIRLMMMLAAELQVLNENIAAIDGCRASGSAYGREEEEFDSLVPSDDRALEPTKKSAANFSDDDLFSRLLNNILHHQAILRCIWLLQTAMNVSIFILLFINMANLCFNMFVTAGLLQDGRNVTKAVTAFSPVPGLLLQTAMYCLFGQITTDQSEKLLHSAFGCGWDDCDTRFKHNLLIFMLMVGRPVEITVGKTYKLSKEMLLQVLNGTYVLLNMLFHVHSDDHI

>LmigOR32

ATARYTDRDTATPVYELLCVLQYFSMQYSHFAAMCIDLFFACLIIHVAAQLAVLNVRIGQIREEYYRGGAEEAVPPEERRAREDEAWQQLRECVEHHKLAIKLVGDLEDLANVIILSQFMGATIIICVTLFLITTSEQHFAALVKLQGYLIVVVYEIFMYCWFGDDVMYQNSRLATSVYTCGWPGAPQKLQRALLIILMRSQRPLGVTAGKFYRVTRETFVSLMKASYSYYALLNQMNK

>LmigOR140

LLDISSPLDGLVGGRTRQALLLLDQAAVAFNHLCSVAAFTTMFVHFVVIACRHLQRSIDDLTADNCDIAAVVRHHQQILRFIREIEEAYCILMFWLFLPMMVVMCLIMFAFLTMTSLDIEFLEMLAFFLIYCVTNGVISICGSMLTSKAERVMVAAYSSAWPERSRGFSGAVRVVMVRFLQPAQLTVGKFVPLSINTFSKLLQESFSYLMVMLSLVNEKDSEAQPGVVVEATANHSAYH

>LmigOR134

ELQVLNDNLASVKAAPLLRSSPYSGWNRATNVTNESWFHSGVSSPTGAIACNSYQSSGDRSESTTSITGYTNRTAVEMYRALANSIKHHQAIIRCVEELESAMTYSIFVLLFLNMMNICVHIFVTSVLLQKEVERTTMSKMLCTLPIYMYETGLYCVFGQTIIDQHGCDVLQSEQLTASAFSGDWPEGDARMRKALLLLMLRASRPLQLTVGKMYVLSRHTFLQILNGSYTLFNMLYQVQKNK

>LmigOR26

EGLPLQLVLPFDTSRPAGWAAGWLFCAFLTVHCVVMNMMADAFNVSLIAQLRMQLMLLRRKIVRIAEEGSSRSLDSLHTTDYRDLHYRLHKCVLHHQAIIRNADLLESCLGAMLLGQSISIGTVACFQMFQVAMSANGLQQVGKFGCYLFAMLAELFIYCWFGDDLITESENVALAAYEAVTSLQGCPLSMKKSLLLVMHRAQRPLRVTAGGFFPLSRESFVSVVNVSYSFFAILRNFKMEDQ

>LmigOR135

AFMMPLWLPLDTQASPTYELLLGVQVPCCWICSETSVLLDCAMLALMLQAAAELAVLNDRLSAVGPGQRRAADDKRDLQANDHMFSGLVDNINHHQIIITYMHLLETLLSRGISVLLICNTISICFHIVATVALLQEDIEPVGMTKMVLGSTLYAYQTAILCLLGQRITTQSERLPVSAFSCDWPSADGRFRKLLMVFCLRSSQALIIRVCGLYSLSRETLLQVLKAAYTLFNFVYQTVGEEEPLN

>LmigOR117

GAGGDSPRLLPLPAWLPLDLQASPTYELVYAAQVLLIPLSTTSVCSDFVFIGLMLRISAELEILNDSISGLHKIRKDVPTAPKNEAKSIGRVSDGEINLHISRNVKHHQAILKSVALLEEAMSTAIFILFLGTMIAISINIFAATAVLQTVGGMTKALKMITAIPPIMFEVGLYCIFGQIVTDQSEKLMHSAYSCGWVDCDPRFRHSLLTFCVGSRRPLEITVGTVYKLSKETFLQVLNASYAMFNMLYGFQSNT

>LmigOR25

GPALHPGAALRLVHAFGLRRHVALLHGGHRARRGDDHGVGRLQRQPHRSVAHAAHVAQHRKLVTIANDSSMRPLDSLRTTDYRELHCKLRKCVLHHQTIIRNADLLERCLSGILLGQSISIGSVACFQMFQVALILLIYNWNHRTSLKKLSFVIGKFGFYLVAMLAELFIYCWFGDDLITESENLALAAYDAVTSLQGCPMSMKKSLLLVMHRAQRPLRITAGGFFPLSRESFVAVVNMSYSFFAILRNFKDQKV

>LmigOR19

QAYIWFGVGACTFFLFSPASAEGLPYILALPFDASQLVGFAVAWLFCMVVTFHVVVMTMVLDSFNVSIIAQLRMQLALLNRKIVSLAKGVNEKLQQCSDTSEYSDLHSRLEKCVLHHQAIIKNADLLEKCLGTMLLGQSLSIGAAACFQMFQIATSANGLQQTGKFCCYLFAMLAELYVNCWFGDDLITESENLALAAYDAVTSLHGCPISIKRSLLLLMQRAQRPLCITAGGFFPLSRESFVAVLNVSYSFFAILRNFKNEDQ

>LmigOR122

MPIWSPVDIYSSPTYEFIYLLQSFASLITSQCCLSIDIFFVHMMLMVAAELDVLNYNLSAMKHYDSQTPISDGEEFISNVKTSGRRLELPSSDKSFGEQALKGDIEGNGLHQLLLKNVLHHQAILRSVSLLQSAMNVSIFVLLFINMANLCSSLFVAAVLLQRDGNAAKALHALLCVPALTYETTIYCTYAHIMTDQSERLMYSAFSCGWVNSDARFKRSLVIFMMVTVRPIEITVGKMCTLSKQMLLQVLNGTYALLNMLYHFH

>LmigOR18

CSATSVSSCRRATCRVVNADGLPFVLALPYDATRPLAFAATWLFSVYIVFCVHIGTMAADSFNVTVILQLHNQLDLLGRNLRSLKDSVSHVKSASTETLPKRMRHSDNSRDIHSRLRKSVLHHQAIIRNVQLLEECLGGMLLGQSLSIGTSFCLQLFQAATRAKRVQELGKTCSYLVTAFSMLFIYCWFGDDLISESEKLAFSAYDAVTSLQSSPASIKRSLLLLMVRAQRPLQLTAGGFFSLSRESFVAVLNASYSFFAILRNFKEEYD

>LmigOR7

MRELEEEWERQRLRGLRLVVRALQLMGAWPPGADAGAGGGGGGGGVRRRLYRAYVAAATALMASYPACALCVLAGTRADIQVTAVLVGVTSSYVGAIFKMFTLCYKRSQVSGLVRAVQREFPRSPLLAGRAQRAVAASAGQHGARLTALFVSCFSSVFNWSLGPIALHLLGGGGGALALCLPWFPDHQPAGAAFRLVYGYQVATLVLVALWVGALDAFLLVLLVYAAGQLRVLNCTLLQMGARSDDGTEGQREKFASNASAMLRECAKHHLEVCRFVQDVEQVAAPALTLQLLVSTFLLCMSAFTATQIPVGSPLLARVVVYMLTGASELLIFCKYCDDVISESERVQQALYGSGWSAQGGAFSRGVLIMLARAQRPLCLRAAHVHPVSLQTFTKVLNASYTVFTLMRQIKD

>LmigOR128

PGAVADWQFPVPHWVPVDMQRSPTYHLLYVLQSFCLLVASQSTIAVDLFFIHMMLMLAAEIEVLSENVSAMGKIDSGLMALENEDCGLSTKDLLSYRDGNGLVSEIFQKEYISEDQMRALLVKNVQHHQTILQAVGLLQDAMDISIVILLFTNMADLCSCMFASAILLQRGGNAAKALKPLMTIPPCFYETAIYCFFGNIVTEKSEQLVTAAWSCGWPLCGSGFRRGLLLFLTEAARPVEITVGKTFKLSKQMLLQVLNGTYALLNMLYHVHRSE

>LmigOR132

PAAPRQLPVHVWLPADLNRSPTYEALFAAQSFSLMVLSQATVCMDIFFVHLMLLVAAELEVLNENLSAMERGRLQHGRSEYSETTDIHGEDSDRSTFTNTDRRLDVTVGTSQREHDERMYAELVKNVRHHQAVLRSVSLLQKAMDASIFILLFINMANLCGAVFVAAVLLQRDGNITKALKEVMLIPCVLYETGMYCLCGHMIISQSERLVTSAFRCGWPDCDRRFKSSLLIFMMAAIRPLEITVGKMCKLSKQMLLQVLNGSYALLNMLYHFHHTL

>LmigOR69

VVQSARRRAYRITLGMLLLMFSQYFVWYPMPFYVDPGARRLPFAQHAWDNNTHLYGLSYFAQCAAGLWMTQMSFGLDCLFASVMVLLAAQLDILARRILALGSGAHDEKAEYPEKKPAPRFGDQMYDDLCLCVQSHQKILSFVIHLQNTMSPVAMTQFAFSVLVICLGLFQATFSEDFSAVFKCASFLPIPCAHLFLYCWAANNVTVQAEAVSAAAYGCSWVGASERFKRALRIIVSRAQKPLVLTAGHLYAIDREAFLTLVNASYSYYALLSQMNNR

>LmigOR115

PLLQHKAADEFSGAEKVERQMPIPSWFPFDVQRSPAYEVVYAVQAVCGTAAVQLSMLLDASFYQLALLLTAELRVLNDNLALVGAATSATGGTGVAGRDSHHQHMPAPKLTTSAVTEDNDRSRTLLYFEFVENVRHHQAIMKCFQLLESVLNYSISILLLTNILTMCFSIFFASVMLQADGGLRRAMKITSSIPNLLIETGMFCIFGQMVVDQSERLPQSAYSCSWVDSDARFKRALLIFVLRTSQPLEFTVGKLIKLSRETFLKILNSSYTLISLLYQFQESND

>LmigOR113

MDITEEELEAVVPCMARSGLLGYWRSSASREGAGSYLRGFLSCCLITCISLSAAERLLTDTPSDLAELTMTAFELTVPLTVVSKGLFFILQRDTIHELVDLLVDMRRRYAERDDGPNRRRACYLYVLAVQRVLLVMALLIIGGWLAGPMLPHVFSFASQNESSVPWQTPLPLWLPVDLQRSPLYEALYLFQGLCVLTSLTSASALDACFCNMMLMIAAELQVLNDNISSPSGNETVVDKGESESITLEVHSELESVVPQFKSGATGDAGLSKTGRSRPRNQTSLRL

>LmigOR14

EGLPLQLVLPFDTSRPAGWAAGWLFCAFLTVHCVVMNMMADAFNVSLMAQLRTQLILLTRHLTDLAGDVQSEGPSLPQTPRKVVEQPVKFYTPVYRHYSLEGTSSHYQKTNPSVVDAEVQRSQHSYEPSNSVDYRLQKIILHHQTIIRNVDRLQQCLGGVLLAQSLSLGSAICMLLFQVALSAQGAQETGKICGYLCAMFTELSVYCWFGDQLMSESEKVAFAAYDAVTSLQECPISMKRSLLLMMHRAQRPLCITAAGFYPLSREAFVSILNVSYSFFAILRNFKEE

>LmigOR111

LASRLFSSDDVVEETSSPLKTPLPTWTPAAAQRSPAYEALYLFEALCLTASSQALLCIDVFFIDLMLLVAAELRVLNDNVAAVSAGAARSDSREDTAGHGSGVSTVQQRSREFPDTFSAFDVLVDRRMSEDMYRQLVGNIRHHQMIIECVELLQMTMTYSIFALLFFNMTSICLNIFVTASLLQSDADLVTAMKAVFTTPVFLYESAMYCIFGQMIIDQSEQLPLSAFNCGWPETHTRLQRALLVFMLRSSQPLRIQVGKTYELSKETFVRVLNGSYALFNMLYTFQGNK

>LmigOR30

RRLTLAVTLLGCPSPGVWTAVPLLAPLVGDGAAPRNRSLPAAARYTARDTESPRFEALTALQFFSMQFSYFTTVGVDMLVVSIMIHASAQLELLNLSFGRLGQAAGSLPGNRERRRAEVCVREAADGPGRSGISRRADAEETPPREKFCQELRDCIRHHQDVIQLVADVERLLTSMILTQVLGATLIICVALFQFATNIENIGTILRVSVYMSFMVNEVFMYCWFAHNIIDQSSRIAESAYSCAWPGVPPSLQRSLLVVICRAQRPLALTAGKFYQVSRETFIQLINASYTYYALLRQMND

>LmigOR89

AYAGCVVSALLFLVASQLSAMLHFWGDLLSATNNACVTSSYSMAVFKFFAFRAMRPSVEHLVRDLDRCLQVYGREYAAEKEAGFGACERQARLVSRLQVSMGVSVYLSWVVLPAIRAQACRSTDCRVHAGFPALVWYPFAFTEPPVYPVIYAVLSIGLFYGCIIFTSQDGFFWSLIIYVGAHLRFLNFMVANMSSSEADAKIPRGSPEMEENMRSRLKECVRYHNDIDRCVQRLSSLLGPVMLGQFLTDIVTISASAFVATMLKADSGWLLKYGSYLSGTIEHMFLFCWFGNDILTESARLQLSAY

>LmigOR28

AARGVTSYYVGVGGAAVVVWNLMPLLLEAGRSLPTIAWYPYDETKTPFFEVTYLLQGISTFYCCITNVGLNVFLVSLMIYISDELKNLNDSISSINYRSIYNCCCNGDKQIGLLNWSSKIVTDDCDINVDTHIAHSKHIRQRAEFCCVVKAQDYLRMCLQYHQELIRTVKKLETTITAIVFIEFVAGIIVTCLTLFQAAVNAGNMALFVKFIMYLLYMTVGMFIYCWYGQDLMQKSEDIKWAAYSCNWQGAPKSFTDLLKIVILWAQQPLTLSAGKYYKISLKTFVTLLNASYSYYAVLRQMSDTQK

>LmigOR80

MQTGEVEDADGHLSWEETARSVLRLNIRHLHLFGLWPLRASRLFPLYTAYAAALGVWNTAEGFLAVYFSWGDLEQTTLVLMNTFTNASGLAKICFFARDRRRYASLARGADRLARLQAQACARDRALAQVLRRARRAARRLTAAMLLFMFSQCFVWFPMPAVAHADERRLPFAQHPWDNNTAHYELAYAVQCVAGLWLSQISYGVDCLFASVMLLAAAQLEILAGRVAALGERRNGRGGEPLPADAPDSMYADLCACVEAHQKILRFVSDLENTMSPIAMTQFVCSVLVACSSLFQATYSKDLSAAFSSMSFLPIPGGQVYLYCWAAHEVTEKAQMVSAAAYGCSWVGASERFKRALRIIVSRAQKPLVLTAGHLYPIDREAFLSLVNASYSYYALLGQMNKR

>LmigOR73

MKKSGMEDLGRTVSWSESGRSVLRLNIRHLWLFGVWPLWDSPLFILYTGYGLSLGVWNVVEGALAASCTWGDMEQTTLALMATSTNCNGLVKMAFFLRDRRRYQALVRRVAALVALQGESCAADPLLGRVQRGSRRRAFRLTAAMLLFMFSQCFVWFPMPAVAHADEGPLPFAPDNTTAYGLSYLAQCAAGLWMTQVSFGMDCLFASVMVLLAAQLQIVAGRIARLGEQNYDFAFSFVGHLQDTMSPIAMTQFAFSVLVVCFGLFQSTYAEAVTMAAYSCSWVEASGRFKRALRILISRAQKPLILSAGHLYPIDREAFLSVRS

>LmigOR101

PGSTSAAGLCRERVDDLEEMSEGIFICTTVIVSTARMLYFLAYRMRLQRLASLLLEARRCFPVQGAALRSRYQRHAANVCIGFQATAVLPVSLWVLDPLLTAAVTSQNTNNASADAAAEASRLPLSLWLPVDGRQSPSYEAVYAFEGFLVVFTAQVLLFLDMLFIVLIIHITAELNVLNDSVAAIREAVAGGGETGNGTDASGKLDNTYGSVSRSDSDMYGQLVEAIRHHQTVMRYVQELEDFMSQPLYILLFTNMMNMCLHMFTFVVLLQKDIERSSMVKMMLTFPAYLYQTGTYCIFGQTIIDQLLKDTYTMFNMLYTLQGNK

>LmigOR9

KLSLSTLRPRRYRRLMDHVTRRLAAHLRHDRAGRLRLRRDSRAAHAFTMGFVVCGHVTVASWSLLPLLLKPADKLRLPLVAWTPFDSSHGTGFLVTYIYQFTCTLFMAWTSGATDLICVNVVMQLCSHLDILCSHLERVGRRCCREGDGGRCAHGHLDDGDQRPAPGRDDLCGQLRGCIRYHQDIIRCRVAREMDSMLWTIVLSQCLCGMGVLCLLLFQTAMYTLTIETIAKYLSYMASILLQIFCYCWFGDNLSSKSTAVARTAYNCDWTRGSSAFGRSLCILMARAHRPLTVTGGSFYVLSREAFIRILNASYSYFAVLYTMSDE

>LmigOR59

MSWGSEEVEQLTWKYSAKSVLKWNIRILNLVGLWPLTESLLFRSSTAVIVMLCVAHIAEAAVNLGTLRGGLQDFTLALSNVSVVCVGVLKLTFFLRHERSYCRLVRSLDVLVDSQREFVQGQPPLAALFEATQKRTVRVTVGFLVYAITQLVAWSFAPMIAAPGTWRLPFQQLPLTDETAFLIYELSYAMQVVSIIFIALINCQMDCFFMATMLHTAAQLRILSARIMCLKLQNEDIPAVFRSNEGEEVQASATHDSTYRNLCLCIKTHQELVRFVRHLDQVMSPIAMMQLGLGVFNGCMLIFPAAYSAESDALVKCLAAVPTISTQLLLY

>LmigOR6

MDVSFAAVSVMRAGLPAEESGGNRRARRVTQWSLALWLGTTCSWMLAALLRLDLPFFAWFPFDTTHHYAEAFIYQLVTANLVVVIISGLDCFCLELMMHLSERLQTLNKLFRSFATDNARQQPQLRMQPSTPASHRGKSPYGIITKKSFMKWVVAPLDVHRKPAKAINSLYSRHDSPNVGNANGSFKHCIQYHWELIELKKETEKFCGVVLFFQILASMFIICFVTFQATVNTMDAGSLTKCVMYLSVALLQLGLFCNEGTNIVTQSEELMLAVYSSEWPDCDAALKQSVIVTMMRLQYPLQIRAASYCTLSFETFSKILHTSYTFFTLLRQVSETQ

>LmigOR56

MDQRATSQSEEEEWAGQSVVRTNTHLLRLLGLWRPARSRLYDAYTAAVLAVGVADLALASAGLWLRPGGLAEVTLGLANLFVILTALSKSVLLLGRRPLFYELVRRVDGATAAQRPFCGEDPLLARLSADARARADRLSRAMHWYVVFAALSWSAVPLLAPPGDRVWPFQQLPPRPWARSPLYEASYALQVAGTTYFALINMDSDCFFMAVMTHVSLQFRILASRFAKLNSTEESLADKKASDVRVTSSESTLPVDDTDRELRACIQTHQKLLRLVNFLNDVMSPMAMMQLALGVINSCMVLFPATYSEDSSDVMKCWGALPLLAIQVFLYCSGAQRLADQLVQATYSYYTLLQHFNSH

>LmigOR91

GGSALAVHAYYCTVLLANTAKTLVFAACRHSLGRAIHVLSRCPRAERSEKSGASARLTFALPQVMVLLAVATHSLVPLLGAGDGACADPEATGQGGGGCFSGRFPLELWYPAAALATPLYQVVYALQLVAIYCTCHTAINVDLFFFAVTNHASSHLQELNDALCRMGVRHTPVNRRRGSDESDHSADGKHGNGPLRHTSEESLVRAEHQRARYQELVRLIRVHQTITRAIKELEPVISYALFGPILTNVLNICLHMLVLTTERDNMGTNSKAFVGILFNLIQNGLYCSFGETLTHQSDRLFISIYSSGWENGCRRFKKAAAILMFQTRKPVQIKVAKLYTLSRRTFLQLLNNSYGLFNLLYQVKNPE

>LmigOR86

MAKYSVRGGGGGAPALDLRLQLRLLRLAGAWGPSPSASSTSLPYAGYSAAVVLLLLAFVGSQVSAMLHFWGDILSVTTNACVTFTYTMAMFKLLVVLIMRPSAEYLIQELNRCMQEYGRDLSSEKAAVLARCGRLSRHVAAAHVAIGAVAYVCGVALPAARGRLCPSAACRAADGFPVLVWYPFPFTVSPAYELVFAAVSLDFFYGYILSTTLDGFFVTLIIYVSGQLRLLNLMAQNVCAGAGGGGSEASEQRVRDRLAQCVRYHTDVDRCVQRLSALLGPILLGQVLADVVTISATAFVTTTGKTDSGWVFKYGSYLAAIAEQLLLYCWFGNDVLTESERLQLSAYSSQWVSAPARFRKGLLVFLCRAHRPLRLTASKFYTISRETFLLLMNASFSYYAVLRQLNSD

>LmigOR53

MSAEPAQTWRSSASSVVSYNVRLLFLCGLWPLRRGRAFSAFTAAVLVAAALHAAGAFVGLCTEPGGLQEVTLALANLFVVCSAIVKSCFFLADRTRFCTLVSTLDRLVQVSGQQSAVGAGLRSRLSASARRAVRLTLAFHLYVLSALVGWCLMPALKRQRRLPFQQLRWLDTSSAAVYGASYALQCFATFFCSFINTHLDVFFMAVMIHVADQFAILAARFADLRLDADADPEGRLPQGERAVLAEDAYQQLRLCVRSHQELVRLVQLLDDVMSPIAMTQFVVGAINACMVLFPATYSTDVGAVLKCWAALPMVGIQIYLYCSGAHDIMEEAGAVSGAAYSCSWLGADRRRRRALLLVTCRAQRPLQLTA

>LmigOR114

ATSAGKLCLDTPQELAAVADYGYAVFHLSAVTVKVACFILQRSTIEELVNLLDETRKTYGKTEANYQVRQLYQRRATNIYRVLQALAVAVLCMWISSPVIQRQGLKEGERPPPNPIWMPDNSPGYEIVYSVQSLCGSAAVQASMLIDTSFYKLTLMVTAELQILNDNLARLGRAAEAADRKGTAAKQDGKEVAVPAIKQTSAPVTEDNDQLLNDQMVDNVRHHQAIIKCFDLLQSVITYSVSIVLLTNILTVCFSIFVVYVLLQSDGGLKSASKTIIGIPSVLGETGMFCIFGQMVINQSERLRSSAYSCGWPDADGRFKRALLILVLRTSQPLQFTVGKLIHLSNETFLQILNCSYTLINVLYQFQGSKE

>LmigOR99

MNAYVCAMGALLSVKVAAFLWHRERLWQLACQLVSCWRQFEDADGGVRDMYRSQAARVVRYMQVMAAIPAMMWILEPLFSGGDEQSQGRSLPLPTWLPLTLQQSPTYEILYVLQVLIIIIAVAVSVYVNIFFAVLMLSIAAELHVLNNNMMAMGQCRDCEVPVRYEREEGRSRDQVTSSFLRTHRRQRVPKAAANKSLPSPVVVHMHKGSYDRMYHQLVKNIRHHQVILRSVEELQKAMTHSIFVLLFLNIFNICVVIFAGTTLLQKQADQVAMYKMLCSIPIYMYETGFFCVVGQTIIDQGERLSMSAFASTWLDGPRRLHRLLLVFMLRCARPPTITVGKTYTLSKRTFVRILNGSYTMFNMLYQFQRNK

>LmigOR93

MRLRAALLAAAHLVLPVTLSVNFLSCRFDSLDLLATNIFLLSGMAGVSSKALLFVLDRERFELLLLRLQRTRMRFPDNSGARERRRRMATRVYYAQHGSAQLVVLLWVSVPGVTALVSGEGRELPMPLCPPATAADVHLSPCFELIYALQAACLFVAVEGMISLDSSYLTLMLNIATELEVLNDNLVSIRSGRLPEDKKCSQLSDQSPSNPAHEDDMYFQLVENIKHHQHIISRAQELESVMSGPTFVHLFYSLVCISLSIVSVTVLLQTEGYTPKILKITFVIVVFVSQLGFFCILGNNVIEQSERLLVSAYSSYWPGAQPRFQRALLVLMLRARHPLHISVAKLYPLSKETYLQILNASYTLFNLVFQTNGRN

>LmigOR38

MKEIRNLTISNANNSMKVRHAYRLGINSEGDRSDDGLGRDRNQSFDNRAFIISVQPSESSVGAFLRKRFIVKYLPKVLSTVHRLARSITLAMLVFGAASSIGWILNSLLLGGNSMLPMVAWYGIDQASSPTYEMLYVSQSLVIFYCFLTSWGLDLLLASVMIHIAALLKTVCIHFSLVTAVSSNRTPGMQMLQRNKTLPTAGNNLETVVLEMTSTDYKYLQCVKCIQDHQRVIAMVKDLEDLANPVILTQFVAGIVVICVNLYHTTTDTHGFLWASKFASYLLMLVFQIFIYCWCAHNIMEQNLLLAEWTWENGWRLWRAAVDLSPGAGGGGRVNGYACTLLFVSVAGIGLFSSSLSSFRKLINTSYSFYAVLRQLNSR

>LmigOR43

ERRPRLYALYTASVLASQAACIAMGLRHALDSWPDTDAVMLTFVNTATLLGGVAKLAHLCAHVRDYRRLVAALRGLVAAQWPACRRDPRLLAAFRRSYRRALRLTFGMIAYLHFIGPIWYAMPLVARATGGEQRQLPFVDLRGAVKEDLSLYVSVYLLQCHAIFFWCFVSPSLDMFFVTCMLHVAAQLGILNARLSELGGVRVPDGGEMVALPAGRRKSRNLEQHSDDSDISEELRDCVKIHQDILSFLQDMQRVMSKVAMAQFVCSSVSICITLFQATCNPEGNSNLKCFMYLPMPAFQIFIYCYGGHELIDQGLAVSLAAYSCAWVGATRRVTSSLHIMMCRAQKPLTLTAGKLYPINRITFVSLLNASYSFYALLRQTRDR

>LmigOR15

RLLAVAAVSYANGCDVVAVCREGITDFDRFTITLSVFDTGNTWVYRRCHVAWHERDFQKLAQQVRDDFGEFMAPSDVPVLRGLAASLRRFVTAYVLMGAFDTVVWLTHPTRGEGLPLLVLLPFQTQRGLGWLSGWLFCAYITADCLTVNFMVDSLNVCLMEQLRMQLLILRKHIAELGNRSESTKYSLSEKESESAKEVGKPQQGKLAFVNETHKGSQVSSEAANSSDIHSRLRGIILHHQAILRNAEALQKCLGNMLLVQSLSLGSVICILLVQIALSAQGARETGKICGYLFAIFGELWLYCWFGDKLTSEGENLTLAVYDAVTSLQESPTSIKRSLLLLMLRSQKPLCITAAGFFPLSRESFVSILNISYSCFTVLRNFKEE

>LmigOR23

DLDHLLRPLHWTATLRHPQSAHSSPLLFRLCKLVIITIIFSFFCSEATVLFRVGAGDLDVFTITIGVADTNIIWFCRMVHISVCERAFHKLALQVGQDFAEFLSWEDLPLLRAQSGAVRRFTRLYVWFGVGCVVYYLFSPASDAGLPVILALPYDMHRPLAYALTWLYVSVTTFHVVVMTMVFDSFNVSLMAQLRTQLSLLSRKVVSLAKEMSEKPVHSPETSAYRELHSRLEKCVRHHQAIINNSDLLERSIGPMLLAQCLAIGACACFQMFQVATNTNGLQETGKYGAHLVVMLAELFEYCWFGEGLITESENMALAAYDAVTSLQDCPISIKRSLLLMLQRAQRPLRITAGGFFPLSRESFVSVVNVSYSFFAILRNFKNEEE

>LmigOR17

MGHLLTPLHWTGVLRHPRYSHQSPLLFRLYTAAVSSFALCFICSEAAALVHDDTGDMDVIILLISTINTASIWIIRMVHIAVFERDFHKLAVQVGHDFAEFLTWEDIPVMRAKSRRVRRFTLGVVWFGVSACSYFLVSPVSPEGLPFILALPFDATTPLGFAVSWLFCTITCMHAVVMTMALDSFNVSLIAQLRIQLTLLSNKIVSLAREMSQRPIYSPETSSYHELHSRLEKCVRHHQTIIRNVDLLERRLGSILLAQSISIGAVACFQMFQIATSANGVQQVGKFGCYLTTMLTELFVYCWFGDDLITESEKLALAAYEALASLQGCPMPITRSLLLLMQRAQRPLCITAGGFFPLSRESYVAVLNVSYSFFAILRNFKEEEQQPD

>LmigOR58

MGWNPREEKPLTWKETANSILRFDVRILYVVGVWAMPVTKLFRAYTAFTLVLAVGYSVEAVIHIWLVRNSMEEVTLAVSTYAVVVTSACKLVSFLQHEPGYWRLVRWMDAVVADQRHFCEERPELRAIFDEARKRAKRYPNALRVYNTSLIISWVFIPLLAPPGLRPLPFQQIPLSETEDFPLFLFSYLLQTFGMLFMCLVSGCLDSFFTAVMIYTAAQFRILGLRIAALRQDNDEIKRQARSDVYEKPRKGAATDHVYEELRLCIRTHQEITSFVTHLESVMNPIAALQLITGVINGCLMIFPTAASSESGALLKCIACVPTISAQVLIYCLGAHAVMEQSEAVSAAAYGCAWPDTSPRCRRSLLVLMTRAMKPLTLTAGGIYTIERSTFLSLLNAGYSYYALLKNFNSR

>LmigOR42

MRGRARLFALYTAALYACFAAVLLMALQLAYLSQRDINELTYALIVVMSHVGVLFKMTHFLTSRGAYLQLVERLNRLVGQSLADDSSGPVLAACHRKAMRLTFCSFAYLALTGAIWYLVPIVDAIRSGSEGRRLPVANPHWIDTSKTALYAFLYVVQFPSIFYFVGISVGLDGFFATTMIHVATQLRLLSLRLSGLNHGGSRISSVTKFSEDNKSDLPSPLRVREIERLDESEGMYQQLVQEIKRHQEIVSFVKFLEAVMSPVAFVQFLFSVGSICVTLFQSTFNPRPDVVLKCAMYLPTPAFQIYIYCWCGHDIMEEGARVSLAAYSCAWTGASKRSKDALRMLACSTQRPLLLSAGKIYPVSKATFLSMINASYSLFAVLQQMRSR

>LmigOR37

MDAREGKISSVQSQCKPGGIIPVVELVIPDAMKRTGCGLLQADALCDYFRLVTELDAFVSEQRSKYYNNEKVIEMLDASSKRTASITKAVMAYLICWTFIVVPALFLIESPYSVLPLMAWYPFTANIWPRYEIIIMLHFLTIGYCFFTSWGMDLFFGCLMYHLSLQLRLLNYHLANIRYRCKSECVLQEGFEGKTDFSTPRVELEVIRNGREKQQVAEYTRSAEDAIYVDLLQCIKHHQRIIRYADNVENVANPVILSQFVLSVLVLCVVLFQTSSELGTLTALVRFLVYLLELLLQIFIYCWVAHQIFEECDSLLQLSFVVKTLDGRRAIRTRSVKCLLQESFWSVMIMPRPVTFSAGKIYAIDRTTFVSIVNASYSYYAVLRQINN

>LmigOR10

MELAHLLLRLLHWSGALRHPSKPCGCYLLYSAAVVTIIGLFVVTQVAAVVDRQAGSDLDQATLALCVASTMCVGMCKILNILRSEHLFLKLADEVSRQPDGLSAWEAGAWLWSRGRVRRLSAVYLSLAASMAVTWPAAPLAVGGGALPFVARFPFDVAAPAGYAAAFAFQVLVVAVVGVVVPCTDLFTVSCVEHLNSLLHILVHRVERLNALETGARPQHATADDKAAAVADPLHRDLADCVALHQRIISEAECLNQAIGGVLLLQVVASSAGICFLLFQVAKKTTFHLVETGKLLGYLTFMLSQLLFYCWFGDDMLSKSESVSLASYRCRWPDAPTRFQRSLLMVSMRAHRPLTLRAGKFFVFSRQAFVQVMNVSYSYFTVLRSLSEA

>LmigOR21

AAAAEASDMGHLLTPLHWTGVLRHPRYSHQSPLLFRLYTAAVSSFALCFICSEAAALVHDDTGDMDVIILLISTINTASIWIIRMVHIAVFERDFHKLAVQVGHDFAEFLTWDDIPLLTSQSRVVRRFSKLYMWGGVGACAYFLVSPVCPEGLPYILALPFDAMQPLGFAVTWVFCSVVTLHAVVMTMVLDSFNVSVIAQLRIQLKLLSTKIVNLSKEILNTDVDSSEANVYQELYYRLEKCIRHHEAIIKNADLLERSLGTMLLAQSISIGASTCFQMFQLATRANGLQQAGKFGCYLFAMLAELFVYCWFGDDLITESENVALAAYEAVTSLQGCPLSMKKSLLLVMHRAQRPLRVTAGGFFPLSRESFVSVVNVSYSFFAILRNFKMEEQ

>LmigOR116

EPADDLLDLTWTVFILFGGAIVFVKMVCFVHQRAVMQEAFQLLLACRNCHYGGDSIRTIRGSYQRLGNITYFSIQVMVIVAWMIIVFMPLLNHRISAAGREPTAKEHGPLPIWLPFEVHNSPFYEFTYAFQALWLAFVAETSICVDCIFVNLMLMITAEIHILNSTLSTLQEHSIINKVPVVTTNQSTEDILPFTHSSRIHDSKQLFEKLISIEGTDYSPSDMCDISCAESQKIIKQQIYHQLLQNVQHHQTVIKCTSSAQKAMNFSVFVLLSTNIIEICSSIFGTVELLKNDMPAAAMKTLCVIPIILSQSGMYCFFGQMISDESEKLLQSAYNCDWYEGDIHFQRVLFILMLRATGPLKLKVGKTMSLSRQTFLQVLNGAYALLNMAYHVSK

>LmigOR76

MNTIDEAVRANRLRWWGHVTRMGEAMLSKRLMSSAAEGPWLETSCQHWCRSRLFGAYTLFAFSLGLWQTAESLLSCYFSRGRMEQATMVLMTTFNIGGSTVKMALLALQRRRYFSLVRRTDLLVTEGAPPADCSKERPRSLPGPVAAAQXAAHGRHAAADRGAHPWDNNRRFYALSYALQCMGAAWTTQASFGVDCLFVTVLMLVVAQLEALASRVRAIRVEPEVGGGAADKAADQMYAELCACVDLHRKILSFVKQLESTMSPIVITQFAISVLVACVSLFPATYSTNFTDVLLCAGFLPVPLGELYLYCWAAHNLTQQAEAVSAAAYSCSWVEASERFKRALRIIISRAQKPLVLTAGRLYPINRAAFVSLVNASYSYYALLGQINSRSTETTH

>LmigOR20

MPPSLPPEAAAASSDLGYLLTLLHWTAVLRHPRFIGTSPFWFRVYTLTLLTIDASFVFSVFIVLFREGTEDLDVFTLTLSVADTNGTWLFRLAHTVACEAAFHKLSQQVGHDFAEFLTWEDIPVMRAKSRRVRRFTLGVVWFGVSACSYFLVSPVSPEGLPFILALPFDATTPLGFAVSWLFCTITCMHAVVMTMALDSFNVSLISQLRVQLMLLNSKLVTLAKEESENSKLSSKTTDYRELHYRLVECIRHHQAIIKNADLLESSLGAMLLGQSISIGASACFQMFQCVTSGNGLQQTGKYGCYLALMLAELFVYCYFGDDLITESENLALAAYDAATRLQGCPLSIQRLLLLLMQRAQRPLRITAGGFFSLSRESFVSVVNVSYSFSAILRNFKE

>LmigOR67

MEDKLSWTFSGNSALKLNIRHLWLLGTWKLGESRLFKMQSTVAFGLSIWSTVECILAVYFIWGDLEQTTLVLLITCTCSSGVVKMFIFVYDRRRYDSLTLRLDALLSLQTGPCSEDPALAAISDWSRKKASRLTMGLLLFMLSQSMVWYFVPLIAHPEERSLPFVQHQWDNNSLYELSYGVQCLSAVWISQISFSVDCLFASVMILVAAQLEILGQRLINLKNGRDSAEKEEKKQLDSKTGESMYDDLCLCIETHQEILRFVTQLQDTMSPIAMTQFALSVVIACMALFQATFSEDFSAVLKCASFLPIPGGQVYLYCWAATNVTEQAEAVSAAAYSCSWVDASERFKRSLRIIISRSQKPLVLTAGHLYPINREAFLTLVNASYSYYALLSQMNNR

>LmigOR68

MEDKLSWTFSGNSALKLNIRHLWLLGTWKLGESRLFKMQSTVAFGLSIWSTVECVLAVYFIWGDLEQTTLVLLITFTCGAGVVKMFIFVTTGRRYDSLTLRLDALLSLQTGPCSEDPALPAIADWSRRKASRLTMGLLLFMLSQSMVWYFVPLIAHPEERSLPFVQHPWDDGGLFGLAYGVQCLSAAYVSQISFGVDCLFAAVMILVATQLEILGQRLVNLRNGRRVAGRREKEQLARKTGESMYDDLRLCVETHQEILRFMTQLQDTMSPMAMTQFAVSVVIVCMALFQATFSEDISAVLKCVLFLPIPTGQVYLYCWAANNVTEQAEAVSAAAYSCNWVDASERFKQSMRIILSRSQKPLVLRAGRLYPINREAFLSLVNASYSYYTLLSQMNDR

>LmigOR27

MAAAAPESSGGDVEYLLRLLHWSGTLRHPRAGRGASLAFYARNAAVATAVLLFVLSQAAVILGEGPADLDRFTRALCFFNTSLTWLLRLAHVALREKQFHAIALQVGSDFGEFLTPRDAETLGRRGRALRRFVLAYLCFGVAAGAGWDVFPVVRHGVCGDGLPFHMALPYRVDRPLPFAATWFYCFCMTMHVAVVTMVFDSFNVSLMAQLRQQLSVLSGNIRSLADEQRRTASSGVDSPEHTERVRYRIRTIVRHHQAIIRNVESLEKCLGDMLLGQSLSIGASICFQLFQSAESLAEAGKFGCYLSVMLAQLFVYCWFGDDLITESEKVSAAVYSVVPSLQGCPTSVKRSLLLVMRRAQRPLRLTAGGFFDLSRESFVQVLNVSYSFFAILRNFKEE

>LmigOR82

SPSGGRRRAGGGARDRLVECIRFHQEIIKSAAEMESILSASVLMQFLTSTLVICFTAFTVITAETAYLHTYITYLATMFYQLFLYCWFGGELLLESEKLQLSAYSCAWPDADASMKRSLRIMMCRLQSPVKLTACRLYALTRETFLLLMNGSYSYFALLHRMNQAPE

>LmigOR65

MEHPYLAAVTPCRVMKESGGGQRAGADDTASLTWRQTAGSVLKVNVRGLALFGSWPLPESWLYHAFFAVVFASNLGNMAEAAVGLWLGRGGLEEITLVLPNTLTTAAGVCKMVFFYRDRGRYYRLVRRTDLLAGSQLAASGRHGADAVRQADRQSLQLTYTVFAFISLQIIVWFPMPLYAYRDQRKLPFVQLPWNEDKDIPVYELSYALQCFSSFTIIFITLGMDCLFAVIMIHVAAQFEILIVRIRNLRLDLQTTGVTQSKASLGQLSRNDVSSINVQTDYKEVSHHQQQINEAHDKLYSELCHCVESHQEIIRFVRHLETMMSPIAMTQFVFSVLVACVALYQATYSDDLSAVFRCAGFLPVPGAQVYLYCWAAHHVMEQSEAVSAAAYACPWIEA

>LmigOR109

VNIAANISLVITSGVELCADKPRQTERASVTAFLFSVSIINFVKAVSLLRHRSRLRRLVRRLVAVRAAFADPAGTRGRYARHAALLASTWLVTAETNVAFWCLDPLISEAAGGAAAERQLPLPLWLPFNQSRPHSYGRLFALEAAVLMSAVQIAILVDALFVTLIINVTAEIHVLNSNIRSMSKAAASGGGLQSGGTIENTTTSDESTLVANLHRSNVRSSNSAISDISASGRNGKSADDEMYGLLVKNIQHHQLIIICVKELEKAVSTGTFALLSINILNLCSHIFSLVVMLEGENSVSAITKMLVAVPVFMCQSGLYCLTGQAIIDESARLSTSAFSCGWPDADQRFKRSLRLFMTRAAQPLHIRVGTLISLSRATFQELLKGSYQLFNVVYQVHTN

>LmigOR98

ATSRTCLVAGCYLALVAFAGSQLLAEPWRPLEELAIAALIFVTSASFTFKFAMFLRYRPRLLKLATLLCGGGLRPAEGEETRRRYRERGRQVFLYLQAILSVPTLLWAMQPLLVPPGGSAANLRNATAPVRMRSTPLPMWLPPALQQSPAYELVYALQVVSMLIVLQTSVFTGVFFLVLMLSIAAELHVLNDSISGNRSKPSSSSGRRCSSLSMEIHCPSNGRLVNDSSVINFEGRLFRQQMYCDLVKNIRHHQLIIMCVKELEAIMNLPIFVLLFLHMVNICAQIFVTSLLLQKDNDSTTMFKVLFTLPIYLYETGLYCVFGQIIIDQSDRLPTSAYSGAWLQGDARFRRALLLLTSRASRPLTLTVGKTYTLSRHTFLQILNGSYSLFNMLYQVQGNK

>LmigOR57

MGWDSKEDQPLTWQYTADSVLKYDVRILHMIGVWPLSGSQLYRCVVTVIIALCLGHFVEAVINLYTLHGDLEDFTLALSNVSVVIVGILKVTFFLRHERGYCHLVRWLDTIVASQREYTRGRPHLEEAFAGAQTLAVRITRGFCMYNATVVLAWVLAPLAAPPEAKRLPFQQLPFGEGSPFSLYALSYAMQGVSMLLIALISVQMDCFFTAAMIHAASQLRILNSRLSDLQLGKAGLQLQGGTTLDSMYDELRLCIHTHQEITRFVEHLENVMNPIAMMQLAVGVFNGCMLIFPATYSAENDALVKCLAAAPTISAQLLLYCLGAHSVREQGEAVPLSAYSCGWADASAAFRRSLLVVMARAQKPLALTAGRIYPIQRATFLSLLNAGYSYYAVLRNFNSR

>LmigOR81

SRLYDAYSVWTILQLLMTGVGQMAGLQGHWDDLQTVFTSLCFDLTVTCTIIKGSIFVAQRGSLDALSRHLEANARQFCSHLPAERRALLERARNLSRVIVCSFQSVGGVTLVSFITGPLVQNGRDRELIASAPGNATLDRHLGHNYPMLMWWFGGLPVSSPGYEAAYLIMCYWLVLMYICTNVPDAYYVGLINYISAQLRLLHIALRQIAHPGADDALAEKLGHIYGLDVKGGSPHKDGAQDASRDSVYDRLVECIRFHQEIIKCVDEMESLLSLTVLIQFFTSTLVICLTAITVINTEAAYLPTYAAYLATMFYQLFIYCWYGGEVYLESESLQFSAYSCNWPYTDARFRKTLKICLARMQRPISLTACKFYKLSRETFLLLLNGSYSYFTLLLQMNQKDD

>LmigOR12

SSGLDLLQRLLHWSGTLRHPRAGRWSSLAFYPLNAAASAAIALFLCSQGAAIWREGARDLDRLTLVLSTFNTIATWLFRLGHIAVHEHQFHYLSFQMERDFKDFLNPRDVPLLQASNQAHRRFVLSYLWFGVVLCMVWTLFPVATFGAGPDGLPFIMALPYDVSPLHAFIPTWIFGAFITVHVTMMTIISDTFNVSLMAQLRFQLVVLNEKIINLTTDIETPKSPLTKEEYITTKSAYESVKHTNIHHRLRQNLLHHQVIIRNTEMLENCIGGILLAQCLSIGAAVSFQLFQVAVSTQSLVQAGKFSCYLTVVLVELFMYCWFGDDLITESENVALAAYTAVTSLQGFPAADRRSLLIAMTRAHRPLRITAGGLFPFCRESFVSIVNMSYSYFAILRNFKDD

>LmigOR22

MPPSLPPEAAAASSDLGYLLTLLHWTAVLRHPRFIGTSPFWFRVYTLTLLTIDASFVFSVFIVLFREGTEDLDVFTLTLSVADTNGTWLFRLAHTVACEAAFHKLSQQVGHDFAEFLTWDDIPLLTSQSRVVRRFSKLYMWGGVGACAYFLVSPVCPEGLPYILALPFDAMQPLGFAVTWVFCSVVTLHAVVMTMVLDSFNVSVIAQLRIQLKLLSTKIVNLSKEILNTDMESSEANVYQELYYRLEKCIRHHEAIIKNADLLERSLGTMLLAQSISIGASTCFQMFQLATRANGLQQAGKFGCYLFAMLAELFVYCWFGDDLITESENVALAAYEAVTSLQGCPLSIKRSLLLLMHRAQRPLRITARGFFPLCRESFVSVVNVSYSFFAILRNFKEEEQQPD

>LmigOR55

MVDRSVETPLTWRESAESVLRPNIRLLCSLGLWQPVDSMLFHAFTAAVLAVGVAHLAVAALGIWQRPADLAEVAIGLSNAFVIFTALSKAVLFLTRRPLFYSLARLVDQMTAEQKAFRAGDPSLQEVFSAARRSAGRLSVFFHWYVLVADVLWSLIPLVQASREKRWPFQQLPLDGWATSPAYQLSYGLQCASTLFFSLISVDVDCFFVAVMTHITAQLKILTFRFAAIGNRMYISDTSLNNQTASTKDASHEKLRGCVQTHQNILRLVSFLNVVMSPVAMMQLAVGVVSSCMVLFPAANSTDSAVVMKCWAALPVLGVQLFLYCSGAQRLIDQAEAVSGAVYSCAWPEAGGRVQRSLLVVVSRAQRPPELTAGRMFPINRPTFLSLVNATYSYYTVLKQVNSH

>LmigOR24

MAPAAASSSAAAAAAASASDLGHLLRPLHWAAVLRHPHSAAASPLFFRLCTVAMASFAFTSTCSEVTVLFRDGTADLDAFTLTLSVVDTNTIWLFRMAHTVACERAYHKLAHQVRNDFGEFLTLEDLPLLRGQSRVVRRFALAYIWFGVGACAYYLVSPVSAEGLPFILALPFDATRPLSFAATWLFCTVTCLHVVVMTMVLDSLNVSLIAQLRIQLTLLSGKIVGIAKEMSEKPVRSSETSLYSELHYRLEKCIRHHQTIIKNADLLERSLGAMLLAQSVAIGAAVCFQMFQIATSANGLQQTGKFCCYLFAMLAELYVYCWFGDDLITESENVAQAAYDAVTSLQECPVSIKRSLLLLMHRAQRPLRITAGGFFPLSRESFVSVVNVSYSFFAILRNFKDEEE

>LmigOR88

MVNVQKAGGGGRRLCTDLRLQQWLLFLVGAWAPHRGAPAICSLLYGVYSACVVLVLLLFVASLLFAMVHYWGHMLGVTMNACLMFVYLMNIVKIVSLLKMQPSAEEFIRELDRCMQEYGQSLEMEKAAVFQWTALKSRIVSVARMLVALSGCLYWAVVPAARAHACGGTVQCRDQVGLPAHVWYPFHFTHTPVYEVVYTVVAGGLLSGALISCIVDAFFVSLIIYQAAHLQLLNLMLAAVGTEKCYQPGPPTSPGSKRRLAEESAGAEQRMHRQLAECVSYHCHIDCCVQHLSSLVGPILLGQFLMDMVTISATAFVAIANNADSAWLLKYTSYLSSVVQQLLLYCWFGTDIITQSERLQLSAYSSQWVSASPRFGRELLVFLCRAHRPLRLTASKFYTISRETFLLLMNASVSYFAVLREISSK

>LmigOR119

MVVFGRGVEAVLGPSAKLLRLLGLWSPQKGDTSHNGSALTGYLTLALIFGLMVTSALKLLMDRPRELDELGACIFIVTMLAEVFFKMLCFVVQRPTLHKLVQLLTEIRADGSTGERNDEIRRGYQILVDRMFLLIMVTATATQTLWAAAPVIYQPLNEDGEVTRLLPLSMWLPLDMNASPNYEVIYLVQVLLMPLASASLLFDFVFIDLMVRIAAELEILDYSFSGLSKNPKSVSATSKNEFKSVHTVSDGEINLQLAKNVKHHQEILRSVDLLEEAMNTGVFIQFLASTIAISCNIFAATSVTLYDSQFKQLIIILLIQSGLYCIFGQVVTDQSEKLMHSAYSCEWVDCDTRFRRSLLTFSVGATRPIEFTVGRMYKLSRETFLQVLQGSYAMFNMLYTFQSNR

>LmigOR13

MWAPAAGLDYLLVSLHWAGVMRHPLAQPGSWSGRSFQLRKAAVAAALRGLPXXXXXXXXXXXXXXXXXXXXXXXXVSACLFRLGHISVHEHQLHYLAAQVERDFGAFLRPRDALLVQKRDRQLRRTLLGYIWFGVAGCAWWIAFPLLRFGFCAAGLPFILELPYEVTSAAAFVPTWLFCCLVTLHTAVLTIVVDSFNVSLMAQLHLQLRLLSRNLLALSDGDQDHDSATFKRVASGNEVSCGYANDIHIAHQLRKIILHHQAIIRNTELLEKCLGAMLLAQSLSIAAAMCFQLFQIALSAGSLAEAGKFGCYLSVMLAQLFVYCWFGDDLITESEKVSLAAYSAVTSLQGLPAPQKRSLLLMMVRGQRPLHITAGGFFPLCRESFVSIVNMSYSFFAILRNFKDE

>LmigOR124

MGLWQPRGRAAQRVNALLASLTLGSLCFMALCVTLKLCADTPQEIEQLTLCTLVASICVGFICKTALFVIQGDTLQQTVRLLEDTSEQFCTGDHNRLTRRRYLRLSNNVYYYCQMVAVPAAILTNTDDEEQQLPWQLPLPAWFPGDIYETPHFQILYVVHSFCVLVAVQSCLSIDIFFVHMMLMVAAELDVLNCNLAAMDHITVQTTRNEEERFIPRYKRNGRRLPLLNSGQSLAEQTLSQDTAHKDLNQQLLNNVLHHQAILRSVSLLQSAMNVSTFTLLFVNMANLCSSLFVAGVLLQKEGNVGKALNALFSIPALLYETIIYCIYGHIMTDQSERLVYSAFSSGWVNSDPGFKRSMLIFMMVTVRPMAITVGKTCRLSKQMLLQVLNGTYALLNMLYHVHRSE

>LmigOR51

MKKEKERRVQLEDLGSGEDLSRAEVESSVLRQNILLLHYMGLWPMGGSRAYRCFTAFNLSSSATIIVMNVVGVCFSLSDIDQVTGALSTILPMSGGLLNGLFLLHQRPTLCRMVRTVDRLVTSQAQFVERDARLVAIVGGARRRTLVVTLGVSGYLFAIASYWVVIAFTLPPSLRVLPFVQLPWMPSSDPGLFWSTFGAQLYTAPFCSYTTLFVEFFFLAVMLHLSAQFRVLGSRFASLGRSSASKVAADSGAVYEELRLCVETHQELLRFVRFVDNVMSPFAMLQFVAGTLAVCVVLFQAANNQDLNTNLKCAGWLPGPSLELYIYCGGAHEVVHAGEALVQAAYDCLWYNVAPRIGRAIRLVITRAQVPPVLTAGHLYPITRPTFVSLVNAAYSYYALLSQMQNK

>LmigOR60

MDWDPKETEPLTWQYTTHSVLKYDLRILHLLCLWPLPGSLFFRLLTAFLIALCLGHFVEGLVNLCTLSGDMEDYTLALSNISVVTIGTVKTAFFLRNERKYFRLVRWLDALVAAEKKSVSSRPLSEAIFPAAQKRSARVAAFLLLYNCFLLFIWLTAPLAARPEARILPLQQLPLTDSNAYPLYELSYAMQALSIFFIGLINVHLDCFFTVAMIQTAALLKSLASRLADLQVRNAPSRRNVDEGRKNIVTADDMYRELCLCIRTHQEITRFVQHLENVMNPIAMMQLALGVFDGCMLIFPAAYSSETSALVKCFGAAPTVCMQLLLYCLGAHSVREQGESVSVAAYSSGWADASARFRRAVQVVITRAQKPLVLTAGGIYPIQRATFLSLLNAGYSYYALLQNFNGR

>LmigOR11

MGNKVRVYLNEGNLPREHGSSLGEGGRGLKLKGGMGGEGDLLEDMGTLEGIDVLENSGHKADYMLDGGRGSEGVAGVQRRVAEMDGDGRSSGSVHLRWCCRQVERDFAEFLSPEDVPLLRASGRRLRRVVRAYLWFGAAGCMWWLLYPVACFGLTVQGVPYQMLLPYDVARPAVFAANWLFCTLPTLHVAVMTMASDSYSVSLMVQLRLQLQVLGKNLVALARGAEANHQKKCVKHGPSAEGLVRRSQLEDAIRQNIRHHQTIIRNTELLEKSMGAILLAQCLSIGATVCIQLYQIAVHAQGLVDAGKFGCYLFIMLAQLFVYCWFGDDFITESLKVSTAAYDAVTSLEGSSCSTKRSLVLMMLRAQRPLRITAAGFFPLSRESFVAVVNMSYSFFAILRNFKDEMNS

>LmigOR121

RGRLARLLGGVVRAATMFGLVFMWLGTVLKLCVDPPPQLEQLTLCSLVSSICTGFIIKAAFFLAFGGTLRQTVRLLADTRARFCTGDHNEATRRRYHKQSNNIYYFIQIVAAIAIVGWILCPLVTHILAKTDEDHPEARMQLPVPVWLPGDIHETPVFEMLYAFQSFTITFGAQFCLSIDIFFIHMMLMLAAELEVLNYNLSAMGHVNLPKLGSHGGKSISRYKSSGRQSALLSSGQQLGEQILTEDSGNEWLHQQLVKNVLHHKAILRSVSLLESSMTVSIFGLLFINMANLCSSMFVASKLLQKEGSIGKALNALLTVPSELYETCIYCIYGNVMTDQSERLLESAFHSDWVNGDTRFKRSLIIFMAVTRRPIVITVGKTCKLSKETLLQVLNGTYALLNMLFNIH

>LmigOR29

PPAAGLLYRAYTAAVVGLLCHITLPEAAGLVHFRGELRTATEVACLLFAFATTCYKLLAVLLRRRRILRFVHDLDARVAAMAEESAEARAAVSRRDRWTRRLAVLMVLQSTSTAFTWSMNSLRLSLSKGCSLRFLPIISWYPYDMTVWSNYAITYIIQFFTLIASAFSNRTCDILIITLMCQVGSLLEMLNLRFLEISRDSQSKADKRRTEWNQKHIMKRATSVLCKTIGANGLLDHVPTTSEEVPQDEMYAKLTRCIKAHQEIIRYAKELESLVNDIFLVDFLCCMIVICSTLYISTSASSNFGDLMAHFGYLVAMTYPLLFYCLFAHDIMEQSGRVAVSAYCLPWFLGNTKYRRAVCVALCRSQRPLTLTAGKFSVVSRATFLAIMNASYSYYQILREINEVKRSE

>LmigOR35

MAVDAPWSDTALWLNARLLALGGMWRPPWCPARCYLLYRAWVFFTLFSFFVAQIQALWHFWGDMDKITHDVCLMISIILSITKFFIFNFKEREVFRLVRRIDDTRAEQIETGDSEITSILDASYRSARGVALMMTCLGGSIPGVWAVIPILMRRLGIFPPERELPGTSWYTGRDGETPIYETLYVLQYFSMQNSFFTAVGPDLLFVAFIIHAAGQLEVLNARLRRVGGASDARKLQKAREEEEESGEAGCGELAWRELCGCIRHHQHVIGLIKEIERMVSKIVLLQFLGATVIICVTLYQSSKHTENMAALLMLQGYLGLIMYEVFMYCWYAEDILYQNSRLAVSAYSSGWVGAVPQLQRALVFVICRTQRPLGLTAGKFYYVSRESFVSLMSASYSYYALLRQVNDK

>LmigOR118

MRQTRSVESVLGSSAALLRLLGLWSPRIEDFSRTHRVLRGGLMLALSFGLMVTTLLKLVMDCPRELEELSACIFSATMLCEVFFKMVFFVLKVPTLHKLVQLLSEIRTEDSIGERNDEIRRRYQIVVDKMFLFLMATAVVTETMWAAIPLMHQLLNMDGEVTRLLPLPLWLPLDVYASPTYEVIYGAQVLLMPLTTTSLFFDFVFIDLMMRIAAELEILNYNISSVHENRKTVSTMSKDIHKLCQTVSDNKTNVQLVKNVRHHQAILRAVVLLEEAMNTGVFILFLATTIAVSSNIFTATALLQAHDGRIKALKMLSAMPPVLFEVGLYCVFGQIVINQSEKLMHSAYSCEWVDCDTRFRRSLHMFCVGAIRPLEFTVGRMYKLSRETLLQVLHGSYVMFNMLYTIQNRK

>LmigOR110

MLPSADDEKRLLGLMRFLLGRSVASRDGSFRTQLANGATTATLLSTAAANLIIMVCSGLKLYLDPPEETEKASQVAFLMTVSVANAMKGFSMVQQRARLQQVVAGLLAMRRAVCDGSGARHRYACSATLIGNIWMVMSVILGIVWGVDPVFNQPPQLNGTSPDPVLPLPIWLPLDASVPLTYWLMFALEAVVCGWTIFFVMIVDLLYVTLILNFAAELHVLNHNIQITCNAVDVTAHPKRKIGVSRHAGVSLYKGHNDDTAAIPNFSLAHFTAANPLMPAYIPDHFRVELSEDYDTYRLLVKSIQHHQLIVKCVNEFGKATGLPVLMVVSINVVNLCSNIISLAVLVEEDPHASAIAKSLIFTIALGSQTALYCLPGQMIIDQSERLAHSAFCCRWPDAGVRFKRSLLVFMACAGRPLRLRVGKLVTLSRETFQELLKLSYQLFNLVYQLQSS

>LmigOR71

MEKYRGKGTVESLSWAESGRSVLKLNIRHLWLMGVWPLGHSPVFKVYTGLTFLMGIWSVVECLLAVYYTWGQLEETTMVLIFTSTCSCGIVKLFFFVRNESSYSLMVREVASVMAAQSEACRDPALAAILRDSRSRAFRLSLGMLLFMFAQNFIWFPIPVVAHAGERRLPFSQHGWDNNSHFYGLSYTLQCLSGLYMSQISFGLDCLFASVMILVAAQLEILSGRILKLNQEVILEQRNESVLWKNKMTMDENRDTFYETLCFCIDSHQKILRFVTLLQDTMSPVAMTQFANSVVIACLALFQATYGEDMSAAFKCACYLPIPGGQLYLYCWAAHSVTENGESVSVAAYSCRWVEGSARARHALRTLMARAQRPLALTAGRLYPINRAAFLSMVNASYSYYALLGQMNNR

>LmigOR72

MEKYRGKGMVESLSWAESGRSVLKLNIRHLWLMGVWPLGRSPVFKVYTGFTFAIGIWSVVECLLAVYYIWGQLAETTVVLMFTFTCSGCIIKMFFFVCNERSYSLMVREVASVMAAQSEACRDPALAAILRDSRSRAFRLSLGMLLFMFAQNFIWFPIPVVAHAGERRLPFSQHGWDNNSHFYGLSYTLQCLSGLYMSQISFGLDCLFASVMILVAAQLEILSGRILKLNQEVILEQRNESVLWKNKMTMDENRDTFYETLCFCIDSHQKILRFVTLLQDTMSPVAMTQFANSVVIACLALFQATYGEDMSAAFKCACYLPIPGGQLYLYCWAAHSVTENGESVSVAAYSCRWVEGSARARHALRTLMARAQRPLALTAGRLYPINRAAFLSMVNASYSYYALLGQMNNR

>LmigOR75

MESGAAEDVEGPMGWSESGGSVLRLNVRHLWACGVWPLPGGWLFEAYAALGLALGAWNAAESLLALSFCWGDMEETTLLLTSTFTIGCGSAKMALLLARRGRYRALARRVQALASLQTGHCLADPALDDIRRGSQRRAFRLTLSMLLFMFSQCFVWFPMPVLAHWEERRLPFAQHAWDNNTRLYALSYAVQCVIGAWTSQLSFGVDLLFVAVMILAAAQLRILTIRIASLKTESWKVKPEGGARCEDVRPENGRDVMYENLCLCIDSHQKILRFLKHLENTMSSVVMTQFCFSVLVACVALFQATYSTDFTAVLKCASFLPVPGGQVFLYCWAAHNVTEQAEAVTMAAYSCSWVEASVRFKRALRILISRAQKPLVLTAGHLYPINREAFVSLVNASYSYYALLGQMNRR

>LmigOR64

MPTPTATETMTWSDSGNSILKVNIRELCLSGVWPLTGRKLFRVYSVIIWILGLENIVEAMVGIYLSNGDLEQITLVLPNTFTTAGGVFKMAFFLRDPYSYNALVRLMDELISDSSRYSTGNQQMLSIVRESRRSARRLSVFIYAFISTQIVIWFPMPLIAYAGEGKLPFIQHPWINSTTFPAYDTMYALQCLSSGFHIFISLGMDCFFAVVMIHTAACLRILSLRISALRSGDAGSSEVPVTSACSWGRESVAHDEMYKNLRACICSHQKIIGFISYLETVMNPIAMTQFAFSVLVACVALYQATYSEDMSAAYRCASFLPTPGAQVFLYCWAAHNIMEQGLAVSAAAYDCSWVSGDARFKRALRILMCRAERPLVLTAGHLYPVNRPAFLSLVNASYSYYALLGRVQSR

>LmigOR77

MKRSGMEDLGRTVSWSESGRSVLRLNIRHLWLCGAWPLPGSWLYRWYAILGLVVGAVNAVESAVSLYFYWGDMEETTLLLTSSVSNGCGTVKLAVVMRNQRQYHALARRVQALMALQGDLVAADPALSAVVEAARRRAYRITLGMLLLMFSQYFVWYPMPFYVDPGARRLPFAQHAWDNNTRLYALSYFLQCAASARGTQLSWSLDLLFMSVMLLAAAQLQVLTRRITALQEEKREAKLGNAANSEKAALADGGGNMYDNLCLCIETHQKILSFIKYLDDTMSSVAMIQFCVSVLLVCVALFQATYSTDSTAVLRCALYLPMPGTQVFLYCWAAHNVTEQAEAVSLSAYSSSWVGTGRRIKQALRIIISRAQKPLVLTAGRIYPIDREAFVSLVNASYSYYALLASMSKRE

>LmigOR34

MAGNIPWTDTVLWMNARVLALGGMWRPPWFQPKWYLLYRAWVLFTQFSFLFAQVQALWFFWGNIDKITHDTCLLITTILGLVKFFTFVLRQEDFFRMVQKIDDSRAEQSKSGDSEIVSILDASYRSARTITLYMTFLGGSSPGVWAIIPTILRRLGVFPPERELPATAWYSRRDTETPYYQMLCTLQYFSMQYSFFMAMCLDLFFVCIIIHAAGQLEVLNARFRRVGQIAGNHSADSHKQQKALEEFSGDVFSPEEIWEDLCDCIKQHQDIIELIKEIERLLSKIVLLQFLGATVIICVTLFQSSKNTDNIAALLLLQAYLGVVIYEIFMYCWYADDILYQSSRLAMSAYACNWPGAPPQLQRALVFIIRRTQRPLGLTAGKFYYVSRETFVRLMSASYSYYALLNQVNDK

>LmigOR94

MEALWWPVSPVRRGLRLLGLWVAPPGRRALHRLALSWVLASHAFLLLVGAASLVMDTPEDLPQLSFTAYTTLTCFGLIAKLVSFSLDGARLTRLLQLLAECRARFPDPGGRRGQHHLMAVRLHRFLQVSYRVNSVVWMFAPVVSAALAARSGGEEPVKRMYVLPLWLPVDTQASPAYEAVYVAQLATGWMLSETTVLLDVALLALMLHAAAELAVLNDRLRSQPAAAAAGPALASPVAYPKDGDTDPKHDHMYRHMVENIQHHQIIIVYTGLLQSVVRRAISVLLACNTVSICFHIIATVALLQKDIELVGMTKMVVGSTLYAYQTAILCLLGQRITTQSERLSASAYSSAWWEGDGRYQKLVVVFCERASGALSIRVCGLYSLSKETLLQVLKAAYSLFNFMYQAMETSNH

>LmigOR5

MLGQGQAGPGGRGQHLPRVDLLAHLSPILRCLAVFGMWPPSVYTSVSQKVAALTTGAATLSLFLMQLVAEVMALAASPVSGAAELYRFIYNFSVVDLHLQGMGKWAVMVARRRRYTALVHRLQHCVRLSGLADADYKNQQAAVSRLRQQLDDCRRWGVRANAVWLSVCVYGVTHWCLVPLLIGDNSLPFDALYLFSTDQPPLRQVAHCIQYVAGLQNVLLSVFFDLFVFWLHLLLCAQLRYLAGNLRRLRHLTDAAQYRRMLAACVAHHSHLLSTMQELNSCAGPSFFLQCFENTIRMCMIAFMATTTVADQMQVWSSAQFFLAAVAQLFLYCWCGQQLSHLAESISEAVYDSGWEDHDVSTQKTVAFIMWRAQKILVFKGGWFYTLTTETFVELIRLSFSYYTVLRNINDT

>LmigOR62

MKQSASLEDCSPLTWEYTQQSLLKLNIRLLWALGVWPLPGSWVFSLLKVWLAALAVGNAVENVLGVWKNWGDLTEVTYSLLNAFTIGAGVAKTWHLSRYQPRYCLLVRRVDRLTRSQRRYCDGDAAMRAVTLGCRRTARRVTLSAFAYLTALCLIWMFMPLVAHPGERLLPFNHIPWEPRRFPLFYELSYAVQSASSVVYVFISFALDCFFAVVMIFLTEQLMVLNLRIRQLYARRDGDGSVLVAKQHSDKTVLDKHEEMYKELCLCIDTHQDIIRLLSFLDAVMNPIVLTQFMLSVMAACVTLFLESYSPDSSSVLNSISYLPTPGIQVYLYCWSAHNVLEEGFAVSEAAYGCAWYEGGGRFKRALRIVMCRAQKPLVVTAGRLYPVSRATFVSLVNASYTYYALLSRVHNRG

>LmigOR50

MKRGDTAVEEDEKDGEVLTWQETGGSVLKYNIRQLHVFGVWPLPGSAPFHAYTVLVSAIGLAGLAQDLAGVCACWGDLQEVTMALIHILSVSSGFVKLAFFVRRRRHFNALVRRTDRLVAAQGHFCDADATLRATFRASHRKAVYVTLLAYGYLSVQGVIWLPLPLIAHPGERRLPFMQMPAAATASVYVYALLYSLQCLSSMFVTFVGVTVDCFFAVVMIHTAVQFRILNTRISALRADAAVPVDTGTAVGGQTSEHDSLYKQLCQCIQTHQRLLRFVTYLDSVMNPIAMTQFTFGVIVVGITLFQASYSPSSSTMFKCVTWLPMPSTQIFLYCWGAHDIMDEGQSVSRALYSCGWVDAPPGFKRALRLVMSCAQRPISLTAGRVYAINRATFISLMNAAYSYYTLLRQFNSR

>LmigORco

MQKPHGLVADLWPLIRMVQYSGHWMLEYSGGKALRAIYSSAVSLLVVTQFALMAVNLIQRSGDVNELAANTITVLFFLHPVTKFGYFAVRSKAFYRTLATWNQSNSHPLFAESQARFHQLSVVRMRRLVMYVVSVTALSVVSWTSITFMGDSTREVTDPDNANETITEEVPRLMISTWYPFDASSGMGYMLAFVYQLYWLTATLMHSNLMDVMFCCWLIYACEQLVHLKEIMKPLMELSATLDTVVPHTSELFRAASTLPTNEPLYDAGNGAADGLTIRGIYSSQRDFSGFNRRSAALSTVREADAGGAVSSAGGIGPNGLSKRQEMLVRSAIKYWVERHKHVVRFVGNIGDAYGAALLLHMLTTTVTLTLLAYQATKIDSVDVYAASVLGYLFYTLGQVFLFCVFGNRLIEESSSVMEAAYSCHWYDGSEEAKTFVQIVCQQCQKSLMISGAKFFTVSLDLFASVLGAVVTYFMVLVQLK

IR

>OchiIR25a

MILLLLTALLSNLLSALGEPTINYLHVNDDRNLIPDKALKQVISELSKQGMIFDGVFKATANGSDVEALIDSMCLGYNSSIDQNKKIHIILDTTLQDVSSEAVKYFTRTLELPTVSASCGQEGDLRYWRNIDKKQEKYLIQVMPPIDTIPEFVRSFCSEQNLTNAGILFDDTFIMDHKYKSLLQNVPTRHIINEIKFQNISNQLSTFKQREVFNYFILGRMDTVNRVLEAAAEMEFYGRQFGWYAISQDEAIPSCKKCGKGASVLHLKPNDPEGTVTGSENPKLTYQFYYELFRNTFLAIAQMIDEGSWPDMEYIPCEKYEESKNVPPMRQLNILDALHQISVQNPGAYGQILLSSNGHSHMQFNMTAFNVSLSDNSATEVGNWAADLDSPFITKVKPSVPVTQYTVVVALQQPFVIKYVDENGNTKFKGYCIDLINAIRNITNFEIEIYEVADGKFGNMDEYGKWNGMIKDLIDKKAHIALGALSVMAERENVVDYTVPYYDLVGITILMKKPKTPTSLFKFLTVLENDVWLCILAAYFFTSFLMWVFDRWSPYSYQNNREKYKDDEEKREFDLKECLWFCMTSLTPQGGGEAPKNLSGRLVAATWWLFGFIIIASYTANLAAFLTVSRLDTPVESLDDLSKQYKIQYAPIANSSAHVYFQRMAAIENRFYEIWKDMSLNDSLSEVERAKLAVWDYPVSDKYTKILQAMTEAGFPSNMEEALERVRASKSSSEGFAFIGDATDIRYQVLTNCDLQMVGEEFSRKPYAIAVQQGSPLKDQFNNAILQLLNKRKLEKLKEQWWNQNPEKRNDCEKQDDQSDGISIQNIGGVFIVIFVGIGLACITLAFEYWWYKLRPQHNAIVEAAPPRTKSDSLQALNMMRSSFDKRYGRRQGVALAGVTNPW

>OchiIR8a

MWPLWVSVVAVQLQFASPQATSDPLIIRFLLVTEVNATWVGGELRANLSGLEARYVGLRLQLDLSAIEVDREHEVEEFQQKVCGEMAAGVSALLDATWTGWRRLRDEAQHRGLPYLRLDATLANLVDAVDKYLQAREASDAALIFHTEEELDQALYHLIGNSVLRVIALNGLEKDTVSRLKDMRPVASYFVIFADTAHMGELYSKAAAGGLVRKAERWTLVFTDWSWASFNYRQLNLSTALLQMKRTSCCPLQGEPDNCKCTLQKVAPAFLRAALSAVVEALTELHSKGLDVRPTARQCSLPEPGSDDDDGGGDDTEAATEAPPNATGNYDAFLRAIAPRGQGNSTLFFRSPPAQLTFNTPLQLRMFNQSEDFNLGDWSPEKGLQLNTQLKPAKRFFRVGTAEGVPWSFPVRDEDTGEPLLGPEGEPIWDGYCIDLLKKLAEPVHMNFDYELVPAKNNDFGSRSPSGTWTGLVGDLAMGETDMIIAPLTMTSEREEVIDFVAPYFDQSGISIVIRKPVRETSLFKFMTVLRLEVWLSIVGALTVTGIMIWLLDKYSPYSAQNNREMYPYPCREFTLKESFWFALTSFTPQGGGEAPKALSGRTLVAAYWLFVVLMLATFTANLAAFLTVERMKSPVQSLEQLARQSRINYTVVMNSDTHEYFRNMKNAEDVLYNVWKDITLNSSSDQSRYRVWDYPIKEQYGHILQAIDQAGPVPNASIGFQKVIDQEEGKFAFIHDAAQIRYEVSRNCNLTEVGEMFAEQPYAIAVQQGSHLQEEISRQILDLQKDRYFESLTAKFWNSSAKGTCPNSDDSEGITLESLGGVFIATLFGLALAMITLAGEIFYYKRKKLATVNVTSTSAKVPKKQVTIGKEFRPVIEKTAPRVSYISVFPRNQLY

>OchiIR76b

MNLSPILKMVVTTVCSNYLFNGTKMEVPEGEPDPGCVLRKPKLMEGKTIRIGTLENPPLTLTNKTDGALIGQGVIFEIVDILKHRLGFNYEVVKPELNILGDENHGIIGLVHKKQVDVAVGYLPQFSQHARLVRHSESLAEAPWVFLMKRPLVSASGTGLLAPFDATVWYLVLASVVLMGPAIYIIILVRVRLCAGSERLTRIFPLSSCVWFVYGALMKQGSTLMPVTDSSRMLFATWWIFITLLTSFYTANLTAFLTLSRFTLQITDLKDISTKKAHWAAQRGSAMEYLVYNNDEYSFLNQSLQAGYGQFVDVSDADMLLRIKKDDLVYLREKQHVEHTMFRDYLEKTRNPLVEEKDRCTFVMTKQPFLHLPIAFYYPLNSSLAHIFDPLLKALVETGIVRHLLRKDLPQIEICPLDLGSKERQLRNSDLYMTYMIVVIGFCAATVAFFGEILTRQVKRCIVEAEYNMEPTASYPDDWKTVKAQAKRAPYTMYLNGSIINVKQPAYGVTKDFQSSSRKSLSKQRNTNYVFQYTS

>OchiIR22

MRAWAHRGLRASVHDPSQLRTDLGRLLDTRRGAVVVVVVADISCPGALLLFHKAAERHLLGMTRHWLLLRGVEPLQFPELALDSSVTIASPGPSGSSSWLLQDAYGLRDKLFLSEPVSWLPGQNLPASPVRDNLHGITIKAGIVRLFTDFAHFEDPTYRHVDTWSKFCYAVSKVLGERLNFTQEIVFTKQWGVRVSPGVYRGLVGQLQRGETEVALGFALRSDRLDFIDFTTPVGRFQPAFMFREPSLAAVSNVYTRPFSRGVWLSYSLAALLLALLVVASQRLLARAQGLLLSADTDPAVVPPWADVPLVAFSIICEEGMQDPPQNVSSRILLMFLLVLAVFHVTAYSACIVSLLQLPSGSINALDSLFGSKLRVVMQDVPYNVNYGNETKDPLTRSFYQDHVYSQSRKVFMPLEECVALMREGSFACHADESAYKVIGDTFLEAEKCGLKSLPMIPLWSIILGVRKQSPYKEVISVTVAWLREAGLLAREWRRWVAQKPRCLNRDSNYAEVGLTEVSPALMMLGYGVAGSLVALLLELVLDRLVATRQGSRQRHRQRQRQPGNDGPRVPTAFLR

>OchiIR6

MRIVLMMAALRIPLCYGRLLLQNDNLSSFDSLIGEMGSRILLKEFGNYKCVVALSDAPEGLTSLNTGFLSTIRVSLDSADAPVEHLLVSALDADCQGLVVRCCDATAGVAALFRASKLAIRRVNRRILVLPATLVPQINVSSIFSLRNIEITPDIIVARVAENTESFELVTLRFTGEDTWRDELVVARWSRGRGFQPPSADLYPDKLADLQGRRLALATFHFPPYVILDPDANVFDGIDTRIILEFFRKINATWRVLEDTMGMGMWGTTWENGTGIGILGAVAMDKADMAFVGLYCWYTEYRFVEFSRPYQRGGITCLVPRPLLMPGWQVPILPFSPVLWASVFCSVIFATAALYAVKKLSDRVLGTEEGSAGGRYSTVEDCFFRSVGLLVLQTPDVERRHTRVVGPTRHVLSWLLIAYLLITSSYGSGLSSVLTIPRYESPIDTVVDLHESGLEWAEIDPVYLNSIKGDTDKLFRDLTNRFRVLGNDVLRSRIATRDLAYAVERLPGGYYTFGDYIDEEAMSKWLRPMREDVYWEPVVLVVRKGWPFLEKLNSVVDRLFQAGIIRASEGQISRKWLAARVQLAAQAGLRSSTQSSDEPIKLQLGHVPGEFILLLLGLCLSLVALLLEVAVHRKTKSAALLEISSTIKITLAVKRS

>OchiIR17

MVGDVHEGKAHVGLSFFTMSAARTSVVSFTTPIFLGRYYIFIRRDGITQMDPHPLELLVPLKNDLWLAAVFTLFAYTIAMASIFYIQGKPLSDAVFSIIGAFVQKHDETTSYHHRITSEQCLRFVAYITGSILLSAYSAVIISILTVTKQKLPFQSFAEFEQDGSYTLSVTGNSFEYDFFKLSQQKLLQKLFSNQLDIHNPPRTKLEGLSRVCTEKKYGFLSTDLSIQSSWSHQDCNVIAVPIDMFRCTLALILRNDSPYIGAINFHIQILRAAGLLNHMLIFYNIRDTSS

>OchiIR20

MTLLCHTSVILGIFILCLTSTVLCKNQNDHSNDIKFILDFTRHTITSSSIKGVHAFVCWNAGDLQLIKALSRSGILASVYNGWQNWQQLPTFHIDGKSLLFILDLKCEKSFFLLQKAGKNEELFRPPHIWLILHKAYSPGNLHTVDVGDDNSPISATINNERKYVMQQNIAAITYNNTKLTEAEDLGGEFNSVEVYDHSYGTGPTRSTGYVNGNGTHGTQISSRIRKRNLGFKSKETNDNNPAHVIMERCFYCEVFRDLNILVDSEVIVGRRETENKYTILEAYRRRKHGDLVVSELGYWERTAGIVWRAVRQVAIRRLDLKGTKLVASIVITNPETLDHLNDLHNRHIDTVTKLNYLLLLHATEVLNASLELVVTDEWGYESNGSWSGLVGSLQRAEADIGGTALFVTADRMPLIDYIAMTTPSVTAFVFRQPPLSLVSNLFTLPFSRAVWASTAGVVAACSALLWAATRWEWRRRLGTDDHLRQLRDQWGDVAMLAVGAVCQQGSPAESRGVPGRIVTLSLLVIVMFLYTSYSACIVVLLQSTTSSIRTLADLLHSPLALGVHDIVYNRHFFPAADDPVRRALYRQKVAPPGSEPHFMTLDEGVGRMRAEPFAFHSELSPCWQLVQETFREEEKCGLQAIPFLQLIHPYIAVQKNTSYKEMFKIAYRRIWERGLQHRQLSRLYTTRKPSCAAGRGSSFVSVGIADCYPALLVPVYGGALAIFVLLLEILLHRRKGQKTTTVTAMNT

>OchiIR28

MFNFSVVLYKTESYGYLTPNGSIDGQAGMLFDGTIDLALSSLGMSHHRLDFIDFTAPSRMWTLKMVFRHPRSRAVYGTIFRPFSAALWLSSGLLFLLVLLVARLGCWAAAHHAADDSWSAAFLLVSSAISQQGTTLDTRRPSWRLLVLLSFACALLLDTYYTAAIVTSLLLPPPRTINTKADLVHSPLAVGMENISYTHEYFEKSSDPVDHALMEHKVWPRGVRQPNYHSLLAGVRKVATEPFAFTAEDVSLYPLLDRYVTEADKCSLVAIDFLRSRSTYMPVRKNSPYRELVTIGLRKLLERGHISRTRSLWHARPPACLRESEFATVELVTLAPAFMLLAAALLLASLLLLFELRHAARNGKQSLVGREQSGRRIEGLLVTSPRRQRVSVSQS

>OchiIR29

MRPPALCLPAVLMAAVGCLLWWQPCSALLAQHATSLAVDYLTLRHARTVVTYTCSPYDSRRLSRLLLERLQAWLVLSPHPRHIPPGSSVFVDHSCQRNRELLQQCAKAEMMNTSTRWLVWSAREPDERLQVRLDSDVTWATWRGGGEGALLWGLSRPHPGWPLERWPVGAWAPAAGLRYDSWRLQRVAKKNLQGLALGAALPIVDTPLDRLGQRLRNVSDRQLDTMARFGWGLSNTLAALLNFSIILYRVPNFGSLVSENEMDGAVALIHNGTVEFGAAGFIMTTRRMDFMDYTGPGRLWAPEIMFRHPKSASVLTTIFRPYTAQLWVSSGALFLLILLVSRLFSWAEHRVTATVHPVDNSWNSTFLLVSSAIGQQGVSRSSEWLSWRVLLFVSFLCTNLLDTHYAAGIVTSLLLPPPRTINSKKDLADSPLGFGLENVSYTYQFFVKSDDPVDRALCRRKLFPAGGRGRPNFFPAEEGVRKMAHEPFAFHAEDVRVGPLIDRLFSDDDKCALVFIPLLTPVATYTAVRKNSPVKEVFNFGLRMMWERGHISYLRKAWYFTRVRCLSETEYASVDLVPMSPAFMLLGCAIFLSGLLLLLEVRQHRRSQREVVNLSGGTREKQTGWHKVHFSSKVHIRLLPSVDKNVYWDRTAPGQ

>OchiGluR8

MPDIEPEQIEVESALMYDAVHLFDATVRDMRDLQIEDSPSGCDNTWGHGHSFINLMRDRYKTLRGLTGPVHFDYEGFRTDFMLHLVELTVDGLQKIGTWNSTTGLTLIREEVSTEVRQEESLQNRTLIVLTAISKPYGMRVDTSLTLKGNAMYEGFGIDLIHEISLMLGFNYTFVLQLDSKYGTLNKETKKWDGMVRELLDCNADLAITDLTITAERESAVDFTMPFMNLGIAILYKKPMKAAPKLFSFMDPFSNDVWIVMFAAYIGVSLMLFVMGRISPYEWTNPYPCIEEPETLENQFTLSNSLWFTIGSLMQQGTEIAPIAVSTRMVAGIWWFFTLIMVSSYTANLAAFLTVESMFQPIKNVKDLADQNTIKYGAKRGGSTLNFFRDSEDEIYRRMYNTMINTPGVLTKDNDEGVEKVKNSNYAFFMESTSIEYVVERNCDLTQVGDKLDNKGYGIAMRKNMTYRNSLSTAVLKLQETGRLQALKNKWWKEERG

>OchiGluR9

MLVALLALLPLASALPEVIRIGGLFHPADDRQEVAFRYAVDRLNADRSILPRSRLSAQIEKISPQDSFHASKRVCHLLRSGVAAIFGPQSGQTASHVQSICDTMEIPHLETRWDYRLRRESCLVNLYPHPTALSKAYVDLVRAWGWKSFTIIYENNEGLVRLQELLKAHGPSEFPIAVRQLGEGTDYRPLLKQIKNSAESHIVLDCSTSRIYDVLKQAQQIGMMSDYHSYLITSLDLHSVDLEEFKYGGTNITAFRLVDPDKPELKKVIRDWGYGSYGRKADMGPIRTETALMYDAVHLFAKALHDLDSSQRIDIKPLSCDAVDTWQHGYSLINYMKIVEMQGLTGVIKFDNQGFRSNFLLDIVELNKDGLKKIGTWNSTEGVNFTRTYGEVYTQIVESLQNKTFVVTTILSSPYCMRKESSEKLTGNAQFEGYGIDLIHEIANILGFNYTFKLVPDGRYGSYNSETKEWDGMMKELLDQKADLAIADLTITYDREQAVDFTMPFMNLGISILYRKPIKQPPNLFSFLSPLSLDVWIYMATAYLGVSVLLFILARFTPYEWQNPHPCNPDPDHLENQFTLLNCMWFAIGSLMQQGCDFLPKAVSTRMVAGMWWFFTLIMISSYTANLAAFLTVERMDSPIESAEDLAKQTKIKYGALRGGSTAAFFRDSNFSTYQRMWSFMESARPSVFTSSNVEGVDRVVKGKGSYAFLMESTSIEYVIERNCELTQVGGLLDSKGYGIAMPPNSPYRTAISGAILKLQEEGKLHILKTRWWKEKRGGGTCRDDTSKSSSTANELGLANVGGVFVVLMGGMGVACVIAVCEFVWKSRKVAVEERIQQKKPSPKYTSRIYTTQ

>OchiGluR12

MAGGRPGLRQRLTLGRGLGAQRLLAASWLVLLLQLPAAAASNKPISSVRIAGIFEETDPPELQYAFQAAVEAVNRQTTSEYPLGLSGHRPLIQEHVEVINGADSYGAKRQACSLVEQGLAGVVGPRQSASTDVVRSMCDRLEIPQIIVNWDPYPPPIASYQFNLHPHADFISQALVDVLYELKWRSYTVLFQNDESLVRLRGVLQEREPWDPPIAVRQLEEDGDNRPMLKEIKQAAESRLVLDCDTDQILPVLQQAADIKLAEVYQSYLLTSLDAHTLDFTQFRLSGTNITILRLIQPESAAVMKTMQDFDYALTKLPQTVTITAQTLRTETALMYDAVRVLAEALFKTSTMETVQTQSLNCSDESAVWDRGVSVREFIRDMRHRGISGDIHFDAEGRRLGFTLEVLELSATGFKRVGTWSPEGGFMGTRSETQTLEETQNIIQGRHLIVSSRLGPPYLKEKAATSPPRVGNDRYEGYSMDLIAQIADLLNFTFEFKLTPDGQYGSFNDKTGSWNGLVGELIAGRADLAICDLTITQERQSAVDFTMPFMTLGISILYKQPEKADPNLFSFLDPFTIDVWIYMATAYLGVSIIFFILARMAPGEWDKGHPCDPDPTELENTFNMINIFWFSTGSLMGQGCDLLPKAVSTRLIAGMWWFFTLIMIASYTANLAAFLTNSKLDAPIEGVEDLAKQTKIKYGTYGGGSTAGFFRNSNDSLYQRMWIVMKQARPDVFTADNQEGVERVKKEKGNYAFFMESTSIEYQTALNCDLRKVGGLLDSKGYGIALQRDSPYRTAVSGAVLTLQERGNLSALKTRWWKAPEGEECDEGDGSADNSAELGILNVGGVFLVLAFGTLGAFFVAVLELLWNCRTIAVEEKITPWEALTSELKFVVQCSNDEKPVRKPPDTDGDGEKEEEVSQLHYGRLSADK

>LmigIR23

MATVVPLLLLTTTLLAVTSTIQPPEDSPSQADTATLVAEFAQRRHLHFVVAFGCWPDGGVSAVRAWSRRGLRASVHHPEGPLPDLGALLSPARGAIVACIALSCPAGLQLFHKAAGRSLLGGLRHWLLVGALDADQLPRLALDSSKVHDDFSRYDDLRYRHLDTWSKLHYAISKVVAARLNFTYDLVFADQWGTRVTKDMYNGQVGQIQRGEVDVAVGGVALRAERSDAIDYTTPTGLFHERCSPVVAASGVAAEGARGRGRKNAAPCLHGGARRKYVARTSDLCDSTLGLTGVLLEQQWLGMQEPPQNVSSRILLMFLLVLAVFHMTAYSACIVTLLQLPSSSINTLESLFNSKMRIVIHDLPYNINYGNETKDPLIKNFYRERVYSQPRKAFRPLEDCVAQMREGGVACHADEAAYKVIGDTFLESEKCSLKAVPMFPLRGIIMAVQKQSQFKESFSRTVAPVWAVGACEQVLHIPHSTAVTLAIRGYKTPPNHVSLHWGHPRPYSVQCRPCLPDRGESDGHLLYRWPDVAAGDGPAEARVDALGGAEAPLPEPRQRLTPSGASSGYAEVGLTEVSPALLMLCYGVAGSLVALLLELLLHRAIAARPGRHQRQPKAPATGNALLNRAFLR

>LmigGluR11

MILVCLARSLTLLMVASILLLVRKAARLAVYEAIMISLKNAHMPAIIRVETALGISSPPPPSSALPVNHRMLDMLNWFSSSSQLGSRGLDFCHSMGAAYMQFRTCKRYPSSICIKNEEEQIEEVNSIKFLGLQLDNKFNWEKHTTELQKQLNKSRFIKGMVKSTTDCRSSVIVRSHIARSAL

>LmigIR5

MSAASIATAPRMPSPEPLAHTLPHLPLVGSTTVQDAFFFLANSRMMRVSTPSNILASLSSTTYGGKLNVARVSGRPPRSANLSGYRSADGGCRPNLRHQRATTSSSRPVSAPVNLCVTSS

>LmigGluR7

PRGGAAAAAAVCVATALLALHGAPSAAALPPVIRIGAIFTEDQKDSSSELAFKYAVYRINKDKELLPNTTLVYDIQYVPRDDSFRTSKKACRQLEHGVQAIFGPSDPLLGAHIQSICEALDVPHVEARLDFEPSFKEFSINLHPSQEHMNRAFKDLMAFLNWTKVAIIYEEDYGLFKLQDLVKSPPVVRTEMYIRQATPATYRQVLREIRQKEIYKLIVDTNPKHMYKFFRAILQLQMNDFRYHYMFTTFDMETFDLEDFKYNSVNITAFRLVAVEDARVRAALEQMSRFAPVGPSILHRSGVLQAEPALLHDAVHVFARGLAALDRSHRLRPANLSCELERPWDDGLSLYNYISSAGLHGLTGHIEFSEGRRANFRLDLLKLKREQLRAVGSWSPSGGVNITDPSAFYETSVNNITLVVMTREEKPYVMVKEDVNLTGNARYEGFCIDLLKWIAAQVGFHYAIRLVPDHMYGVYDPETREWNGIVRELMEKRADLAVASMTINYARESVIDFTKPFMNLGIGILFKVPTSQPTRLFSFMNPLAVEIWIYVLAAYLLVSFTLFVMARFSPYEWNNPHPCLAESDVVENQFSVSNSFWFITGTFLRQGSGLNPKATSTRIVGGIWWFFTLIIISSYTANLAAF

>LmigGluR2

FYPQVLAPSSGLLDYAISMRPDYHQAIIDVVGHYGWKGVIYLYDSNDGLLRLQQIFQSLRPGRGSFHVEAVKRVQNTSDAIDYLHSLEEVSRWSNKYVILDCPTEMAKEIVVTHVRDITLGKRTYHYLLSGLIMDDRWESEVIEYGAINITGFRIVDSSRSYVRQFLDSWRKFNHSSSTAVSHGYISVSIYKNTSALTTIRIGRPSYFRKLVEISSTTVVKHPSTGIAPVGTVSCASNGYPGSNRERNINTTIDMTLYHKPLLLILSFVTILIFQKINAVSAQVEIEGLTGDIRFSEDGRRRNYTLHVVEMTVNSAMVKVAEWSDQTGLSEVAAKYVRPWPGSSIYRNKTYIVTTIVEEPYIILRKPEAGEVLTGNDRFEGYCKDLADLIAKKIGIHYELRIVKDGQYGAENPDVEGGWDGMVGELVRREADIAIASMTITSERERVIDFSKPFMSLGISIMIKKPVKQKPGVVSFLNPLSKEIWVCVIFSYVGVSIVLFIVSRFSPYEWRLLQPALHEPPGHSLHDRYVGGSNPPPSGMDVCVVFSISCGVAVVNFCFANTFTMTEYKDRYIGGELAPLPPPRELAAFVTDLALDRSISGRIVGSVWWFFTLILISSYTANLAAFLTVERMVAPINSPEDLASQTEVEYGTLYHGSTWDFFRRSQITLYSKMWEFMNSRKHVFVRTYDEGIRRVRNSKGKYALLIESPKNDYINEREPCDTMKVGRNLDAKGFGIATPLGSPLRDEINLAVLSLTENGDLTKLKNRWWYDRTECKHSDKQDASRNELSLSNVAGIFYILIGGLLLAMAVALLEFCYKS

>LmigGluR8

MLGSHLFITDNCWSKKNVLVSLIILFCAIQNCGCFTEIKIGALFHGENSQQEQTFQLAVAHVNKKQHGYELKPYVQYVEEHDVLQVDAAVCDLLSNGVWAIFGPNSSSASEQVQSICDTIEIPHIEMRWNTKQRRQDVALNLYPHPKALSRVYADLVKAWKWKSFTILFESDEGLIRLSYVLKMYGPKDFTVTVRHLNDSSNYRPVLRRVKRSGEINFILDCSIQNLPEIMKQAQQVGLMTDAYNFIITTMDVHTIDLEPYQHGGTNITGFQLVDTMSEHVQSIINSWKNFEIEDADWPDIEPEEIEVESALLYDAVLLFDNTVREMRDLHIEDSPSGCDNVWGHGHSFINLMRDRYKTLRGLTGPVHFDYEGFRTDFMLHIVELTGDGLQKIGTWNSTTGLSLIREEISTEVRQEESLQNRTLIVLTAISKPYGMRVDTSQTLKGNDMYEGFGIDLIHEIAIMLGFNYTFVLQLDSKYGTLNKETKKWDGMVRELLEGNADLAITDLTITAERESAVDFTMPFMNLGIAILYKKPMKAAPKLFSFMDPFSKDVWIVMFAAYIGVSLMLFVMGRISPYEWTNPYPCIEEPETLENQFTLSNSLWFTIGSLMQQGTEIAPIAVSTRMVAGIWWFFTLIMVSSYTANLAAFLTVESMYQPIKNVKDLADQNTIKYGAKRGGSTLNFFRDSEDEIYRRMYNTMINTPGVLTKDNDEGVEKVKNSNYAFFMESTSIEYVTERNCDLTQVGDKLDNKGYGIAMRKNMTYRNSLSTAVLKLQETGRLQALKNKWWKEERGGGQCVDAEENTDVSGLKLENVGGVFVVLMGGVVFAIFVTFFELLWDVGHKSLKEKIPFKTLLMEELRFVAKLHGTTKPVRKYNQDAEVEDTNNFIPLSPYTNSYGFVDSKEPLT

>LmigGluR6

LVSGAVFTQDQKDSSTELAFKYAVYRINKDKHLLPKTTLKYDIKYVPKDDSFHASKKACQQVRFGVQAVFGPSDPLLGAHIHSICDALDIPHLEARLGLPSKEFSINLYPSQSLLNNAFQDVMAFLNWTRVAIVYEEDYGLIKLRELVRAPTNRDVEIHLRQADPDTYRSVLKEIKSKEIHNLVVDTKPEHMHHFLRGILQLQMNDYKYHYLFTTFDIETFDLEDFKYNFVNMTAFRIVDAEDVGVKEILKDMERFQPVGHSILNRSNIIQAEPALMYDSVYVFAIGLQTLDQSHTLRLSNVSCEEEVPWDGGLSLINYINSVELKGLSGPIEFKEGRRIQFKLDLLKLKQHALVKVGEWSPGAGVNITDRAAFFDPGTMNVTLVVITILENPYVMMHYGKNYTGNDRFYGFCVDLLERIARDVGFDYILDLVPDRKYGAQDPVTGEWNGMVLHLMKHKADLAVGSMTINYARESVIDFTKPFMNLGISILFKAPRGAPPRLFSFMNPLAVEIWLYVLAAYVLVSITMFVVARFSPYEWHNPHPCDTENDLIENQFSLANSFWFTIGTLMQQGSDLNPKATSTRIVGGIWWFFTLIIISSYTANLAAFLTVERMITPIENAEDLAGQTDISYGTLDSGSTMTFFRDSMIETYKKMWRFMENKKPSVFVSTYEEGIQRVLEGNYAFLMESTMLDYIVQRDCNLTQIGGLLDSKGYGIATPMGSPWRDKISLAILELQEKGEIQILYDKWWKNPGDTCTRTEKGKESKANALGVDNIGGVFVVLLCGLAIAVIVAIFEFCYNSKRNTQSELSLCAEMAEELCFALRCRGSRQRPALKRQCSKCAPAASAAPYVPAVLDLPPANGLRSHVSTRR

>LmigGluR13

MRQEGLSGDIVFDEEGRRVGFSLEVLELTATGFRRVALWTPEQRFVSSKTETQKLEEKHSILQGRHLIVSSRIGAPYLMEKQPTTPPRVGNDRYEGYSLDLITQISEILNFTFEFKLAPDGKYGAIDEKTGTWNGLVGELIAGFRDPRLSHIRDTLRLGFNTRNAIAYIQESDFKCSNVCIAYETMFQRADLAICDLTITQERQSAVDFTMPFMNLGISILYSKSEKADPNLFSFLDPFTVDVWIYMATAYLGVSLTFFALARLVILAALFQFYLKKQIYA

>LmigGluR12

AVAAASCLLPALASNNPTYSVRIAGIFEESDPPELRHAFLSAVEAVNRQTTMEFVLGNAGHRPLIRQHIELIDSADSYGAKRRACSLVEEGIAGVVGPRQSASTDVVRSMCDRLEIPQIIVNWDPYPPPIASYQFNLHPHADFIAQALVDVLHELKWRSYTVLFQNDESLVRLRGVLQEREPRDPPIAVRQLDEDGDNRPLLKEIKQAAESRLVLDCDTDLILPVLRQAADIKLAEVYQSYLLTSLDAHTLDFTQFRLSGTNITILRLIQPESDMVMKTMQDFEYPGTEVPMTITPQTIRTETALMYDAVRVLSEALFKTSTMSWVQAQPLNCSDESSVWNSGVSVREFIRDMRHEGISGEIKFDAEGRRTGFQLEVLELSAAGFKRVGTWTPEEGFLGSRTETQTLEETQNIIQGRHLVVSSRLGPPYLKEKTPTTPPRVGNDRYEGYSMDLISQIADLLNFTFEFKLAPDGQYGSRDEKTGTWNGLVGELIAGRADLAICDLTITQERQSAVDFTMPFMTLGISILYKQPEKADPNLFSFLDPFTIDVWIYMATAYLGVSIIFFILARMAPGEWDKSHPCDPDPTELENTFNMINIFWFSTGSLMGQGCDLLPKAVSTRLIAGMWWFFTLIMIASYTANLAAFLTNSKLDAPIEGVEDLAKQTKIKYGTYSGGSTAAFFRNSNDSLYQRMYLVMKQARPDVFTSDNQEGVERVKKEKGNYAFFMESTSIEYQTALNCDLRKVGGLLDSKGYGIALQRDSPFRTAVSGAVLTLQERGNLSALKNRWWKAPEGQECAEEDPAATDSNELGILNVGGVFLVLFFGTLGAFFVAILELLWNCRKIAVEEKITPWEALKSEVKFVVQCSNDEKPVRKPPDTDGDKEEEASQLHYGRLSADK

>LmigGluR3

GAIFEQGTDEIQAAFKFAMLKHNQKDDKRFELQAFVDVINTADAFKLSRLICGQFSRGVFSLVGAVSPDSFDTLHSYSNTFQMPFVTPWFPENVLAPSSGLMDYAISMRPDYHQAIIDVVRHYVYKIQYITKERSSSSHSLQIYCMIREAQDDIQHHNIVSNYEAHAIDYLHSLEEVSRWSNKYLVLDCPTEMAKEIVVTHVRDISLGKRTYHYLLSGLIMDDRWETEVTEYGAINITGFRIVDNTRQSVRDFLDGWRKVDLGTAARTGRDTISVSTARLANEATDTVAVLKTIRGPVEISGISIIVRRVKILCSAQQSSFPHWERTWYSEQSSAQETSSHHGDGRMVCARGSIGGRISTASRGVECSTSRGWVTPWEHGDKISRFLRKVELEGLTGDLSFGEDGRRRNYTLHVVEMTVNSAMVKVAEWSDETGLTPVAAKYVRLRTSTDIQKNRTYIVTTIVEEPYIMLRKPEPGEILMGNDRFEGYCKDLADLIANKIGINYELRIVKDGQYGAENPDVEGGWDGMVGELVRREADIAIASMTITSERERVIDFSKPETLFSGWFLVVETFGVFQHMAREICLRTRSVGHSFFPAFESKVRIWNLEKRTILVYDFLHLHLHTYPQFNRKCMAEGTLYNCKAFPTYSTPKYNEAIRMYLRLRTSPNFPSLVFVITTRSASRCQQDHSTLGFKFCPSKFPQQNFSKRTSSISGRIVGSVWWFFTLILISSYTANLAAFLTVERMVAPINSPEDLASQTEVEYGTLYHGSTWDFFRRSQITLYSKMWEFMNSRKHVFVRTYDEGIRRVRTSKGTYARSSTSPKNDYTNEREPCDTMKVGRNLDAKGFGVATPLGSPLREKINLAVLSLTENGELTKLKNRWWYDRTECKHSDKQDASRNELSLSNVAGIFYILIGGLLFAMAVALLEFCYKSHVEASKLKMSLSDAMKAKARLTIGAGIDFNSQRYYTPTHQMNSIDNDQLHSNTHTQV

>LmigGluR10

VCGLTSTGLSGIIGPRHSSSMDIVRSICDTLEIPQIVTNWDPSPPVPRNYQINLHPAAHFVSQALADVVRALGWRSLALVYERDDALARLRGLLQARERGQPPIVLRQLAADADHRQPLLKEVKKSSETRVVLDCEPDHILDVLRQADEVKLMHVYQSFFITSLDAHTVDYSQFLKSGINITTLRLIQPGRESVVKKMQDFTYGKYYNRELLKPERIRLETALAYDAVHVYANALFKRETAVPVTDEILFCNESLVWDHGISVRDFMLKMEHEGLSGSITFDSMGTRVGFTLEAVELSSAGFKRIGTWTPENGFSTARTEKETIDDTLHKIYGRHLIVASRLGPPYLREKPKKVPPRIGNDRYEGYSMDLITQISEILNFTFEFKLVPDGEYGTYDPRTQRWNGLIGELISRRADLAICDLTITFERESVVDFTMPFMNLGISILYTKSEKPQRNMFSFLEPFTIDVWIYMATAFLGVSVMLFLLARATPSEWKSSHPCSAESEELENTLTMSNSIWHNCGSIMQQGSDIAPQAISTRMVAGIWWFFTLIMISSYTANLAAFLTVTRMDFPINSVQDLAKQSKIKYGTFERGSTAAFFRTSNDSLYQRMWTVMKQARPDVFTNDNMEGVDRVKKQKGDYAFFMESTTIEYQTALNCDLRKVGGLLDSKGYGIALPRNSPFRTAVSGAVLKLQERGNLSALKAHWWKAEGPGCDDTDDDGVQKSSDELVLLNVGGVFLVLLVGTLAAFLIAILEFLWNCRRIAVDEQMTPWEAVVSELRFVVQCGNNSKPVRPRRAGSTSRTCSFDGSYLRE

>LmigGluR1

AMVLLGARHAPALLCLLVLSEHTPAEKIPIGAIFEQGTDEVQTAFKYAMLSHNQNATGSNSRRFELQAFVDVINTADAFKLSRLICNQFSRGVFSLLGAVSPDSFDTLHSYSNTFQMPFVTPWFPENALAPSSGLLDYALSMRPDYHQAIIDVVRHYGWTSVVYLYDSNDGLLRLQNIYQNLRPGPRALNVAAVKRVQNASEAIDYLHSLEEVSRWSNKYVVLDCPTEMAKEIVVTHVRDISLGKRTYHYLLSGLIMDDHWEGDVTEYGAVNITGFRIVDGSRQFVRDFLDGWRKIDPSASPGAGRDYISAPAALMYDAVFVLVEAFNRLLRKKPDVFRANFRRGQVFNNGSRGIECNTTKGWVTPWEHGDKILRILRKVELEGLTGEVRFNDDGRRRNYTLQVVEMTVNSAIAKVAEWSDELGLTPVAAKYMRLRTSADNKKNKTYIVTTIVEEPYIMIRTEEPGENLTGNDRFEGYCKDLADLIAHKLGINYELRIVKDGQYGAENPDVEGGWDGMVGELVRKEADIAIASMTITSERERVIDFSKPFMSLGISIMIKKPVKQKPGVFSFLNPLSKEIWVCVIFSYVGVSIVLFIVSRFSPYEWRLLQPALHEPPGLAFVS

>LmigGluR4

LANDFSILNSLWFALGAFMQQGCDISPRSISGRIVGSVWWFFTLILISSYTANLAAFLTVERMVAPINSPEDLASQTEVQYGTLYHGATWDFFRRSQITLYSKMWEFMNSRKHVFVRTYDEGIRRVRTSKGKYALLIESPKNDYINEREPCDTMKVGRNLDAKGFGIATPLGSPLRDEINLAVLSLIENGELTKLKNRWWYDRTECKHGDKQDTSRSELSLSNVAGIFYILVSGLLLAMAVALLEFCYKSHVEASRAKIPLSDAFRVKARLTVGGGREFDNGRYYTPANQINYLDDEEYHTNTHTQV

>LmigGluR9

GGLFHPADDRQEVAFRYAVDRLNADRSILPRSRLSAQIEKISPQDSFHASKRVCHLLRSGVAAIFGPQSGQTASHVQSICDTMEIPHLETRWDYRLRRESCLVNLYPHPTALSKAYVDLVRAWGWKSFTIIYENNEGLVRLQELLKAHGPSEFPIAVRQLGEGTDYRPLLKQIKNSAESHIVLDCSTSRIYDVLKQAQQIGMMSDYHSYLITSLDLHSVDLEEFKYGGTNITAFRLVDPEKPELKKVIRDWGYGSYGRKADMGPIRTETALMYDAVHLFAKALHDLDSSQRIDIKPLSCDAVDTWQHGYSLINYMKIVEMQGLTGVIKFDNQGFRSNFLLDIVELNKDGLKKIGTWNSTEGVNFTRTYGEVYTQIVESLQNKTFVVTTILSSPYCMRKESSEKLTGNAQFEGYGIDLIHEIANILGFNYTFKLVPDGRYGSYNSETKEWDGMMKELLDQKADLAIADLTITYDREQAVDFTMPFMNLGISILYRKPIKQPPNLFSFLSPLSLDVWIYMATAYLGVSVLLFILARFTPYEWQNPHPCNPDPDHLENQFTLLNCMWFAIGSLMQQGCDFLPKAVSTRMVAGMWWFFTLIMISSYTANLAAFLTVERMDSPIESAEDLAKQTKIKYGALRGGSTAAFFRDSNFSTYQRMWSFMESARPSVFTSSNVEGVDRVVKGKGGYAFLMESTSIEYVIERNCELTQVGGLLDSKGYGIAMPPNSPYRTAISGAILKLQEEGKLHILKTRWWKEKRGGGTCRDDTSKSSSTANELGLANVGGVFVVLMGGMGVACVIAVCEFVWKSRKVAVEERSSICSEMAAELKFALQCRGSTKPVRRKRHAQSEEEHPTQFMPLGAYGQYDFIGKELLP

>LmigGluR5

LPDVPRVRKRNAYHHVSDFDKGRIVAYRDCGLSYRTIAAGRDPVTVREYGIGGFRRAIWIAMLDPNCLGSLAVEMTYAGSQLPRITSSRASYRVAPHRHLRQYLSRVPAASYMAMRLSASDVTGDRAATFCVALVLRFAVRHVVVHCVSPPTLSSVPCRQEEPYIMMRKAMPGETLSGNDRFEGYCKDLADLIARRIGINYELRIVKDGKYGAENPEVKGGWDGMVGELVRKEADIAIASMTITSERERVIDFSKPFMSLGISIMIKKPVKQKPGVFSFLNPLSKEIWVCVIFSYVGVSIVLFIVSRFSPYDILNSLWFALGAFMQQGCDISPRTIFRPLAPSWWTVAVAGGGMNCCMQEGNFLLVLNCFYAATIAEVVQTTSGKTISVQNIRNVLNVADMHGRSSRKKPNISEINRQKRLRFAKDYISQAMGKTTIQKMKTREWLLYNAPRRVLTPPQSPDLNAIENLALDGAAVTQQVQWGGAHATKARSRRACAVRAPGPVCSSLPASLPSADGNVMTKCAPQA

>LmigNMDAR1

MLLFTASAVTTVIFLGCVPWQADCGNPTYFNIGGVLSNNDSIFNFQQTISNLNFAEHIVPKGITYYATAMQMDPNPIRTALSVCNHLIAQRVYAVIVSHPLTGDLSPAAVSYTSGFYHIPVIGISSRDSAFSDKNIHVSFLRTVPPYSHQADVWVELLKHFNYKKVIFIHSSDTDGRALLGRFQTTSQSLEDDVEIKVQVEFAIEIEPGLDSYTDYLTEIKGAQSRVFLMYASKQDSEVVFRDAELLNMMEVNYVWIVTEQALEAPNVPMGTLGLKLVNATVETDHIKDSIYVLALALKAMNQTENITEAPNDCDTSGSIWETGKILFEYIRKQELLDGATGKVAFDDNGDRINAEYDIVNVQENQRKVSVGKYFYSHEQNRMKLKVNDSNILWPGATHAKPEGFMIPTNLKVLTIEEKPFVYVRELVHEGDTCTPEEIPCPHFNSTEEVFCCKGFCMDLLRELARNINFTYNLTLSPDGQFGSYIIKNSSAGGKKEWTGLIGELVNDRADMIVAPLTINPERAEFIEFSKPFKYQGITILEKKPSRSSTLVSFLQPFSNTLWILVMVSVHVVALVLYLLDRFSPFGRFKLANNDGTEEDALNLSSAIWFAWGVLLNSGIGEGTPRSFSARVLGMVWAGFAMIIVASYTANLAAFLVLERPKTKLTGINDARLRNTMENLTCATVKGSAVDMYFRRQVELSNMYRTMEANNYDTAEEAIRDVKQGKLMAFIWDSSRLEFEAAQDCELVTAGELFGRSGYGIGLKKGSPWADSVTLAILDFHESGFMESLDNRWILQGNLQQCEQFEKTPNTLGLKNMAGVFILVAAGIVGGIGLIIIEMAYKKHQIRKQKRMELARHAADKWRGAIERRKTLRATVAAQRRLKANGVNDPATVSLSVDALPQLETRSPARAWPGGCDVRQRGSRTDEIRVATTAQSNPAYTSNLPDMIV

>LmigNMDAR2

CCYGLAMDLLENVAQELEFDFHLYLVADGMFGTRYASPAGASAWNGVMGELVSGAAHMSFAALSVSSARSEVIDFSAPYFFSGVSFLAAPAERNDVPLLAFLLPFSPELWIAIFTSLNITAMAVAVYEWLSPPFGLNPWGRQRSKNFSIASALWVMWGLLCGHLVAFKAPKSWPNKFLINVWGGFSVIFVASYTANIAALIAGLFFQNAVSNYHDRXLLSQRVGAPRASAAEYYVHKANQLLWQHMQRFSVRDVQEGIAQLKNGSLDILIADTPILDYYRATDHGCKLQKIGDAINEDTYAVGMTKGFPLKDSISAVISKYSNNGYMDILQEKWYGALPCFKLATDMAQPRPLGVAAVAVLTLSDGRKIILKLTSNAGQLSFPKGVSRRDRRFITEDSNFSSKSIFNIFFLQKLYRFINCVELVSPHHAARELVHTLRQGQITSLFQKSVKRSVALFQKEHEQRRRRKSKAQFFEMIQEIRR

>LmigNMDAR3

MNYEKYGRSTASAQYFLQLAGYLGIPVIAWNADNSGLERRASQSSLQLQLAPSLEHQTAAMLSILERYKWHQFSVVTSQIAGHDDFVQALRERITEMQDRFKFNILNTILVTKPGDLQELVNSESRVMLLYSTREEAVHILKAARDLKITGENYVWVVTQSVIENRETPSHFPVGMLGVHFDTSSSSLVNEITTAIKVYAYGVEDFVNDARNADLSLNTQLSCHGAGDSRWKTGDRFFRYLRNVSVEGEQGKPNIEFTPEGVLKAAELKIMNLRPGVSKQLVWEEIGVWKSWQKEGLDIKDIVWPGNSHTPPQGVPEKFHLKITFLEEPPYINLAPPDPVTGKCSMNRGVLCRVANDVDMAGVELPSAHRNGSLYQCCSGFCIDLLEKFAEELAFSYELVRVEDGRWGTIENGKWNGLIADLVNRKTDMVMTSLMINSERENAVDFTVPFMETGIAILVAKRTGIISPTAFLEPFDTASWMLVGAVAIQAATFTIFLFEWLSPSGFDMKQQAAPAPNHRFSLFRTYWLVWAVLFQAAVHVDSPRGFTARFMTNVWAMFAVVFLAIYTANLAAFMITREEFHEFSGIDDHRLAKPFSHKPMFKFGTIPWSHTDSTLHKYFKEMHAYMKNFNRSTVAEGIESVLSGELDAFIYDGTVLDYLVAQDEDCRLLTVGSWYAMTGYGLAFTRNSKYVQMFNKRLLDFRENGDLERLRRYWMTGTCKPGKQEHKSSDPLALEQFLSAFLLLMSGILLAAVLLLLEHLYFKYVRKHLAKTDRGGCCALISLSMGKSLTFRGAVYEAQDLLRHHRCSDPLCDTHLWKVKHELDLARLKIRQLQKELESRGIKPSQRKKKRGRSWASCLNCNELPADRQIMDSTDVLKHRDMTRNHITSTEYSGRFDGSNQLFRPCRTEIAEMETVL

>LmigIR27

GTDLLCADHCRNHGFRRSLGGLILRAGTVVHDLPLVNLGHRLTNVEEKHVDTMARFGWELTTILSKKLNFTPVLYATDSFGYKQDTGNDTLDGLVGMLETGLVDVGSAGLAMFKDRLNRIDFVGPSRLWVSKIMFRHPTVAAEQGALFRPFTPGLWLGVMLVFAALALVSHAICRAHGWSAQRAWGDTFLLVASAVGQQGSSEGVQWPSWRLLLMVSLVCATLLDVHYSAAIVSSLLLPPPRTINTREDLLRSPLHFGIENISYAHDLIEMSEDPVIRSLYQKKVAPPGAVRPNYFTLEEGMTKVATEMFAFHSQEFQMYQLVEKLFTEQDKCVLVTIPLFPPQMTYVTVAKNSPLRETFTVGMRAMWEQGHLRHLRMRWHSKKPACMAERDFVNVDLATISPAFMLLLSATLISLLLLLRESAKARQTQDNTPVPLSH

>LmigIR20

MPSLCKASVIMTVLTLCLTSTIFCESQKSYGNSINFILDFIRHTTTASSIKGVHAFVCWDAGDLQLLKALSRNGMLASVYSGWQHWEQLPTFHMDGNSLLFILDLKCEKSFAFLKKTGRNVEFFKPPHMWLILHDAYHPDTQLFTDHSAETMLPAAKNNGKNVLKQQHNVSSYNNSIILDAMELGDEDFSSADVYDLSYGTSPKRRAGYTNSNSTHVTHVPSGVGMKNSEFTSNHINDNSAVVEAAETGFYCEVFRGLDILVDSQVTVGRRDADSKYTLLEAYRRRRQGELVVSELGYWEATAGIVWRNVREVSVRRLDLKRTKLVASIVVTNPETLDHLDDIHNRHIDTVTKLNYVLLLHVADVLNASLELLVTDEWGYESNGSWSGLVGSLQRAEADVGGTALFVTADRMRLIDYIALTTPSVAAFVFRQPPLSLVSNLFTLPYTVRQS

>LmigIR2

VSSIFALRSIEIIPDILVARQLDDVDSFELVTMKFTGEETWRDELVLARWRRGHGFDPPTVDLFPDRMADMEGRQLVFATIDYPPYVILKSDSNILDGVESRILLEKLFVVGWQKNATWRVVDHAVDRWGTIWENGSGNGLLGAVATGAADAGFAAVYRWFPEFLWVDYSRPFLRAGIACLAPRPPEVPLLPFAPPLWAAVAASVLLATIALYTARITSNWVLGDVQATGRYSTVEDCFFRSVGLLVLQTPDLERRHTRVVGPTRHVLSWLLIAYLLVTASYGSGLSSVLTVPRYDRPIDTVSDLHESGLEWAERHLVYLYSIRELTDQIYVDLIDLFRVLTSDVLHSRTTTRDLAFVIERLPGGYFTIGDYIDEEAASRWLRPMREDIYWEYVVFAVPKGWAYLQRLDDLIDRLLQAGIMYAWEGQVAHKWLVPRVQLAAQIGMRSYAQSPDGPIQLQLTHVQGEFALLGLGLCLALVVLLVEVAV

>LmigIR26

MLGGKAGSVDAGATNLRQNVSRVPTCKRSCCPAADDDVAEALMFEIITITITQHAEYLITVTSTQTDNLHERLISENQKHLDPLARFNYALFLHLKEFYNFTFSVQATRSWGYKTKAGRFDGMMGVIQRNEADIGASSALIKKERLEIVDYAGHTWKFWPRFLFLHPSGQRLHTALLTPLSTKVWFCAILAGLMITLILNTSSYVHADNFCNLDGSWSSTLITTIGTFTLQGAGSSWSQISWRITLLTALLLASLLNIHYGAAVVGSLLIPAPHTIRTLQDLMESPLRVAFENVSYNREYVARTTDKLGRELIHRKKPEFVQLSEGVIKIRKGFFAFHTEGGSVFRLAAMTFTESQKCALSDVSLFTPAVMSMPVKKKSPLRELFARGWSTRRPNCDFLRQSLPMDGPMVEVKKIDSTNPWLKL

>LmigIR25

LHNGTYGGMIELMQRNLSQVSGSSLIMKKNRWDVVDYTGPAWRFKSYLAFRHPTSQGLRSSLFAPFCSSVWVASGAVWLLIMSSLRLITWIGARQAGGIVQAESSWGAVVVFAACAIGLQGTTERSQWLSWRVVLLFTFLLSLMLNTYYGAAVVGSLLVPPPKTIRTLRHLIDSPIQVGIEDIGYNRDYLEKSTDPLVRELFVRKVFPPSAKRPHYYPIDVGVERMRTSLFAFQAEAV

>LmigIR11

MSACEVTIGADRIGVLTFAQPVRNAGCRFYFQTQFQEVMFKPYVQPFEDRVWIALLGYVVATALLKWLFHYVLRERADPITALFAVIAALLNQASGDPRRCAVRVLYFSLCALFLILRIGYGARLTATSTLRQAAPPYRRMEDILASDWGVNVISNSFALESMQMSPPNSTAWKLWQTKIDNNPYSTVETTEDGLWNVLDKKKLAFFGFEDACRDVLHRRFSPDQSCRISELDGVFFKAPLSFALPRDSEFLLTINYWILRMFETGIIDRLSRKWLPKPPVCDETKYEAETFADVLPMVILFASGFCLAIIVLIGEICVYSFKNRNMKVVSKNRKVRKTQKNYLYPML

>LmigIR28

LEALYRVHPSRPLTVRQAGSWTPHQGIVLHRSAVHNGWRLLDGLILGAAGVVVDTPTDRLGERLRVVQDRHLDTNIRFGWDLCDKLRFMFNFSVVLYKTESYGYLTPNGTIDGQAGMLCDGTIDLALSQLMLSHHRLDFIDFTTAPSRMWTLKTVFRHPASRAVYGTIFRPFSAALWLSSGLVFLLVLVAARLGCWAAAHNPADDSWSAAFLLVSSAISQQGTTLDTGRPSWRLVVFLSFSCALLLDTYYTAAIVTSLLLPPPRTINSKADLVHSQLAVGMENISYTHEYFEKSSDPVDHALMKYKVWPAGTARPNYHSLEAGVRKVAAEAFAFTGEDVSLYPLLDRYVTEADKCALVALDFMKSRSTYMPVRKNSPYRELLTIGLRRLLERGHLAR

>LmigIR19

MCINKPPDGELSYKIHIYDIGKESDYKYYARDTVVRTDGNFQMDMNGNRELFDKSILQLLQDILEFSLLCVEYHEIKIYLIKLGHIQYGRVRRLKVSNHNGKKYSRDLSILRQLATDKIDIGSTTILLTYDRIGMADYTIGIDTFNSSYVFVKNPAAENSPVTGINASRKQFSLNMLEQPSWDLSEVMLVTIGALSQQGADRHPNTMAARILFLVLFLLAVLMYTAYSASVISIMSSTKPVSNSLQGILESSSKMSVALHDIHYYHTQFQIERNPLSQNLHKLELVPEFLSLEEGLEGALEGNIAFSCGRVDAHTYLQNSQHTDESLCGLGEIPILRGMGYQRSFALKHNSPLRRAFDRGLLRLMEHGLIKREWHRYFGKRPASELHCEETSSSTGFVRITLDDVWPAVKMFGVGVIIAILLLPTELLVRQWLVLLEGNNLCENVLRSKMLTPIIK

>LmigIR25a

MEAGVTWVPDLQAETPEGVPAAAQGAPSAEKVHVNDDKNSIPNKALKQVVADLSKQGMIFDGVFKATANGSDVEALIDSMCLEYNTSIDENKKIHVVLDTTLQDVSSEAVKYFTRALELPTVSASCGQEGDLRRRLSWWENMYKKNLSTICLPRCVCVCICVGVGVFAYLPFSPQVIKTLLFSLYAVMDHKYKSLLQNVPTRHIINEIKFQNIANQLSTFKQREVFNYFILGRMDTVNKVLEAAADMEFYGRQFGWYAITQDEGNPSCQKCGKGASVLHVKPNDAEGTVVGAENPKLAYQFYYELFRNTFLAIGQMIKEESWPDMQYIPCEEYEENKNVPPTRKLNLLEALQQISMLNPGAYGQLMLSSNGHSHMQFNMTAFNVSLSDNSATEVGTWAADLDSPFITKVKPSVPVTQYTVVVALQQPFVIKYQDENGNTKFKGYCIDLINAIRNITNFEIEIYEVADGKFGNMDEEGRWNGMIKDLIDKKAHIALGALSVMAERENVVDFTVPYYDLVGITILMKKPKTPTSLFKFLTVLENDVWLCILAAYFFTSFLMWVFDRWSPYSYQNNREKYKDDEEKREFDLKECLWFCMTSLTPQGGGEAPKNLSGRLVAATWWLFGFIIIASYTANLAAFLTVSRLDTPVESLDDLSKQYKIQYAPIANSSAHGGGEAPKNLSGRLVAATWWLFGFIIIASYTANLAAFLTVSRLDTPVESLDDLSKQYKIQYAPIANSSAHVYFQRMAAIENRFYEIWKDMSLNDSLSEVERAKLAVWDYPVSDKYTKILQAMTEAGFPANIEEALERVRASKSSSEGFAFIGDATDIRYQVLTNCDLQMVGEEFSRKPYAIAVQQGSPLKDQFNNAILQLLNKRKLEKLKEQWWNQNPEKRNDCEKQDDQSDGISIQNIGGVFIVIFVGIGLACITLAFEYWWYKLRPQHNAVVEAAPPRTKSDSLQALNMMRSSFDKRYGRRQGVALAGVTNPW

>LmigIR4

EEDKWGAIWENGSGNGLLGAVAMDDADAGYAAVYHWYPEYHFVEYTRPYVRAGLTCMAPRPRPQPGWQRSVDMLDNRDFVLVCYIITRCLLHVVPMNRVIGWIESSLDACMVLDTRLLTQRRCLLSSPDSVVLWGHRLQYLHLHVGLYHIKYYSHLVTIWSSLGVTLSALFGGRLRNLLCLRRYEPPIDSVVDLRDSGLEWAETHIAFLNSLRGRTEQVYLDLVKQYRVLSVDVLKSRTTTRDLAFIIERLPGGYFTIGDYIDEEAASRYLRPMREDIYWEYVVLAVQKGWPYAQQLDELIDKLFEAGIMHAWEGQV

>LmigIR76b

MQVSPVLRTVLTTVCSNYFLNGTKMEVPEGEPDPGCVLRIPKLMEGKTIRIGTLENPPLTMINKTDGTLIGHGVIFEIVDILKHKLGFNYEVVTPRANILGDENHGIIGLVHSRQVDLAVGYLPQFSQQARLVRHSESLAEAPWVFLMKRPLVSASGTGLLAPFDATVWYLVLASVVLMGPAIYFIILVRVRLCAGSERLTRIFPLSSCVWFVYGALMKQGSTLMPVTDSSRMLFATWWIFITLLTSFYTANLTAFLTLSRFTLQITSLKDIATKKAHWAAQKGSAMEYLVYNNDEYSFLNQSLQAGFGQFVDISDTDMLLRIKKDDLVYLREKQHVEHTMFRDYLEKTRNPKVEEKDRCTFVMTKQPFLHLPISFYYPLNSNLAHIFDPLLKALVETGIVRHLLRKDLPQIEICPLDLGSKERQLRNSDLYMTYMIVVTGFCAATVAFFGEILTRQVKRCIAEAELQTGPSTSYPDDWKTVKAQANRMPYTMYLNGNIINVKQPAYSITKDFQSSNRKSLSRQRNTNYVFQYTS

>LmigIR21

LSLVSNLFTLPFSRAVWACAAALVVTCTVLLLAATRWERTRGDPQLADYLSQFYAVAGQLRDKWGEVAMLAVGAVCQQGSPAESRGVPGRIVTLSLLVTVMFLYTSYSASIVVLLQSTTSSIRTLADLLYSPLGLGVHDIVYNRHFFPAADDPVRRALYRQKVAPPGAEPRFMTLEEGVRRMRTEPFAFHSELSPAWQLVQETFREDEKCGLQAIPFLQLMHPYIAVQRGSAYKEMFKIAYRRLWESGLQHRQLSRLYTMRKPRCAAGRGSSFVSVGIADCYPALLVPVYGVAVAIIVVLAEILFHRRVEVLRW

>LmigIR3

FELVTMKFTGEETWRDELVLARWRRGHGFDPPTVDLFPDRMADMEGRQLVFATIDYPPYVILKSDSNILDGVESRILLEFVSKKNATWRVVEHAVDRWGTIWENGSGNGLLGAVAAGAADAGFAAVYRWFPEFLWVDYSRPFLRAGIACLAPRPPDIKGGFEPPTAYSLTLRIIAPQCTRPLPRAPCPPWSDMSVQYTISSSYSGRYSTVEDCFFRSVGLLVLQTPDLERRHTRVVGPTRHVLSWLLIAYLLVTASYGSGLSSVLTVPRYDRPIDTVSDLHESGLEWAERHLVYLYSIRELTDQIYVDLIDRFRVLTSDVLHSRTTTRDLAFVIERLPGGYFTIGDYIDEEAASRWLRPMREDIYWEYVVFAVPKGWAYLQRLDDLIDRLLQAGIMYAWEGQVARKWLVPRVQLAAQIGMRSYAQSPDGPIQLQLTHVQGEFALLGLGLCLALVALLVEVAV

>LmigIR24

MKPPAALLVLLLVCQESSGQDSYFTDVISLTRDYFVAKRVSLVTVYTCWNSWLEKDLLRSLWDRGLRASRLPLAEAAETSCGGSHGAALASLEAAGPYRSGVLVDVACPGGQQLLTKASARRMFGIQRHWLLVDSSGNSTAGDEPEFPALPARWRDVLSSLWMMPDSEVVWMGAADDGAIQLLDVYRLTSFTPVLNISLAGWAVRRNSGVSLYLLPRPDTSKRRNSLHGAKLKAGAAILFPKYFTGMYDLRLPHLDTWTKITYPLIEYLGQNFNFTMEVFYTDSYGWQTNGTFDGVIGMMQREEIQIAASSLFMRRDRMPYVDFAAEAFYLKTAVVFRQPTLASVANIFTLPFSAAVWACCLLLCVLTLLLFGVQLRLAAKRGIEGELTHVTYAELFTFVLGSICQQGLPQTPTSLSGRVTVFVLALTSLFLFTSYSANIVALLQSPSHSIRTVSDLASSPLTLGVQDIAYNKVYLGETTDRELRQFVRRKMQPLGNRVFYNGAEGMERVRKGMFGFQVDTSTAYKIISETYTEREKCGLMEVNLFPLPPLCVATTKHAGYREMFSQRVGWQREVGILTRQRRLWLPQRPVCENMVSGFVSVGIMDFYPALLVLQYGVAGAVVVLALELLYFHRSRLWQRMICISQLSGAKPTSMYAVATDQISQKRSQLQRTGSRLK

>LmigIR8a

MWPLWMSVVAAHLQLASSQATSEPLLIRFLLVTEVNASWVGGELRANLSGLEARYVGLRLQLDLSAVEVDREHEVEEFQQKVCGALASGVSALLDATWTGWRRLRDEAQHRGLPYLRLDATLANFVDAVDKYLHAREASDAALIFHTEEELDQALYHLIGNSVLRVIALNGLEKDTVSRLKDMRPVASYFVIFADTAHMAELYSKAAAGGLVRKAERWALAFTDWDWRSFRTDQLNLSTALLQMKPASCCALQGEPDTCKCALRKVAPAFLRAALSAAVDTLAELHSKGMDVRATPRQCSSTGDGGADDEDGDGAGEEEESAEPPANATGNYDAFLRAIATRGQSNSTLFFRAATAQLTFNTPLQLRMVNRSEDVSLGDWSPEKGLQLERQLKPAKRFFRVGTAEGVPWSFPVRDEKTGEPLVGPDGEPIWDGYCIDLLKKLAEPTHMNFDYELVPAKDNDFGSRSPSGSWTGLVGDLAMGETDMIIAPLTMTSEREEMIDFVAPYFDQSGISIVIRKPVRETSLFKFMTVLRLEVWLSIVGALTVTGIMIWLLDKYSPYSAQNNKEMYPYPCREFTLKESFWFALTSFTPQGGGEAPKALSGRTLVAAYWLFVVLMLATFTANLAAFLTVERMKSPVQSLEQLARQSRINYTVVLNSDTHEYFRNMKNAEDVLYNVWKDITLNSSSDQSRYRVWDYPIKEQYGHILQAIDQAGPVPNASVGFQKVIDQEEGKFAFIHDAAQIRYEVSKNCNLTEVGEMFAEQPYAIAVQQGSHLQEEISRQILDLQKDRYFESLTAKFWNNSAKGTCPNSDDSEGITLESLGGVFIATLFGLALAMITLAGEIFYYKRKKLTAVNVTSSSAKVPKKQVTIGKEFRPVMDKTAPRVSYISVFPRNQLY

>LmigIR1

NASWRFTVDAEHEWGEIWDNNGSGNGILGAVVRDAADAGFGALYQWLHEYLFLDFSRPYIRTGITCLAPRPRPGWQVPLLRPSPPLWAAVTASVLLATVALFAARRSSDRLLGEDAVRAAAGSRYSTVEDCFFRSVGLLVLQTPDVERRHTRVVGPTRHVLSWLLIAYLLVTASYGSGLSSVLTVPKYEPPIDSVRDLYESGIEWAATHEAWVFSLREATQPVISDLYRRFRVHKEDVLHARTVARDLAYSIERLPAGYFAIGSYIDEASRWLRPMREDIYWERSNVVVRKGWPHLPRLDALIDRLLDSGLLLAWEGQV

>LmigIR8

LCGKATLADVTRPAQSIIQTVAPVTTEKAAAPLQEPHVCLASPQIPIRCGCTYGTAICIVEARKPPHLGNVCGSWEWDTAVKFHKVNKRLNIFEQTNLHWTVDKRRKTRNERCSLSLLSRKNSGLTSLETFANTRRISAAEVWEMTSRIFFHETINSDRIVDWPPRYADLNPCDLYLWGMLKELACREMNFSSGTVLTYDPFLVKDSIWSDSYTPKLQEVKETADIYYKKYMNLNGNNVRASMFTVNPSAIEDNTTVSKFRGCDAQMIETLAKYMNATLVMLPNDGSGFGKWNGTVNTGTDGDVMFDRADIAPNTRYVIVERLKVHGYTYPHDKEDLCILVNKSPRIPQYLNIILPFALIAWLTILLSLPFSALFWSLIRRFGARPSNSEPFARVYIGSFLKIFSAFLSVAVSALPTVGRERILFVMWTFFSLIITNTYQGSLTSYLTIPKYMPDIDTMEELSKSGLKILIHPELLPVFKLDTGNPVMDALNRNLVSDIDMEGYPEKIQSNTDTCALVNAYVGQFLIRSRHYVINGFPLLHLTNECPMPAVVAFATPKFSPLQPRFDVLIRRIVEAGLYKKWQKNMLDESIASGDLLLISNREGKADPERITLSHLQMPFYLLFLGYFLTSVLFLTEYLRLKMNTRSTNEEEAKLERREGCQTLENDSASYLLDV

>LmigIR10

LDVRLLASTHGLCRSAVADVATSILRSHHPRPAVVSTGPTYLPPATTFLTPLQNEQRLLVTDSASPVVEFFQFGRAHSRLWSATNDFIILLLKAYDHDGGDCNADCANLLRHLWSAFGAARVLVRHVEVSDGCHVHRLGPTVALFDPFDTSAGTIVSTTWTNVELIKTLLDNRTRDLRGRPFRVCMFARNYTALPVDANGVLVYRSSLETVKYAGVDGWALQTLAQYMNFTTVFRQPYDRERYGELLPNGSFTGALGELMRGRVEIIFNAIFMKSYGSHEIYFTKGVSKDDIRLVVPKGGLLSKRHLLTMLMRPEVQLSILVTYLLMTLLWYASSRLHMFFKPEDAERPDILMIAFQNFHPPTSSALEKLPRHAAERFLVVSYLLFHMLVFTIVQGKMLEVINNPRYEDDINTLQELVASHLPIKTGSATLIDIFGPTGSAPQPESLLQSLSRKTNENHQKGGRKPDGNLRFNVMIFRAYVSASFVALLLSTGHRHSSVAPMAFTFTVLGNECIKELDDVIDNMVLLEKGSAVEEVARSRDAACVVRDSDLRHGKYSAYRRADGSWLLHAVQEAALSFELGYATRRDSPFLAAIDRFLWRAVEAGLSRCWERRGYLLVTLRARADPASIDTAAAAAKTYKQFTVSDLQMPFYAWIVGVASSVICFAAELLYSWWLSGHKN

>LmigIR7

MLAWVAVTSFFLWPDVVARHQPAVVTNSSLEDCLAYVFSSQPFTWAPLLLVTTTSPDDREPDVSAITNAVVHTVQLPVVLVKLPTTSVYADLVAGYIIVLPDSDLDQDTFQSILVNISYVANWSPRRPVLVIFRAGYGRHEDSVRMILDCLDKRNAIEATVFVVNVERTSWEVFGYYTVRNISAYTWFPYRQGHCRDDGVETELIGNIATTGRHNFTVFPEKLPRSLHGCSVVAATYHLPPFVSKVESEGRQTSFSGYDVILFRLLARAANFRPVVSAPHFSGPQWGERLENGSWSGVVGRMLRRQADAAFGGLHDNAXXXXXXXXXXXXXXXXXLPDALTWFAPRARRACALCEFGLIVPARDAGLLLLGALLMSVPFKLFSGQDEISALRRLDAAFLFALRMLVTTPARAPRTLRPRIVFIFWLQLTYILITGYQSTVTSKMVQPGTEEQILTLEQLLRSDLYCGTLLPFQRWMDTVMKSKAPKISYLCPNIADCMFRMAYSKDLAVMTTKVHTEFMGKVKYVDQAGRSLFLPFTENLFTYYVAVHVQQGSPLLPLFDDVILRVQAGALMTPAMREYERLFRYGTVDHSDLPEEVDSQGFIALSHLSGAFTAWAVALATAFCVFVAELAYWRSGFRLFARQLRCSRLPEKEKDKNAFGSDNFITEKKGRKLRL

>LmigIR9

MQLHPSPCENWTEPHVWIHYPYRKENPMLRVPASQFVVVETLATTHRYWNLEGAHISAVMYPAYPTAMPAGQREINTKTCDGDTTGYIELHANSVRIFKYVRGSDHFLRLTLEEKLNARVVARYPKDENFVEGEELPNGQLTGALAEMASGQAQLGLNLRFVRPLPTPEILYLQPVCSFARVGIAVARAPLAPLHVAIMRSFDLKVWFSVAAFYILSAMIWSLLTSSINYKSVLQEIQKFLLIGCGSFAQSPSQKFFASACAIFSLIIVTIFQEKEKERKGEEEIRKELGKTNGSLVNVLTTPVYDRDINTANELLQSGLPIATNRTSLIHMFQLAAEGSPVMTELCKHMKPTDKHNLTELLFDRKQALIIADAGLAQLKNHHPSQEKMLHMIKDTFIVLQRAYAARTDCPVKEQVEKIIWCLTEAGIPRYWNTQASRSEFSNCPLPEDTPKPFSLKQFKIAFYTLCIGLLFSSFVFLLEIISYKRCQKHILK

>LmigIR14

MRRGAININGDYTGKKVELAETSSKMGLDVLAFSDVRVRGEKEDEVGEYNVYLSGVKAGRAQWGVGFYIRKAIEPSIIAIRPFFTWQELLKTRQSEFMWLLLLRRDQSPSAVLQDIDIRFDTLFFVAVPTTESDVHIVEAYRVAKGYPLVMQTFCTWNTTYHPSCRTESTIARRGDLMGYKMKSGIVEHSPMVQEGLSNLMRPPRHELASRYQFKEMSGWGIRSRDGSWSGLIGRLVSGNLDVGVAYKLYIQTPSEQEFIWGHFLWPFDKWLWLCSFALVVTTWLVLRVQERFTRDDESEISARGTYNDFLQVLGIFLMQGVYGGRTAPRACVRASLCAASLLALLLYTAYCGALTASLASRRPRLPFRDMAGLLRDASYSLVTIADSSILNVLSAERQAEDEALRGGSDECGPQMEHRQPPVEREILNPVHSESSLRSHLMKDSNNPVDRKIYEKHIEGKHMPHTSEELFGWLCSSQKHAMLVSKTRYMHGTDFVQCPVTSVHETSGSQEGFVLTKKSPYREAINYHHDCHRDGEASGSDPPLSSKPMDPINPGNYATNQQLCLYPVCEGSSLHQFRQLPIGTEDVLRTQATSIIDADVSHDLQSTAPHNLDSRQQDRIHISDEAPRQTH

>LmigIR15

MFIGFEQYASCKIPIKTVAAVVSSLQDEIHFGCVYLTYSSREAELLPQSRFHVEFRRGLRNSIQLGFLPDTHETQLRHNCSSSPLLVVPHTDLLLNHSSVIKDLLRKRQSDFMWLLLLNDGNMDAALADIYVEFDSNFFVIFESTDGTVVIVEAYRVAKDFPLVTKKFCTWQINTNLTCTKEGIRSRRNDLMGYELKTGVVEVWPDMYDLGNGSCSGYLCDIWNILSRKLNFTKLAKHNGHTIIRKPLNVSNTVAIEEEEKEARQRKINVWIHSTWITKPVQKEFRTLFPHLMEPYGPAAVAELSAARQSQSARPLQSWVRMRPRAWLFVPSLLLVSSKQTRWVKDGVSQQRVGFESGELMLLFLRMQNSQCGDHLLRLLQENLFGVRYRLQRSSLFGSRLSDGSWSGAVGLLAGGEAEVALSVMMITPSRLSAVDFSLPFFSTREYLYIREPEEEDLAWDGFLRPFDGPLCVCTVAVVLVIWGVLKVHHRCAPSAGTQLDEGRLYSDFLHVIGIFCMQGCASASRDSARLSLLVASMCALVVYTAYCGALTASLATHQPRLPFTDLKGLLQDGSYQLLLLDGSGEMTILAIWPSCAFVWSPSHIGVLVNEHVNRLAKQATTTPALEIGFTESNLKLALHLKESEDPVTKEIYNNMVVKDDLPEDSNEAFDRLCSSEKQVLLCQLMDFRLNENSLKCNVTTTQEYITSQLAIAFRKRSPYREIINYQLTNYEWGAQPSVVRTSVLAVCVSASEYEAPVWDASAHTKQVDVSVKETLRIVTGCLRPPPTGRRRHQGLKEACGESALPYRTVARWVKAFNEDRQTVAATHRAGLPSAPEEDCILLPRWRTVIDARRFVNSPMKPD

>LmigIR6

MRSLLIVTMLVNSMCDGRLVLQNNDVSSIDSLIRELGSRILSKEFGSYKCVAALSDVPEAVAYFTQWFPTTVLISLVSGDVSAEDLLVSALDADCQGFLVRCRDAAAGVDAILKASKFAMRRVNRRLLVLPTAQSPHINITAIFDLRNIDLVPEIVVARPSGDNSFDLVTLKLTGEDTWRDELLVAQWYRGRGLQPPSADLYAHRLADLGGRRIVISTVDYPPYVIFNSGDNVFDGIEARIIIEFIKKINVTWSVVEDTTYKWGTVWCNGSGNGMLGAVAADEVDVAFSALYRWYPEFMEYSRQYVRSSVVCLAPRPLMLAGWQVPVLPFSPAMWAAVGFSVVVATAALYAVKKLSDWVLGSDDDPSGGRYSTVEDCFFRSVGLLVLQTPDVERRHTRVVGPTRHVLSWLLITYLLVTSSYGGGLSSVLTIPRYEPPIDTVTDLHDSGLEWAQTDIAYLNSLRGLTDPVYLDLIDRYRVLTPDMLRSRVTTRDLAFAVEKLPGGYFAIGDYIDEEAVSNWLRPMREDIYWGTVVLATRKCWPHLERLNDIIDRLFEAGIIRAWEGTVAREWLVARVQLAAQVGMRSAQAPDGPIKLRLTHVQGGFALLILGLCLSLLLFFLEIFVYWKSNGSTRLEFSSTIKVTLEHYRS

>LmigIR13

MDRMCDCCVRAQEELAAVREQLSVLLAAVSRLKALGGSGASHGTPQVVLASPTIPAVGTSSRVPDAVESPSPQGVWRVQRRSRRTGRKVNVEAGRAATPALPNKEFCTKSIWLVFLRGDEILEKAFEDMYIPLNCQLLVVHQYGQQHFITEIFHLKEKLHLRKVLYGIWSAEHDLQKTADGFYSRRCDLLGETVKVSTFKSYNSEYGLSQLRLLSETLNFTYKFVKVKEMFPGRLTASGYSGMLGAITRREVDMTIDLLTHTTARSHVVDFLFPTQKDTHSMFIKLSLDEGIPWTSYLSPFCGRLWGTVVLIITLDAVALAVLLRSSGHKVWTSDFLHLLLDVLGMYSLQGLKGSTGTDRPPCHPPPEVVIGYEIAKDLEEQLNGMENVLKGTHEMNINKSKTRVMECSLTKSGDAQEIRLGVSVGSRTSAAQLVSVSAYVTAIVLQAAYCANVVSVVASRRYSPPFSDLAGLLADRTYRVAVMRESITNDVFEFAGDKEMKLVYQHFIEPHNNDMPVLEEDIDVILCHRYRYCFTGESKLVDSHTVSCEIFEVRLKTPPSHLGFALWKGHPYKNIFNYRYTIRIARLADVTRPAQLIVQTVAPVTTEKAAAPLQEPHICLSSPQIPLCCGFTYGTPICILEARKPPHLGNVFDYDPALVPFLTDRLTRDENSTNGIEDDTSNGEGD

>LmigIR18

FADSVNSVWLEKNLAKQRKALTFAAHNWTSGVRRCPRNKVLTIADLSYVATPGVFTKGVRRNVTWLVAANGGEWRRQLERCYVPLDALLLVAEALSAGQLVEITEVFSPRRGRPPTFAFFGRWTPATGLYTKRDDVTALYRRRTSLDGAQLAVITYHDPPSVTVTQNETHTTVGGYFGEVWNTLAKHLRFRTQWLVQEWPNPGYNRADGTWDGAAGALLKGEADVALVATTMRPYQGTPIAFSYPLYYSSRSDDIEGRPGVNSSYSASGAGRDRLFVRRMDSPRSSWGDFVAPFERRLWVAVALSVPALAAALAALYRLGRHFGTADATGPYDYSFYDSLLYVFGAFCQQAIITFAAYSAALISSLTVVSDDLPFSDIEGLLRDGTYTVGVLNRTETFHGLMSPPGNTNGGLFYLRNIFTREDALIIYYTVELHVLGILPYPIKGSNIPRSNLLLPLFRNHTFVYPLHKHPSVLVVPTRFIALEVKSSKVFKVLKTRENQVGRHAKTATWSNKEMVSLLMPKHVLIFLALLVNIEVSLYEGC

>LmigIR29

LLKDLQGLALGAALPIVDTPLDNLGERLKNVNDRQLDTMARFGWGLSTTLAELLNFSIILYRVRNFGSLINDHEMDGAVALIHNGTVEFGAAGFIMTTRRMDFMDYTGPGRLWAPEIMFRHPKSASVLTTIFKPYTAELWVSSGALFVLILVVSRLFCWVEHKVTATVDEIDNSWNSTFLLVSSAIGQQGVSRSSEWLSWRMLLFVSFLCTNLLDTHYAAGIVSSLLMPPPRTINNKKDLADSTLGFGLENVSYTYQFFVKSDDPVDRALCSRKLYQAGGRANFFPAEVGVLKMAREPFAFHAEDVRVGPLIDRFFSDDDKCALVFIPLLTPVATYTAVRRNSPVKELFNFGLRMMWERGHVNYLRKAWYFTRVRCLSETEYASVDLVPMSPAFMLLGCAFLLSGFLL

>LmigIR12

LQPEVSSHSTDPSGKKFDGHRFKKDTNVNRCRGGVAKRIKALCSDERGHSPLGDYMAFETAWQIKIVCRNRDSNLGPLPFAGKRTTDCAIPTRLTTPPHNFTSASTSPPSSRSASLVTSSALPKNWYEISGKCLSCVHATVSQTYQKLDKVLRHVIATARYEVSTYSGLVPGNQKEDKSWTGVIGLLQQGSAEIITDLLTVTASRVNAVDFSSPATTDKFGLFIKDEVYTDVNRWSFVSPFETTLWMAVIGTILLYMLCVTVMNLASYDKNPQCSTKEILMGIFAAFCLRGYSLLLPRWSLRLAYLSAFITAVVIHAAYCARVVSHLANSNRSLPFTDLEEAYAAGYEIQVVPGTSAAETFKYASDGIIRTINEEMIEPRYFYLPVTINEGLLHMCNWKKTCFVSERNSVRCSQKQPCGIIEVSVTMPSVYLAFALRKKSPFRRIISYQMEKLRTGGILKRLKSTSCNRAQADQGSDFKRVQLKNAAPLLAIIYFAVIISFLVVILERALFRYTQKTNSKSVPKFRKYRKRIRQTPMYLP

>LmigIR22

MDEGHQADCVFLDFKKAFDTVPHCRLLTKIRAYGIGSQLCDWLEDVLSNRTQPAFMFREPSLAAVGNVYTRPFSRGVWLSYSLAAMLLALLVVGSQRLLASARGLLDDMTDPAVAPPWADVPLVGFSIICEEGASLVWGYELLSAFSAVHSFFNKHTVYYNPLSIFVFEIFDFEIRESFSGHPVFSTRELDQFLYQRTSKTLQEINNFFSSMILSVHSRRSPIEFPEHFRDNRALVEPTSNEPSCSPLNCFYISSKRDRKYHKNNWMYFKVRVNPYCEGYRDTFNINARPTPWRWSGIRPPPQNVSSRILLMFLLVLAVFHVTAYSACIVSLLQLPSGSINDLHSLFGSKLRVVMQNLPYNFNYGNTHLVTPLCLAVKETKDPLTRSFYQERVYPQPYGKVFKPLEDCVALMRGGRFACHADEAAYKVIGDTFLEAEKCSLKSVPMFPLRAIILGVRKHSQYKETLSVIQAWLRETGLLKREWTRWVAQKPRCLNRDSGYAEVGLTEVSPALLMLCYGVAGSLVALLLELLLHRAIAARPGRHQRQPKTPARGNALVNRAFLR

>LmigIR17

MFFYLLIRDVATGGIREVYRFYVGHAVLYFNPYPLSKNSTQMDGSQLTVNEDGVINDVFPAGELWKFIAKRLNISTQLIIKKDPSCGSYDHGQWSGMVGDVHEGKAHVGLSLFTMSASRASVVSFTTPIFLGRYFIFIRSDEITHVGPNPLELLVPLKHDLWLAVIFTLFAYTTVMAGIFYIQRKTLCDAVFTIIGAFVQKSGKNMAADRIIRFGPLDGITSYHQRITSEQCLRFAAYVTGSVLLSAYSAGIISILTITEPKLPFQNFAEIEEDGSYMLGVTDNSFEYDFFKLSQQKLLQKLFNHQMDIHNLPRSKPEGLSRVCTGTKYAFLSTDLSVQSSWSQQNCNVIAVPIDMFRCTLALILRKNSPYIGGMNFHIQILRGAGLLNHMLILYNIRDTSFHQLYAERNIMELQNIFPIICVLVLGIIFSFMCLIME

>DmelIR100a

MATTLQLIMLALVGGTLGQANNTDHKQVLTSIVKQLEGGLELHLRTSEDGGNDLVQFLMQEKSSIIISAKQEEVPSRAKIMRHHFFIFDGVHQMQEIRTSLFNTDGFYILALENNTIEDDVLLMEFAADVWLQHGHSRIYYVQLSKKSVLLFNPFLQRLVVVQDSKTYSRIYKDLEGYHLRIYIFDSVYSSVIGDGENKVLSVTGADAKLAKTVARQLNFTADFVWPDDEFFGGRLANGEYSGGVGRAHRGEVDIIFAGFFIKDYLTTHIQFSAAVYMDELCLYVKKAQRIPQSILPLFAVHMDVWLCFLLVGLLGALVWLILRAVNLILGIEGVPDGSRATRISYFGAARRIFVDTWVIWVRVNVGRFPPFHSERIFVASLCLVSVIFGALLESSLATVYIRPLYYRDVNTLRELDESGQPIYIKHPAFKDDLFYGHNSEVYRRLDAKMMLVAEGEERLIEMVSKRGGFAGVTRSASLQLSDIRYVMTKKVHKIPECPKNYHIAYVLPRPSPYLEEVNRIVLRLVAGGIVGLWTGEAKERAKWSIQRFPEYLAELDVGRWKVLTLSDVQLAFYALTIGCLLSAIVCMAEILLGRQRRLHSPK

>DmelIR10a

MAVLGTVFLLFMLDLKTLNLTRLNGLLVEPTRDLPQLELWLRAGSDHQDAENPYVQWFLLRTEIPLSIVTYQENRYWMDDPFGRRNLVLVMSLDQLLTNRGAAAPIQKASTFFYILADQDKDLSADEQLRLEGSCRQLWTQHKVYNRFFLTRDGVWIYDPFKRRDSAFGRLVRYYGSETLDKLLFRDMAGYPLRIQMFRSVYTRPEFDKETGLLTRVTGVDFLVAQMLRERLNFTMLLQQPEKKYFGERSANGSYNGAIGSIIKDGLDICLTGFFVKDYLVQQYMDFTVAVYDDELCIYVPKASRIPQSILPIFAVGYDIWLGFVLTAFACALIWLTLRVINLKLRIVSLGNQHIVGQALGIMVDTWVVWVRLNLSHLPASYAERMFIGTLCLVSVIFGAIFESSLATVYIHPLYYKDINTMQELDESGLKVVYKYSSMADDLFFSETSPLFASLNKKLSWNRDLRADVIDEVARFRNKAGVSRYTSLILESSHFTLLRKIWVVPECPKYYTISYVMPRDSPWEDAVNALLLRFLNAGLIVKWIQDEKSWVDIKMRSNILEADAESELVRVLTIGDLQLAFYVVIGGNLLAFLGFLAEHFRWKLQKKGV

>DmelIR11a

MRFAILWLFSGCLLPGIQVGIWVVVRAQPTGRDVLLSRLGNQQNELNTRRLANASSYLTRNYIANRINTLVVREICVECPYELSERQRQLVDQILASLAPELSVLLHKGTAEETTWEYTLFVVNDHTAFTGQVFIFPDELLEREFFCIVVVSEIQSRQFVRQTVGSIVKSNLQMHFVNVVVVAQLEDGTVGTYSYKLFKANCTPGITVRQINHFDRITGKPQQSMPDLYPVRNGHLGDCPFNVGAAHMPPHLIYKRHKDPPPASNVSIPAEDLAGIDWDLLQLLAKALKFRIQLYMPQEPSQIFGEGNVSGCFRQLADGTVSIAIGGLSGSDKRRSLFSKSTVYHQSNFVMVVRRDRYLGRLGPLILPFRGKLWGVIIVILLLAVLSTCWLRSRLGLSHPIEDLLTVIVGNPIPDHRLPGKGFLRYLLASWMLLTLVLRCAYQARLFDVLRLSRHRPLPKDLSGLIKDNYTMVANGYHDFYPLELTCRQPLDFSARFERVQRAAPDERLTTIALISNLAYWNHKHPNISRLTFVRQPIYMYHLVIYFPRRFFLRPAIDRKIKQLLSAGVMAHIERRYMQYENKRKVASNDPVLLRRITKSIMNGAYRIHGLVIVLATGMFILELLAGRSNGRLRRWMEWVHQ

>DmelIR20a

MLASLNRSTGLSAELLDLYGLVVHFLLSGEHTTLVYFNPAGLDCSWGVLWQRNLTAHPQIVWQRNYSYPDLYYQFNAKLLVLACLPMDSRAAIQLEILANSLSHLRTVVRLLIEVAGPDQVTLARQYLSFCLRRSMLHVELYFRDYHHSLILYSFRAFPSFELVMRWISVGQGVKLFLHKLDDLRGHRLRVIPDLSPPNTFFYRDARGDNQVTGYLWDFLATFAGRLNAGLEVVRPSWRAGSASDSSYMLEYSAKGLIDVGLTTTLITKWNLWAIHQYTYPLLVSSWCTMLPVEKPLATPDLFGRIVCPTLAMTLLLIILVTWLVFRQLRCLTRLKNSRPARIVPHLLTLLLLTTCSAQLLSLLIFPPYHVRIASFEDLLRGDQKILGMRNEFYNFDGAFRARYAGVFYLIDDPNELYDLRNHFNTTWAYTMPYIKWLVIKTQQRHFSKPLFRWSKDLCFFDFMPTSVIVAPDSIYWESIKDFTFRIHQAGLMKHWIRKSFYDMIKAGKMSIKDYSDLETLKPLNIGDLEIVWRVCGAAIAVASAIFIMELLYFYINVFFNSL

>DmelIR21a

MSYYWVALVLFTAQAFSIEGDRSASYQEKCISRRLINHYQLNKEIFGVGMCDGNNENEFRQKRRIVPTFQGNPRPRGELLASKFHVNSYNFEQTNSLVGLVNKIAQEYLNKCPPVIYYDSFVEKSDGLILENLFKTIPITFYHGEINADYEAKNKRFTSHIDCNCKSYILFLSDPLMTRKILGPQTESRVVLVSRSTQWRLRDFLSSELSSNIVNLLVIGESLMADPMRERPYVLYTHKLYADGLGSNTPVVLTSWIKGALSRPHINLFPSKFQFGFAGHRFQISAANQPPFIFRIRTLDSSGMGQLRWDGVEFRLLTMISKRLNFSIDITETPTRSNTRGVVDTIQEQIIERTVDIGMSGIYITQERLMDSAMSVGHSPDCAAFITLASKALPKYRAIMGPFQWPVWVALICVYLGGIFPIVFTDRLTLSHLMGNWGEVENMFWYVFGMFTNAFSFTGKYSWSNTRKNSTRLLIGAYWLFTIIITSCYTGSIIAFVTLPAFPDTVDSVLDLLGLFFRVGTLNNGGWETWFQNSTHIPTSRLYKKMEFVGSVDEGIGNVTQSFFWNYAFLGSKAQLEYLVQSNFSDENISRRSALHLSEECFALFQIGFLFPRESVYKIKIDSMILLAQQSGLIAKINNEVSWVMQRSSSGRLLQASSSNSLREIIQEERQLTTADTEGMFLLMALGYFLGATALVSEIVGGITNKCRQIIKRSRKSAASSWSSASSGSMLRTNAEQLSHDKRKANRREAAEVAQKMSFGMRELNLTRATLREIYGSYGAPETDHGQLDIVHTEFPNSSAKLNNIEDEESREALESLQRLDEFMDQMDNDGNPSSHTFRIDN

>DmelIR25a

MGSRLDWGVADVALWAIADQIDYHQVFINEVDNEPAAKAVEVVLTYLKKNIRYGLSVQLDSIEANKSDAKVLLEAICNKYATSIEKKQTPHLILDTTKSGIASETVKSFTQALGLPTISASYGQQGDLRQWRDLDEAKQKYLLQVMPPADIIPEAIRSIVIHMNITNAAILYDDSFVMDHKYKSLLQNIQTRHVITAIAKDGKREREEQIEKLRNLDINNFFILGTLQSIRMVLESVKPAYFERNFAWHAITQNEGEISSQRDNATIMFMKPMAYTQYRDRLGLLRTTYNLNEEPQLSSAFYFDLALRSFLTIKEMLQSGAWPKDMEYLNCDDFQGGNTPQRNLDLRDYFTKITEPTSYGTFDLVTQSTQPFNGHSFMKFEMDINVLQIRGGSSVNSKSIGKWISGLNSELIVKDEEQMKNLTADTVYRIFTVVQAPFIMRDETAPKGYKGYCIDLINEIAAIVHFDYTIQEVEDGKFGNMDENGQWNGIVKKLMDKQADIGLGSMSVMAEREIVIDFTVPYYDLVGITIMMQRPSSPSSLFKFLTVLETNVWLCILAAYFFTSFLMWIFDRWSPYSYQNNREKYKDDEEKREFNLKECLWFCMTSLTPQGGGEAPKNLSGRLVAATWWLFGFIIIASYTANLAAFLTVSRLDTPVESLDDLAKQYKILYAPLNGSSAMTYFERMSNIEQMFYEIWKDLSLNDSLTAVERSKLAVWDYPVSDKYTKMWQAMQEAKLPATLDEAVARVRNSTAATGFAFLGDATDIRYLQLTNCDLQVVGEEFSRKPYAIAVQQGSHLKDQFNNAILTLLNKRQLEKLKEKWWKNDEALAKCDKPEDQSDGISIQNIGGVFIVIFVGIGMACITLVFEYWWYRYRKNPRIIDVAEANAERSNAADHPGKLVDGVILGHSGEKFEKSKAALRPRFNQYPATFKPRFMNLLISMFILILAAGEGEIIPSMEESVVTNFVKSLVKTKQAIVFSCLFKDFKEISLALMRINQFVSVVNLNQSYSLTSILTRENYARTSVMVNARCSGSSELLFEASENRYFNKTYQWFLWGVDLEVQSLFPLNLNYVGPNAQITYVNETADGYAYWDIHSKGRHLKSNLEINLIATLINDTLNIARDIFHLQSIDFRGQFNGLTLRGASVIDKEDIISNEQIESILSRPTKDAGVAAFIKYHYELLGLLRERFNFTVNFRNSRGWAGRLGNTTFRLGLLGIVMRNEADIAASGAFNRINRFAEFDTIHQSWKFETAFLYRYTSDLDTHGKSGNFLSPFSDRVWLFCLLTLGAFSIIWVLFEIIDYKILRIRVNSQKLEHLNQKSSVICIKTTCIERILQTFGACCQQGLDPNPVDRSVRFLVMTLFLFSLVMYNYYTSSVVGGLLSSSDQGPSTVDEITASPLKISFEDIGYYKVLFRESQNRSITRLIEKKLSSSRSLNELPIFSHIEDAVPYLKAGGFAFHCEVVDAYPVISEYFDANEICDLREVSGLMEVEILNWILHKNSQYTEIFKTAMCNAQEKGFVERILRRRQIKKPACQSLYTVYPVSLSGVLPGFVILICKSINKFS

>DmelIR31a

MNLLISMFILILAAGEGEIIPSMEESVVTNFVKSLVKTKQAIVFSCLFKDFKEISLALMRINQFVSVVNLNQSYSLTSILTRENYARTSVMVNARCSGSSELLFEASENRYFNKTYQWFLWGVDLEVQSLFPLNLNYVGPNAQITYVNETADGYAYWDIHSKGRHLKSNLEINLIATLINDTLNIARDIFHLQSIDFRGQFNGLTLRGASVIDKEDIISNEQIESILSRPTKDAGVAAFIKYHYELLGLLRERFNFTVNFRNSRGWAGRLGNTTFRLGLLGIVMRNEADIAASGAFNRINRFAEFDTIHQSWKFETAFLYRYTSDLDTHGKSGNFLSPFSDRVWLFCLLTLGAFSIIWVLFEIIDYKILRIRVNSQKLEHLNQKSSVICIKTTCIERILQTFGACCQQGLDPNPVDRSVRFLVMTLFLFSLVMYNYYTSSVVGGLLSSSDQGPSTVDEITASPLKISFEDIGYYKVLFRESQNRSITRLIEKKLSSSRSLNELPIFSHIEDAVPYLKAGGFAFHCEVVDAYPVISEYFDANEICDLREVSGLMEVEILNWILHKNSQYTEIFKTAMCNAQEKGFVERILRRRQIKKPACQSLYTVYPVSLSGVLPGFVILICKSINKFS

>DmelIR40a

MHKFLALGLLPYLLGLLNSTRLTFIGNDESDTAIALTQIVRGLQQSSLAILALPSLALSDGVCQKERNVYLDDFLQRLHRSNYKSVVFSQTELFFQHIEENLQGANECISLILDEPNQLLNSLHDRHLGHRLSLFIFYWGARWPPSSRVIRFREPLRVVVVTRPRKKAFRIYYNQARPCSDSQLQLVNWYDGDNLGLQRIPLLPTALSVYANFKGRTFRVPVFHSPPWFWVTYCNNSFEEDEEFNSLDSIEKRKVRVTGGRDHRLLMLLSKHMNFRFKYIEAPGRTQGSMRSEDGKDSNDSFTGGIGLLQSGQADFFLGDVGLSWERRKAIEFSFFTLADSGAFATHAPRRLNEALAIMRPFKQDIWPHLILTIIFSGPIFYGIIALPYIWRRRWANSDVEHLGELYIHMTYLKEITPRLLKLKPRTVLSAHQMPHQLFQKCIWFTLRLFLKQSCNELHNGYRAKFLTIVYWIAATYVLADVYSAQLTSQFARPAREPPINTLQRLQAAMIHDGYRLYVEKESSSLEMLENGTELFRQLYALMRQQVINDPQGFFIDSVEAGIKLIAEGGEDKAVLGGRETLFFNVQQYGSNNFQLSQKLYTRYSAVAVQIGCPFLGSLNNVLMQLFESGILDKMTAAEYAKQYQEVEATRIYKGSVQAKNSEAYSRTESYDSTVISPLNLRMLQGAFIALGVGSLAAGVILLLEIVFIKLDQARLWMLCSRLQWIRYDRKV

>DmelIR41a

MFIDLSWSLVLSAIVGKYLNESTICIFWNDKFEFQLLHKSDYISFVGINIKSFDDNGGHYIIDTGLKKKELQNKHLFLDELVIKIIISIEVTHCETFVVFDKDIDRFVNAFNKASVYSIWRSLHNKFVFAHIANESPESRNHFFEDQPNILFVVRDHSSASSFDIKTNKFVGRKAENPSQMILVDRYLASEQRFQFGKSLFADKLNNLQGREVIIAGFDYPPYTVIKHNMSTNAQDMGVSGESDFKNVYIDGTETRIVLNFCEQFNCTIQIDSSAANDWGKVYPNMSGDGALGMLINRKADICIGAMYSWYEDYTYLDLSMYLVRSGITCLVPAPLRLTSWYLPLEPFKETLWAAILLCLCAEATGLVLAYKSEQALYVLPGYREGWWTCTSFGVCTTFKLFISQSGNSKAYSLTVRVLLFACFLNDLIITSIYGGGLASILTIPSMDEAADTVTRLRFHRLQWAANSEAWVSAIRASDEALVKDILYNFHIYSDDELLRLAQDQHMRIGFTVERLPFGHFAIGNYLGPQAIDQLVIMKDDIYFQYTVAFVPRLWPLLDKLNTLIYSWHSSGFDKYWEYRVVADNLNLKIQQQVQETMTGTKDIGPVPLGMSNFAGFIIVWILGSAIATLTFLLELSLTYILKQSNLK

>DmelIR47a

MRQIKLLVWLLVVGVVSSTEQLQFLKNFLEAVHKERSISTILLIQRKVHKNDFLHGLYPIFWPIICLDETKRVELVNNFNKDFLALVYMESEADTLLLSALAADLNHIRDARIMIWLQMSPSENFLDRIVFQASKQKFLNLVVIENTLKTRRFYPFPQPKVQVIDKPFEEKEIYPALWRNFMGKNAIAVPDLVPPRSFNSFDPKTGHRRESGSIYNVFKAFTQRYNITMLLKWPLIRNTTQEEIIGKSVRGEIDLPITGQLISFRHPNGSRSQPLLGMTALSIAVPCGPELPMFDRFFLFYGLATPITITGYYVLLNTIEIILGTLSDRIKRHPRRKKILNLVLNLRVFSCILSLPTPQGNRLRSVKGQLTMVMSITGLILSCIVAAQTSTILTMKPQYRHIKNFQELSDSNITVVCNHLNYLTIKQQMDPKFMAKFMQNIWIVNSIEQMKMIFDLNTSYAYQTFSYKKDPFTLLQMHTTRKAFCRTPGLDLVSGLAYTAVLEKNSIYALALQDYTLKAFSAGLVYYWAEESIRDLISTVGRTQFEKLPIVIGYQSLKLQDYNVCWKILLIGGALAFCVFIVEVVVGLINRRI

>DmelIR48b

MILQQSSNLLKLLLLLAISSVRTQGLNDIIIELNQRLLISNNFLYCNQSDKLNEYEIKYLQHMPPISLMIFTSIESMNFTQVEYNLGADNKLFLIMGNEEPPYDFLHALNLHFQFAEYIIVIDEPVDLKKSTKWLDFVNHLWQQGYVQLLIYTSYDEKLYHKIIFPETVIEETLVEQYISIRGSFNNLYGYPVRVAAYNNAPRSMLYVNRWGKHIFAGFYMRFLRAFIDARNGSFVPVLTPSNSPGNCTLNLVNETVDVCADALAANPAAFSLTHGFRIASANVLVTHAKPLHSYRYLTAPFQWSVWACLVIYVLLVVNFLSFIGWLRSGKWEFSKYLLEVFSSLLFSGFYLKEIRGRERYILFGVLFIAGFVYSTEYLGLLKSMLISEVFEKQIDTFEALVESNITLMVDPYDKILFAKYNMPEILSPIMELVSFETLLKHRNRFDQDYAYILFSDRMALYDYAQQFLKHPKLLRIPIDFSFLYTGIPMRKRWFLKHHLGRAWYWAFESGLTRKLALDADFEAVRVGYLSFLITEHVEAQPLNVDYFVMPAIALAIGYILALLSFVIEMTAWRIREFLGCRKATMTSTGCSEGGHVDVD

>DmelIR48c

MSLLRIILIIIFLRIVSSIPDTIISHLSAELQIKIQIYFGLGNDLYDFSRLDGNYQKIIISHNISEEFKTYHDEPVLIIIRLERDLNLNLATLDVLRSYLTDRQYNDILLIDNDEENLNSYVDIRKAYWNAGFSQVLIYNSQQRTWSIKPYPYLQIRPTSLKEYIENRNTRNLMGYPLRVLVTNDPPHCFVDKDELPGSPNRYKGSIVTMLKIFADQLNATFQANPFREFRRYSTADCVQMVSDDEIDACGSIFIRTYTYATSQPVRLNRVVIMAPFGNPIEKFYYFFRPFDLYVWIGTGIIVVYIAVMGSLLHRWHFKEWNVGQYLLLAVQTLLNRELSLPQSSSGSKFMLLLLLFAIGFILSNLYVALLSMMLTTKLYQRPIENLADLKAANVNILLQTHNIRPNSVYGSSEELRERFLLVEESQHLEKRNGLDPSYAYVDSEDRMDFYLYQQKFLRRRRMKKLSNPVGYTWAVQVIKQNWVLEKHYNDHVQRFFETGLQNKLVDDVHELAVKAGFLHFFPTQTQTIEPLRLEDIVMAAMVLGGGHALAVICFLVELFA

>DmelIR51b

MCKVLTLLVVILLLALTNAAYNVTLLKSVLSLISTREPWINTPIFVGHNTQGGDLNDLIIWLHQTMGVTSLTMNLFLQPEHIRPLGHFKITRYNGIALFFCHDKHDIMWLTLDRNLRKLRRIRLIIILRNQRSGSQGAIKSIFNALWQYQFLNVLVLQRDQLYSYTPYPAMRFFKLDIHTEPLFPHAARNFHGYVVSTPAENDIPRVFHVHDPLTKSRKVLGYAYRTFVEYLDHYNASLRLTNPDENLDPTTSVNMNHIVQLIIDGQLEISLHPYVFTPPTATKSYPLLIYPNCLIVPMRNEIPRHMYLLRPFQLYSWYILLFAVFYITGILYCISPKLNKSSWPQRLGLNFLDAISKILFISPPITIYRPTWRHLIIFLQLSVLGFMSTSWYNIELDSFFTTIVVGEQVNSMDQLVHQQQRVLVKEYEINTFLRHVEPRLVEKVSRLLVPVNASEQVSALLSFNRSFAYPFTEERWQFFAMQQQYAFKPIFRFSSACLGSPHIGYPMRVDSHLETSLNHFILKIQDTGLLNHWVVSDFNDAMRAGYVRFVDNVLGYQSIDVDTLRLGWCVLGIGWILSALVFSCEYWHLYPWRFIA

>DmelIR52a

MALGWSVIILGFIGQLSAQILNYTQSRDLELLEGSLFRVLSRLNLEEEYNTLLIYGKECVFHSLLRKLEISAVTVPSGSTDYDWSFSTAILILSCGYDAENEENSYTLMKLQRTRRLIYLEDNSEPESVCMRYSLKEQHNIAMVKSDFDQSDTFYSCRLFQTPNYVEGHFFKDQPIYIENFQNMRGATIRTVADSLVPRTILYRDEKSGETKMMGYLGHMINTYAQKLNAKLHFIDTSKLGAKKPSVLDIMNWVNEDIVDIGTALASSLQFKNMDSVWYPYLLTGYCLMVPVPAKMPYNLVYSMIVDPLVLSIIFVMLCLFSVLIIYTQHLSWKNLTLANILLNDKSLRGLLGQSFPFPPNPSKHLKLIIFVLCFASVMITTMYEAYLQSYFTQPPSEPYIRSFRDIGNSSLKMAISRLEVNVLTSLNNSHFREISEDHLLIFDDLSEYLVLRDSFNTSFIFPVSVDRWNGYEEQQKLFAEPAFYLATNLCFNQFMLFSPPLRRYLPHRHLFEDHMMRQHEFGLVTFWKSQSFIEMVRLGLASMEDLSRKRNEEVSLLLDDISWILKLYLGAMFISSFCFILEILRCGERCKRLWRCRW

>DmelIR52b

MTWLVILLCFLGYMAAHIADISVQNQSLMDNELINLLLKLRNEEFYDTLLVYGKDCEFHSVIKNVDVAVVLVSDSMNFEWNFSSLTLILSCGPDIDNGGPNSTSIKLQRNRRLVLLKEDFQPSNICNIYTQKEQYNIALVRENFTKSKSIYTCRYFQDPNVDEVNLSGTKPIFIEQFQNMKGKAIRIVPDLLPPRVMLYQDANDGELKMIGYVANLITNFAQKVNATLQLDFLKPSTSITEISRMAKDDELDMGITLEASLNTSNLETSSYPYLLTSYCLMVQVPAKFPYNLVYALIVDPLVLGIIFVLFLLLSVLLIYSQKMSWQDLSVANILLNDKSLRGLLGQSFPFPLNASKKLRLIFTILCFASIMLTTMYEAYLQSFFTNPPSEPEICSFQDVGSYNRRIAMSALEVNGLIKTNNSHFREIRMDDLEIFDNMPECYELRDAFNLSYNYVVTGDRWRSYAEQQTLFKEPVFYFARDLCFSRLIFLSVPLRRHLPYRHLFDEHMMQQHEFGFVNYWMSHSFFDMVRLGLTSLKDLSRPLAYTPSLLMDDISWIMKIYLAAIVLCVFCFLLEIGVDKWKRWMKFRNLQILNTC

>DmelIR52c

MVWLIIILFCLGNSSSQILDVTNNSHLDFDYRLFGLLQRLQVEKSYDTLLVYGEDCAIPSLFERLQVPAVLVSSGSTNFDWNFSSLTLILSCNFQDEREENYRTLMKLQTSRRLILLKGHIKPESVCDFYSKKEQHNVAMVKENFYQLEVVYSCRLFQDQNYEKLNLFDGKSIYKDQFRNMHGAPIRTLSDKEPPRTIPYIDSKTGEEKFKGYVGMLISQFVKKVNATMQIREDLIKDDEEVSFVDITNFTSNDILDIGICEARTLEMSNYDAISYPYLMSSYCFMAPLPDSLPFSDVYMAIVAPSILIMFLIIFCICSVLIIYIQERSYRSLTIRSVLMNDICLRGFLAQPFPFPRQYNRKLKLIFMLVCFSSLISTTMYTAYLQAFLWGPPIEPRLTSFDDVKKSRYTMAINIYEREFLEALNVSLEDVEIYDYGKFSKLRSTFNTNYLFPVTALQWFTINEEQKLFKYKIFYYCDAFCLNQFDILSIPLRRHLPYRDIFEEHMLLQKEFGLTKYWIDQSYRDMIRANLTTFKDFSPLLENDYIEVHNLYWVFTMYFVGMGMGLCFFILEILRPLRYWRNCKIKCEYCYAFLKNFAK

>DmelIR52d

MVRIIIILLCLGYTKARILDATNTNHTDLEERLLSLLLRLQQEQFFNTLLIYGEDCAFSSLSRRLQVPTILVSSGSTSFEWNYSSLALILTCEFKAEREENYQTLKKLQMNRRLILLNGNIKPDSVCDFYSKKDQYNIAMVNNNFHQVGIIYACRLFQERNYEKVYLSEGNPIYVDQFRNMQGALLKSITFNLIPGSMAYRDPKTGQEKHIGYVANLLNNFVEKVNATLDMQVKLHKAGKKTSFYNITKWASEDLVDIGMSYAAYFEMTNFDTISYPYLMTSTCFMVPLPDMMPNSEIYMGIVDPPVLVVLIAIFCIFSVMLNYIKQRSWRSLSLVNVLLNDICLRGFLAQPFPFPRQSNRKLKLISMLVCFFSVITTTMYTSYLQSFMWGPPIDPKMCSFADLENSRYKLAIRRYDIEMLRPFNVSMDHVVVFDESSQLEYLRDSFDDNYMYPMSALSWSAFKEQQKLFAFPLFYYSEKLCLKPISFFSFPIRRHLPYRDLFEEHMLQQNEFGLSTYWIDRSFSDMVRLKLATMNDFSPPRLEDYIEVSDLSWVFGMYFTGLGISCCCFGLELLGLPSWTRRLRLTNWLRVRN

>DmelIR54a

MWTVITGIVLWAPVLVAGSAVDFIFRAAAEHSLSVIMIRIDYCPYNWAKDIFENQTIPVVVLSDSETFINIRMFSRPLHVACLPGHELQKDLALLENFTSSLMDFPSQKKIVYISNNFSDPTRMDYIFETCYHRRIWNIVGLLASDEHRYFYRYHLYPSFRTEYRSLESSTIFDKDFPNMHGHPLTVMPDQWLPRSVLYVDRRTGKQILAGSVGRFFHVLSWKLNATLQLSKKVTTGRFLNATALKELSESFSVDVPASLTIMERVEQLASTSYPMEVTHVCLMVPVARRIPIKDIYFILSSASNMFLAIVIVSSYGLALNLLRNMTHRDVRLVDFVLNDKALRGILGQSFNLPLSRSFSTRLIFLMLGIVGLNVSSIFGAGLDTLMAHPPRQFQARSFAGLRRTKIPLVTTEEDFPTWMKLRVPMLVVNVSEYNHLRNGRNTSNAYFASRLYWNLFSEQQKRFTRELFIYSTDDCLWSLALLSFQWPQNSLFTEPVSQLILEVNANGLYDFWVGMHYYDMTAAGLSGLEDPSLQLKEREHPTSLRIVDFQWMWQAYGTFMVIAILVFLLEVSWHRITSLFVSLVY

>DmelIR56a

MGSRFFIRNLILFGLLASSNMQIPFGELEKKFELDVDFLLGVTELVGHIQGLYSITVYADCIDIHPSIQQRIMDKFMVPVNTIGSNLSRPNYHKLDNSRIRIVLFTGLNDTILVNLNKTDVPYSDNFYMLAYASAIKNKCIELDFIEEVFTLLWKMSIQNAILLIRGEFMMEMWSYLYMGKIHKIKLTKPNSYLESLRKYNYRFSLEVINDPPAIFWYNSSEQADVTGGGNLSVSGPLGLIIINFLRHLNVTIDIVPIPGKQTSQYELFQQPDNLRAENGVNMVGSALLKYSPMVTQSRMCLLVSNRRMIPFSRFLDRLVSPGVHKLTFVSSIGIFVIKYFSHRPRSFVDAIFCTIRFFFAIPLPSIILNRLPVVDRFIEVFIIIFVQILLSSNISITTSALTTGFWEPPIINVETMRASGLHILTEDPTILQAFKENILPSSLADLVILVDEDTYFHHVTTLNNSYVYVVQAHNWQIFRLYQQQMTNEPFEIASEELCSKWRILGIPLNPKSPLRFMFKDYFYRILESGLREQWVHSGFKKFCEFNNLKKLPVDSVDSWQPLSIEFYSNVIRAYIIGLVIATLAFVAELLHNGYRRKNVKKT

>DmelIR56b

MLLDTDLASGVIRSPYSFDIPHAFIFNETQFVVPKFCGPYMEIVKHFAEVYHYQLFLDSLESLPKKSVVEQDIISGKYNLSLHGVIIRPEETSDFFNATQHSYPLELMTNCVMVPLAPELPKWMYMVWPLGKYIWTCLFLGTFYVALLLRYVHWREPGNATRSYTRNVLHAMALLMFSANMNMSVKLKHASIRVIIFYTLLYIFGFILTNYHLSHMTAFDMKPVFLRPIDTWSDLIHSRLRIVIHDSLLEELRWLPVYQALLASPSRSYAYVVTQDAWLFFNRQQKVLIQPYFHLSKVCFGGLFNALPMASNASFADSLNKFILNVWQAGLWNYWEELAFRYAEQAGYAKVFLDTYPVEPLNLEFFTTAWIVLSAGIPISSLAFCLELFIHRRKQRRPQYERFECYDY

>DmelIR56c

MQHLLNLLAPFGRMNVFQEIVWFVSPHQRLDQLDEFIMRIDEAFGKSATQTVVNNNTEMRMIYSSARRNHMSFVFTTGAEDPIMKVFSKVLLGRHFYVSMVIYVDKVGDMHPIYDLLTFAYNQQFFNSMVHFESMEGVNQLFGVSKFPVMSFENRTDFLKYMGKIWKQVQNARSDVGGFGFTTPLRQDLPHLFQSQGHYDGSTYRIIETFVRFINGSFKELIMPPDSLGGQVINMKDALQLIRERKMEFCAHAYALFMSDEELEKSYPLLVVQWCLMVPLYNSVSTYFYPLQPFDWNVWFFALGALLALVLLELMWLRMFGGWSGYRGAVLNSFCYIINVPIEGQLQQPCLLRFLLLATVFFHGFFLSAYYTSNLGSILTVNLFHAQINTMNDIVSAQLPVMIIDYEMEFLLNLNKELPQEFLELLRPVDSAVFSEHQTSFNSSFAYFVTEDHWEFLDEQQKHLKQRLFKLSSICFGSYHLAFPLQMDSSLWRDIEYFTFRIHSSGLLNFYARSSFGSALHAGLVQRMPDTQEYTSAGLQHLAIAFILLLVMSFLAGIVFVLETLSR

>DmelIR56d

MDNRAAELILRERNIFPTNGSDNITLLNNMFVLEMFYRITQLYHFKNFIFYISERLDLNNKDSQEFFHNFWTYFPMAPNLIITREHHLGIPMMQFISTPSLVMVFTTGKDDPIMELASHNQQGIHWLKTIFVLFPSLQSRDFETNPESLAQFTAEIKDVYDWVWRKQFINTFLITIKDNVFILDPYPTPSIVNKTGVWQAEEFFHKYAKNMKGYLVRTPILYDMPRVFKSDRPTNRYEKNFIHGTSGNLFLGFLEFVNATLMDTSANVTADYLNMTNLLDLVSQGVYETLIHSFTEITTKFVVSYSYPIGINDCCIMVPYRNQSPADQYMHEALQENVWVLISLFTLYITVAIYLCSPLRPRDLSAAFLQSICTLTYSVPTFIIRTPTLRMRYLYILLAIWGIVTSNLYISRMTSYFTTAPPVRQINTVQDVVEANLRIKMLAIEYERMAKSPLQYPESYLNQVDLVDKHMLDLHRDPFNTSFGYTVSSDRWRFLNLQQLHLRKPIFRLTEICEGPFYHVFPLHKDSHMRSVMTEYIMIAQQAGLMNHWERETFWEAVHLHRIHVHLFDDEPMALSLDFFSSLLRTWTLGLILAGLAFAAEMKWHEHVTFKRRPVIRITRKPRSFLRRFMKL

>DmelIR60a

MWCNNPGLIIIIFLGQILNLCQGIVNLSNETANTVIFMLPEKDLGPDVWKAGVGCLDSFAQIFFFRNPKERFTRAYNLMLVHAFHLSSPADQIQEGFSKLINEAVTNPGPPDREELFQMRVASDYNITNGTEDKGELILADNYVIVVDSVDRLKELMKKKIVEMRSWNPGARFLVLFHNATCRNRPLGVASNIFKDLMEMFYVHRVALLYANSTMNYNLLVNDYYSNVNCRILNVQSVGQCHDGKLYPNNAVVKASMQDYVSGFSPRNCTFFACSSISAPFVEADCILGLEMRILGFMKNRLKFDVNQTCSLESRGEMDGPANWTGLLGKVQNNECDFVFGGYYPDNEVADHFWGSDTYLQDAHTWYIKMADRRPAWQALVGIFEAYTWIGFILILIISWLFWFTLVMILPEPKYYQQLSLTAINALAVTISIAVQERPICETTRLFFMALTLYGLNVVATYTSKMIATFQDPGYLHQLDELTEVVAAGIPFGGHEESRDWFENDDDMWIFNGYNISPEFIPQSKNLEAVKWGQRCILSNRMYTMQSPLADVIYAFPNNVFSSPVQMIMKAGFPFLFEMNSIIRLMRDVGIFQKIDADFRYNNTYLNRINKMRPQFPETAIVLTTEHLKGPFFILVVGSCWAALTFIGELIIHRWRTQLVSTSEQQDRRSDKRRRRRRRRKPEKDNRWQRQVQVAPVVRFTPVKRRKVFQGQTSQK

>DmelIR60b

MRRSLYLIIAIGLVDVHCVSLRYILNALENELQYRAILLVESASEIESCWEQKYIQGAVPILNFNANQSLYLKDALNTNILALVCLNENVESTMQALYENLEDMRDTPTILFVLSDSKVQDVFLECLRRKMLNVLAFKGLDRGFVYSFRAFPTFRVIERNVMDILQYFEQQLEDLGGHTLTTLPDNIIPRTVVYKSPDGSRQLAGYLYPFLRNYVSTINATLKVCWHLVPEDGMIQLGEVVRLSEIHDVDFPLGMHGIEHGSTSQNVPLEVSSWFLMLPMEPSLSRAQFFIMLGFEKVTPVLLLLTILLSTAHRIEMGLRPSWRCYVLGDRVLQGTLGQAFFLPRRLSVKLMLVYSLILLNGFTFSNYSITSLETWLVHPPSGHPIHSWEQMRTLNLKVLIVPSELDSMTKALGKQFTESNSDLFELSKSGNFQDKRLAMDQSYAYPVTCTLWPLLEHAQIRLPKPEFRRSREMVLIPLLIMAMPLPKNSMFHKSLNRYRALTHQSGLYEFWFKRSFNELVALRKIHYKVNGDHQIYRDFEWQDFSYVWLGFVGGTIASILVLLAEIGYHRWQLNQN

>DmelIR60d

MRLAIYVAFLSSIGNRSGFLSSLLMSLGKELHYKTILLVGGSSTCWSLEPFETGVPILNLRGENNAYPQDTFNSQMLALACLQTESEDAVKLLYRSLKDMRDTPTLLFASSEEHIHDTLFLGCFRENMLNVLALTASSKEFIYSYQAFPTFRVIKRKLVEIHRYFEPQLKDLGGHIVSALPGNIMPRTMCYRNAEGERQLAGYLNTFIRNYVESINGTLRISWGLVPEDDMRHLTISRLSKIQHVDFPLGIIPLYNKTDKQHVYMEISSWFLMLPMETSVPRAHLFVKLGLERLLPIIVVVGAVLGNAHRIEVGLGPSWRCYYLADKVLRGALAQPIVLPRRLSPKLMLIYSLLLLSGFFLSNYYMASLTTWLVHPPASDRILEWDQLRYLHLKVLTIPEEFKYMSLILGTDFMTAYGSIFQLTNSTDFQRRRISMDPSYAYPVTTSLWPFLELSQVRLRRPLFRRSYDMVLQPFQVMSLPLPRNSIFHKSLLRYAALTRETGLYYYWFRRSYYELVALGKISYKEEEGNPYCDLKWNDFRIVWLAFLGGTIISCLALLLEVAHYRWHLGNSSL

>DmelIR60e

MVIKMISFLLVSVLLCLVGASDSESMQVQVLQDLNLALQTELNVFIDFECCATSEILHKLDSPRILLSSNSREARDLRIRGNFTESTLIIVSVMDSDLNPLVASLLPRLLDELHELHIVFLSNEEPGFPKQDLYTYCFKEGFVNVILMSGKGLYSYLPYPSIQPISLSNVSEYFDRARIIRNFQGFPVRILRSTLAPRDFEYSNEQGGLVRAGYLFTAVKELTYRYNATIESVPIPDLPEYDVYLAVAEMLHTKKIDIVCYFKDFSLEVAYTAPLSIIREYFMAPHARPISSYLYYSKPFGWTLWAVVISTVLYGTVMLHLAARGARVEIGKCLLYSLSHILYNCHQKIRVAGWRDVAIHGILTIGGFILTNVYLATLSSILTSGLYDEEYNTLEDLARAPYPSLHDEYYRSQMKAKTFLPERLRRNSLSLNATLLKAYRDGLNQSYIYILYEDRLELILMQQYLLKTPRFNMIRQAVGFTLESYCVSNSLPYLAMTSEFMRRLQEHGISIKMKADTFRELIHQGIYTLMRDDEPPAKAFDLDYYFFAFVLWTVGLISSLLVFFAELVSGHL

>DmelIR62a

MYLQFLFALFLSRYQIVATENFDRAFELALFLDRIGRVHRLHAITIVNSLGSVDPSYLDDLHRGLMCNSSNHFYMLPQMTATDKDSSHVHFSSLQDEETIYLVFARDSKDAVIYLQAERARGRRYTRTMFLLRKQESQKDIKYFFELLWKLQFRSALVVVAARNFYQMDPYPTVRVIRMRRLSSYDPHHVFPPANRKNFRGYRMRLPVQQDVPNTFWYKNRRTKAWELAGLGGILINQLMMHLNVTMDLFRFEVNGSSLLNMAALTDLIVKGKVELSPHLYDTLQSNTSVDYSYPTQVAPRCFMIPLDNEISRSLYVFLPFSLTMWLCLLFVLLVVHFVYVRRLIPDGHFWAILGVPGAGQVRYGNRKPVRRFSTFLILFGIFILGQTYSTKLTSSLTVTLIRRPDNSLEELFLLPYRILVLPTDVYAIVDSLGHAEQFSTKFSCTDAENFSQKRISMHPEYIYPISTIRWRFFDMQQRFLRKKRFYFSKICHGSFPYQYQLRVDSHLKDALHRFLLHVQQAGLHDLWLDTCYRKAHRMGYLKDFSTLAELEEKLRLRPLALNLLVPAFSLFLCGMLGSGIAFLVEIRHSFGCRQKPPSINRNPGD

>DmelIR64a

MHWWLLVFLPLSCQGLPEHELLELELDYGLAEPQRTSLLQSSLILQFSQDYKHIPRITYFTCQKPHLQTPNQIPNAAEHRDAFAAKNFQLIKSLYESELFVRIVLLDVLAQSPTSGRPNRPGNGPTGGFSQTPSQAQSNSEWLEGVLRMEALRQIAVVDLACGAVSRRFLELASAKMLYSEKFHWLLIEDFAWHGRTQTAEGSGKRDDGEMEEEEPPGQQIQATDDEDLPSIESFLGGMNLYMNTELTLAKRMSEAAHYTLFDVWNPGLNYGGHVNLTEIGSFTPTEGIQLHTWFRTTSTVRRRMDMQHARVRCMVVVTNKNMTGTLMYYLTHTMSGHIDTMNRFNFNLLMAVRDMFNWTFVLSRTTSWGYVKNGRFDGMIGALIRNETDIGGAPIFYWLERHKWIDVAGRSWSSRPCFIFRHPRSTQKDRIVFLQPFTNDVWILIVGCGVLTVFILWFLTTIEWKLVPHDGSALIKPKGGAPPRHHYQQQQQQEQVEAPVRPITAVSVVVSKEKVEEKQEEYEDSTPIDAGTLWQRCYQKLNKYIKDRKAKQKKAPERVGLFLESVLFFVGIICQQGLGFSTSFVSGRCIVITSLLFSFCIYQFYSASIVGTLLMEKPKTIKTLSDLVHSSLKVGMEDILYNRDYFLHTKDPVSMELYAKKITSVPTTKENEADEDEPVDPNPVSTDPAKSYRDIVHSHETGAHAKDNAASNWLDPETGLLRHLGFAFHVDVAAAYKIIAETFSEQDICDLTEVSMFPPQKTVSIMQKNSPMRKVISYGLRRVTETGILTYHFNVWHSRKPPCVKKIETSDLHVDMDTVSSALLILLFSYAITLMILGTEILYSKWHNRIQLKWVGAT

>DmelIR67b

MELLYLNTLQSLSLLEGNRLVQTVQELNNIYQTELNVFLEFGNGADILESAQGTFVPTLWIKNPQNQKVMKGNFTSCTLTILYLEDEHLDRGLYYLANWLWEYHHLEVLIFFNGGSYDKLIQIFSRCFNEGFVNVLVMLPGSDELYTFMPYQDLKILNLKSIKEFYSLSRKKMDLNGYNITSGLVIAGAPRWFSFRDRQNRLILTGYMLRMIVDFTNHFNGSVRLMNVLTVNDGLELLANRTIDFFPFLIRPLKSFSMSNILYLENCGLIVPTSRPLPNWVYLLRPYAFDTWIAWLIMLIYCSLALRILSKGQISISAAFLKVLRLVMYLSGSRDMGTRPTTRRLFLFVILTTSGFILTNLYVAQLSSNSAAGLYEKQINTWEDLDKSDSIWPLIDVDIKTMEKLIPDRTKLLKKIVPTLEADVDTYRRNLNTSCIHSGFFDRIDFALYQQKFLRFPIFRKFPHLLYQQPLQISAAFGRPYLQLFNWFVRKIFESGIYLKMKDDAYRHGIQSGLLNLAFRDRHLEVKSNDVEYYYLIAGLWFGGLTLATVCFLLELLIGYAKIKVTISCKMNIM

>DmelIR67c

MFCWLIFLNIILLSDRSESWSAREVIHQFNHDQQLQLNIYLDCNDVELQIGQEVSNLFVNSTADKMKILGRFSSHSLIIACFKDSTRNRTLNGVKELLWGLQYLPILFVVDSNMDFYFQQALRHGFIHVLALNFMNGSLYTYKPYPKVEVHQIKDMQKFYKLTKLRNLQGQAVRTTVETMTPRCFRYRNRHGQLVYAGYMYRMVKEFISTYNGTEEHVFGNVDTVPYKEGLAALKNGEIDMMPRIIHALEWYYFYRSHILYNIKTYIMVPWAEPLPKSLYFIQPFRGTVWITIMVSFVYASIVIWWIRYRQQGNSSLTQSFMDVLQLLFQLPLSKIWHFNMGTHQVVSFIVLFVFGFMLTNLYTAQLSSYLTTGLFKSQINTFDDLFREKRTLLVESFDAEVLHNMTKEKIIQKEFESIILITSIEEVFKHRKSLNTSYAYEAYEDRIAFELSQQRYLRVPIFKILKEVYDQRPVFVALRHGLPYVELFNNYLRRIFESGIWIKLQEDSFLEGIASGEISFRKSKSREIKIFDKDFYFFAYILLGMGWCVSTIALFLELWSFKYSVTNVLHEG

>DmelIR68a

MRCLWILIVAFISLAMATSIPIPIANPAPLSGYEMQLKILLQKILWVANVKRCFAVITDDLHYPIYDRIFFESVGRRVIPFFVMRTNESDDLQRPSRQVELFVKAIKSSDCELNVITILNGWQVQRFLGYIYDNRSLNMQKKFVLLHDLRLFESDMIHLWSVFIDAIFLKRQLDNKYTISTIAFPGILSGVLVMKNIANWELGKGLNGRILFADKTSNLFGTSLPVAISEHVPMVLWANATKSFQGVEVEIMNALGKALNFKPVYYKPNQTENMDWTELDGGASVAYGSGNPDGYAQNGTHIDSMLVDEVAAHSARFAIGDLHLFQVYLKLVELSAPHNFECLTFLTPESSTDNSWQTFILPFSAGMWVGVLLSLFVVGTVFYAISFLNAIINGNVSSEFFRCLRPNRNVPMDPKIYRRISFRIAISRYRSSKGDRMPRDLFDGYTNCILLTYSMLLYVALPRMPRNWPLRVLTGWYWIYCILLVATYRASFTAILANPAARVTIDTLEDLLRSHIPPSTGATENRQFFLEANDEVARKVGEKMEVFGYSDDLTSRIAKGQCAYYDNEFYLRYLRVADESGSALHIMKECVLYMPVVLAMEKNSALKPRVDASIQHLAEGGLIAKWLKDAIEHLPAEALAQQEALMNIQKFWSSFVALLIGYVISMLTLLAERWHFKHIVMKHPMYDVYNPSLYYNFKRIYPQH

>DmelIR68b

RNMEAHELEPFRRTDSLSIFQIPAAEKGDSLVRRILDMLNPHQRRKHMHKYLFVWPNAGRHQLLRLFRGSWAKKLLYGLAITGRENGTFDFDPFAWGGLQVIQRLDGEVPYARKVKDLRGYPLRFSMFTDPLMAMPRSPVETAGYQAVDGVAARVVGEMLNASVTYVFPEDNESYGRCLPNGNYTGVVSDIVGGHTHFAPNSRFVLDCIWPAVEVLYPYTRRNLHLVVPASAIQPEYLIFVRVFRRTVWYLLLVTLLVVVLVFWVMQRLQRRIPRRGVIQFQATWYEILEMFGKTHVGEPAGRLSSFSSMRTFLMGWILFSYVLSTIYFAKLESGFVRPSYEEQVDRVDDLVHLDVHIYAVTTMYDAVRSALTEHQYGLLENRSRQLPLGIATSYYQPVVRRRDRRAAFIMRDFHARDFLAITYDSQAERPAYHIAREYLRSMICTYILPRGSPFLHRLESLYSGFLEHGFFEHWRQMDLITRVGASPDAEEFLEDLGDQTDTDSGSNELAIRNKKVVLTLDILQGAFYLWSVGIGISCLGFAVEHAHWFWRRQTLRNAVEARTS

>DmelIR75a

MQLVQLANFVLDNLVQSRIGFIVLFHCWQSDESLKFAQQFMKPIHPILVYHQFVQMRGVLNWSHLELSYMGHTQPTLAIYVDIKCDQTQDLLEEASREQIYNQHYHWLLVGNQSKLEFYDLFGLFNISIDADVSYVKEQIQDNNDSVAYAVHDVYNNGKIIGGQLNVTGSHEMSCDPFVCRRTRHLSSLQKRSKYGNREQLTDVVLRVATVVTQRPLTLSDDELIRFLSQENDTHIDSLARFGFHLTLILRDLLHCKMKFIFSDSWSKSDVVGGSVGAVVDQTADLTATPSLATEGRLKYLSAIIETGFFRSVCIFRTPHNAGLRGDVFLQPFSPLVWYLFGGVLSLIGVLLWITFYMECKRMQKRWRLDYLPSLLSTFLISFGAACIQSSSLIPRSAGGRLIYFALFLISFIMYNYYTSVVVSSLLSSPVKSKIKTMRQLAESSLTVGLEPLPFTKSYLNYSRLPEIHLFIKRKIESQTQNPELWLPAEQGVLRVRDNPGYVYVFETSSGYAYVERYFTAQEICDLNEVLFRPEQLFYTHLHRNSTYKELFRLRFLRILETGVYRKQRSYWVHMKLHCVAQNFVITVGMEYVAPLLLMLICADILVVVILLVELAWKRFFTRHLTFHP

>DmelIR75b

MLQLHNLILHNLIHMAKLSHVLILHCSLSHLALLAQSKNIFTQFQPLHSDIQLNDDFLNHNILKLGVFLDINCDKSGTVLDMASAKRFFSHRYHWLIYDRSMNFSVLESHFKEAQIFVDADVTYVTHDPFSKNFLLYDVYNKGRQLGGELNITADREIFCNKTNCRVERYLSELYTRSALQHRKSFTGLTMRATAVVTALPLNVSIKEIFDFMNSKYRIQLDTYARLGYQARQPLRDMLDCKFKYIFRDRWSDGNATGGMIGDLILDKADLAIAPFIYSFDRALFLQPITKFSVFREICMFRNPRSVSAGLSATEFLQPFSGGVWLTFALLLLLAGCLLWVTFILERRKQWKPSLLTSCLLSFGAGCIQGAWLTPRSMGGRMAFFALMVTSYLMYNYYTSIVVSKLLGQPIKSNIRTLQQLADSNLDVGIEPTVYTRIYVETSEEPDVRDLYRKKVLGSKRSPDKIWIPTEAGVLSVRDQEGFVYITGVATGYEFVRKHFLAHQICELNEIPLRDASHTHTVLAKRSPYAELIKLSELRMLETGVHFKHERSWMETKLHCYQHNHTVAVGLEYAAPLFIILLGAIILCMGILGLEVIWHRHCTLH

>DmelIR75c

MTSWPLYRLIVFNLLEINLSNLMVFHCWSIKEAFPLVEMLNQNGIFSQYIDVQNPDNLANVHKEYLDSDLVRLGVFLDLGCDKAELVTNQSSRARLYNQNLHWLLYDEAGNFTKLTQLFEGANLSLNADVTYVSREDEERFILHDVYNKGSHLGGKLNITVDQTLQCNRSHCQVKEYLSELHLRPRLQHRMDLSSVTFRLAALVSVLPINSSEEELLEFLNSDRDSHMDSISRIGNRLIMHTQEILGFKLHYIWCGTWSVQDAFGGAIGMLTNESAELCTTPFVPSWNRLHYLHPMTEQAQFRAVCMFRTPHNAGIKAAVFLEPFMPSVWFAFAGLLIFAGVLLWMIFHLERHWMQRCLDFIPSLLSSCLISFGAACIQGSYLMPKSAGGRLAFIAVMLTSFLMYNYYTSIVVSTLLGSPVRSNIRTIQQLADSSLDVGFDTVPFTKTYLVSSPRPDIRSLYKQKVESKRDPNSVWLSPEEGVIRVRDQPGFVYTSEASFMYHFVEKHYLPREISDLNEIILRPESAVYGMVHLNSTYRQLLTQLQVRMLETGITSKQSRFFSKTKLHTFSNSFVIQVGMEYAAPLFISLLVAYFLALLILILEICWARYAKKKFSTIIPQNQ

>DmelIR75d

MKVQVAHWLPLIFFLLVSGTPRVAGSWRSEYSRQDPDPKTRWGNQLPDMLVAYYRHHGVHSLMLVVCHTDIADFRLWKLWQHFNLNNFYVQVSTESSLRDLQHVDALDEHKDAPPPKSFHANNSTHWETSFLLPALPYKMGILLLEFSSECALNLLRWSAASEHNYFTTNRFWLLLTEDPGDIDLLEDPEIFIPPDSELRVLHYENVGNFSCSLIDLYKVAAWKPLKRTLVGHNIRNSRHVIHALQHFGSAITYRQDLEGIVFNSAIVIAFPDLFTNIEDLSLRHIDTISKVNHRLMLELANRLNMSYNTYQTVNYGWRQPNGSFDGLMGRFQRYELDLAQLAIFMRLDRIALVDFVAETYRVRAGIMFRQPPLSAVANIFAMPFENDVWVSILMLLIITTVVLVLELFFSPHNHDMSYMDTLNFVWGAMCQQGFYVEVRNRSARIIVFTTFVAALFLFTSFSANIVALLQSPSDAIQSLSDLGQSPLEIGVQDTQYNKIYFTESTDPVTKNLYHKKIASKGENIYMRPLLGMEKMRTGLFAYQVELQAGYQIVSDTFSEPEKCGLMELEPFQLPMLAIPTRKNFPYKELIRRQLRWQREVSLVNREERKWIPQKPKCEGGVGGFVSIGITECRYALGIFGCGAAVSFVLFLFEFIFRHFKQVYRIIKGYREVQR

>DmelIR76a

MFVYTKEFEDKKDSYLSGYIFQDQPNILVITSQYLNSSTFEIKTNRFVGPRNFNKNPEPVEFYILQRFDAKGTKATWETQSAMSSKMRNLKGREVVIGIFDYKPFMLLDYEKPPLYYDRFMNTTDVTIDGTDIQLMLIFCELYNCTIQVDTSEPYDWGDIYLNASGYGLVGMILDRRNDYGVGGMYLWYEAYEYMDMTHFLGRSGVTCLVPAPNRLISWTLLLRPFQFVLWMCVMLCLLLESLALGITRRWEHSSVAAGNSWISSLRFGCISTLKLFVNQSTNYVTSSYALRTVLVASYMIDIILTTVYSGGLAAILTLPTLEEAADSRQRLFDHKLIWTGTSQAWITTIDERSADPVLLGLMEHYRVYDANLISAFSHTEQMGFVVERLQFGHLGNTELIENDALKRLKLMVDDIYFAFTVAFVPRLWPHLNAYNDFILAWHSSGFDKFWEWKIAAEYMNAHRQNRIVASEKTNLDIGPVKLGIDNFIGLILLWCFGMICSLLTFLGELWRGQG

>DmelIR76b

MATGIELLVAAALCVACPPLNDSPPTNLIQMGENGTLSPVTELPMDVDASEAGFDADAPVETLETINRKKPKLREMLDWIGGKHLRIATLEDFPLSYTEVLENGTRVGHGVSFQIIDFLKKKFNFTYEVVVPQDNIIGSPSDFDRSLIEMVNSSTVDLAAAFIPSLSDQRSFVYYSTTTLDEGEWIMVMQRPRESASGSGLLAPFEFWVWILILVSLLAVGPIIYALIILRNRLTGDGQQTPYSLGHCAWFVYGALMKQGSTLSPIADSTRLLFATWWIFITILTSFYTANLTAFLTLSKFTLPYNTVNDILTKNKHFVSMRGGGVEYAIRTTNESLSMLNRMIQNNYAVFSDETNDTYNLQNYVEKNGYVFVRDRPAINIMLYRDYLYRKTVSFSDEKVHCPFAMAKEPFLKKKRTFAYPIGSNLSQLFDPELLHLVESGIVKHLSKRNLPSAEICPQDLGGTERQLRNGDLMMTYYIMLAGFATALAVFSTELMFRYVNSRQEANKWARHGIGRTPNGQSVAPSRWLRGWRRLNSGHGQLLGASTHGQNVTPPPPYQSIFNGGSHGDPLNRWRRPLANGNALGNGVLLGGDSEGGVRRLINGRDYMVFRNPNGQSQLVPVRSPSAALFQYSYTE

>DmelIR7a

MFHHLWLLMGLRSLAMGALHPPQPEAMTPLVAAALEILAEQVSPSQSTLAVMDLTQDAEHRDERQEQLMTIILRSVGSEMALRTFQKPPAEVPASFVVFLVNSAQAFNTLGFHFTDIHSTREFNFLILLTHRMSSRAERLQVLRDISRTCVRFHTSNVILLTEKRDGVVLVYAYRLLNMDCDLSVNLELIDIYKNGLFRHGHEARSFNRVLSLSGCPLQVSWYPLPPFVSFIGNSSDPEERAQIWRLTGIDGELIKLLASIFDFRILLEEPCNKCLSPDIKDDCSGCFDQVIISNSSILIGAMSGSHQHRSHFSFTSSYHQSSLVFIMHMSSQFGAVAQLAVPFTVIVWLALVVSSLLLVLVLWMRNRLVCGRSDLASHALQVLTTLMGNPLEARSLPRSSRLRILYAGWLLLVLVLRVVYQGKLFDSFRLPYHKPLPTEISELIRSNYTLINQEYLDYYPRELTVLTRNGSKDRFDYIQGLGKEGKFTTTSLIATMEYYNMMHWSTSRLTHIKEHIFLYQMVIYLRRHSLLKFAFDRKIKQLLSAGIIGYFVREFDACQYRKPFEEDYEVTPIPLDSFCGLYYISLIWLSAAVVAFILELLSQRIVWLRRIFE

>DmelIR7b

MKYWLYILSCCSLVASTMESSSDWDLAEALAQVVANSEMGRFKTLYIYTHTNSQSTGGHLEELLDQVLMIVPNNLQARRLLLQQSMEYKPYVHAVLALVDGLPSLSAIYARIRATQDLSHTLIYMSMPTDAYGEEMQATLRFLWRLSVLNVGVVLRPPGDHILMVSYFPFSALHGCQVISANVVNRYQVGTKRWASQDYFPSKLGNFYGCLLTCATWEDMPYLVWRPDGSGSFVGIEGALLQFMAENLNFTVGLYWMNKEEVLATFDESGRIFDEIFGHHADFSLGGFHFKPSAGSEIPYSQSTYYFMSHIMLVTNLQSAYSAYEKLSFPFTPLLWRAIGLVLILACLLLMLLVRWRHHHELPRNPYYELLVLTMGGNLEDRWVPQRFPSRLVLLTWLFATLVLRSGYQSGMYQLLRQDTQRNPPQTISEVLAQHFTIQLAEVNEARILASLPELRPEQLVYLEGSELQSFPALAQQSGSSARVAILTPYEYFGYFRKVHPMSRRLHLVRERIYTQQLAFYVRRHSHLVGVLNKQIQHAHTHGFLEHWTRQYVSAVDEKDESVARIASTSYSTLDGIDGDPSLSESEEDQQVAPVRQNVLSMRELAALFWLILWANLGAVVVFVLELLLPRIKLRKILRKMKSDIKKQISKLVRK

>DmelIR7c

MLHSAVHNVSLVYALVWAIDNYYGMATSTPLAVVQFPTSRESRRLHNDLIDAALGRSSGTGRIQFLLEDDRVEMTETDTDPPPPSGLTGRPIAIWFLDSLRSYFRLEMYLNQLGSPYKRNGFFLVIYTGLEDQPMESLKIMFRRLLNMYVLNVNVFLQRDGTVHLYTYYPYGPHHCQSSLPVYYTAFQDLAAPANGFGLTKPLFPRKLTNMHGCEMVVATFEHRPYVIIEDDPKTPGGRSIHGIEGLIFRSLAERMNFTIKLVEQKDKNRGEILPDGNFTGILKMMVDGEVNLTFVCFMYSKARSDLMLPSTSYTSFPIVLVVPSGGSISPMGRLTRPFRYIIWSCILVSLIFGFVLICLLKITALPGLRNLVLGRRNRLPFMGMWASLLGGLALYNPQRNFARYILVMWLLQTLILRAAYTGQLYLLLQDVEMRSPIKSLSEVLAKDYEFRILPALRTIFKDSMPTTNFHAVLSLEESLYRLRDEDDPGITVALLQPTVNQFDFRSGPNKRHLTVLPDPLMTAPLTFYMRPHSYFKRRIDRLIMAMMSSGIVARYRKMYMDRIKRVSKRRNLEPKPLSIWRLSGIFVCCAGLYLVALIVFILEILTTNHRRLRRAFNVINRYAA

>DmelIR7d

MDIRCVVALLLGLCKVQAVVWPHQHLLEEQLASQISATLQKIFINGLAVYNFGVFISTSYEEMDRDRVILVHQVLNRNLYPPNFPVAVVLASKMNRKITAQVFTQLLFVQNAEQAIAIAEGVNRNGLCVIVLLTSQPERPIMTKIFTYFMQERYNINVVILVPRLHGVQAFNVRPYTPTSCSSLEPVEIDIKDGDLWDVFPRRLKNLHGCPLSVIVWDIPPYMRINWKSSDPMDGLDGLDGLLLRIVARKMNFTLKLIPNEPNGLIGGSSFMNGTFTGAYKMLRERRANITIGCAACTPERSTFLEATSPYSQMSYIIVLQARGGYSIYEVMLFPFEKYTWLLLSTILGLHWIVGSRWRMPSPILAGWMLWIFVIRASYEASVFNFIQNSPVKPSPRTLDQALSGGFRFITDHASYRMTLKIPSFQGKTLISAGQPVDVFDALLKAPWKTGAFTSRAFLADHLVRHRKHRNQLVILAEKIVDNMLCMYFPHGSYFAWEINKLLFNMRSFGIFQHHSQILAWDNLPTTTDTDTPGKRIHSSTESVATGFAESMSFVVAALNCLMGALCISIVVFGLELLSRRRHWTGLEWLFERV

>DmelIR7e

MNISALLNSYYDLSGEQMNHINEFVARAVLHVVHHYILSVTPSLVLTLCCRSNHTCNFYNKMMSTLFREWGLAPLQIVNVLRGVPWHPVPGRRHFNVIFTDSFAAFEEIRMEYYSREYNYNEHYFIFLQARDRLLQGEMRLIFDYCWRYRLIHCSIQVQKSNGDILFYSYYPFGEHGCSDMEPQLINRYNGSMLVEPDLFPRKLRNFFGCPLRCALWDVPPFLTLDEDQEEVLRVNGGYEGRLLLALAEKMNFTIAVRKVHVNMRDEALEMLRRDEVDLTLGGIRQTVARGMVATSSHNYHQTREVFGVLASSYELSSFDILFYPYRLQIWMGILGVVALSALIQLIVGRMLRERMGSRFWLNLELVFVGMPLLECPRSHTARLYCVMLMMYTLIIRTIYQGLLYHLIRTHQLNRWPQTIESLVQKNFTVVLTPIVQEVLDEIPSVQHMRFRLLEANSELDPLYFLEANHQLRQHVTASALDIFIHFNRLSADKVHQRGEQGSGAHFEIVPEDIISMQLTMYLAKHSFLIDQLNEEIMWMRSVGLLSVWSRWELSESYLRNEQSFQVLGTMELYAIFLMVLVGLIVGLLVFILELVSMRSIYLRKLFT

>DmelIR7f

MNTTSDSNAGSSLSSGSGYSIYKSYLENSRIDMQGEDANLYVARALRLVIENVLAQLSTTLVVTISTRHLGTAHWFEYMMNILMDSWRMVAVQLLRIRPDLVVNPVPGRKRVSLLMVDSYQGLLDTNITASNANFDDPDYYFIFLQARDHLIPKELQLILDHCLAHFWLHCNVMIQTAQVEVLVYTYYPYTADACQKAYPIPVNTFDGRKWKASQMFPDKLSQMHGCPLTVLTWHQPPFVELVWDPKHNRSRGSGFEIQLVEHLARRMNFSLELVNIALLRPNAYRLAEGSSEGPIEKLLQRNVNISMGYFRKTARRNQLLTTPMSYYSANLVAVLQLERYRIGSLALLVFPFELSVWMLLLLALLIHLGIHLPSARRGNEEDGGGGLQVVALLLGAALARLPRSWRHRFIAAHWLWASIPLRISYQSLLFHLIRLQLYNTPSFSLDQLLAEGFQGICTANTQRLLLEMPQLARDPDSIQSVDTPFDWDVLNVLTRNRNRKIFAVANQDVTLSFLHSSAHPNAFHVVKQPVNVEYAGMYMPKHSFLYEKMDDDIRRLDASGFIHAWRRASFASVHRKEQVHMTSRRYINHAKLSGIYMVMAGLYLLAGLLFAGEVLLRQRN

>DmelIR7g

MNVTSLLNFESMKYIGAQTQAASINHHVAQALRVFIEDFYQRIAPAFIVVLSCRRPSPMNFYRNIMQLLYESVDTMIVQLVLVELGRPRRIAGPRTHNLLLVDSLDALLDIEIHTYTAQSDTSEYYFIFLQQRDALIPHDMQGVFAYCWRHQLINCNVMTQSSGGQVLLHTYFPYAPGQCNDSQPTRINMFLGESWKHRDYFPSKLHNLNGCPLIVLARKVSPFLDLDEGQRELRGLEGRLLQELSRRMNFSIQFSGLQDQLKNRTTWTEKQLLQKLVQERIAHLAIGYVRKRIQYATNLTPVFPHYSNRVVGCLLLNAHNLTSLEIWSFPFQALTWICLLLFASWLIFGLIVRSMYSALLFFILRYHLHQRLPGNLQDLTHGDYAAVMGRTTLQDLREVPSLQDLLGLKSVIVTSEREEEVLRTLDRCTLREGAGSHPLFFGLISQDALLHLTQRGHRAGAYHIIPQDVLEQQLAIYLQKHSHLASHLDHLVMSIRSVGLVHHWAGQMASERYFRSRFLYREKRIRQPDLWAVYILTAGLYLLSLVVFICELLASRRAGL

>DmelIR84a

MIKLQVKVISWPLIILTAFLRVLQIESINTNFLELAAFEDFLRSEHLSHVLVVRGDDADGDWKIECHQKLLANYRVQFYRPEMSANFEDLMFYGSPRTAVLVLNSEHVLVRRQVFGVASEAGYFNNSLAWFILGSGRESLPVEQLIDQLLSGYRMGIDADITVALRGPDNASMLFYDVYRISRQANTPLIIEKKGLWTHSGGYQKFGNFKNTWVIRRRNFLNVTLIGSTVLTEKPPGFGDMEYLADDKQLQQLDPMQRKTYQLFQLVERMFNLSLAISLTDKWGELLDNGSWSGVMGQVTSREADFAVCPIRFVLDRQPYVQYSAVLHTQNIHFLFRHPRRSHIKNIFFEPLSNQVWWCVLALVTGSTILLLFHVRLERMLSNMENRFSFVWFTMLETYLQQGPANEIFRLFSTRLLISLSCIFSFMLMQFYGAFIVGSLLSESARSIVNLQALYDSNLAIGMENISYNFPIFTNTSNQLVRDVYVKKICKSGEHNIMSLQQGAERIIQGRFAFHTAIDRMYRLLLELQMDEAEFCDLQEVMFNLPYDSGSVMPKGSPWREHLAHALLHFRATGLLQYNDKKWMVRRPDCSLFKTSQAEVDLEHFAPALFALALAMVASALVFLLELFLHWLPDFRRRLGTMST

>DmelIR85a

MSIQWLKHILLLAILVNLAGTRENHIPLDLKKSSIVMVKMSQILCKARIKVLFVYFENQTSHEHTGQILKEVTKCDISNQNTPLEAVKDDGILMYMVMITTNISQPLELSLIRKKSAAKHRSHVFLLVRDADTVSDAWMRASFRQFWKIWLLNIVILYWRDGRLNAYRYNPFMDNYLIPVDNKPNEVPTLEQLFPKTIPNMQRKPLRMCIYKDDVRAIFWRQGTILGTDGLLAAYVAERLNATMMITRPHSYNNHNLSSDICFLEVAKEYVDVAMNIRFLVPDTFRKQAESTVSHTRDDLCVIVPKAKTAPTFWNIFRSFGSLVWALILVSVLVANVFCYILKSEVGRVPMQLFAGALTMPMTQIPPNHSIRLFLIFWLYFGLLICSAFKGNLTSMMVFQPYLPDINQLGALARSHYHIIIRPRHVKHIQHFLTLGHKHESRIREQMLEVSDTQMYEMMRNNDIRFAYLEKYHIARFQVNSRVHMHLGRPLFHLMNSCLVPFHAVYIVPYGSPYLGFLDSLIRSSHEFGFERYWDRIMNSAFIKSGVKVVNRRRGSGNDEPVVLKLQHFHAVFALWLVGIGMACIVLAWEHLTHNYNLAVTKRRD

>DmelIR87a

MSTPEQRFWLAALLFLLSQHSEVRGFGINLMKVQTEDKGQEACILALLRKYFDSGDGLSGSVLCINRNYQLPNIEEQLLRGVNNYENYPWSLLITNSREGPSPAKFLMNEKPQCYFLIVDNLEDEDLDEVFEHWKGMVNWNPLAQFVVYLASLEETDEEMNDLMVELLLTFINKKIFNVNVIGQSEENQFYYGKTVFPYHPDNNCGNRVISVELLDACDYPSEETDSEDENDEDEGDGAQEEDDGPQEEGDGEQEEEDGPQEQEDGDQAKGDEGQENDDGGLENKVENEFRIGASDDDELENDLSSNSSEPEAIIEEFFRAKFEDKFPRDLSGCPLTASFRPWEPYIFRNSEEQPVDDYYYGLQGDEDDYNDTSPNYGESDDESYADPGEDGDGAIPDTETQSGGKLKLSGIEYEMVQTIAERLHVSIEMQGENSNLYHLFQQLIDGEIEMIVGGIDEDPSISQFVSSSIPYHQDELTWCVARAKRRHGFFNFVATFNADAGFLIGIFVVTCSLVVWLAQRVSGFQLRNLNGYFPTCLRVLGILLNQAIPAQDFPITLRQLFALSFLMGFFFSNTYQSFLISTLTTPRSSYQIHTLQEIYSNKMTVMGTSEHVRHLNKDGEIFKYIREKFQMCYNLVDCLNDAAQNEHIAVAVSRQHSFYNPRIQRDRLYCFDRRESLYVYLVTMLLPKKYHLLHQINPVIQHIIESGHMQKWARDLDMRRMIHEEITRVREDPFKALTFDQFRGAIAFSGGLLLVASCVFAFELCYVKYVYRTEKRERKTKKITKKVHNIKIQHD

>DmelIR8a

MELPLLVLLLALRFAGSEVLKITFWIEPVQRAEFDTDIAMVLKELDALRLDVKVDDTTLTLTRSEDGLDMQRFCEILSTVGASAVIDLTYSHWEEGYNLVRSLGIGYVRLERIMRPFLDMFGDFMRQKRANNVAMVFMNARDAVEAMQQMLVGYPFRTLIMDASQTDPGQHFLERIRSLRPAPTYIALFARAAAMNGIFEKVQKADLFQRPLEWHFVFLDTRDRVFKYRRQAELCTRFTLNPRAICRSMPMPDLYCGSGFTMQRAMLLNVLRSLINAAQVSPGYPLAIYQDCNATASSSEVSDPLEKDDYNWLDMVHWSNFLAYAPPLPHIQDQFQSPVPGLTFAVNISAGYYSSEHEAKTDLAAWSSVGEMRLLNETISPARRFFRIGTAESIPWSYLRREEGTGELIRDRSGLPIWEGYCIDFIIRLSQKLNFEFEIVAPEVGHMGELNELGEWDGVVGDLVRGETDFAIAALKMYSEREEVIDFLPPYYEQTGISIAIRKPVRRTSLFKFMTVLRLEVWLSIVAALVGTAIMIWFMDKYSPYSSRNNRQAYPYACREFTLRESFWFALTSFTPQGGGEAPKAISGRMLVAAYWLFVVLMLATFTANLAAFLTVERMQTPVQSLEQLARQSRINYTVVKDSDTHQYFVNMKFAEDTLYRMWKELALNASKDFKKFRIWDYPIKEQYGHILLAINSSQPVADAKEGFANVDAHENADYAFIHDSAEIKYEITRNCNLTEVGEVFAEQPYAVAVQQGSHLGDELSYAILELQKDRFFEELKAKYWNQSNLPNCPLSEDQEGITLESLGGVFIATLFGLVLAMMTLGMEVLYYKKKQNALEITQVRPVNDSSGSGGNSSTAPPTATSTTKQAWHIPVLEAEEKPAKVSPPPSFETATFRGKKLPARITLGDGKFKPRHGLYARRNLGASDSHSGYME

>DmelIR92a

MLLQPLVMHLSQLLRIIVGQYFAEFPSILIVYNNSASTTPLQLEYLSALELVLRELSKPIRLQWINVAFLKDLNDLEDQVMGALNSSVTEGFITILSQTHHFIHARYYATRNANVRLKDKRYLFLCEDESPAELLCMDILQFYPHHLMVRPGTETAPTGPTGPHPDPRRGGGASVSTKNKDDGEGGAGNKTTSPYRDINFELWTQKFVGAVGNLDALLLDAFLPNETFANRVELYPNKLLNLQRRSLLVGSITYVPYTITNYVPAGQGDVDPIHPQWPNRSLTFDGAEANVMKTFCQVHNCHLRVEAYGADNWGGIYDNESSDGMLGDIYEQRVEMAIGCIYNWYDGITETSHTIARSSVTILGPAPAPLPSWRTNIMPFNNRAWLVLISTLVICGTFLYFMKYVSYRLRYSGTQVKFHHSRKLEKSMLDIFALFIQQPSAPLSFDRFAPRFFLATILCATITLENIYSGQLKSMLTFPFYSAPVDTIEKWAQSGWKWSAPSIIWVHTVQSSDLETEQILARNFEVHDYSYLSNVSFMPNYGFGIERLSSGSLSVGDYVSTEALENRIVLHDDLYFDYTRAVSIRGWILMPELNKHIRTCQETGLYFHWELEFIDKYMDKKKQEVLMDLANGHKVKGAPQALDVRNIAGALFVLAFGVAFAGCALVAELLIHRMDLSK

>DmelIR93a

MNPGEMRPSACLLLLAGLQLSILVPTEANDFSSFLSANASLAVVVDHEYMTVHGENILAHFEKILSDVIRENLRNGGINVKYFSWNAVRLKKDFLAAITVTDCENTWNFYKNTQETSILLIAITDSDCPRLPLNRALMVPIVENGDEFPQLILDAKVQQILNWKTAVVFVDQTILEENALLVKSIVHESITNHITPISLILYEINDSLRGQQKRVALRQALSQFAPKKHEEMRQQFLVISAFHEDIIEIAETLNMFHVGNQWMIFVLDMVARDFDAGTVTINLDEGANIAFALNETDPNCQDSLNCTISEISLALVNAISKITVEEESIYGEISDEEWEAIRFTKQEKQAEILEYMKEFLKTNAKCSSCARWRVETAITWGKSQENRKFRSTPQRDAKNRNFEFINIGYWTPVLGFVCQELAFPHIEHHFRNITMDILTVHNPPWQILTKNSNGVIVEHKGIVMEIVKELSRALNFSYYLHEASAWKEEDSLSTSAGGNESDELVGSMTFRIPYRVVEMVQGNQFFIAAVAATVEDPDQKPFNYTQPISVQKYSFITRKPDEVSRIYLFTAPFTVETWFCLMGIILLTAPTLYAINRLAPLKEMRIVGLSTVKSCFWYIFGALLQQGGMYLPTADSGRLVVGFWWIVVIVLVTTYCGNLVAFLTFPKFQPGVDYLNQLEDHKDIVQYGLRNGTFFERYVQSTTREDFKHYLERAKIYGSAQEEDIEAVKRGERINIDWRINLQLIVQRHFEREKECHFALGRESFVDEQIAMIVPAQSAYLHLVNRHIKSMFRMGFIERWHQMNLPSAGKCNGKSAQRQVTNHKVNMDDMQGCFLVLLLGFTLALLIVCGEFWYRRFRASRKRRQFTN

>DmelIR94a

MALPKQLKFINIFLVLLIIYGSSDGTENQHEIFLNRLLQAVHNERSVETLFLLHHSNLANCSLQDWNPPRIPTIRSNELTVFNVEKTFNHNALALVCLMKNSYREILNTLAKSFDCMRQERIILMIHRKSDSKFIEDITHEVKNLQFLHLIVLIVQEKYNGQVFASTLRLQSFPEPHFKRIRNVFAIQRIFYRPINFHGKVLNAIPNDIPILFVALNEMFTEYARRYNSTLRIQNRTIKEDIEITEDNYDIDMKIQLHNSQNFLHHMNIAMDIGSNSLIILVPCATELRGLDIFKELGVRTLTWLALLFYIIFVLVEMLFVFISNRFNGRNFTMRYTNPLINLRAVRAILGQTSPISNRYSLSIQHFFVFMSLFGTLFGGFFDCKLRSFLTKRPYYSQIENFSELRKSGVTVVVDHTTRQFIEQEINANFFRDEVPNVRTTTIQELINHVYSYDRKFAFVANSIPWRTFREEMKSINQKILCDSKNLTILENVPLTFSIRRNAIFSHHLRNFIINAADSGMITCWFKMAGKVIRKHIKTTLRESEQQPSHLPLSFDHFKWLWAVLCIAYVMSFMVFVMEILWSKYQRRTRSVSIV

>DmelIR94b

MSLIFNLLFILILSQAVSQETEFLQLKYLNNIVRSMIKLHKMETLVIVKHHLDNNCSLQNWNAHGMGIIRTNDQGKLIMKDTFNSRTLAIICIGQNSHITLLRNVFETFGKVQQKKIILWTQMELKEKFFQEISKKSRDLKLLNLLVLKAVTKDKLLIYRLNPFPSPHFKRIENIWTPNDTLFMDTKFNFHGMTAVVKHDYNWTIQMGNIRKFPISRIEDKEVIEFALKYNLTLQFFNDVERFDIELRKRIILKSNSTQPIDSGIPMVFSSLLIVVPCGNYLSIQDVIKVSGIEKWIFYIILVYVIFVLIEITFLGVTILISRQSRHQMIPNTLVNLCAFRAILGLPFPETRRTSLSLRQLFLAIALFGMIFSIFINCKLSSMLTNPCPRPQVNNFEELKTSGLTVVMDHDAENFIEKEIGVDFFNQYMPRKVTLTFTERAKLLFSLKGNHAFTLFSESFAIIESYQRSKGLRAHCTSEDLIVAERVPRIYILENNSILDRPLRRFIRQMQESGITNHWLKNIPSSLEKNLMQITIPYDRERVHPLSIEHLTWLWCILILGYSISMIVFFVEMSLKRRKKNLENRAPNICIC

>DmelIR94c

MSKVFKLLVLPLIYLSLTKGSKNPQLKFLRELINVIEEGREIRTIMVIKHSRDEYCHLDQWNPRGSPILRTNEMGSIRISGYFNDQAVILACMGENSDYGLLKSLANAMDNMRQERIILWSEREPTKMLMDYISQQADRYNFAQIIIVTMNEDVDAVPSLHQLNPYPTPRFRQITNISNIRRTSFFGCGLSFQGKTAILKESVVSNIRFKVWSPSGPIPLSELKDYEIVQFAVKYNLSLKLYDQNESKSDHFDIQLGPLFITKDFPTQMAFVSPNTACSLIVIVPCSPKWRFMDVLHKLGVLKLIGCLLIAYAVFVLIETLILWLTHRISGREVRLTSLNQLLNPRAFRGILGLPFPEFRRSSISLRQLFLVISVFGLVYSNFVSCTLSALLTKPAQNPQVRNFKELRDSGLITIMDKYTHSFIEKHIDPEFFDHVLPHYLILQKKEALRMIWNFNDSYSYVMYTTTWKSLNTVQKSFDERVFCESESLTIAWNLPRMYVLGNNSVLKWMLSRYITYMPQTGIPDSWTEQLPKVLKLLYNVTSPRRIKEGAVPLSIQHLSWIWHLLFIGESIATLVFIVEILLQKSNQHTSNMRERSSEDDDFV

>DmelIR94d

MGQLHLLLVALVLLSPGGDSFYHSLIHHLNRELKIEYVLLLGNFDTTWLDILWQLPVSVLQIKEHSRETYSLLENPSHNVLTIAFVNDSPEDILEILYRNLRMLNTQPVLLVIRKSTIRVNSLLEWCWHHQLLKVVAIAQDFMESLIVYSYNPFPVLQFIERRLDNSTVIFEKRLENLHGYEVPIALGGSSPRLIVYRDLEGKLIFSGPVGNFMKSFEQRYNCRLVQPYPFDESAISPARDLIASVQNGSVQIALGAIYPQVPYTGYSYPIELMSWCLMMPVPEEVPHSQLYSMVFSPMAFGITIVAMVLISLTLSMALRLHGYRVSFSEYFLHDSCLRGVLSQSFYEVLRAPALIKAMYLVICLLGLLITSWYNSYFSTFVTSAPRFPQLTSYESIRHSNIKIVIWKPEYEMLLFFSENMEKYSSIFQLQEDYKEFLHLRDSFDTRYGYMMPMEKWSLMKEQQRVFSSPLFSLQDDLCVFHTVPIVFPMVKNSIFKEPFDRLILDVTATGLLSRWRDMSFTEMIKAGQLGLEDRGHPKEFRAMKVGDLIQIWRFVGWMLGLATIVFLLELICFWRHKMWQNMKYMFCRNKNI

>DmelIR94e

MWQQVLLAETSNWFRSDVLQRFWTHLRVEIRFRTMLNYRLESCDCWFDNVLGSDNSTALLWNDQTYPHYLRRRQDTDILVVSCLRFHQYQEVLLALSLMLDQMRSMPVVLQLCGDEDSMQELNSARLLLKHSQDLKMPNVVLLSSTFFTSATLYSYEMFPEFNVQKLVYQAYLTLFPYKLGNLKGHPIRTVPDNSEPLTIVRKTLNGSIAIDGLVWQFMIEFAKHINATLQLPIEPHPEKSIKLVQILDLVRNQTVDIAASLRPYSLNVQRSSTHIYGSPMMVGNWCMMLPTERVIGSHEALTRLMKSPWTWLILLLFYSVHRFLAQKTRLRSSLIHLIKLLINLSLICFLQAQLSAYFIGPQKVNHISNMQQVEESGLKIRGMRGEFMEYPIDMRSRYASSFLLHDLFFDLAQYRNSLNTSYGYTVTSVKWELYKEAQRHFRRPLFRYSEEICVQKLSLFSLIQQSNCIYCYRSRIFILRMHEAGLIRLWYRRSYYVMVTAGRFPIGDLSTVHRAQPIRWTEWQNVVLLHGVGLLFSVVVFVIELTVHYANVCLNNL

>DmelIR94f

MSTAVNSVHSKLVSLISRGQELTSIFFYAPAKEKCHLEDTISSATWGLPLVIWRTDRTVILNGFIGEGLLVLACLPGFHWRALLGSLARSLKYLRQARILIELMQDRDEFLVSEVLQFCLSQDMINVNAIFDDFPETENLSSFEAYPSFEVVNQTFTPDTQVSDLYPNKMLNLRGGVIRTMPDYSEPNTILYQDKEGNKEILGYLWDLLEAYAHKHNAQLQVVNKYADDRPLNFIELLDAAQSGIIDVGASIQPMSMGSLSRMHEMSYPVNQASWCTMLPVERQLHVSELLTRVIPYPTLALLLLLWIFYEVLRGRWRRHSRLQSIGWLVLATLVSSNYVGKLLNLFTDPPSLPPVNSLAALMESPVRIISIRSEYSAIEFTQRTKYSAAFHLALHASILIGLRNAFNTSYGYTITSEKWKIYEEQQKRSSKPVFRYSKDLCFYEMIPFGLVIPENSPHRAPLHSYTLLLRQAGLHDFWVNRGFSYMVKAGKINFTAVGERYEAKTLTITDLRNVFIIYVSVLLISLILFTCELFVSWVNYWLGF

>DmelIR94g

MLSNISFSSAPELVDLYGLVLKFLVSSETTLFYFNPTGQKCSWETLPRTILSNHPQIIWFREETYPGLYKRHSSNLFVMACLSSTSYDGQLQLLAESLTRYRSVRVLIEVQDKEGSFLASQILLLCQQHSMLNVVLYFSRWTRTLNVFSYLAFPYFKLLKQRLSGSLRPKIFINQLKDLQGYKIRVQPDLSPPNSFSYRDRHGECQVGGFLWRIVENFSKSLKGDTQVLYPTWAKAKVSAAEYMIQFTRNGSSDIGVTTTMITFKHEERYRDYSYPMYDISWCTMLPVEKPLSVEILFSHVLSPGSALLLILAFILFFLIVPQLIKCLGITFRGRLIGMASRIFALVMLCSSSAQLLSLLMSPPLHTRIKSFDDLLTSGLKIFGIRSELYFLDGGFRAKYASAFHLTENPNELYDNRNYFNTSWAYTITSVKWNVIEAQQRHFAHPVFRYSTDLCFSSETPWGLLIAPESFYREPLQHFTLKINQAGLITQWMTQSFHEMVRAGRMTIKDYSRTNLMKPLRIQDLRKCWVIFAVGLGTSTVVFTIELLLIYTNVFLNSL

>DmelIR94h

MLSNISFSSAPELVDLYGLVLKFLVSSETTLFYFNPTGQKCSWETLPRTILSNHPQIIWFREETYPGLYKRHSSNLFVMACLSSTSYDGQLQLLAESLTRYRSVRVLIEVQDKEGSFLASQILLLCQQHSMLNVVLYFSRWTRTLNVFSYLAFPYFKLLKQRLSGSLRPKIFINQLKDLQGYKIRVQPDLSPPNSFSYRDRHGECQVGGFLWRIVENFSKSLKGDTQVLYPTWAKAKVSAAEYMIQFTRNGSSDIGVTTTMITFKHEERYRDYSYPMYDISWCTMLPVEKPLSVEILFSHVLSPGSALLLILAFILFFLIVPQLIKCLGITFRGRLIGMASRIFALVMLCSSSAQLLSLLMSPPLHTRIKSFDDLLTSGLKIFGIRSELYFLDGGFRAKYASAFHLTENPNELYDNRNYFNTSWAYTITSVKWNVIEAQQRHFAHPVFRYSTDLCFSSETPWGLLIAPESFYREPLQHFTLKINQAGLITQWMTQSFHEMVRAGRMTIKDYSRTNLMKPLRIQDLRKCWVIFAVGLGTSTVVFTIELLLIYTNVFLNSL

>DmelGluRIIA

MRLCPVVIYAFIIIIGFLEGIIALGGDDRNEITVGAIFYENEKEIELSFDQAFREVNNMKFSELRFVTIKRYMPTNDSFLLQQITCELISNGVAAIFGPSSKAASDIVAQIANATGIPHIEYDLKLEATRQEQLNHQMSINVAPSLSVLSRAYFEIIKSNYEWRTFTLIYETPEGLARLQDLMNIQALNSDYVKLRNLADYADDYRILWKETDETFHEQRIILDCEPKTLKELLKVSIDFKLQGPFRNWFLTHLDTHNSGLRDIYNEDFKANITSVRLKVVDANPFERKKTRLTKVDQILGNQTMLPILIYDAVVLFASSARNVIAAMQPFHPPNRHCGSSSPWMLGAFIVNEMKTISEDDVEPHFKTENMKLDEYGQRIHFNLEIYKPTVNEPMMVWTPDNGIKKRLLNLELESAGTTQDFSEQRKVYTVVTHYEEPYFMMKEDHENFRGREKYEGYAVDLISKLSELMEFDYEFMIVNGNGKYNPETKQWDGIIRKLIDHHAQIGVCDLTITQMRRSVVDFTVPFMQLGISILHYKSPPEPKNQFAFLEPFAVEVWIYMIFAQLIMTLAFVFIARLSYREWLPPNPAIQDPDELENIWNVNNSTWLMVGSIMQQGCDILPRGPHMRILTGMWWFFALMMLSTYTANLAAFLTSNKWQSSIKSLQDLIEQDKVHFGSMRGGSTSLFFSESNDTDYQRAWNQMKDFNPSAFTSTNKEGVARVRKEKGGYAFLMETTSLTYNIERNCDLTQIGEQIGEKHYGLAVPLGSDYRTNLSVSILQLSERGELQKMKNKWWKNHNVTCDSYHEVDGDELSIIELGGVFLVLAGGVLIGVILGIFEFLWNVQNVAVEERVTPWQAFKAELIFALKFWVRKKPMRISSSSDKSSSRRSSGSRRSSKEKSRSKTVS

>DmelCG3822

MRSSGVLVLPLLLLQLILNCRKAQSLPDIIKIGGLFHPADDHQELAFRQAVDRINADRSILPRSKLVAQIERISPFDSFHAGKRVCGLLNIGVAAIFGPQSSHTASHVQSICDNMEIPHLENRWDYRLRRESCLVNLYPHPNTLSKAYVDIVRHWGWKTFTIIYENNDGIVRLQELLKAHGMTPFPITVRQLSDSGDYRPLLKQIKNSAEAHIVLDCSTERIHEVLKQAQQIGMMSDYHSYLVTSLDLHTVNLDEFRYGGTNITGFRLINEKIVSDVVRQWSIDEKGLLRSANLTTVRSETALMYDAVHLFAKALHDLDTSQQIDIHPISCDGQSTWQHGFSLINYMKIVEMKGLTNVIKFDHQGFRTDFMLDIVELTPAGIRKIGTWNSTLPDGINFTRTFSQKQQEIEANLKNKTLVVTTILSNPYCMRKESAIPLSGNDQFEGYAVDLIHEISKSLGFNYKIQLVPDGSYGSLNKLTGEWNGMIRELLEQRADLAIADLTITFEREQAVDFTTPFMNLGVSILYRKPIKQPPNLFSFLSPLSLDVWIYMATAYLGVSVLLFILAKFTPYEWPAYTDAHGEKVESQFTLLNCMWFAIGSLMQQGCDFLPKALSTRMVAGIWWFFTLIMISSYTANLAAFLTVERMDSPIESAEDLAKQTRIKYGALKGGSTAAFFRDSKISTYQRMWSFMESARPSVFTASNGEGVERVAKGKGSYAFLMESTSIEYVTERNCELTQVGGMLDTKSYGIATPPNSPYRTAINSVILKLQEEGKLHILKTKWWKEKRGGGKCRVETSKSSSAANELGLANVGGVFVVLMGGMGVACVIAVCEFVWKSRKVAVEERLSAILNE

>DmelCG5621

MWFVKMISTEASFPLGFILTSLLLAFPGCRGERTNVGLVYENTDPDLEKIFHLAISKANEENEDLQLHGVSVSIEPGNSFETSKKLCKMLRQNLVAVFGPTSNLAARHAMSICDAKELPFLDTRWDFGAQLPTINLHPHPATLGVALRDMVVALGWESFTIIYESGEYLPTVRELLQMYGTAGPTVTVRRYELDLNGNYRNVLRRIRNADDFSFVVVGSMATLPEFFKQAQQVGLVTSDYRYIIGNLDWHTMDLEPYQHAGTNITGLRLVSPDSEQVQEVAKALYESEEPFQNVSCPLTNSMALVYDGVQLLAETYKHVNFRPVALSCNDDSAWDKGYTLVNYMKSLTLNGLTGPIRFDYEGLRTDFKLEVIELAVSGMQKIGQWSGEDGFQENRPAPAHSLEPDMRSLVNKSFVVITAISEPYGMLKETSEKLEGNDQFEGFGIELIDELSKKLGFSYTWRLQEDNKYGGIDPKTGEWNGMLREIIDSRADMGITDLTMTSERESGVDFTIPFMSLGIGILFRKPMKEPPKLFSFMSPFSGEVWLWLGLAYMGVSISMFVLGRLSPAEWDNPYPCIEEPTELENQFSFANCLWFSIGALLQQGSELAPKAYSTRAVAASWWFFTLILVSSYTANLAAFLTVESLVTPINDADDLSKNKGGVNYGAKIGGATFNFFKESNYPTYQRMYEFMRDNPQYMTNTNQEGVDRVENSNYAFLMESTTIEYITERRCTLTQVGALLDEKGYGIAMRKNWPYRDTLSQAVLEMQEQGLLTKMKTKWWQEKRGGGACSVSDQPNLNLWQLVHVWVCAGRGRGLWRRGPGD

>DmelCG9935

MLIASGFLLFQFLSYGLGVPPLVRIGAIFSNQPGMYNSELAFRYAIHRLNMDKSLLPETTVDYYVEYVNRFDSFETVQKVCKLIRVGVQAVFSPTDSVLATHINSICDALDIPNIGRSAHDFSINVYPSKQLVNYAFNDVIQYLNWTRFGILHEKENGIINLHQLSRSFHGEVHMRQVSRDSYVSALNEFKGKEIHNIIIDTNSNGISILLKNILQQQMNEYKYHYLFTSFDLETYDLEDFKYNFVNITSFRLVDTADVGVKQILKDIGLYSHHIFKKPYLNLHIKKSTILESEPALMFDSVYVFAIGLQTLEQSHSLTLLNISCEEENSWDGGLSLINYLNAVEWKGLTGPIQFKDGQRVQFKLDLIKLKQHSIVKVGEWTPHGHLNITEPSMFFDAGSMNVTLVVITILETPYVMMHYGKNFTGNERFYGFCVDILETISREVGFDYILDLVPDRKYGAKDPETGEWNGMVAQLMKYKADLAVGSMTITYARESVIDFTKPFMNLGISILFKVPTSEPTRLFSFMNPLAIEIWIYVLIAYFLVSLCIYIVGKLSPIEWKCINACDLENISIGNQFSLTDSFWFTIGTFMQQSPDIYPRAMSTRIISSTWGFFSLIIVASYTANLAAFLTTERMINPIENAEDLASQTEISYGTLDSGSTMTFFRVCN

>DmelCG11155

MVRKKREIVIKENIQGRSYLKKICCSYIILSILVISNALPPVIRVGAIFTEDERESSIESAFKYAIYRINKEKTLLPNTQLVYDIEYVPRDDSFRTTKKVCSQLEAGVQAIFGPTDALLASHVQSICEAYDIPHIEGRIDLEYNSKEFSINLYPSHTLLTLAYRDIMVYLNWTKVAIIYEEDYGLFNLMHSSTETKAEMYIRQASPDSYRQVLRAIRQKEIYKIIVDTNPSHIKSFFRSILQLQMNDHRYHYMFTTFDLETYDLEDFRYNSVNITAFRLVDVDSKRYLEVINQMQKLQHNGLDTINGSPYIQTESALMFDSVYAFANGLHFLNLDNHQNFYIKNLSCTSDQTWNDGISLYNQINAAITDGLTGTVQFVEGRRNIFKLDILKLKQEKIQKVGYWHPDDGVNISDPTAFYDSNIANITLVVMTREERPYVMVKEDKNLTGNLRFEGFCIDLLKAIATQVGFQYKIELVPDNMYGVYIPETNSWNGIVQELMERRADLAVASMTINYARESVIDFTKPFMNLGIGILFKVPTSQPTRLFSFMNPLAIEIWLYVLAAYILVSFALFVMARFSPYEWKNPHPCYKETDIVENQFSISNSFWFITGTFLRQGSGLNPKATSTRIVGGCWFFFCLIIISSYTANLAAFLTVERMISPIESASDLAEQTEISYGTLEGGSTMTFFRDSKIGIYQKMWRYMENRKTAVFVKTYEDGIKRVMEGSYAFLMESTMLDYAVQRDCNLTQIGGLLDSKGYGIATPKGSPWRDKISLAILELQEKGIIQILYDKWWKNTGDVCNRDDKSKESKANALGVENIGGVFVVLLCGLALAVVVAIFEFCWNSRKNLNTENQSLCSEMAEELRFAMHCHGSKSRHRPRKRSCLNCSSVPTYVPSNVSTSNVGVYYNYFN

>DmelClumsy

MEKMVCNLISQGVIAIFGPSTGSSSDIIASICDTLDIPHIVYDWIPNESIPDREHSTMTLNVHPDNLLLSQGLAEIVQSFAWRSFTVVYETDKELQQLQDILQVGEPISNPTTVKQLGPGDDHRPFLKEIKLSTDNCLILHCAPDNLLKILQQANELKMLGEYQSVFIPLLDTHSIDFGELSGVEANITTVRLMDPSDFHVKNVVHDWEEREKREGRYFKVDPNRVKSQMILLNDAVWLFSKGLTELGIFEELTAPDLECRRKKPWPFGKRIIEFIKARSEETSTGRIDFNENGQRSFFTLRFMELNSDGFLDLATWDPVNGLDVLNDDEESEKRVGQKLSNKTFIVSSRLGAPFLTLREPQEGEILTGNSRYEGYSIDLINEIAKMLNFKFEFRMSPDGKYGALNKVTQTWDGIVRQLIDGNADLGICDLTMTSSRRQAVDFTPPFMTLGISILFSKPPTPPTDLFSFLSPFSLDVWIYMGSAYLFISLLLFALARMAPDDWENPHPCKEPEEVENIWSIMNTTWLSIGSLMGQGCDILPKAASTRLVTGMWWFFALMMLNSYTANLAAFLTNSRQANSINSAEDLAAQSKIKYGAMAGGSTMGFFRDSNFSTYQKMWTAMESASPSVFTKTNDEGVERVQKGKNLYAFLMESTTLEYNVERKCDLVQIGGWLDYKSYGIAMPFTPLLARLHKSNGLMALCPF

>DmelGluR1

MHSRLKFLAYLHFICASSIFWPEFSSAQQQQQTVSLTEKIPLGAIFEQGTDDVQSAFKYAMLNHNLNVSSRRFELQAYVDVINTADAFKLSRLICNQFSRGVYSMLGAVSPDSFDTLHSYSNTFQMPFVTPWFPEKVLAPSSGLLDFAISMRPDYHQAIIDTIQYYGWQSIIYLYDSHDGLLRLQQIYQELKPGNETFRVQMVKRIANVTMAIEFLHTLEDLGRFSKKRIVLDCPAEMAKEIIVQHVRDIKLGRRTYHYLLSGLVMDNHWPSDVVEFGAINITGFRIVDSNRRAVRDFHDSRKRLEPSGQSQSQNAGGPNSLPAISAQAALMYDAVFVLVEAFNRILRKKPDQFRSNHLQRRSHGGSSSSSATGTNESSALLDCNTSKGWVTPWEQGEKISRVLRKVEIDGLSGEIRFDEDGRRINYTLHVVEMSVNSTLQQVAEWRDDAGLLPLHSHNYASSSRSASASTGDYDRNHTYIVSSLLEEPYLSLKQYTYGESLVGNDRFEGYCKDLADMLAAQLGIKYEIRLVQDGNYGAENQYAPGGWDGMVGELIRKEADIAISAMTITAERERVIDFSKPFMTLGISIMIKKPVKQTPGVFSFLNPLSQEIWISVILSYVGVSFVLYFVTRFPPYEWRIVRRPQADSTAQQPPGIIGGATLSEPQAHVPPVPPNEFTMLNSFWYSLAAFMQQGCDITPPSIAGRIAAAVWWFFTIILISSYTANLAAFLTVERMVAPIKTPEDLTMQTDVNYGTLLYGSTWEFFRRSQIGLHNKMWEYMNANQHHSVHTYDEGIRRVRQSKGKYALLVESPKNEYVNARPPCDTMKVGRNIDTKGFGVATPIGSPLRKRLNEAVLTLKENGELLRIRNKWWFDKTECNLDQETSTPNELSLSNVAGIYYILIGGLLLAVIVAIMEFFCRNKTPQLKSPGSNGSAGGVPGMLASSTYQRDSLSDAIMHSQAKLAMQASSEYDERLVGVELASNVRYQYSM

>DmelGluR1B

MRFGLKLSCLWPSFLLWLTWSSGGGGGSGVGVSAQPSLTEKIPLGAIFEQGTDEVQSAFKYAMLNHNLNVSSRRFELQAYVDVINTADAFKLSRLICNQFSRGVYSMLGAVSPDSFDTLHSYSNTFQMPFVTPWFPEKVLTPSSGFLDFALSMRPDYHQAIIDTIQFYGWRKIIYLYDSHDGLLRLQQIYQGLRPGNESFQVELVKRISNVSMAIEFLHTLEQIGRFENKHIVLDCPTEMAKQILIQHVRDLRLGRRTYHYLLSGLVMDDRWESEIIEFGAINITGFRIVDTNRRLVREFYDSWKRLDPQMSVGAGRESISAQAALMYDAVFVLVEAFNKILRKKPDQFRNNVQRRSQTLMVAQAAASTSSDGYNYSASGGGGGNGGAGGGFAGSDSGGSGGMASRALDCNTAKGWVNAWEHGDKISRYLRKVEIEGLTGDIKFNDDGRRVNYTLHVVEMTVNSAMVKVAEWNDDAGLQPLNAKYVRLRPHVEFEKNRTYIVTTVLEEPYIMLKQVAFGEKLHGNNRFEGYCKDLADLLAKELGINYELRLVKDGNYGSEKSSAHGGWDGMVGELVRKEADIAIAAMTITAERERVIDFSKPFMSLGISIMIKKPVKQTPGVFSFMNPLSQEIWVSVIFSYIGVSIVLFFVSRFSPHEWRLVQQQPQQSQSPDPHAHHEQLANQQPPGIIGGAPLPAPPGPPTPGAQTAAGAAALQAALSAGSPGSGGSSSAVVNEFSVWNSFWFSLAAFMQQGCDLSPRSVSGRIAAASWFFFTLILISSYTANLAAFLTVERMVTPINSPEDLAMQTEVQYGTLLHGSTWDFFRRSQIGLHNKMWEYMNSRKHVFVPTYDEGIKRVRNSKGKYALLVESPKNEYVNAREPCDTMKVGRNLDTKGFGIATPLGSALKDPINLAVLTLKENGELIKLRNKWWYEKAECSTHKDGETSHSELSLSNVAGIFYILIGGLLVSVFVAILEYCFRSRDSRSASSGSGMGLGMGLGGGMSGGSLGKANGSMMLGPSSAVPGGMPSSHQRSTLTDTMHAKAKLTIQASRDYDNGRVGYLNCASLQYYPPAQLSATPPDAGDSLHMNAHGQV

>DmelGluRIIB

MHGLQFLVLLALAIASGANEDTLVIKIGAIFFDTEMKLADAFSAALEEVNAINPALKLDAIKRYVTVDDSIVLQDISCDLIGSGVAAIFGPSSKTNSDIVEVLCNMTGIPHLQFDWHPQQSNRERMNHQLTVNVAPMELFLSAAFSDILASKTFDWKSFTIAYERSSHLIRLQHILAWKQLHKAGIKMQEFERGDDYRILWKRINNAREKFVLLDCPSDILVDVINASIGYNMTGSFNHLFLTNLDTHLSGIDGFYSRDFTVAVAAVRIRTYVPPPVHDEIDVFDNSVDTRFSSLGSQLVYDSIVLFYNALLEISQRPGFYIPNFSCGRGFWQPGPRLVEQMKQITPKMVKPPFKTQRLQINADGQREDFNLEVYNPIIDRVTHIWNKEFQLVDFEKLRENSTQALKQKRLQNKEDFSQKPIRYTVATRVGKPYFSWREEPEGVHYEGNERFEGYAVDLIYMLAQECKFDFNFEPVRDNKYGSYDANTDEWDGIIRQLIDNNAQIGICDLTITQARRSVVDFTVPFMQLGISILSYKEPPPKADIYAFLNPYNAEVWLFVMIAMMITAFALIFTGRIDQYEWDQPVENVNREMERQNIWHLSNALWLVLGSMLNQGCDLLPRGLPMRLLTAFWWIFALLISQTYIAKLAAFITSSKIAGDIGSLHDLVDQNKVQFGTIRGGATSVYFSESNDTDNRMAWNKMLSFKPDAFTKNNEEGVDRVKLSKGTYAFLMETTNLQYYVQRNCELTQIGESFGEKHYGIAVPLNADFRSNLSVGILRLSERGELFKLRNKWFNSNESTCDSNVPTIDDGQFDMDSVGGLFVVLIVGVVVGLVIGVAEFLWHVQRISVKEKIPPMLALKAEFYFVIRFWLTRKPLHTYRQSRDSTSTGYSSLEQITSASSAKKKKKTRRIEK

>DmelGluRIIC

MWQRILLLGCMWSAFFMCRSRGQQINIGAFFYDDELELEKEFMTVVNAINGPESEQTMRFYPLIKRLKPEDGSVTMQEHACDLIDNGVAAIFGPSSKAASDIVALVCNSTGIPHIEFDISDEGIQAEKPNHQMTLNLYPAQAILSKAYADIVQNFGWRKFTIVYDADDARAAARLQDLLQLREVHNDVVRVRKFHKDDDFRVMWKSIRGERRVVLDCEPNMLVELLNSSTEFGLTGQYNHIFLTNLETYTDHLEELAADNETFAVNITAARLLVNPDPPPYSLPYGYVTQRDNIVYESSDPPRTLIHDLIHDALQLFAQSWRNASFFYPDRMVVPRITCDFAASGGRTWAMGRYLARLMKGTSGVNNTNFRTSILQFDEDGQRITFNIEVYDPLDGIGIAIWDPRGQITQLNVDVKAQKKMIYRVATRIGPPYFSYNETARELNLTGNALYQGYAVDLIDAIARHVGFEYVFVPVADQQYGKLDKETKQWNGIIGEIINNDAHMGICDLTITQARKTAVDFTVPFMQLGVSILAYKSPHVEKTLDAYLAPFGGEVWIWILISVFVMTFLKTIVARISKMDWENPHPCNRDPEVLENQWRIHNTGWLTVASIMTAGCDILPRSPQVRMFEATWWIFAIIIANSYTANLAAFLTSSKMEGSIANLKDLSAQKKVKFGTIYGGSTYNLLADSNETVYRLAFNLMNNDDPSAYTKDNLEGVDRVRKNRGDYMFLMETTTLEYHREQNCDLRSVGEKFGEKHYAIAVPFGAEYRSNLSVAILKLSERGELYDLKQKWWKNPNASCFEEPDPDATPDMTFEELRGIFYTLYAGILIAFLIGITEFLVYVQQVALEERLTFKDAFKKEIRFVLCVWNNRKPIVAGTPISSVRTTPRRSLDKSLDRTPKSSRRVVIGRSSEEMREMAQGSGSSSGSNNAGRGEKEARV

>DmelGluRIID

MHFCWISLIILSLSRVQAQFYGGNAYEASSGQSIRLGLITDDATDRIRQTFEHAISVVNNELGVPLVGETEQVAYGNSVQAFAQLCRLMQSGVGAVFGPAARHTASHLLNACDSKDIPFIYPHLSWGSNPDGFNLHPSPEDIANALYDIVNQFEWSRFIFCYESAEYLKILDHLMTRYGIKGPVIKVMRYDLNLNGNYKSVLRRIRKSEDSRIVVVGSTTGVAELLRQAQQVGIMNEDYTYIIGNLNLHTFDLEEYKYSEANITGIRMFSPDQEEVRDLMEKLHQELGESEPVNSGSTFITMEMALTYDAVRVIAETTKHLPYQPQMLNCSERHDNVQPDGSTFRNYMRSLEIKEKTITGRIYFEGNVRKGFTFDVIELQTSGLVKVGTWEEGKDFEFQRPPQAVNFNDIDDGSLVNKTFIVLISVATKPYASLVESIDTLIGNNQFQGYGVDLIKELADKLGFNFTFRDGGNDYGSFNKTTNSTSGMLKEIVEGRADLAITDLTITSEREEVIDFSIPFMNLGIAILYVKPQKAPPALFSFMDPFSSEVWLYLGIAYLGVSLCFFIIGRLSPIEWDNPYPCIEEPEELENQFTINNSLWFTTGALLQQGSEIAPKALSTRTISAIWWFFTLIMVSSYTANLAAFLTIENPTSPINSVKDLADNKDDVQYGAKRTGSTRNFFSTSEEPIYIKMNEYLNAHPEMLMENNQQGVDKVKSGTKYAFLMESTSIEFNTVRECNLTKVGDPLDEKGYGIAMVKNWPYRDKFNKALLELQEQGVLARLKNKWWNEVGAGVCSAKSDDDGPSELGVDNLSGIYVVLVIGSIISIIISILCWCYFVYKKAKNYEVPFCDALAEEFRIVIRFSENERPLKSAQSIYSRSRNSSQSIESLKTDSEENMPVED

>DmelGluRIIE

MFFNHFVILWSLFSIHISVNWAQYENFGGYDNYQSLESVPIGLLTDQNTEQMNIVFDHAIDVANQEVGTSLTSLKEEVNYGDAYQSYGKLCRMLETGIAGVFGPSSRHTAVHLMSICDAMDIPHIYSYMSENAEGFNLHPHPADLAKALYSLITEFNWTRFIFLYESAEYLNILNELTTMLGKSGTVITVLRYDMQLNGNYKQVLRRVRKSVDNRIVVVGSSETMPEFLNQAQQVGIINEDYKYIIGNLDFHSFDLEEYKYSEANITGLRLFSPEKMAVKELLMKLGYPTDQDEFRNGSCPITVEMALTYDAVQLFAQTLKNLPFKPMPQNCSQRTESVRDDGSSFKNYMRTLRLTDRLLTGPIYFEGNVRKGYHLDVIELQPSGIVKVGTWDEDRQYRPQRLAPTTAQFDSVDNSLANKTFIILLSVPNKPYAQLVETYKQLEGNSQYEGYGVDLIKELADKLGFNFTFVNGGNDYGSYNKSTNESTGMLREIMTGRADLAITDLTITSEREQALDFTIPFMNLGIAILYLKPQKATPELFTFMDPFSEEVWWFLGFSFLGVSLSFFILGRLSPSEWDNPYPCIEEPEELENQFTLGNSIWFTTGALLQQGSEIGPKALSTRTVASFWWFFTLIVVSSYTANLAAFLTIEKPQSLINSVDDLADNKDGVVYGAKKTGSTRNFFMTSAEERYKKMNKFMSENPQYLTEDNMEGVNRVKTNTHYAFLMESTSIEYNTKRECNLKKIGDALDEKGYGIAMRKDWPHRGKFNNALLELQEQGVLEKMKNKWWNEVGTGICATKEDAPDATPLDMNNLEGVFFVLLVGSCCALLYGIISWVLFVMKKAHHYRVPLRDALKEEFQFVIDFNNYVRVLKNSASIYSRSRQSSMSVASVAQESQ

>DmelNmdar.1

MAMAEFVFCRPLFGLAIVLLVAPIDAAQRHTASDNPSTYNIGGVLSNSDSEEHFSTTIKHLNFDQQYVPRKVTYYDKTIRMDKNPIKTVFNVCDKLIENRVYAVVVSHEQTSGDLSPAAVSYTSGFYSIPVIGISSRDAAFSDKNIHVSFLRTVPPYYHQADVWLEMLSHFAYTKVIIIHSSDTDGRAILGRFQTTSQTYYDDVDVRATVELIVEFEPKLESFTEHLIDMKTAQSRVYLMYASTEDAQVIFRDAGEYNMTGEGHVWIVTEQALFSNNTPDGVLGLQLEHAHSDKGHIRDSVYVLASAIKEMISNETIAEAPKDCGDSAVNWESGKRLFQYLKSRNITGETGQVAFDDNGDRIYAGYDVINIREQQKKHVVGKFSYDSMRAKMRMRINDSEIIWPGKQRRKPEGIMIPTHLRLLTIEEKPFVYVRRMGDDEFRCEPDERPCPLFNNSDATANEFCCRGYCIDLLIELSKRINFTYDLALSPDGQFGHYILRNNTGAMTLRKEWTGLIGELVNERADMIVAPLTINPERAEYIEFSKPFKYQGITILEKKPSRSSTLVSFLQPFSNTLWILVMVSVHVVALVLYLLDRFSPFGRFKLSHSDSNEEKALNLSSAVWFAWGVLLNSGIGEGTPRSFSARVLGMVWAGFAMIIVASYTANLAAFLVLERPKTKLSGINDARLRNTMENLTCATVKGSSVDMYFRRQVELSNMYRTMEANNYATAEQAIQDVKKGKLMAFIWDSSRLEYEASKDCELVTAGELFGRSGYGIGLQKGSPWTDAVTLAILEFHESGFMEKLDKQWIFHGHVQQNCELFEKTPNTLGLKNMAGVFILVGVGIAGGVGLIIIEVIYKKHQVKKQKRLDIARHAADKWRGTIEKRKTIRASLAMQRQYNVGLNSTHAPGTISLAVDKRRYPRLGQRLGPERAWPGDAADVLRIRRPYELGNPGQSPKVMAANQPGMPMPMLGKTRPQQSVLPPRYSPGYTSDVSHLVV

>DmelNmdar.2

MMNNEQFGHSTASAQYFLQLAGYLGIPVISWNADNSGLERRASQSTLQLQLAPSIEHQSAAMLSILERYKWHQFSVVTSQIAGHDDFVQAVRERVAEMQEHFKFTILNSIVVTRTSDLMELVNSEARVMLLYATQTEAITILRAAEEMKLTGENYVWVVSQSVIEKKDAHSQFPVGMLGVHFDTSSAALMNEISNAIKIYSYGVEAYLTDPANRDRRLTTQSLSCEDEGRGRWDNGEIFFKYLRNVSIEGDLNKPNIEFTADGDLRSAELKIMNLRPSANNKNLVWEEIGVWKSWETQKLDIRDIAWPGNSHAPPQGVPEKFHLKITFLEEAPYINLSPADPVSGKCLMDRGVLCRVAADHEMAADIDVGQAHRNESFYQCCSGFCIDLLEKFAEELGFTYELVRVEDGKWGTLENGKWNGLIADLVNRKTDMVLTSLMINTEREAVVDFSEPFMETGIAIVVAKRTGIISPTAFLEPFDTASWMLVGIVAIQAATFMIFLFEWLSPSGYDMKLYLQNTNVTPYRFSLFRTYWLVWAVLFQAAVHVDSPRGFTSRFMTNVWALFAVVFLAIYTANLAAFMITREEFHEFSGLNDSRLVHPFSHKPSFKFGTIPYSHTDSTIHKYFNVMHNYMRQYNKTSVADGVAAVLNGNLDSFIYDGTVLDYLVAQDEDCRLMTVGSWYAMTGYGLAFSRNSKYVQMFNKRLLEFRANGDLERLRRYWMTGTCRPGKQEHKSSDPLALEQFLSAFLLLMAGILLAALLLLLEHVYFKYIRKRLAKKDGGHCCALISLSMGKSLTFRGAVFEATEILKKHRCNDPICDTHLWKVKHELDMSRLRVRQLEKVMDKHGIKAPQLRLASSSDLLNHHHLKERPPLLGNLSLAASAQDLYRWSYKTEIAEMETVL

**SNMP**

>OchiSNMP1

MQLPVGLAAGGGGVFFLAVVAGWYGMPKLISSQISSGLALKKGSDIRQMWANFSDPIDFRVYMLNLTNPEAVHRGEKPVVQQVGPYFYEEYKQKVKLRDHKEDDTVSYNNKITWLFNQGKSAPGLTGDEIITMPHPLLLGMLLALERDKPGMLALVNKAIPPLFRKPESIFVTAPARNFLFDGIVINCTVTDFSAKALCTGLKKEAKELKREGDNFFFSFFGHKNGTVDAGRLRVKRGIQNIDDLGRVISFNGEPKMSSWRGDPCNDLRGTDSTIFPPFRDPKEPIIAFGADLCLSLGATWERKAEYMGVPGNRYTAEMPDMKGNPEHHCYCPTEQTCLEKGALDLSPCAGAPVIATLPHFYLASETYLRTVSGLHPTKEEHELFMVFESTTGSPMEARKRLQFNMFLHKINKIDLLANVPDALMPLLWVEEGLALEEKYISTLRMLFRMQGIMSGVKWALMMVGMGMAGAGGYLHFKRRKELVVGPAEPKKVAAGHNDTPGHPIRLESSHSRY

>OchiSNMP2

MLSAGVCGCGRRGLLWALVAGAAVLALALALRWAAFPAILNAKISQAVKLQDGTPAMERFVEIPQPLLYKTYLFNVTNPDEISQGGKPVLQQVGPYVYEERRRRHDVRRQSDGSLAYRLETSFYFSPERSPGLSEDDEFTYLNVVMLGVVVQVSEDYSSLLSMVEPVLRELLPGGSQLFRVATARQLLWSGVPTVDCRGNLSALAALACGALPPLLPPTVRQAEPGLYLFSFFGFKNGTSRQWWRVDSGERDVRALGSVLSYDNSSRLKTWSPSDSSCNEIRGTDSTLFPPFITPNDTLYVFAHDICRSMHVEYEREQEISGLKGLRFVASRDMLRRGGPNACTCAGGRCLPSGAMSVRECLKAPIAVSFPHFYQAAPEYLQYAEGLSPSKDHHETFVVVEPESGTPLLGAKRLQFNMRAARVEQIPSLRSVPEGLFPLLWVDEGVELEEPQLSQVRALYVARASMGGVAWGVLAVGVAALLLSAFLLVRARLRERGRSLSLEKGAAVGGKLSVPTLGAAYPESTPRRASPPATTPAAPVDATRF

>SgreSNMP1

MQLPVGLAAGGGGVFFMAVVAGWYGMPKLISSQIASGLALKKGSDIRQMWSNFSDPIDFRVYVLNLTNPEAVHRGEKPIVQEIGPYFYEEYKQKVKLRDHKEDDTVSYNNKITWLFNQGKSAPGLTGDELVTLPHPLLLGLLLTLERDKPGMLALVNKAIPPLFRKPESIFVTAPVRNFLFDGIVINCTVTDFSAKALCTGLKKEAKELKREGDNFFFSFFGHKNGTVDAGRLRVKRGIQNIDDLGRVVAFNGEPKMSAWRGDPCNDLRGTDSTIFPPFRDPKEPIVAFGPDLCLSLGANWERKAEYMGVPGNRYTAELPDMKGNPEHHCYCPTEQTCLEKGTLDLSPCAGAPVIATLPHFYLASETYLQTVSGLQPTKENHELFMVFESTTGSPMEARKRLQFNMFLHKINKIDLLANVPYALMPLIWVEEGLALEEKYVSTLRMLFRMQGIMSGVKWTLMAVGMGMAGAGGYLHFKRRKELVVGPAEPKKVVAGHDTTGHPIRLESSHSRY

>SgreSNMP2

MLSARVCGCGRRGLWWGLAAGAALLAVALVLRWAAFPAILTAKIKQAVQLHDGSPAMERFVQLPQPLLYKVYLFNVTNPDEVEQGAKPVLQQVGPYVYEEWRRRRDVTRMANGSLDYRLETTYHFSPERSPGLSEDDEFTYLNVVMVGIVVQVSEDYSSLLSMVEPVLSELVPGGAQLFQRASARQLLWSGVPTVDCRGNLSAVATLACGALPSLLPATVQQTEPGVYVFSFFGFKNGTSKQWWRVDSGVEDVRTLGSVISYDNSSRLKVWSPSNSPCNEIRGTDSTLFPPFITPNDTIYIFAHDICRSMHAEYEREQDVSGVHGLRFVASGSLLRRGGPNACTCPDGRCLATGAISVRECFRAPIAVSFPHFYQASPEYLQYAEGLSPNKELHETFVVIEPETGTPLVGAKRLQFNMKAVRVSQVPALRNVSDGLFPLLWVEEGVELEEKQLSQVRALYVARASMGGVAWAVLAVGVAALLFCAVRLAKARVAERNRSLSLEKGVTAGGKLSVPTLGAAYPESATKRPSPPAAAPAASTAPAAPVDATHF

>OasiSNMP1

MQLPVGLAAGGGGVFFLAVVAGWYGMPKLIHSQIANGLALKKGSDIRQMWSNFSDPIDFRVYILNLTNPEAVHRGEKPIVQEIGPYFYEEYKQKVKLRDHKEDDTVSYNNKVTWLFNRGKSAPGLTGDEMVTMPHPLLLGLLLTLERDKPGMLALVNKAIPP

>OasiSNMP2a

MLSARVCGCGRRGLWWGLAAGAVVLAAALVLRWAAFPAILTAKITQAVQIQDGSAAMERFVQLPQPLLYKAYLFNVTNPDQVSLGAKPALQEVGPYVYEEWRRRRDVTRLEDGSLSYRLETTFRFSPERSPGLSEDDEFTYLNVVMMGIVVQVAEDYASMLTMVEPVLSELLSGGAQLFLRATARQLLWDGVPTVDCRGNLSAVAVLACSALPSLLPPTVQQAQPGVYAFSFFGFKNGTARQWWRVDPGLSDVRSLGAVLSYDNSSRLKVWTPASSPCNDIRGTDSTIFPPFRDPKEPIVAFGADLCLSLGATWERSAEYMGVPGNRYTGEMPDMTGNPEHHCYCPSETSCLEKGALDLSPCAGAPVIATLPHFYLSSESYLRTVSGLQPTKEHHELFMVFESTTGSPMEARKRLQFNMFLHKINKIDLLANVPYALMPLIWVEEGLALEEKYVSTLRMLFKMQGIMSGVKWTMMVVGMGMAGAGGYLHFKRRKELVVGPAEPKKVAAGHAEPPGHPIRLESSHSRY

>OasiSNMP2b

MLSARVCGCGRRGLWWGLAAGAVVLAAALVLRWAAFPAILTAKITQAVQIQDGSAAMERFVQLPQPLLYKAYLFNVTNPDQVSLGAKPALQEVGPYVYEEWRRRRDVTRLEDGSLSYRLETTFRFSPERSPGVSEDDEFTYLNVVMMGIVVQVAEDYASMLTMVEPVLSELLSGGAQLFLRATARQLLWDGVPTVDCRGNLSAVAVLACSALPSLLPPTVQQAQPGVYAFSFFGFKNGTARQWWRVDPGLSDVRSLGAVLSYDNSSRLKVWTPASSPCNDIRGTDSTIFPPFITPNHTIHIFAHDICRSMHADFEREQEVSGVRGLRFVASRSLLRQGGRNACTCPEGHCLPSGAISVKECFRAPIAVSYPHFYQAEPEYLQYAEGLSPSKELHETFVVIEPESGTPLLGAKRLQFNMRAARVPQVPALANLTDGLFPLLWVEEGVELEEAELSQVRALFVARASLGGVAWAVLAGGVAALLFCAYRLARGRLRERNTTLALDKGAAAGGKLSVPTLGAAYPESAPRRASPPPAAPVDATNF

>DmelSNMP1

MQVPRVKLLMGSGAMFVFAIIYGWVIFPKILKFMISKQVTLKPGSDVRELWSNTPFPLHFYIYVFNVTNPDEVSEGAKPRLQEVGPFVFDEWKDKYDLEDDVVEDTVSFTMRNTFIFNPKESLPLTGEEEIILPHPIMLPGGISVQREKAAMMELVSKGLSIVFPDAKAFLKAKFMDLFFRGINVDCSSEEFSAKALCTVFYTGEIKQAKQVNQTHFLFSFMGQANHSDSGRFTVCRGVKNNKKLGKVVKFADEPEQDIWPDGECNTFVGTDSTVFAPGLKKEDGLWAFTPDLCRSLGAYYQHKSSYHGMPSMRYTLDLGDIRADEKLHCFCEDPEDLDTCPPKGTMNLAACVGGPLMASMPHFYLGDPKLVADVDGLNPNEKDHAVYIDFELMSGTPFQAAKRLQFNLDMEPVEGIEPMKNLPKLILPMFWVEEGVQLNKTYTNLVKYTLFLGLKINSVLRWSLITFSLVGLMFSAYLFYHKSDSLDINSILKDNNKVDDVASTKEPLPSANPKQSSTVHPVQLPNTLIPGTNPATNPATHHKMEHRERY

>DmelSNMP2

MIHWSLIVSALGVCVAVLGGYCGWILFPNMVHKKVEQSVVIQDGSEQFKRFVNLPQPLNFKVYIFNVTNSDRIQQGAIPIVEEIGPYVYKQFRQKKVKHFSRDGSKISYVQNVHFDFDAVASAPYTQDDRIVALNMHMNAFLQVFEREITDIFQGFANRLNSRLNQTPGVRVLKRLMERIRGKRKSVLQISENDPGLALLLVHLNANLKAVFNDPRSMFVSTSVREYLFDGVRFCINPQGIAKAICNQIKESGSKTIREKSDGSLAFSFFGHKNGSGHEVYEVHTGKGDPMRVLEIQKLDDSHNLQVWLNASSEGETSVCNQINGTDASAYPPFRQRGDSMYIFSADICRSVQLFYQTDIQYQGIPGYRYSIGENFINDIGPEHDNECFCVDKLANVIKRKNGCLYAGALDLTTCLDAPVILTLPHMLGASNEYRKMIRGLKPDAKKHQTFVDVQSLTGTPLQGGKRVQFNMFLKSINRIGITENLPTVLMPAIWVEEGIQLNGEMVAFFKKKLINTLKTLNIVHWATLCGGIGVAVACLIYYIYQRGRVVEPPVK

>AaegSNMP2

MMVMNTELRQDTPQFKRWEAVPQPLDFKVYIFNVTNPYEVQMGRRPRVVEVGPYVYFQYCHKDNIRFSRDRSKVHFSQQQMYVFDAESSYPLTENDQLTVLNMHMNSILQIIDTQAKETITNFRSDVNNTLEKIPVVRVIKRIIEKTTPIQSILQLAEDETYDSLRLINAELNRIFGRPDSMFLRTTPREFLFEGVPFCVNVIGIAKAICKEIEKRNTKTIRVQPDGSMKFSFFNHKNMTNDGTYTINTGIKEPALTQMIEYWNGRNTLDRWINQSAGSSSKCNKIVGTDGSGYPPFREGVERMTIFSSDICRTVDIKYVGPSSYEGIPALRFETDSHFLNEIGPEYGNDCYCVNRIPKAIVKNNGCLYKGALDLSTCFDAPVVLTHPHMMGAAQEYTSLIDGLYPDPEKHQIFVDVEPLTGTPLNGGKRVQFNMFLRRIDSIRLTDRLQTTLFPVLWIEEGIALNEDMVKLIDDSLMKVLTLLDIVQWVMIGSGLLLAIIMPIVYFIKRKPSSGSITPTLTTTTSTVSISDGGGLGGNPQK

>AgamSNMP1

MELKERNFKKIGLICVAVLLCGMVFSYGIFPSILRFMIKQNVLLKPGTQIRDMFEKIPFPLDFKLHIFNVTNPDEIMRGGKPRVNDIGPLYFEEWKEKYDTVDNVEEDTLTFTLRNTWIFRPDLSALTGEEIVTIPHPLIMGVLLMVQRDREAMMPLVKKGVNILFDPLESAFLKVRIMDLLFDGIYVDCSSQDFAAKALCSGMDSEGAVMPHNETHYKFSFFGMRNHTEAGRWVVYRGVKNIRDLGRVVSYNEETEMDIWDGDECNQYIGTDSTIFPPFLTAQDRLWAWSPEICRSLGAHYVHKSKYAGLPMSYFELDFGDLKNEPHNHCFCRDAPDDCPPKGTMDLSPCLGGPIIGSKPHFYGADPKLVEAVDGLAPNKAAHDVYIHFELASICWVSPVSAAKRLQFSMELGPIRDHELFGQLPDVILPMFWAEEGASLNKTWTNQLKYQLFLGLKFNATVKWLTIIIGTVGAVGSAYMYFRKETKTTDVAPVDVSTPDTNPSSAKDGVVNVSLGRNLPPVIDGLDKPPKLRATELQQERY

>AgamSNMP2

ATELRQGTDQYKRWEALPQPLDFKVYIFNVTNPYEVMQGRRPKVVEVGPYVYFQYRQKDNVRFSRDRSKVHFSQQQMYVFDAESSYPLTENDELTVLNMHMNSILQIIDNQAKETITNFRSDVNNTLEKIPVVRVIKRIIERTTPIQSILQIAEDETYDSLRLINVELNRIFGRPDTMFLRTTPKQFLFDGVPFCVNVIGIAKAICKEIEKRNTKTIRTMPDGSLRFSFFSHKNMTDDGMFTINTGIKDPSRTQMIELWNGRTTLDVWNNRSSGLSSSCNKIHGTDGSGYPPFRTGVERMTIFSTDICRTVDIKLTGSSSYEGIPALRYEIDNNFLHEIGPEYGNDCYCVNKIPKSIVKSNGCLYKGALDLSNCFDAPVVLTLPHMLGVAEEYTALIDGMDPEPERHQIFVDVEPYTGTPLNGGKRVQFNMFLRRIDAIKLTDRLQPTLFPVIWIDEGIALNEDMVKLIDDSLMKVLSLLDVVQWVLIGVGLLLAVLMPTVYFVKRCRGEGSRTVSPAVTATTSAASLSTVAGVTGDRSK

>CpipSNMP1a

MVVGLTVSMAILPELVNLMLRQNLRLKPGSDLRKMYEKVPFGLDFKVHIFNITNPQEIMQGGRPRVKDIGPFYFEEWKEKYDIEDNDGEDTMTFDMKNTWIFRPDLTAPLTGNEMITVPYLLVIGVLLAIQRDKEAMLPLISKGLDIIFEPLESAFVTVRVMDLLFDGIPVDCSSEEFAAKALCSGLDSEGAVAPLNDTHVKFSMFGLRNGTSIGRFKVYRGIKNVADLGRVITYNDETEMDFYDGDECNKYVGTDSTIFPPFLTKKDRLWAWSPEICQSLGAVYAGKSSYQGFPTSFFTIDFGDLRDDPVHQCYCRDPPDGCPPKGTIDLGPCVGAPILGSKPHFIGGDPKLLRDVDGLEPDPKEHDIFIHYDLQTGTPFSAAKRLQFNLELEPIRGHEVFGKLPKMVLPMFWAEEGASLNKTWTKQLKPLFMIRKFNATVKWLSIVLGTLGTIGAGFMHYKLHIKPVNVRPMEVQKTTVKEVEPSVETNGTGKEPPEKIEPRVVESAHRNLPPLFDGGLAGKQKPVPSDQRER

>CpipSNMP1b

MKLEELNFKKIAIICACTLVGGLTFCYGIFPPILKFMLKQNVLLKPGTQMRGMFEKMPFPLDFKIHLFNVSNPEEIMKGGKPKIKDVGPYYFEEWKEKFDTEDDLEEDTLSFTLRNTWKFRPDLSSPLTGDEMITLPNMLLMGVFLMVQREREAMMPLIRKGAKIIFDPLESAFMTVRVMDFLFDGLPVDCSSQDFASKALCSGMESEKVVLPLNDTHYQFSIFGGRNATDAGRWVVYRGVKNIKDLGRIVSFNGETEMDTYDGDECNQFVGTDSTIFPPFLTKEDRLWAWSPEICRSMGATYGGKSKYAGMPMSYFELDFGDLKNEPENHCFCRDPPEDCPPKGTMDLAPCLGAPLLGSKPHFIDADPKLLEEVQGLEPNREDHDMFINFELISGTPVSAAKRLQFNLEMEPVRDHEVLGNLPNVILPVFWVQEGVSLNKTWTNQLKYQLFLGLKFNATVKWLTIIIGTVGSIGAGIMHYKRSTKSVNVTPVEAVSNGSGRIISVSSAGKDREAINNSKNLPAVLDGLGERIPKMAPVEQRY

>CpipSNMP1c

NVLLKPGTQMRWMFEKIPFPLDFKIHLFNVTNPDEVMKGGKPKIRDVGPYYFEEWKEKFDTEDDLEEDTLSFTLRNTWIFRPDISAPLTGDEMITVPHLLVLGVFLSVQRDREEMMPLISSGMKIIFDPLESAFMTVRVMDLLFDGIPVDCGSEEFAAKAVCSGMESEGAVAPLNETHVKFSMFGMRNATDAGRWVVYRGVKNIRDLGRIVSYNGEPEMDIYDGDECNQYIGTDSTIFPPFLTKQDRLWAWAPEICRSLGAHYIGKSKYAGMPMSLFKLDFGDLKNEPENHCFCRDPPEDCPPKGTMDLSMCIGVPILGSKPHLLDADPKLLEGVDGLEPNEAEHDVFIHFELLSGTPVSGAKKLQLNLEVEPIRDHEVLGNLPTVVLPMIWVEEGVSLNKTWTNQLKYQLFLGLKFNATVKWLTIIVGTLGSIGAGFMHYKRTSQVTQVEQVAAGAKSEGGGRFISVSAATTEEGKNGGGGNLPAVLDGLDPSGISKRMSDPAQKERY

>CpipSNMP2

MKARNLNPPAPSQGGKLIKGKVLCSSPWVKAKAGCGAARLAKDNTELRQGTPQFKRFEALPQPLDFKVFIFNVTNPYEVQMGKRPRVVEVGPYIYFQYRQKDNIRFSRDRSKVHFSQQQLYVFDAESSYPLTENDPLMVLNMHMNSILQIIDNQAKETITNFRSDVNNTLEKIPIVRVIKRIIEKTTPIQSILQIAEDETYDSLRLINVELNRIFGRPDSMFLRTTPKEFLFDGVPFCVNVIGIAKAICKEIEKRNTKTIRVLPDGSMKFSFFNHKNMTEDGIYTINTGVKNALETQMIEFWNGKNMLDKWSNSSRGSSMTCNKIEGTDGSGYPPFREGVQRMTIFSSDICRTVDIKYVGSSSYEGIPAARYVTDDNFLNKIGPEHNNDCYCVNRIPKAIVKANGCLYEGALDLSTCFDAPVVLTLPHMMGAAEEYTSLIDGMHPDPEKHQIFVDVEPLTGTPLNGGKRVQFNMFLRRIDSIRLTDRLPTTLFPVLWIEEGIALNEDMVKLIDDSLMKILTILDIVQWTMIAIGLFLAISMPILYFTKRRPSSGTITPTLTTTTSAASIPERGGLGGNPDK

>BmorSNMP1

MQLAKPLKYAAISGIVAFVGLMFGWVIFPAILKSQLKKEMALSKKTDVRKMWEKIPFALDFKIYLFNYTNAEDVQKGAVPIVKEVGPFYFEEWKEKVEVEENEGNDTINYKKIDVFLFKPELSGPGLTGEEVIVMPNIFMMAMALTVYREKPAMLNVAAKAINGIFDSPSDVFMRVKALDILFRGIIINCDRTEFAPKAACTTIKKEAPNGIVFEPNNQLRFSLFGVRNNSVDPHVVTVKRGVQNVMDVGRVVAIDGKTKMNVWRDSCNEYQGTDGTVFPPFLTHKDRLQSFSGDLCRSFKPWFQKKTSYNGIKTNRYVANIGDFANDPELQCYCDSPDKCPPKGLMDLYKCIKAPMFVSMPHYLEGDPELLKNVKGLNPNAKEHGIEIDFEPISGTPMVAKQRIQFNIQLLKSEKMDLLKDLPGTIVPLFWIEEGLSLNKTFVKMLKSQLFIPKRVVSVVCWCMISFGSLGVIAAVIFHFKGDIMHLAVAGDNSVSKIKPENDENKEVGVMGQNQEPAKVM

>BmorSNMP2

MLAKYTKTIFSVSVAFLVVSIVLATWGFPKIIRKQIQKNVQISNTSKMYDKWVKLPMPLDFKIYVFNVTNRDAINQGEKPNLKEIGPYVYKQYREKIILGYGDNDTIKYNLKKTFVFDPVASGDLREDDELTVINFSYMAAIISVQEMMPAAVGMINRALEQFFTNLTDPFQTVKVKDLFFDGLFLNCEGDNTALGLICGKIRAEKPPTMRISKSANGFYFSMFSHMNRTVSGPYEMVRGTENLSDLGHVISYQGKRIMSAWDDQYCGQLNGTDSTIFPPLEDGNIPEKLYTFEPDICRSLFASLVGKDTLFNISTYYYEISDMTLGSKSANPDNKCFCKRNGSVKHDGCLLMGVLNLAPCQGAPAIASLPHFYLGSDELADFFGDGIKPDKEKHNTYVHLDPITGVVIKGVKRLQFNIELRNVPSVPQLKEVPSGLFPLLWIEEGAEIPEWLRKEIMDSHTMLWYVDAARWLVLAVAVVAVLVSATLVARSAALIPWPRNSNSISFILGNSVNTSKVHS

>HvirSNMP1

MQLPKELKYAAIAGGVALFGLIFGWVLFPTILKSQLKKEMALSKKTDVRKMWEKIPFALDFKVYIFNFTNAEEVQKGATPILKEIGPYHFDEWKEKVEVEDHEEDDTITYKKRDVFYFNPEMSGPGLTGEEIVVIPHIFMLGMALTVARDKPAMLNMVGKAMNGIFDDPPDIFLRVKALDILFRGMIINCARTEFAPKATCTALKKEAVSGLVLEPNNQFRFSIFGTRNNTIDPHVITVKRGIKNVMDVGQVVAVDGKLEQTIWRDTCNEYQGTDGTVFPPFVPETERIQSFSTDLCRTFKPWYQKKTSYRGIKTNRYVANIGDFANDPELNCFCPKPDSCPPKGLMDLAPCMKAPMYASMPHFLDSDPELLTKVKGLNPDVTQHGIEIDYEPITGTPMVAKQRIQFNIQLLKTDKLDLFKDLSGDIVPLFWIDEGLALNKTFVNMLKHQLFIPKRVVGVLRWWVVSFGSLGAVIGIVFHFRDHIMRLAVSGDTKVSKVTPEEPEQKDISVIGQAQEPAKVNI

>HvirSNMP2

MLGKHSKIFFGVSLIFLVIAIVLASWGFQKIVNKQIQKNVQLANDSKMFERWVKLPMPLDFKVYVFNVTNVEEVNQGGKPILQEIGPYVYKQYREKTILGYGDNDTIKYMLKKHFEFDPEASGSLTEDDELTVVHFSYLAALLTVHDMMPSLVTVVNKALEQLFPSLEDAFLRVKVRDLFFDGIYLSCDGDNSALGLVCGKIRAEMPPTMRKAEGSNGFYFSMFSHMNRSESGPYEMIRGRDNVYELGNIVSYKGQENMPMWGDKYCGQINGSDSSIFPPIKEDDVPKKIYTFEPDICRSVYADLVDKRELFNISTYYYEISETAFAAKSANPNNRCFCKKNWSANHDGCLLMGLLNLTPCQGAPAIASLPHFYLGSEELLDYFQSGVQPDKEKHNTYVYIDPVTGVVLSGVKRLQFNIEMRQINNIPQLKSVPTGLFPMLWLEEGATIPESIQQELRDSHKLLGYVEVAKWFLLTIAIISVIASAVAVARANALLSWPRNSNSVSFILGPSVTQVNKGN

>MsexSNMP1

MRLARGIKYAVIGAGVALFGVLFGWVMFPAILKSQLKKEMALSKKTDVRKMWEKIPFALDFKIYLFNYTNPEEVQKGAAPIVKEVGPYYFEEWKEKVEIEDHEEDDTITYRKMDTFYFRPELSGPGLTGEETIIMPHVFMMSMAITVYRDKPSMMNMLGKAINGIFDNPSDVFMRVNAMDILFRGVIINCDRTEFAPKAACTAIKKEGAKSLIIEPNNQLRFSLFGLKNHTVDSRVVTVKRGIKNVMDVGQVVAMDGAPQLEIWNDHCNEYQGTDGTIFPPFLTQKDRLQSYSADLCRSFKPWFQKTTYYRGIKTNHYIANMGDFANDPELNCFCETPEKCPPKGLMDLTKCVKAPMYASMPHFLDADPQMLENVKGLNPDMNEHGIQIDFEPISGTPMMAKQRVQFNMELLRVEKIEIMKELPGYIVPLLWIEGGLALNKTFVKMLKNQLFIPKRIVSVIRWWLLSFGMLAALGGVIFHFKDDIMRIAIKGDSSVTKVNPEDGEQKDVSVIGQSHEPPKINM

>MsexSNMP2

MLAKHSKLFFTGSVVFLIVAIVLASWGFPKIISTRIQKSIQLENSSMMYDKWVKLPIPLIFKVYFFNVTNAEGINEGERPILQEIGPYVYKQYRERTVLGYGPNDTIKYMLKKNFVFDPEASNGLTEDDDVTVINFPYMAALLTIQQMMPSAVAMVNRALEQFFSNLTDPFMRVKVKDLLFDGVFLNCDGDSPALSLVCAKLKADSPPTMRPAEDGVNGYYFSMFSHLNRTETGPYEMVRGTEDVFALGNIVSYKEKKSVSAWGDEYCNRINGSDASIFPPIDENNVPERLYTFEPEICRSLYASLAGKATLFNISTYYYEISSSALASKSANPDNKCYCKKDWSASHDGCLLMGVFNLMPCQGAPAIASLPHFYLASEELLEYFEDGVKPDKEKHNTYVYIDPVTGVVLKGVKRLQFNIELRNMPRVPQLQAVPTGLFPMLWIEEGAVMTPDLQQELRDAHALLSYAQLARWIILAAAIILAIIATITVARSTSLISWPRNSNSVNFIIGPMVNDKMR

>TcasSNMP2

MGCSCCTIKVLLVCVVISVALLIVSLALAFKVFPDLLESEVNKAVRLEDGTKQYDRFVELPFPVDFKVYLFNVSNPQQVLDGTEKPKLEEIGPFVYKQYRKKTILGKNEEEDTISYTQKETFEFDAEASKPLTEESVVTVLNPALMSIYQLAEDLHLAGAADTCIKQTFENNQGKVFIEANVRKLLFDGFSFCKNTSPGICGLVNDLICAIAATKRNSDLVLPDYSLIFSYLNYKRKPDDGKYTVKRGLTNIEKLGHIVAWNDSLYTKFWGEGTTCSEVKGTDSTLYPPRVTTDSAFYIYSTDICRFVKINYKGEESYKGIDGYLFETSEDTLRSSAPEEDCYCSKLSRDMEGKKSCFLDGVIDMQTCFGVPVLFSFPHFLWADNKYLSAVEGLNPVEEKHKTYLVVEPNTGTPLKGMKRIQLNGVIRPIVGIKSMLQTKRALLPLLWIEEGVSLPQKYVDELKSSYFDKVQIVDGVRYALIVISAILVGAFGIIILRKRSHAKHHV

>TcasSNMP1a

MRLPVKIAIGCAIGLVVIIVFGFIAFPKMIKGKVKSMINLNKGSEIRQMFVKVPFALDFKIYMFNVTNPMDVQKGALPVLKEVGPFCFEEWKEKVDLDDNDDEDVMFYNPKDTFYKANGPGCLDGSQMITMAHPLILGMVNTVVRTKPGAISLISKAINSIYGNPDSIFMTASAMDILFDGVVIKCGVKDFAGKAVCSQLKEAPDLRHVDENDLAFSFIGPKNATPGKRFKVLRGVKESHDVGRILEYDNKKEMEVWPTKECNQYKGTDGTVFPPYLTKEEGLASYAPDLCRSLVAVYSGDTKYDGIPVRIYTATLGDMSKNADEKCYCPTPDTCLKKGMMDLFKCAGVPVYVSLPHFYESDESYVKGVVGLNPNKKDHGIQILFESTTGGPVKAAKRLQFNMPLEPNPKLPIFANLPNTVLPLFWVEEGVALNNTFTKPLKDLFKIMKIVKIAKWLIMLGCLGGLGAAGYLYFSKKGEANITPVHKVKPAENGVSTLGGEVNHAMSDNEIEKY

>TcasSNMP1b

MVKWQRQLKPGNEVRDFYIKLPIPLDFRVYFFNISNPEEVKQGEKPILKQIGPYCYDAYKEKINVEDDKDNDTLTYNPYDTYFFNQMRTGDLSQDDYVTILHPLTVGIVNAVATQKPQYLSAVNKALPVIFKENSSIYLTAKVREILFDGVLINCNVKDFSANAVCSQFKGQPAMVEVEKNIYSFSLLGSRNGSIPTRITIHRGVKNAADIGRVVTIDNKTDLDVWPEPECNAFRGTDGWVFPSFLEKEDGIWTVASDLCRSFKAQYVEDLKFHGVVVRKYFADLGDMSSNPAEKCFCPAPEKCLPKGVMDLTKCMKVPLYCTLPHFLRADEKLLQQVEGLSPELERHIIKIYFEPLTGTPMLGQRRIQFNLQLMPIPKVAMMKTVPEALHPILWIEEGVELEGFLLKKVTSVFTLLKLMTFVRYIMLGLSIQGILYGGYKLYQESKSKKVSPVQNGTTESKNHNQGKTGGIELPSMNKRNKENTKNA

>TcasSNMP1c

MSYKKITIISACCVVTIIGVAYIYAIRDISHRRNVRYKYIDRVNNVSNDVNGGVVSVGYCYDYKRIDVDNADSTYTYDIYNRSGNSDDYVTIIHVVSVNYVSVKTHYNDAGKSITAKVRDIDGMINCTSRDTAMAVCTIRTKIGISKDYKYAGNGTTRITVRGIKSNGKVAVDNVTKSDWSNCNYKGTDGWISGRKTIWMHATTCNIHADVGATSNGAVNKYYSDNICTNCSCGIDVTKCTAIYISHRSDSIRGVKGNDTSHITRIGTSMAIRNVVKKITIMNVSVIHVWVMGVVNGWRMIKTYTAVMKYISVASGTAYGGYHYKNKKYSKNIVSSK

>TcasSNMP1

MTSTARRRNIMKKVYKIMDRVYNITNSVNGVVKVGYCYDAKKIDVNGDSTYTYTYNDKSGRTADDYVTVHIVGIVNTVSRDSIVDRAIKSIKDNIYITTKVRDDGMTINCKVDSATAVCTKAIGIIKNVYKSIGRNGTNRYKVRGMKKWHGRVVNHKSTVWSTKKCNRRGTDGWIIDKVGWTYSSDCRNMHVVTSHGVAKYYADGDMSSNDKCYCKTCKGMMDTRCMGVIYATHRVDKVRRTVRGKITDHIVRVIIGTAKRMNIVKKISMKTAHIWIAIVGKMIKVVVAKVDVVKYCAVCAVAGSYCYKRKKKAVTVSKTAKA

>DponSNMP1

MMLSNKIWSSSRFLYGSVILLVSSVLLKLWLFESMVKFVIRDQTALRKRNQVREVYLKIPFPLNFKLYFFNVTNPEEIQTGSKPKLKEVGPFWYDEIKEKVQIIDNDTEDSLTYTPYDLFEYNQNKSNQLREDDYVTIIHPAIVGMVNLVLRDSPVFLSIVSKAIPSIFNNPQTIFLTAKVKDILFDGVELNCLGKDFGTTAVCSQMKSQIPGLKFKKDNENIFLFSLLGSRNGTLTRRLKVHRGIAHAKDLGRLVELDGKKEINIWRQAECNRFHGTDGWIFPALSTPEEGLPSFSTDLCRSVNLRYINDTVLKKIPVRIYETDLGDQMTDENEKCYCRSADSCLKKGVFDLSKCMGVPIYATLPHFLRTDPSYINLVDGLAPSELLHAIRVYFEPMTGTPLFAAKRMQFNLDLKPTNKIPLFSHLPTALFPMFWLEESVDLDGYLLKKVQTVFLLLHAVDIIQYLMIVIGCGCVTISMYFRLKNRKSVTITPATGSKKSAPPKPIDEMDVSHLSIAGILGDRPQKKAVVSQVMSGHEFDKY

>DponSNMP1a

MNFPMRLAIGSACSLLFIILVGFVGFPKMIKGKVKDMVNLKPGMEIREMFVKVPFPLSFNVYIFSVLNPAEVQGGAKPHLKEMGPFCYNEWKTKINVEDNEGDDTISYDPVDTFENAKRPKCLSVDTLVTIPHPMILGMVNTILRQKPGALTLANKAIKSIWSNPSSLFITVKAQDLLFDGVVIHCGVSDFAGKAICTNLKAEPSLTHLGEDDLGFSLMGPKNGTAGKRIKAFRGTQDFHKVGRIIEFDGKSKLDVWNNSKCDTIVGTDGTIFPPMLKKEEGLASFAPDLCRSLIAQFDKHDKYDGIPVSSFFASLGDQSKNPAEKCFCTTPETCLKRGLMDLYRCAKIPLYVSLPHFYDSHESYLKGVKGLKPDVEKHGIRIMFELLTGSPLSARKRLQFNMPLEPNPKVELFHNFTPTVLPIFWVEEAVDLNSTFTKPLKTLFLTKKLVNIVKYLVLLMSIGGFCAAVYLYFKSDDSMNVTSVQKVQPDQNGHRNIISTVFNGNHTAGQDNEAYEDKY

>DponSNMP2

MFRNCCSPRLVFLYNLLAVLLLIASLVLAFWGLPQIISKQIHKQTELTENTDQWDRFKELPFPMEFNIRFFLVTNPADVLNGSMPILKESEPYKYKSTIKRTDIRFDDIEEDSVTYRRSFSFEFDGSGTTREDDSITVINPLLMASFQLTNDIQRLAMAGCRKYILEPAGLDQVFLTTTVRKLLFDGIYFGFQNATGKGVACEMVRKELGKIVANVRVVEHLNDTDCYRLAIFNYKTDNFLKNSPDGIYTINRGRNNATALGSIMRWNGATTSTTYGTSTSINNLTCHSIKGTDSTIYSPELKAGENLMIFNTDLCRTIQLVQVSSNEVFNGINAFRYSTGYTLFRPETILKENDCYCSHGTKGADGKPSCFLDGLLDFRPCLGAPVLISQPHFLHADVKYIRAVSGLSPDEDKHDIYLLLEPNTGTPLEGRKRVQMNSVLRRQPLLSMITPPNMYEAVVPLLWLDEGFTLPQKYLDDLNAKYFKTVRIATGFKFGFIAVALALLVGCLFVACRKMYFRNAK

>ItypSNMP1

MPHPKNIAWAGGALAFGGVLFKVWLFDVLVRFGVKDQTALRYRNEVRGIYLKIPFPLNFKIYFFNVTNPEEIQNGAKPVLNEVGPYWYDEYKERVDVIDNDTEDSLTYTPYDLFKFNPNMSTPLSDNDYVTIIHPVIVGMVNLLLRDSPMLLKVVSKAIPFIFNDPKTIFLTGRVKDILFDGVVLNCTSKEFASTAVCGQMKGQVPGLKPTPGQPNLLLFSLLGPRNATRTGSLKVLRGIKHFQDLGRLLEVNGRKSIGIWAGDQCNRYDGTDSWIFPPLIQPESGLKSFSTDLCRNIKMKLVNETVVKKIPVGVFEPTWGVKVVTRRKSATVPTLPVXXXXXVFDLTKCMGVPLYATLPHFLDTDPNYLKLVDGLKPDHEKHRIVVFFETMTGTPLKAAKRMQFNLELQQTNKLELFSKLPAALFPIFWLEEGMELEGYFLKKIQTVFMLLLFADVTIYVTIATGLSVCGAGFYQYWKNTKSLSITPLTKNNNGLSEPKLN

>ItypSNMP1a

DLFKCAGVPLYASAPHFYDCHVSYLKGVRGLHPDEQKHAIKILFESLTGSPVYAKKRLQFNMPLEPNQKIDMFKNITPTVLPLFWIEEGVKLNNTYTKPLKSLFMMKKIVGVVKYLILLGSIAGVTVGVYLYFKSGDTVNVQ

>ItypSNMP2

MRFLQRVKFNLKTVFLCGISGVSLLVVALFLGFIIFPKVVNDQLLETKILREDTEQWAIFKKIPFAFTFNVYLFTVENPEEILKGAKPVVKEKGPYVYKLYKWKEDIIWNYTTDEISYYEYEKYVFDQEASGSLTEHDKVTLLNLPYLTFLYTAEANEATSGFLPLIDEALEFIFSGHNSPFLVNVTVRDYLFEGVEICKNGCEDDGFVAKMACGKIKDNLKVAKQMRLHHKDILFATFHYRNNTHQKYLTVNSGRQNHLEIGAITQLDNSSTMNVWNQFGCNQVSGLTGIFPINLGFKTTFQSFSAEICRPVKLHFSTIKPFGSIKGYKYVALNTTFNTSMVENQCYCTGKIPNLDGNLGCLYDGVLDLSTCLGAPIVVSFPHFLYADWRYVNNVKGLSPNETNHQIFVNLEPISGTPLEAATRIQFNLFLRPVRNITSLDSVADALVPLFWIEELTYLPQKYQDVITGKLYRSIFILNAIKYVLLAIALVIITVCILIFLYTD

>AmelSNMP1

MKPKKLGIIGGSLLAFGILICAIAFPPFLRSQVKKQIALKDGSEMRELWSNFPVPLDFKIYLFNVTNPMEITAGEKPILEEVGPFFYDEYKQKVDLVDREEDDSLEYNLKATWFFNPSRSEGLTGEEELIVPHVLILSMIKLTLEQQPAAMGILNKAVDNIFKKPESVFVRAKAREILFDGLPVDCTGKDFASSAICSVLKEKDDALIADGPGRYLFSLFGPKNGTVLPERIRVLRGIKNYKDVGKVTEVNGKTKLDIWGEGDCNEFNGTDSTIFAPLLTEQDDIVSFAPDICRSMGARFDSYTKVKGINTYHYKADLGDMSSHPEEKCFCPSPDSCLTKNLMDLTKCVGAPLIASLPHLLGAEEKYLKMVDGLHPNEEEHGIAMDFEPMTATPLSAHKRLQFNLYLHKVAKFKLMKNFPECLFPIFWVEEGILLGDEFVKKLKTVFKTISIVGFMKWFTIVSGTCVSGAAAALFFKNKDKNKLDITKVTPQKGEEKKWPNQMTISTIQSAAVPPNLDAD

>AmelSNMP2

MPDSTLFDMKPSDGLFSIKNAILKNLPLIKGKDMYDEWILPVNLIFKCYFFNVTNPDEVMEGNNPNLVEYGPFTYREVFEKQIVDVDEELDEIIYDVKSTFTFDKYASLNISKRDTVTILNPAYIGTISMLTTLPPSYIEKFVKLTCNERKFPELSTICKTLKALRSPVLKEGEKEGVYYLSIFQRVNGTIRGRFSVNRGVNNISELGNIGSYNGRRVQTIWRTEKCNTVRGSDTITWAPLINPMPSVLSFIPDLCRSIEADYDKEVSIYGLIGSRFVMRERTWFLNQSQCYCLERNKVPNCLPQGLIDVSDCLKVPIIMSEPHFLHGDPQLLMYALGLNPSEDLHETFIVIEPYTGTPLSGQKKIQLNLKLERQPVDLLSNISEGYFPLLWCANGNTPDLSVIILTFQLLRLVKLIKFIDVIPLIIGIHMTIVAMLYCNCKKRKRQPTISIADSLLISSNSNNAHRST

>ApisSNMP2

MHNKKGKLCTKLTSSFLRKWWVVLAIAVALLVGGLLVVAFFTAAVELVIDDQITLRPGSQTFEMWRKPPVRPLLKVYVYNVTNADEFLNVVAPGEVREKPILDELGPFVYVETWEKVNLTFHDNGTLTYNQQKVYRFDPEQSVGSEDDIVVVPNIPMLSATSQSKHAARFLRLAMASIMDILKVKPFVEVSVGQLLWGYEDPLLKLAKDVVPKEQKLPYEEFGLLYGKNGTSRDNMTVFTGATDIRLFAALDKFNGRTHLPHWTTDSCNRMSGGSDGSLFPPRIQPDTILHVFDKDMCRKLPLVFKKQVEAKGGVKAYRFGPDHRAFADPDHEPENRCYCPSLMNSPQCAPHGTFNVSLCQYDSPVLLSFPHFYMGDPRLREAVSGMDEPDADRHEFYIDVQPEMGVAMRARARVQINLAVSQVVDIKQVATFPDIVFPIMWFEEGIDELPDNVIQLLKLATQTPPVAKAALQYALFASGAVLLLLALGCLVRNSHRQETMSLEGTAHYSQDEKKKPKKTIPPAPLNSTVLSNGNGTAAKANAGYVADDE
